# Chemically Stabilized DNA Barcodes for DNA‐Encoded Chemistry

**DOI:** 10.1002/anie.202104348

**Published:** 2021-08-03

**Authors:** Marco Potowski, Verena B. K. Kunig, Lukas Eberlein, Alexandros Vakalopoulos, Stefan M. Kast, Andreas Brunschweiger

**Affiliations:** ^1^ TU Dortmund University Faculty of Chemistry and Chemical Biology Medicinal Chemistry Otto-Hahn-Strasse 6 44227 Dortmund Germany; ^2^ TU Dortmund University Faculty of Chemistry and Chemical Biology Physical Chemistry Otto-Hahn-Strasse 4a 44227 Dortmund Germany; ^3^ Medicinal Chemistry Bayer AG Aprather Weg 18a 42096 Wuppertal Germany

**Keywords:** DNA-encoded chemistry, DNA-encoded libraries, multicomponent reactions, solid-phase chemistry, stabilized DNA barcodes

## Abstract

DNA‐encoded compound libraries are a widely used small molecule screening technology. One important aim in library design is the coverage of chemical space through structurally diverse molecules. Yet, the chemical reactivity of native DNA barcodes limits the toolbox of reactions for library design. Substituting the chemically vulnerable purines by 7‐deazaadenine, which exhibits tautomerization stability similar to natural adenine with respect to the formation of stable Watson–Crick pairs, yielded ligation‐competent, amplifiable, and readable DNA barcodes for encoded chemistry with enhanced stability against protic acid‐ and metal ion‐promoted depurination. The barcode stability allowed for straightforward translation of 16 exemplary reactions that included isocyanide multicomponent reactions, acid‐promoted Pictet–Spengler and Biginelli reactions, and metal‐promoted pyrazole syntheses on controlled pore glass‐coupled barcodes for diverse DEL design. The Boc protective group of reaction products offered a convenient handle for encoded compound purification.

## Graphical abstract

DNA‐encoded compound libraries (DELs) are a widely used screening technology. DNA reactivity limits the reaction scope for library synthesis. Substituting the purines by 7‐deazaadenine yielded functional DNA barcodes with enhanced chemical stability. Controlled pore glass‐coupled barcodes tolerated isocyanide multicomponent reactions, the acid‐promoted Pictet–Spengler reaction, and Yb^III^‐ and Au^I^‐mediated pyrazole syntheses for DEL design.

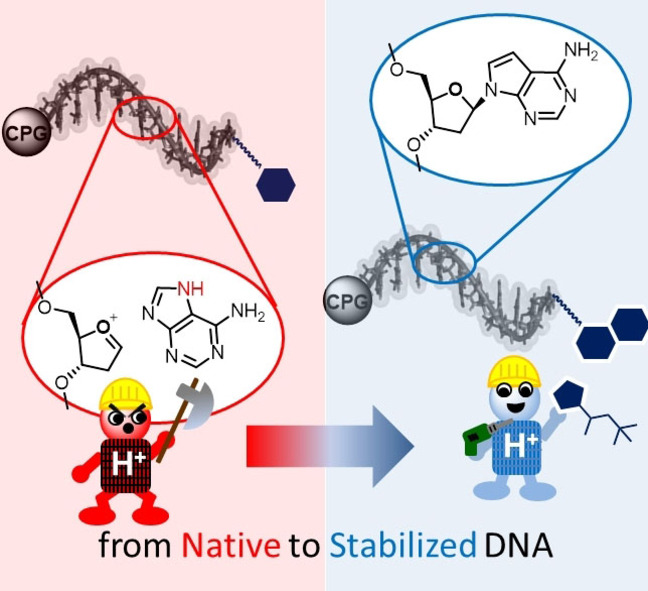

DNA‐encoded libraries (DELs, Figure 1 A,B) are widely used for compound screening on protein targets (Figure 1 B).^[1]^ The technology combines efficient compound handling with selection‐based screening. Thus, it is highly attractive for de novo library design and scanning protein surface with chemical space.^[1]^ Solution phase DEL synthesis, the most common DEL format, requires reactions that tolerate aqueous solvents, are robust, and yield well‐defined product mixtures. Reaction conditions must avoid damage to the barcode by, for example, acid‐ or metal‐promoted depurination (Figure 1 C), metal ion‐promoted deamination and purine oxidation, or by nucleophile addition.[^2^, ^3^, ^4^] Currently, efforts are dedicated to diversifying DEL design with a broad chemistry toolbox.[^5^, ^6^, ^7^, ^8^, ^9^, ^10^, ^11^] Solution‐phase DEL synthesis can be initiated on a controlled pore glass (CPG) solid phase.[^12^, ^13^, ^14^, ^15^, ^16^] A DEL strategy based on a CPG‐coupled hexathymidine adapter “hexT” avoided most DNA damage reactions but required encoding of individual hexT‐conjugates.[^15^, ^16^] Here, we explored replacing the hexT by DNA barcodes that should enable a similar scope of reactions, yet on encoded mixtures of starting materials, to increase synthesis efficiency. Experimental evidence gained in the context of encoded solid phase chemistry^[17]^ and synthetic biology^[18]^ suggest the substitution of the more vulnerable purines^[3]^ by 7‐deazaA (7De‐dA, **1**; see literature summary in the SI), while the 8‐aza‐7‐deazaA (7De8a‐dA, **2**) had to be explored. In combination with the CPG approach, such three‐letter codes are stabilized against depurination and protected against deamination; chemistry development benefits from free solvent choice, and products can be purified to improve library quality. Nucleobase tautomer stability for reliable formation of Watson–Crick base pairs is a precondition for unambiguously reading the DNA barcode by DNA polymerases at the PCR amplification step prior to barcode sequencing.

**Figure 1:**
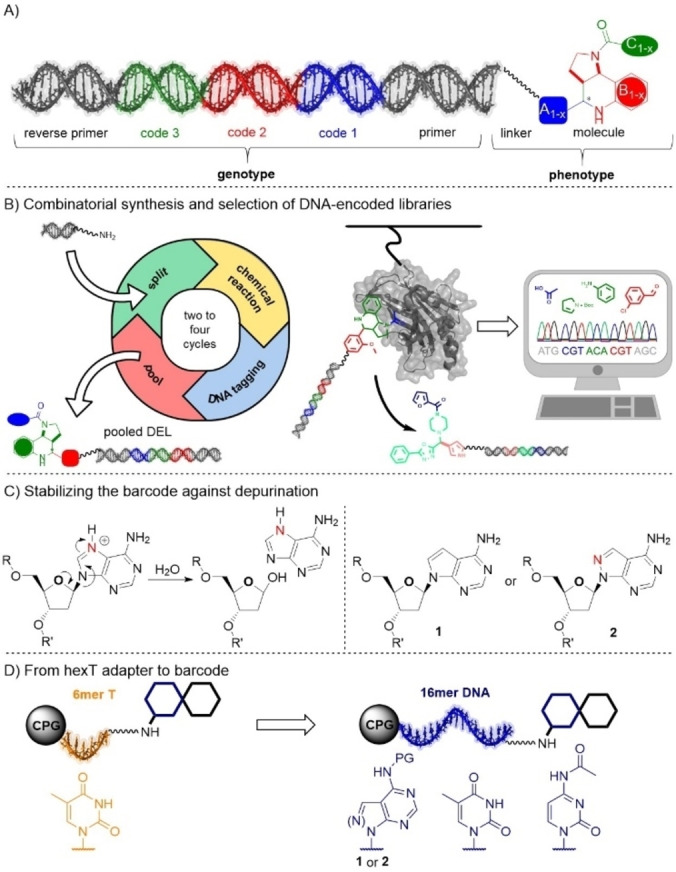
The technology of DNA‐encoded libraries. A) A DNA‐encoded compound. B) Combinatorial DEL synthesis and screening by selection. C) DNA damage by depurination. The nucleosides 7‐deazaA **1** and 8‐aza‐7‐deazaA **2** are studied for use in encoded chemistry. D) From hexT to a chemically stabilized code. The code consists of nucleobases T, C, and either 7‐deazaA **1** or 8‐aza‐7‐deazaA **2**. PG, protective group: benzoyl (**1**) or DMF (**2**).

Nucleobases that display more than one stable tautomer exhibit different protonation patterns, which can cause mismatches, potentially resulting in wrong barcode assignment. Such a phenomenon has already been observed with non‐natural nucleobases, like the Hachimoji code^[19]^ in which the isoguanine is not tautomer stable.^[20]^ Here, using the computational methodology validated earlier,^[20]^ we investigated all possible tautomers of adenine **Ia–c** as well as 7‐deazaadenine **IIa–c** and 8‐aza‐7‐deazaadenine **IIIa–c** (Table 1). According to this analysis, the reaction free energy between Watson–Crick pair‐forming and both alternate tautomers (Table 1), which translates into tautomer populations, was reduced in **II** and **III** compared to natural adenine. However, the smallest energy difference was still more than 7 kcal mol^−1^, resulting in negligible mismatching tautomer fractions. The validity of the computational model is further supported by p*K*_a_ calculations for protonation of the mismatch‐relevant nitrogen position 1 (see SI for details), yielding 1.83 and 3.20 for compounds **I** and **II**, respectively. Corresponding macrostate p*K*_a_ values that account for all tautomers in the protonated state are 1.84 and 3.20, which indicates that protonation occurs predominantly at position 1. These results, with a p*K*_a_ difference of 1.36 between **II** and **I**, are in line with the experimental difference of 1.8 (**I**: 3.5, **II**: 5.3 for respective nucleosides).^[21]^ Hence, we conclude for all nucleobases that at pH values at which Taq DNA polymerase and ligases operate, the Watson–Crick tautomer is the dominant species with a population of more than 99.99 %. From the tautomer perspective, DNA oligonucleotides containing either 7De‐dA or 7De8a‐dA should both be well suited for barcoding chemistry.

**Table 1:** Calculated standard reaction free energies Δ*G* (kcal mol^−1^) and populations for selected tautomeric forms of adenine derivatives **I**–**III** relative to the Watson–Crick tautomers [**I**–**IIIa**]. 
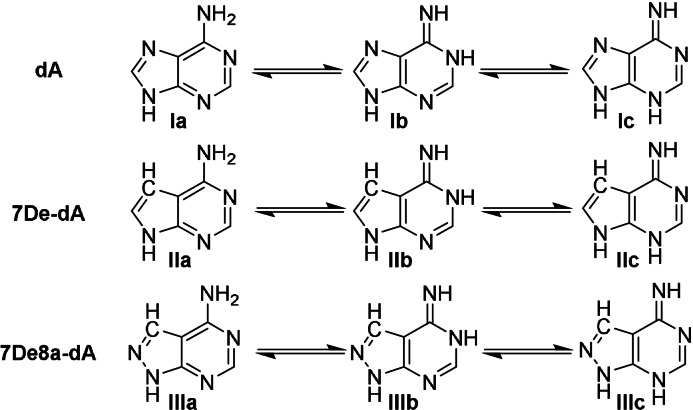

Tautomer	Average Δ*G*	Population
**Ia**	0.00	>0.9999±4.72×10^−8^
**Ib**	9.82±0.44	6.31×10^−8^±4.72×10^−8^
**Ic**	19.26±2.19	7.66×10^−15^±2.83×10^−14^
		
**IIa**	0.00	>0.9999±5.64×10^−7^
**IIb**	8.51±0.59	5.69×10^−7^±5.64×10^−7^
**IIc**	16.60±1.02	6.75×10^−13^±1.16×10^−12^
		
**IIIa**	0.00	>0.9999±2.82×10^−6^
**IIIb**	7.24±0.34	4.96×10^−6^±2.82×10^−6^
**IIIc**	14.43±0.65	2.66×10^−11^±2.91×10^−11^

A stability screen of CPG‐coupled 10mer DNAs **3**–**6** against metal salts, organic reagents, and protic acids confirmed that the pyrimidine‐DNA **3** tolerated most reagents, while native DNA **4** was degraded by acids, oxidants, and certain metal ions, as previously reported (Table 2).^[3]^ To our delight, both 7De‐dA‐ or 7De8a‐dA‐modified 10mer codes **5** and **6** mirrored the stability profile of pyrimidine DNA, and we did not notice differences in stability between adenosine analogues **1** and **2**. Therefore, we focused on the more readily available 7De‐dA **1** for development of the DEL strategy (Figure 2 A). Beside the increased DNA stability, ligation of chemically modified DNA barcodes by T4 ligase and correct reading of the resultant template by DNA polymerases are indispensable for functional compound barcoding. For DEL synthesis, DNA oligonucleotides were designed that consisted of an 8mer code and terminal 4mer overhangs (Figure 2 A) for ligation, and the linker for compound attachment was introduced at the 5′‐end ‐1 position.^[22]^ Furthermore, we introduced a universal adapter as a counter code for the stabilized DNA codes. Chemically stabilized DNA barcodes were ligated at the 5′‐terminus to a hairpin that contained the forward primer sequence, and at the 3′‐terminus to further compound identifier barcodes (Figure 2 B, Figure S1 and Table S3).^[21]^ To our delight, amplicon sequencing of ligation products **12 a**–**e** confirmed that they could be copied by Taq polymerase with high fidelity, experimentally supporting the tautomer population calculations (Figure 2 B, Figure S2, Table S4). Next, we compared the amplification efficiency of 7De‐dA‐containing ligation products with one native DNA ligation product by qPCR (Figure 2 C and Figures S3–S5). The chemically modified template required 2–4 cycles of enzymatic template copying more to reach the detectable log‐linear phase of amplification at all tested template concentrations. Having reached the threshold, both PCRs proceeded with comparable efficiency in the log‐linear phase. The ligation, amplification, and sequencing results demonstrated that chemical stabilization of the DNA results in a viable novel barcoding technology.

**Figure 2:**
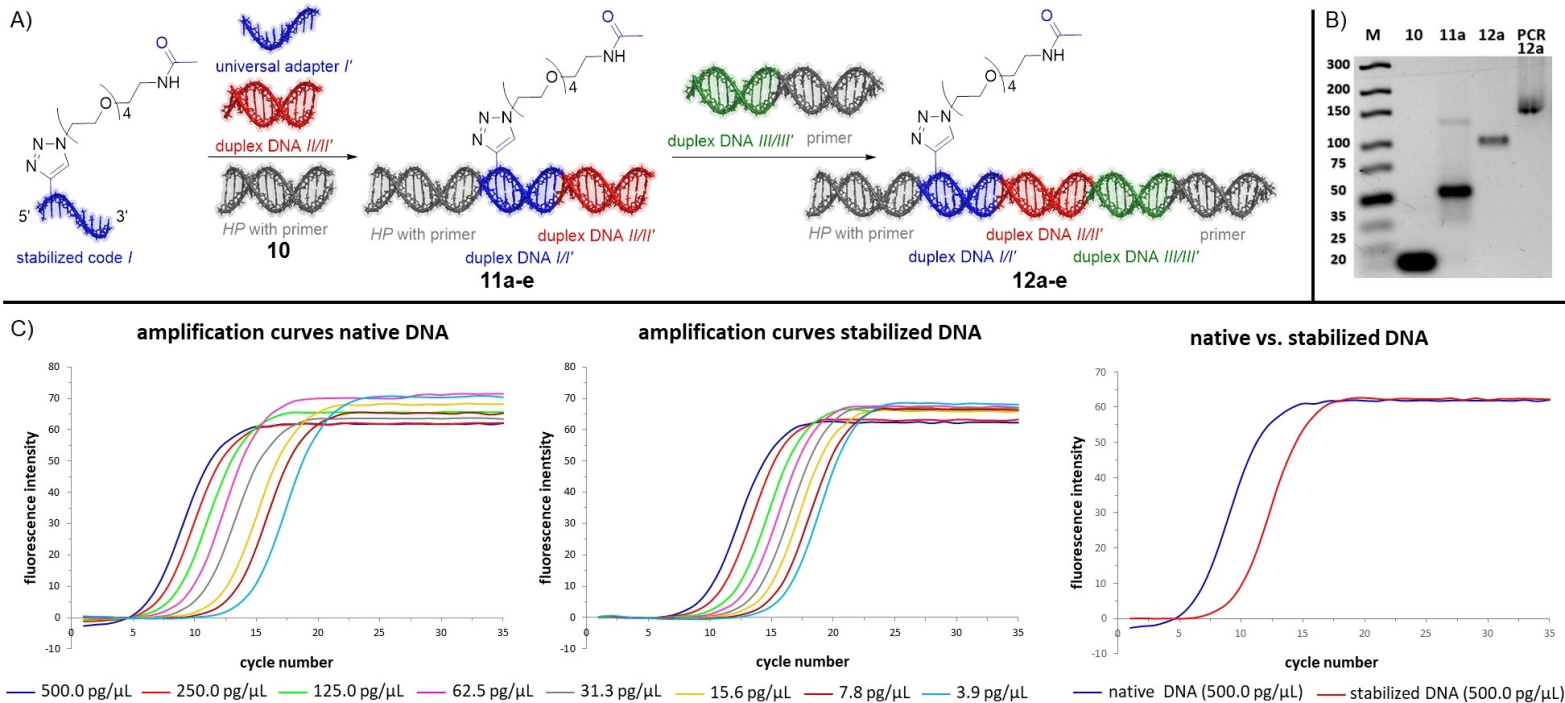
A ligation strategy for chemically stabilized DNA barcodes. A) Schematic presentation of the DEL strategy. B) Analysis of T4 ligation and PCR amplification products. C) Analysis of the amplification efficiencies of a chemically stabilized DNA template with a native DNA template strand.

**Table 2:** Chemical stability screening of DNA barcodes.^[a]^ 
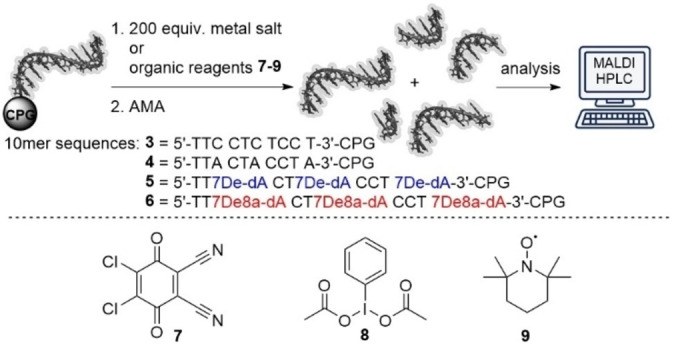

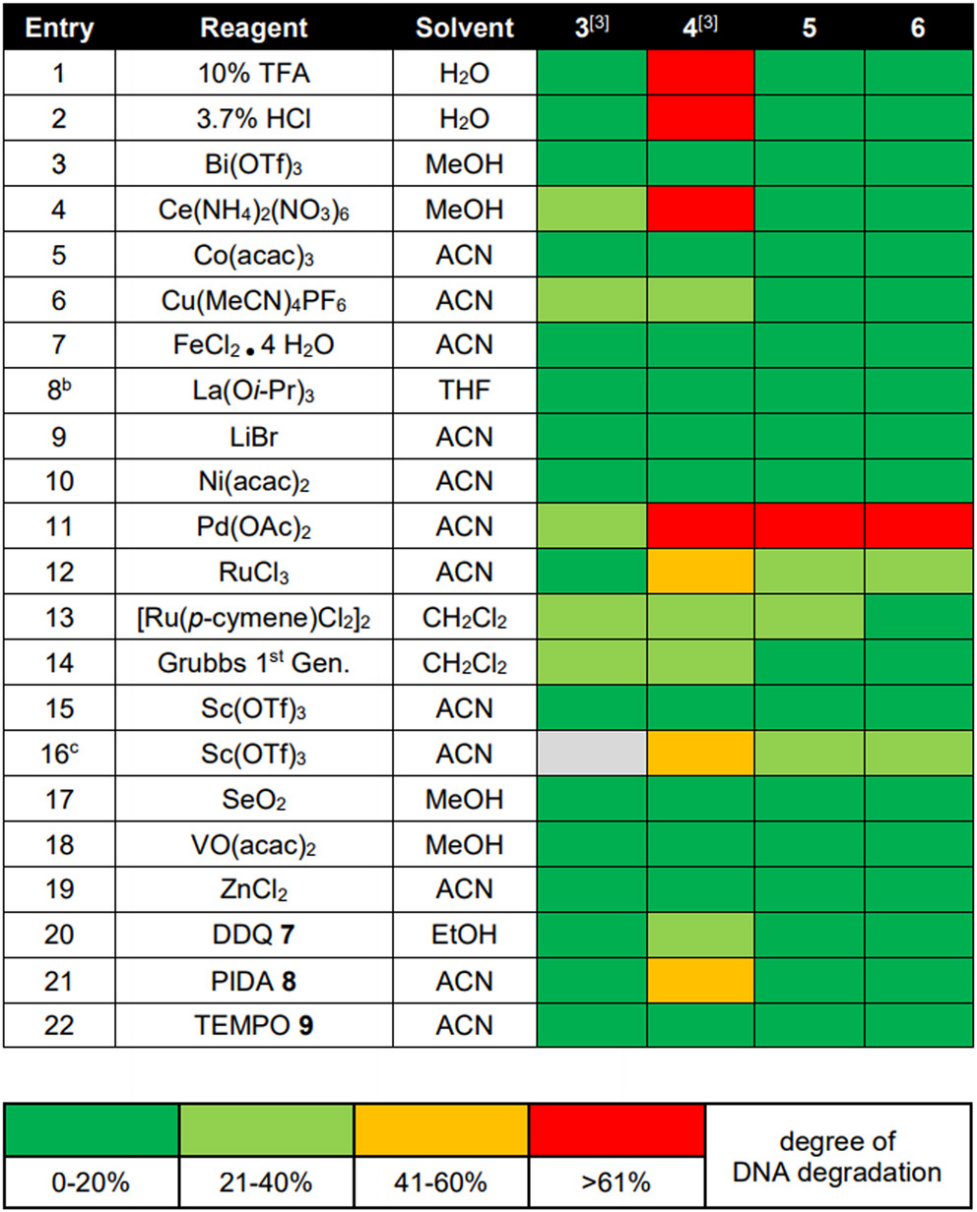

[a] 20 nmol DNA, aqueous acids, or 200 equiv transition metal salt or organic reagents, 50 μL solvent, rt, 22 h. [b] Added as suspension. [c] 40 °C. ACN=acetonitrile, MeOH=methanol.

Previously, several reactions for CPG‐initiated DEL synthesis were shown to require the hexT adapter while others could be performed on a very short model 10mer native DNA.[^15^, ^16^, ^21^, ^22^, ^23^, ^24^] The 7De‐dA‐containing three‐letter barcodes will enable one unified barcoding strategy to synthesize scaffold structures as second synthesis cycle on encoded, pooled starting materials. Here, we explored the compatibility of 16 reactions including isocyanide MCRs, Brønsted acid‐ and metal ion‐promoted reactions with CPG‐coupled 7De‐dA‐DNA‐encoded starting materials (Figure 3). Four isocyanide‐based multicomponent reactions were tested on the stabilized DNA barcode (Figure 3 B–E).^[23]^ The Ugi four‐component reaction (U‐4CR), Ugi‐azide four‐component reaction (UA‐4CR) and the Groebke‐Blackburn‐Bienaymé three‐component reaction (GBB‐3CR) could previously be performed with broad scope on short 10mer native model DNA oligonucleotides in contrast to the Ugi‐aza‐Wittig four‐component (U‐4CR/aza‐Wittig) reaction which required the hexT adapter. The U‐4CR, UA‐4CR, and GBB‐3CR could be performed smoothly with a model substrate set on a CPG‐bound 16mer 7De‐dATC‐DNA‐encoded aldehyde **13** leading to nearly quantitative product conversions with less than 5 % DNA degradation (Figure 3 B–D). The U‐4CR/aza‐Wittig reaction on **13** resulted in moderate conversions (32–49 %, Figure 3 E and Table S10). Still, DNA degradation was observed, possibly due to the highly nucleophilic isocyanide reagent. However, the reaction with a same set of reactants showed a slightly higher degree of DNA degradation for the native 10mer ATGC‐DNA (32 %) compared to the stabilized barcode (24 %, Table S9). In addition to the acetic acid‐promoted GBB‐3CR, we investigated also the Brønsted acid‐mediated Biginelli, Povarov,^[3]^ and Pictet–Spengler reactions,[^15^, ^22^] as well as Boc chemistry on the stabilized barcode (Figure 3 F–I). The 10mer‐DNA‐compatible (*R*)‐(−)‐BNDHP‐mediated Biginelli and Povarov reactions^[3]^ were readily transferred to the DNA barcode with excellent conversions and without any DNA degradation (Figure 3 F,G). A Boc‐protected product **31** of the Povarov reaction could be deprotected with 10 % TFA (Figure 3 H). The transfer of the TFA‐mediated Pictet–Spengler reaction that caused massive DNA damage[^22^, ^26^] onto the 16mer 7De‐dATC‐tryptophan conjugate **33** led to complete conversions of a broad scope of aldehydes **34 a**–**s** to the desired products **35 a**–**s** with only low degrees (<5–17 %) of DNA damage (Figure 3 I and Table S11). Next, we explored the compatibility of several metal‐mediated reactions with the chemically stabilized DNA‐barcode (Figure 3 J–Q). The DNA‐compatible Zn^II^‐mediated Diels–Alder^[3]^ and Cu^I^/bpy‐mediated Petasis^[24]^ reactions as well as the Ag^I^‐mediated azomethine ylide 1,3‐dipolar cycloaddition^[22]^ and Yb(OTf)_3_‐mediated Castagnoli–Cushman reaction[^22^, ^27^] could be readily translated to the stabilized barcode with conversions of 49–90 % and no detectable DNA degradation, arguing for the robustness of the CPG‐based DEL approach (Figure 3 J–M).

**Figure 3:**
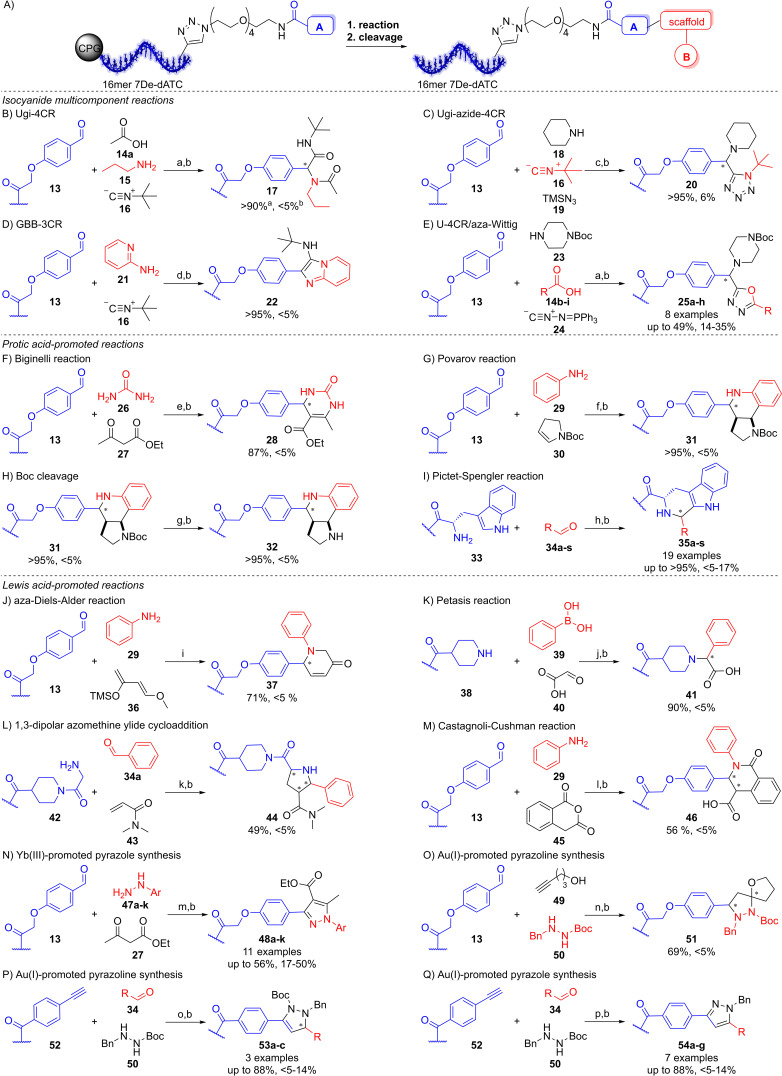
Translation of reactions to DNA‐encoded starting materials. A) Reactions were performed on a CPG‐coupled 16mer DNA. B–E) Isocyanide MCR chemistries; a) MeOH, 50 °C, b) aq. NH_3_/MeNH_2_, c) trimethylsilylazide, MeOH, 50 °C, d) 1 % acetic acid/MeOH, 50 °C. F–I) Protic acid‐promoted reactions; e) (*R*)‐(−)‐BNDHP, EtOH 50 °C, f) (*R*)‐(−)‐BNDHP, EtOH/TEOF, 50 °C, g) 10 % TFA, CH_2_Cl_2_, rt, 4 h, h) 5 % TFA, CH_2_Cl_2_, rt, 20 h. J–Q) Metal ion‐promoted reactions; i) ZnCl_2_, ACN/TEOF, rt, then aq. NH_3_, 50°C, 6 h, j) CuCl/bpy, DMF/TEOF, 50 °C, k) AgOAc, ACN/TEOF, 50 °C, l) Yb(OTf)_3_, CH_2_Cl_2_/TEOF, rt, m) Yb(PFO)_3_, toluene, 50 °C, n) Au^I^/AgSbF_6_, THF, rt, o) *aliphatic aldehyde*: Ipr AuCl/AgOTf, ACN, 50 °C; *aromatic aldehyde*: Ipr AuCl/AgOTf, glacial acetic acid, 50 °C, p) Au^I^/AgOTf, glacial acetic acid, 60 °C. ^a,b^ Conversion and DNA degradation determined by HPLC. Au^I^=[Tris(2,4‐di‐*tert*‐butylphenyl)phosphite]gold chloride.

We then investigated the compatibility of reactions employing potentially DNA‐damaging aryl hydrazines. DNA‐encoded aldehydes **13**, arylhydrazines **47 a**–**k**, and ethyl acetoacetate **27** can be reacted with Yb(PFO)_3_ to diverse substituted pyrazoles **48 a**–**k** under harsh reaction conditions (Figure 3 N, Tables S12–S14).[^28^, ^29^] This reaction caused massive, 69 % degradation of the 10mer model DNA (Table S13). On the CPG‐coupled stabilized DNA, this reaction resulted in only 33 % DNA damage (Figure 3 N, Table S13). However, also on a CPG‐coupled 10mer pyrimidine‐DNA‐aldehyde conjugate 22 % of DNA degradation was observed under these conditions (Table S13). The DNA damage may be partially attributed to reaction of the highly nucleophilic arylhydrazines with the DNA. This is supported by the observation that electron‐rich hydrazines caused higher levels of DNA damage (up to 50 %) even to the stabilized DNA barcode (Table S14). Au^I^‐mediated pyrazoline[^15^, ^25^] or pyrazole^[15]^ syntheses caused high degrees of damage to native DNA (Figure 3 O–Q). However, the Au^I^‐mediated reaction of 16mer 7De‐dATC aldehyde conjugate **13** with alkynol **49** and hydrazide **50** towards a spiroheterocycle worked smoothly with 59 % conversion (Figure 3 O). The reaction of stabilized DNA alkyne conjugate **52** with aliphatic aldehydes and benzaldehyde **34** and hydrazide **50** mediated by Ipr AuCl/AgOTf led to the desired pyrazolines **53 a**–**c** with high conversions (up to 88 %) and low DNA degradation (<5–14 %, Figure 3 P and Table S15). Surprisingly, even the Au^I^‐promoted pyrazole formation in glacial acetic acid at 60 °C (!) was tolerated by the chemically stabilized 7De‐dATC sequence and resulted in the products **54 a**–**g** with up to 88 % conversions and low DNA damage (<5–14 %, Figure 3 Q and Table S16). The fully ligated chemically modified and native control DNA oligomers tolerated amide bond formation and Suzuki reaction conditions as exemplary standard DEL reactions for a plausible third synthesis cycle well, giving evidence for the viability of the novel barcoding strategy (Figures 4, S11–S14).

**Figure 4:**
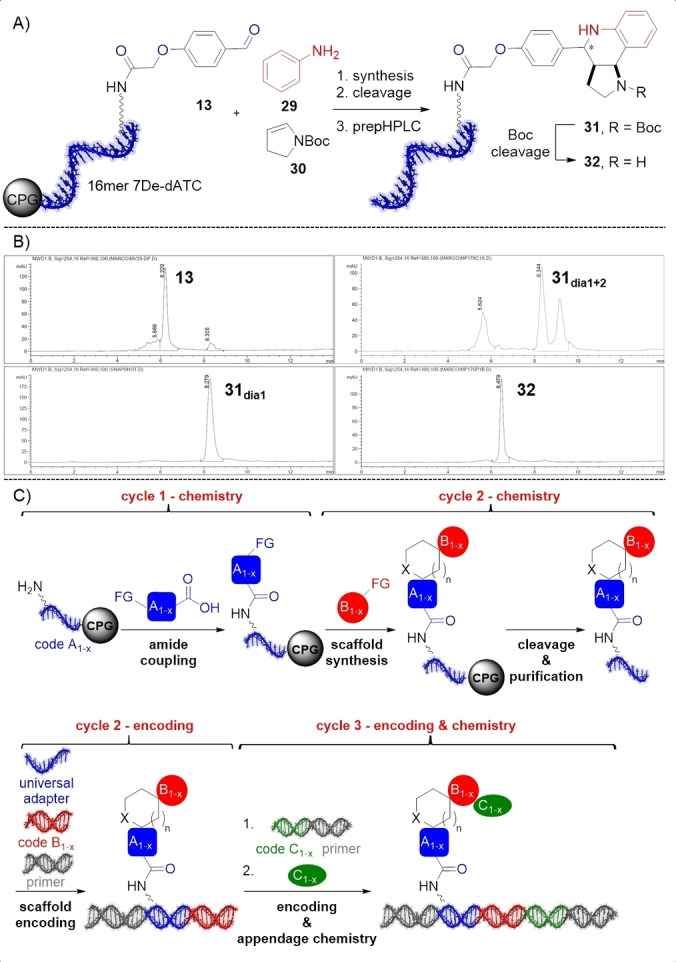
Barcoding and compound purification strategy. A) Povarov reaction on a barcoded aldehyde (Figure 3 H), and Boc chemistry for purification; B) HPLC analysis of products. C) Generic library strategy.

Importantly, on‐DNA reactions can lead to substantial retention time shifts of DNA‐product conjugates in ion pair chromatography.[^13^, ^14^, ^15^] Playing in our hands, the Boc protective group facilitated product purification and was cleaved from two purified, exemplary Boc‐protected DNA‐conjugated heterocyclic compounds **25 a** and **31** in aqueous solution with 10 % TFA for 4 h (Figure 4 and Figure S15). The desired deprotected Povarov and U‐4CR/aza‐Wittig products **32** and **55** were obtained without noticeable DNA degradation and can be used for further DEL synthesis (Figure 4 C, Figure S15 and detailed practical information in SI).

In conclusion, we demonstrate here a DEL technology that uses chemically stabilized, functional three‐letter DNA barcodes composed of T, C, and 7‐deazaadenine. These still showed vulnerability to hydrazines as examples for strong nucleophiles, but notable stability against protic acids and metal ions. In line with the current trend to increase the structural diversity of encoded libraries,[^8^, ^9^, ^10^, ^11^, ^29^, ^30^] this newly developed barcoding strategy allowed for translation of sixteen reactions for diverse DEL design, furnishing diverse scaffold structures. Among them were the U‐4CR/aza‐Wittig reaction, the TFA‐promoted Pictet–Spengler reaction, or Yb^III^‐ and Au^I^‐promoted pyrazole syntheses which were performed under harsh reaction conditions that can hardly be reconciled with native DNA integrity. Conveniently, the Boc group of encoded products can be used as a handle for purification, enhancing fidelity in a DEL synthesis. Translation of a larger reaction scope, investigation of the reaction scope in solution phase which would allow performing reactions under basic conditions, and screening of DELs synthesized by this new barcoding technology on protein targets will be reported in short time.

## Conflict of interest

A.B. is co‐founder of the company Serengen GmbH that provides DEL technology services. Data presented in this manuscript is included in the patent application EP 20 166 145.1.

## Supporting information

As a service to our authors and readers, this journal provides supporting information supplied by the authors. Such materials are peer reviewed and may be re‐organized for online delivery, but are not copy‐edited or typeset. Technical support issues arising from supporting information (other than missing files) should be addressed to the authors.

Supporting Information
