# Supplementary material for: Chemically Stabilized DNA Barcodes for DNA‐Encoded Chemistry
Source: Angew Chem Int Ed Engl. 2021 Aug 3;60(36):19744–9. doi: 10.1002/anie.202104348 (PMC8456907; doi:10.1002/anie.202104348)
Supplement: Supplementary file 1 — Supporting Information [file ANIE-60-19744-s001.pdf]

Angewandte Chemie
Eine Zeitschrift der Gesellschaft Deutscher Chemiker

Supporting Information

Chemically Stabilized DNA Barcodes for DNA-Encoded Chemistry

Marco Potowski[+], Verena B. K. Kunig[+], Lukas Eberlein, Alexandros Vakalopoulos, Stefan M. Kast, and Andreas Brunschweiger**

anie_202104348_sm_miscellaneous_information.pdf

Table of Contents	Page

**General methods and materials**	S5
**Biochemical and -physical characterization of DNA oligonucleotides modified with 7De-dA**	S7
DNA duplex formation and duplex stability	S7
Mismatch discrimination by 2'-deoxy-7-deaza-adenosine	S7
Tolerance by DNA polymerases	S8
**Computational investigations of nucleobase tautomer stability**	S9
Computational details	S9
Calculation of tautomer populations	S10
**Synthesis of chemically stabilized nucleoside phosphoramidites A and C**	S15
NMR spectra	S18
**Chemical stability screening of DNA barcodes**	S22
Representative procedures	S22
HPLC traces and MALDI-MS spectra	S24
**Biological experiments**	S46
T4 ligation and amplification by PCR	S46
Results from Sanger sequencing	S48
Results from qPCR	S52
**DNA-encoded chemistries**	S55
Representative procedures	S55
HPLC traces and MALDI-MS spectra	S73
CPG-bound DNA-starting material conjugates	S73
Isocyanide multicomponent reactions	S80
Ugi four-component reaction	S80
Ugi-azide four-component reaction	S81
Groebke-Blackburn-Bienaymé three-component reaction	S82
Ugi four-component/aza-Wittig reaction	S83
Brønsted acid-mediated reactions	S95
Biginelli reaction	S95
Povarov reaction	S96
Boc cleavage on solid phase	S97
Pictet-Spengler reaction	S98
Lewis acid-promoted reactions	S120
aza-Diels-Alder reaction	S120
Petasis reaction	S121
1,3-dipolar azomethine ylide cycloaddition	S122
Castagnoli-Cushman reaction	S123
Yb(III)-mediated three-component synthesis of pyrazoles	S124
Optimization	S124
Scope	S130

Au(I)/Ag(I)-promoted pyrazoline-containing spiroheterocycle synthesis S144
Au(I)/Ag(I)-promoted pyrazoline synthesis S145
Au(I)/Ag(I)-promoted pyrazole synthesis S150
Boc cleavage in aqueous solution S159

qPCR analysis after treating DNA with common DEL synthesis methods S161

Practical aspects of DEL synthesis S163
Phosphoramidite building block and DNA synthesis S163
Barcode design S164
DEL synthesis S164

References S165

Supplementary Scheme

Scheme S1 – Selected tautomeric forms of protonated adenine derivatives **IV-V** S10

Supplementary Figures

Figure S1 – Encoding scheme for ligations with chemically stabilized barcodes S48
Figure S2 – Agarose gel S48
Figure S3 – Amplification curves (qPCR) of DNA sequence containing barcode *Id* S52
Figure S4 – Standard curves (qPCR) of DNA sequence containing barcode *Id* S52
Figure S5 – Melting curves (qPCR) of DNA sequence containing barcode *Id* S53
Figure S6 – Amplification and melting curves (qPCR) of DNA sequence containing barcode *Ia* S53
Figure S7 – Amplification and melting curves (qPCR) of DNA sequence containing barcode *Ib* S53
Figure S8 – Amplification and melting curves (qPCR) of DNA sequence containing barcode *Ic* S54
Figure S9 – Amplification and melting curves (qPCR) of DNA sequence containing barcode *Ie* S54
Figure S10 – Differences in the amplification rate of different DNA sequences containing stabilized DNA barcodes (*Ia*-*e*) and native DNA (*Ia*) S54
Figure S11 – Encoding scheme for test ligations with chemically stabilized/native barcode *If* S161
Figure S12 – Amplification and melting curves (qPCR) of the ligation product containing stabilized DNA barcode *If* or its native analogue S162
Figure S13 – Amplification and melting curves (qPCR) of the ligation product containing stabilized DNA barcode *If* or its native analogue after treatment with amide coupling conditions. S162
Figure S14 – Amplification and melting curves (qPCR) of the ligation product containing stabilized DNA barcode *If* or its native analogue after treatment with Suzuki reaction conditions. S162
Figure S15 – Barcoding strategy using CPG-coupled chemically stabilized barcodes S163

Supplementary Tables

Table S1 – Calculated Δ*G* and populations for selected tautomeric forms of adenine derivatives S11
Table S2 – Results (in kcal mol^{-1} for energies) of EC-RISM and vacuum calculations S12

Table S3 – Results (in kcal mol^{-1}) of PCM (MP2/6-311+G(d,p)) and TI calculations S13
Table S4 – Results of MP2/6-311+G(d,p)/EC-RISM microstate pK_a calculations S14
Table S5 – Stability of chemically modified oligonucleotides **5** and **6** S23
Table S6 – Sequences of DNA oligonucleotides *I* – *IV*/*IV'* S46
Table S7 – Sanger sequencing results S49
Table S8 – Overview of diverse chemical reactions on CPG-bound stabilized barcode S70
Table S9 – HPLC traces for the on-DNA U-4CR/aza-Wittig reaction S83
Table S10 – Scope of the U-4CR/aza-Wittig reaction S84
Table S11 – Scope of the Pictet-Spengler reaction S98
Table S12 – Optimization of the Yb(III)-mediated three-component synthesis of pyrazoles S124
Table S13 – HPLC traces for the Yb(III)-mediated on-DNA pyrazole synthesis S129
Table S14 – Scope of Yb(III)-mediated three-component synthesis of pyrazoles S130
Table S15 – Scope of Ipr Au(I)/Ag(I)-mediated synthesis of pyrazolines S145
Table S16 – Scope of Au(I)/Ag(I)-mediated synthesis of pyrazoles S150
Table S17 – Investigated DEL synthesis methods S161

General methods and materials

Unless otherwise noted, chemicals were purchased from *abcr, Acros Organics, Alfa Aesar, Fisher Scientific, Merck, Sigma Aldrich, TCI* and *VWR* and were used as provided without further purifications. Dry solvents (ACN, CH_2Cl_2, DCE, DMF, EtOH, MeOH, THF, toluene) were used as commercially available.

5'-Aminolinker-modified DNA oligonucleotides on controlled pore glass solid support (CPG, 1000 Å porosity) were synthesized by *IBA* (Göttingen, Germany). The branched 16mer 7De-dATC-alkyne conjugates on controlled pore glass solid support (CPG, 1000 Å porosity) were synthesized by *Ella Biotech GmBH* (Martinsried, Germany). DNA hairpin and barcodes for ligation experiments were purchased from *Integrated DNA Technologies* (IDT).

The 10mer 7De-dATC- and 10mer 7De-8a-dATC-oligonucleotides for the stability screen were prepared by DNA solid phase synthesis on a DNA Synthesizer H-8 from *K&A Laborgeräte GbR* on a 1 µmol scale using standard CPG-based phosphoramidite DNA synthesis methods. The DNA phosphoramidites were used with the 4,4'-dimethoxytrityl (DMT) and β-cyanoethyl (CE) protecting groups (DMT-7De-dA-CEP, DMT-7De-8a-dA, DMT-dT-CEP, DMT-dC-CEP and DMT-Ethynyl-dUCEP). 5-(Benzylthio)-1H-tetrazole (BTT) was used as activator.

CPG with oligonucleotide-small molecule conjugates were filtered and washed through synthesis columns using a vacuum manifold (Vac-Man®) from *Sigma Aldrich*.

Oligonucleotide concentrations. Concentrations were determined by UV spectroscopy using a NanoDrop 2000 spectrophotometer from *Thermo Fisher Scientific*.

Semi-preparative ion pair RP-HPLC. Compound purification was performed on a *Shimadzu Prominence* HPLC System equipped with a C_{18} stationary phase (*Phenomenex*, Gemini, 5 µm, C_{18}, 110 Å, 100 x 4.6 mm). A gradient from 100 mM aqueous triethylammonium acetate (pH = 8.0, eluent A) to MeOH (eluent B) was used at a flow rate of 5 mL/min. Fractions containing the desired product were pooled and concentrated.

> **Method:** Step gradient of 20% to 70% B within 13 min, then 70% to 100% B within 1 min followed by 100% B for 3 min using 100 mM aqueous triethylammonium acetate (pH = 8.0, eluent A) and MeOH (eluent B) at a flow rate of 5 mL/min.

Analytical RP-HPLC. HPLC analysis was performed on an *Agilent* 1100 series chromatograph equipped with 1100 Quaternary Pump (*G1311A*), a 1100 Multi-Wavelength Detector (G1365B) and an *Agilent* Eclipse Plus C_{18} (4.6 x 100 mm, 3.5 µm) column. The conversion and purity of DNA conjugates were determined by integration of peaks recorded at 254 nm wavelength.

> **Method:** Step gradient of 10% to 70% B within 10 min, then 70% to 100% B within 2 min followed by 100% B for 2 min using 10 mM aqueous triethylammonium acetate (pH = 8.0, eluent A) and MeOH (eluent B) at a flow rate of 0.6 mL/min.

MALDI-TOF. Mass analysis was performed on a MALDI TOF/TOF MS from *Bruker Daltonics* using 2',4',6'-trihydroxyacetophenone (THAP) matrix (*Dichrom*).

^{1}H-NMR and **^{13}C-NMR** were recorded on a Bruker AVANCE 500 (1H NMR, 500 MHz; 13C NMR, 126 MHz) or Bruker AVANCE 600 spectrometer (1H NMR, 600 MHz; 13C NMR, 151 MHz). Data are reported in the following order: chemical shift (δ) values are reported in ppm with the solvent resonance as internal standard (CD_2Cl_2: δ = 5.32 ppm for 1H, δ = 54.00 ppm for 13C) or relative to TMS (δ = 0 ppm); multiplicities are indicated s (singlet), d (doublet), t (triplet), q (quartet) m (multiplet); coupling constants (J) are given in Hertz (Hz).

The biochemical and biophysical characterization of DNA oligonucleotides modified with 2'-deoxy-7-deaza-adenosine (2'-deoxy-tubercidin, 7De-dA)

DNA duplex formation and duplex stability

A few reports investigated the base-pairing properties of DNA oligonucleotides modified with 7-deaza-adenine (7De-dA). Ingrid Luyten and Piet Herdewijn summarized early research on the substitution of adenine by 7De-dA in a seminal review on nucleobase modifications, and concluded from the literature that 7De-dA had mostly a destabilizing effect on the DNA duplex.[1] The research group of Bernard Connolly investigated systematically the effect of nucleobase modification on duplex stability and duplex conformation.[2] According to CD spectra, the substitution of dA by 7De-dA did not lead to perturbation of the helical structure of a dodecamer mixed-base duplex DNA. Melting curve measurements of the investigated sequence showed a nucleobase-context-dependent reduction (1 °C, 5 °C) of the melting temperature in the 7De-dA-modified DNA versus native DNA. This finding was reproduced by the groups of Martin Egli and Michael Stone.[3] They could show by CD spectroscopy, by NMR, and by x-ray analysis that replacement of dA by 7De-dA in the Dickerson-Drew dodecamer, a model oligonucleotide used by the research community for investigations in B-DNA structure, had minimal effect upon base pairing geometry and conformation of the dodecamer duplex. They attributed helix destabilization to a loss of enthalpic stabilization, which can be explained by decreased base stacking interactions.

Mismatch discrimination by 2'-deoxy-7-deaza-adenosine

The substitution of *N*-7 by carbon leads to an increase of nucleobase pKa, i.e. 7De-dA is more easily protonated (dA: pKa= 3.50, and 7De-dA: pKa= 5.30). Seela *et al*. found that 7De-dA lost the capability to discriminate between T and C in a counterstrand at lower pH values.[4] However, at higher pH values, which are required for DNA polymerases (pH 8-9.5), and are therefore relevant to the application as DNA barcode, 7De-dA showed the same mismatch discrimination as dA. This finding supports our Sanger sequencing results that did not show introduction of mutations by Taq polymerase.

Tolerance by DNA polymerases

The tolerance of the 7De-dA-modified template by at least one member of the diverse family of DNA polymerases from thermophilic organisms is vitally important to the application in DNA-encoded library technology, as DNA amplification is needed for subsequent barcode analysis by sequencing. The group of Andreas Marx could show by several x-ray analyses that family B DNA polymerases tolerate modifications in the 5-position of pyrimidines and in the 7-position of purines of dNTPs very well, and these structural analyses suggest that the 7-position can also be modified in the template strand.[5] Experimental evidence in agreement with these structural studies was independently delivered by the groups of Michael Famulok,[6] Piet Herdewijn,[7] Andrew Pike,[8] and Michal Hocek.[9] They demonstrated that DNA template sequences heavily modified in the 7-position of purines, and in the 5-position of pyrimidines, which is important for our linker strategy, are read with high fidelity by DNA polymerases in polymerase chain reactions.

Computational investigations of nucleobase tautomer stability

Computational details

Following the procedures outlined in Ref. 10, solution-phase structures were generated by manual construction of an exhaustive set of NH rotamers and optimized at the B3LYP/6-311+G(d,p)/IEFPCM level of theory with the default parameters for water as implemented in Gaussian 16 rev. C.01.[11] These structures were reoptimized in vacuum using B3LYP/6-311+G(d,p), using frequency calculations to confirm the structures as local minima and providing data for thermal corrections to yield gas-phase free energies. The vacuum-optimized structures were employed in MP2/6-311+G(d,p) calculations using Gaussian 16 for determining the gas-phase leg of the solvation free energy and in CCSD(T)/cc-pVTZ calculations using the ORCA software within the R1-F12 approximations for the gas-phase reaction energy baseline.[12,13,14] The PCM-optimized structures were submitted to MP2/6-311+G(d,p)/IEFPCM (using Gaussian 16), MP2/6-311+G(d,p)/EC-RISM.[15,16] (all using Gaussian 16) for computing the solvation free energy relative to MP2/6-311+G(d,p) in the gas phase, and to rigid-body thermodynamic integration (TI) calculations in order to provide an alternative, molecular dynamics-based approach to the solvation free energy. EC-RISM calculations were performed using the computational setup developed during the SAMPL6 blind prediction challenge[17] (140^3 grid points with 0.3 Å spacing, the PSE-2 closure,[18] a modified SPC/E water model, the GAFF force field (version 1.7)[19,20] with Lorentz−Berthelot mixing rules for Lennard-Jones (LJ) interactions, and exact periodicity-corrected solute-solvent electrostatics) on the MP2/6- 311+G(d,p) level of theory in Gaussian 09 rev. E.01.[21] For the TI calculations, 4167 SPC/E[22] water molecules were placed in a 50^3 Å cube around the molecule using packmol 1.1.2.023.[23] The NAMD 2.11[24] software was used for the simulations together with AM1-BCC charges, GAFF 1.7[19,20] parameters for LJ interactions, and a timestep of 2.0 fs. Each setup was minimized followed by 0.4 ns equilibration. The TI coupling parameter λ was scaled equidistantly in steps of 0.1 between 0 and 1 first for the LJ terms using soft-core scaling and afterwards linearly, using the same step size, for the electrostatic interactions, followed by a hysteresis estimation in the reverse order. For each λ step the system was equilibrated for 60 ps simulated for 0.4 ns. Langevin temperature and pressure control was used for setting the temperature to 298.15 K and the pressure to 1 bar. A smooth cutoff switching scheme for LJ interactions between 10 and 12 Å and a 4th order particle mesh Ewald interpolation (1.0 Å grid spacing) for the electrostatic interactions were employed. The water geometry was constrained using the SETTLE algorithm as implemented in NAMD.

Calculation of tautomer populations

The strategy used for the calculation of the tautomer populations of adenine and its derivatives followed closely Ref. 10 on the basis of the thermodynamic cycle shown in Figure 4 of Ref. 10. Reaction free energies can be derived from two “direct” routes by considering the free energy differences of species in solution only (PCM and EC-RISM), and from three “indirect” routes by computing the solvation (i.e. hydration) free energies per species explicitly (PCM, EC-RISM, TI) and supplementing the gas-phase reaction free energies taken from CCSD(T) calculations including thermal corrections on the B3LYP/6-311+G(d,p) level. Results for adenine, 7-deaza-adenine, (both including the protonated forms, see Scheme S1 for respective tautomer structures) and 7-deaza-8-aza-adenine tautomerizations are summarized in Table S1, all referenced to the Watson-Crick tautomer. All five approaches revealed similar trends which allowed us to average reaction free energies and resulting tautomer populations over all methods, thus providing a measure for uncertainty. Individual free energy components are shown in Table S2 and Table S3; structures are provided in machine-readable format in the accompanying zip file. The pKa calculations reported in Table S4 were carried out using the pKa model introduced in Ref. 17 using the so-called partition function approach for macrostate pKa’s which is described in detail in Ref. 10.

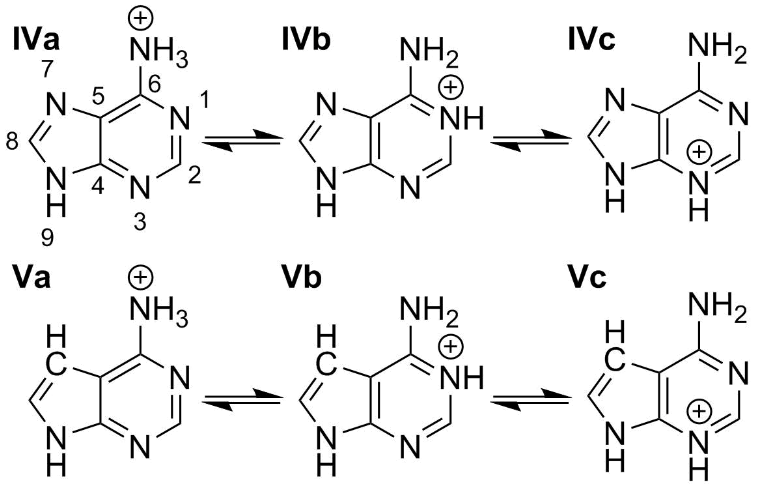

Scheme S1. Selected tautomeric forms of protonated adenine derivatives **IV-V.**

Table S1. Calculated standard reaction free energies Δ*G* (kcal mol^{-1}) and populations for selected tautomeric forms of adenine derivatives **I-V** relative to the Watson-Crick tautomers [**I-Va**] from direct MP2/6-311+G(d,p)/PCM calculations (column 2), MP2/6-311+G(d,p)/PCM hydration free energy differences and CCSD(T)/cc-pVTZ gas phase reaction free energies (column 3), direct MP2/6-311+G(d,p)/EC-RISM (column 4), MP2/6-311+G(d,p)/EC-RISM hydration free energy differences and CCSD(T)/cc-pVTZ gas phase reaction free energies (column 5), and from TI hydration free energy differences and CCSD(T)/cc-pVTZ gas phase reaction free energy (column 6); resulting averaged free energies and populations are shown in columns 7 and 8.

Δ*G* / population	PCM	PCM CCSD(T)	EC-RISM	EC-RISM CCSD(T)	TI CCSD(T)	Average Δ*G*	Population
**Ia**	0.00	0.00	0.00	0.00	0.00	0.00	>0.9999 ± $4.72\cdot10^{-8}$
**Ib**	8.79	8.73	10.02	10.16	11.41	9.82 ± 0.44	$6.31\cdot10^{-8}$ ± $4.72\cdot10^{-8}$
**Ic**	16.67	14.07	19.91	17.32	28.31	19.26 ± 2.19	$7.66\cdot10^{-15}$ ± $2.83\cdot10^{-14}$
**IIa**	0.00	0.00	0.00	0.00	0.00	0.00	>0.9999 ± $5.64\cdot10^{-7}$
**IIb**	9.12	7.72	10.32	8.97	6.47	8.51 ± 0.59	$5.69\cdot10^{-7}$ ± $5.64\cdot10^{-7}$
**IIc**	16.72	12.81	19.74	16.03	17.72	16.60 ± 1.02	$6.75\cdot10^{-13}$ ± $1.16\cdot10^{-12}$
**IIIa**	0.00	0.00	0.00	0.00	0.00	0.00	>0.9999 ± $2.82\cdot10^{-6}$
**IIIb**	7.27	6.92	8.14	7.85	6.00	7.24 ± 0.34	$4.96\cdot10^{-6}$ ± $2.82\cdot10^{-6}$
**IIIc**	14.63	12.23	16.77	14.51	14.00	14.43 ± 0.65	$2.66\cdot10^{-11}$ ± $2.91\cdot10^{-11}$
**IVa**	0.00	0.00	0.00	0.00	0.00	0.00	$4.11\cdot10^{-10}$ ± $6.97\cdot10^{-10}$
**IVb**	-10.62	-13.88	-12.06	-15.32	0.00	-12.79 ± 1.00	0.983 ± 0.061
**IVc**	-7.77	-11.69	-9.68	-13.59	0.00	-10.38 ± 1.18	0.017 ± 0.061
**Va**	0.00	0.00	0.00	0.00	0.00	0.00	$3.05\cdot10^{-10}$ ± $4.12\cdot10^{-10}$
**Vb**	-9.86	-13.99	-11.60	-15.73	0.00	-12.97 ± 0.80	0.979 ± 0.060
**Vc**	-6.89	-11.65	-9.11	-13.88	0.00	-10.68 ± 0.97	0.021 ± 0.060

Table S2. Results (in kcal mol^{-1} for energies) of EC-RISM and vacuum calculations. Electronic solute energy (E_{sol}), corrected and uncorrected excess chemical potential (μ^{ex}, $\mu^{ex,corr}$), infinite dilution partial molar volume (V_m in Å^3), vacuum energies (MP2/6-311+G(d,p) and RI-F12-CCSD(T)/cc-pVTZ results), and thermal corrections (B3LYP/6-311+G(d,p)). "b/c1" and "b/c2" denote different NH rotamers, the lines "b/c" without index show results from a discrete partition function which enter the full reaction free energy. Physically unreasonable partition function estimates are left blank.

Compound	E_{sol}	μ^{ex}	V_m	$\mu^{ex,corr}$	E_{vac}(MP2/ 6-311+G(d,p))	E_{vac}(CCSD(T)/ cc-pVTZ)	TC(B3LYP/ 6-311+G(d,p))	$E_{sol}+\mu^{ex,corr}$
**Ia**	-292513.74	-19.02	119.76	-31.26	-292526.64	-292753.30	49.14	-292545.00
**Ib,1**	-292494.85	-29.34	116.80	-41.28	-292514.55	-292742.49	50.61	-292536.12
**Ib,2**	-292480.88	-42.27	116.29	-54.15	-292507.82	-292736.19	50.11	-292535.04
**Ib**	-	-	-	-	-	-	-	-292536.21
**Ic,1**	-292457.12	-59.25	113.51	-70.84	-292494.61	-292723.57	48.83	-292527.96
**Ic,2**	-292457.45	-58.76	114.11	-70.42	-292494.58	-292723.48	48.79	-292527.87
**Ic**	-	-	-	-	-	-	-	-292528.33
**IIa**	-282451.86	-12.95	134.39	-26.68	-282462.85	-282686.96	57.28	-282478.54
**IIb,1**	-282436.12	-19.77	131.41	-33.20	-282451.61	-282677.38	57.64	-282469.31
**IIb,2**	-282429.33	-25.71	130.74	-39.06	-282448.35	-282674.19	57.30	-282468.40
**IIb**	-	-	-	-	-	-	-	-282469.43
**IIc,1**	-282399.30	-49.03	128.39	-62.15	-282432.06	-282658.89	55.90	-282461.46
**IIc,2**	-282408.46	-39.76	128.75	-52.92	-282435.86	-282662.36	56.10	-282461.37
**IIc**	-	-	-	-	-	-	-	-282461.83
**IIIa**	-292496.35	-17.70	121.36	-30.10	-292509.53	-292737.63	49.84	-292526.45
**IIIb,1**	-292483.99	-22.88	119.04	-35.04	-292500.53	-292729.61	50.59	-292519.04
**IIIb,2**	-292478.01	-28.13	118.57	-40.25	-292497.36	-292726.53	50.24	-292518.25
**IIIb**	-	-	-	-	-	-	-	-292519.18
**IIIc,1**	-292452.28	-47.09	115.84	-58.92	-292482.33	-292712.37	49.13	-292511.20
**IIIc,2**	-292461.49	-38.14	116.78	-50.08	-292486.24	-292715.95	49.26	-292511.57
**IIIc**	-	-	-	-	-	-	-	-292511.82
**Iva**	-292728.38	-57.90	137.36	-87.24	-292739.20	-293066.73	58.41	-292815.63
**IVb**	-292746.92	-48.98	147.09	-79.32	-292756.39	-293087.43	58.67	-292826.25
**IVc**	-292743.41	-49.64	147.21	-79.99	-292754.04	-293085.74	58.67	-292823.40
**Va**	-282666.63	-54.83	153.32	-85.80	-282677.67	-283000.42	65.77	-282752.44
**Vb**	-282689.15	-41.18	163.06	-73.15	-282696.41	-283023.07	65.56	-282762.30
**Vc**	-282688.27	-39.04	163.52	-71.06	-282694.49	-283021.96	65.73	-282759.32

Table S3. Results (in kcal mol^{-1}) of PCM (MP2/6-311+G(d,p)) and TI calculations. Additionally, the solvation free energies $\Delta_{solv}G$ calculated using MP2 vacuum energies (with the frozen core approximation for PCM, without for EC-RISM), the sum of solvation free energies, CCSD(T) and TC, and the free energies relative to the Watson-Crick tautomer [**I-Va**] are given. "b/c1" and "b/c2" denote different NH rotamers, the lines "b/c" without index show results from a discrete partition function which enter the full reaction free ene. Physically unreasonable partition function estimates are left blank.

Cmpd.	PCM	$\Delta_{solv}G$ PCM	$\Delta_{solv}G$ EC-RISM	$\Delta_{solv}G$ TI	PCM+ CCSD(T)	EC-RISM+ CCSD(T)	TI+ CCSD(T)	$\Delta\Delta_{solv}G$ PCM	$\Delta\Delta_{solv}G$ PCM+ CCSD(T)	$\Delta\Delta_{solv}G$ EC-RISM	$\Delta\Delta_{solv}G$ EC-RISM+ CCSD(T)	$\Delta\Delta_{solv}G$ TI+ CCSD(T)
**Ia**	-292535.79	-9.16	-18.36	-15.59±0.21	-292713.31	-292722.52	-292719.75	0.00	0.00	0.00	0.00	0.00
**Ib,1**	-292525.76	-11.21	-21.57	-14.25±0.23	-292703.09	-292713.45	-292706.13	-	-	-	-	-
**Ib,2**	-292523.54	-15.73	-27.22	-22.26±0.22	-292701.80	-292713.29	-292708.33	-	-	-	-	-
**Ib**	-292525.78	-	-	-	-292703.15	-292713.79	-292708.34	10.02	10.16	8.79	8.73	11.41
**Ic,1**	-292515.47	-20.86	-33.35	-15.72±0.21	-292695.60	-292708.09	-292690.46	-	-	-	-	-
**Ic,2**	-292515.47	-20.88	-33.29	-16.62±0.23	-292695.58	-292707.98	-292691.32	-	-	-	-	-
**Ic**	-292515.88	-	-	-	-292696.00	-292708.45	-292691.44	19.91	17.32	16.67	14.07	28.31
**IIa**	-282471.27	-8.42	-15.69	-13.08±0.22	-282638.10	-282645.37	-282642.76	0.00	0.00	0.00	0.00	0.00
**IIb,1**	-282460.91	-9.30	-17.71	-16.49±0.20	-282629.04	-282637.45	-282636.24	-	-	-	-	-
**IIb,2**	-282459.42	-11.07	-20.05	-17.88±0.23	-282627.96	-282636.94	-282634.77	-	-	-	-	-
**IIb**	-282460.95	-	-	-	-282629.13	-282637.66	-282636.28	10.32	8.97	9.12	7.72	6.47
**IIc,1**	-282450.49	-18.44	-29.40	-19.10±0.22	-282621.43	-282632.39	-282622.09	-	-	-	-	-
**IIc,2**	-282451.42	-15.56	-25.51	-18.77±0.24	-282621.82	-282631.77	-282625.03	-	-	-	-	-
**IIc**	-282451.53	-	-	-	-282622.07	-282632.57	-282625.04	19.74	16.03	16.72	12.81	17.72
**IIIa**	-292518.12	-8.59	-16.92	-14.45±0.23	-292696.38	-292704.71	-292702.24	0.00	0.00	0.00	0.00	0.00
**IIIb,1**	-292509.91	-9.38	-18.51	-17.20±0.24	-292688.40	-292697.53	-292696.21	-	-	-	-	-
**IIIb,2**	-292508.67	-11.31	-20.89	-18.19±0.22	-292687.60	-292697.19	-292694.49	-	-	-	-	-
**IIIb**	-292509.97	-	-	-	-292688.53	-292697.79	-292696.24	8.14	7.85	7.27	6.92	6.00
**IIIc,1**	-292500.07	-17.74	-28.87	-21.32±0.23	-292680.98	-292692.11	-292684.56	-	-	-	-	-
**IIIc,2**	-292501.28	-15.04	-25.33	-21.56±0.21	-292681.72	-292692.01	-292688.24	-	-	-	-	-
**IIIc**	-292501.35	-	-	-	-292681.87	-292692.48	-292688.24	16.77	14.51	14.63	12.23	14.00
**IVa**	-292799.02	-59.82	-76.43	-	-293068.14	-293084.74	-	0.00	0.00	0.00	0.00	-
**IVb**	-292811.08	-54.69	-69.86	-	-293083.45	-293098.62	-	-12.06	-15.32	-10.62	-13.88	-
**IVc**	-292808.70	-54.65	-69.35	-	-293081.73	-293096.43	-	-9.68	-13.59	-7.77	-11.69	-
**Va**	-	-59.53	-74.76	-	-282994.18	-283009.42	-	0.00	0.00	0.00	0.00	-

	282737.20											
**Vb**	- 282748.81	-52.39	-65.89	-	-283009.91	-283023.40	-	-11.60	-15.73	-9.86	-13.99	-
**Vc**	- 282746.31	-51.83	-64.84	-	-283008.06	-283021.07	-	-9.11	-13.88	-6.89	-11.65	-

Table S4. Results of MP2/6-311+G(d,p)/EC-RISM microstate pK_a calculations. Microstate transitions from the unprotonated species [**I/IIa-c**] to the corresponding protonated species [**IV/Va-c**] and from the macrostates [**I/II**] to the protonated microstates [**IV/Va-c**] are given. The corresponding macrostate pK_a's for the species [**I/IV**] and [**II/V**] accounting for all tautomers in the neutral and the charged states are 1.84 and 3.20, indicating the dominant role of position 1 for protonation under acidic conditions.

Cmpd.	**IVa**	**IVb**	**IVc**
**I**	-3.93	1.83	0.29
**Ia**	-3.93	1.83	0.29
**Ib**	0.84	6.60	5.06
**Ic**	5.12	10.88	9.33
	**Va**	**Vb**	**Vc**
**II**	-2.15	3.20	1.58
**IIa**	-2.15	3.20	1.58
**IIb**	2.79	8.14	6.53
**IIc**	6.92	12.26	10.65

Synthesis of chemically stabilized nucleoside phosphoramidites A and C

The syntheses followed published procedures.[25]

Synthesis of N^6-Benzoyl-2'-deoxy-5'-O-DMT-7-deaza-2'-deoxyadenosine 3'-CE phosphoramidite A

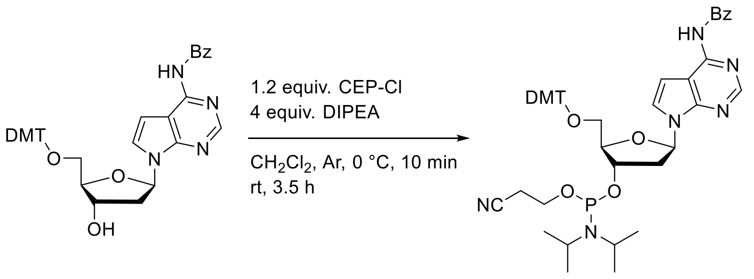

A

Prior to use, N^6-Benzoyl-2'-deoxy-5'-O-DMT-7-deaza-2'-deoxyadenosine was dried in high *vacuo* overnight. To the stirred solution of N^6-Benzoyl-2'-deoxy-5'-O-DMT-7-deaza-2'-deoxyadenosine (500 mg, 0.76 mmol) in dry dichloromethane (5 mL) and dry DIPEA (523 µL, 3.05 mmol, 4 equiv.) was added 2-cyanoethyl diisopropylphosphoramidochloridite (215 µL, 0.91 mmol, 1.2 equiv.) at 0 °C under an argon atmosphere. The cooling bath was removed after 10 minutes and the solution was stirred at ambient temperature for 3.5 hours. The solution was filtered and diluted with 5 mL dichloromethane. The organic phase was washed with a saturated aqueous solution of $NaHCO_3$ (2x 10 mL), brine (10 mL) then dried over anhydrous $MgSO_4$, filtered and concentrated in vacuo. Product **A** was obtained as colorless foam and as a diastereoisomeric mixture with 92% (602 mg, 0.70 mmol) yield. It was used without further purification for solid-phase oligonucleotide synthesis.

^{1}H NMR (500 MHz, CD_2Cl_2) δ = 8.75 (s, 2H), 8.51 (s, 2H), 7.99 (d, *J* = 7.4 Hz, 3H), 7.63 (t, *J* = 7.4 Hz, 2H), 7.55 (t, *J* = 7.6 Hz, 3H), 7.47 – 7.42 (m, 3H), 7.39 – 7.19 (m, 14H), 7.02 (d, *J* = 2.6 Hz, 2H), 6.84 – 6.77 (m, 8H), 4.77 – 4.70 (m, 2H), 4.28 – 4.21 (m, 2H), 4.21 – 4.05 (m, 2H), 3.92 – 3.83 (m, 2H), 3.79 – 3.57 (m, 16H), 3.54 – 3.44 (m, 2H), 3.41 – 3.26 (m, 4H), 2.76 – 2.51 (m, 8H), 2.49 (t, *J* = 6.4 Hz, 2H), 1.28 – 1.11 ppm (m, 28H); ^{13}C NMR (126 MHz, CD_2Cl_2) δ = 159.22, 159.21, 145.45, 136.39, 136.36, 136.34, 133.16, 130.67, 130.63, 129.41, 128.75, 128.69, 128.38, 128.37, 128.22, 128.21, 127.38, 127.35, 124.08, 124.06, 113.64, 86.90, 84.08, 84.04, 74.72, 74.58, 74.19, 74.06, 64.34, 64.21, 59.09, 59.00, 58.94, 58.85, 55.78, 55.76, 45.86, 45.81, 43.89, 43.87, 43.79, 43.77, 40.18, 40.13, 24.98, 24.94, 24.93, 24.88, 23.24, 21.00, 20.94, 20.85, 20.80 ppm; ^{31}P NMR (162 MHz, CD_2Cl_2) δ = 148.57, 148.53 ppm,.

Synthesis of *N*[6]-DMF-2'-deoxy-5'-O-DMT-7-deaza-8-aza-2'-deoxyadenosine B

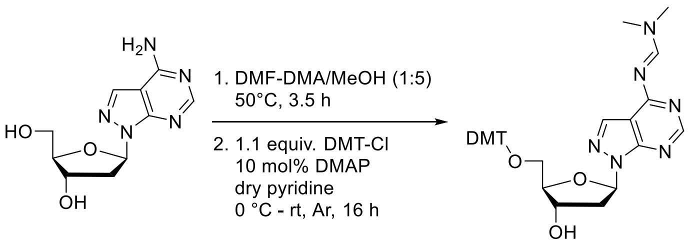

B

Prior to use, 7-deaza-8-aza-2'-deoxyadenosine was dried in high *vacuo* overnight. The solution of 7-deaza-8-aza-2'-deoxyadenosine (500 mg, 2.00 mmol) in dry methanol (10 mL) and DMF-DMA (2 mL) was stirred at 50 °C for 3.5 hours under an argon atmosphere. The solvents were removed under reduced pressure. The orange residue was coevaporated two times with each 5 mL dry methanol and 5 mL diethyl ether, dried in high *vacuo* for 16 h and directly used in the next step without further purification.

To the solution of N^6-DMF-2'-deoxy-7-deaza-8-azaadenosine (612 mg, 2.00 mmol) in dry pyridine (6 mL) were added DMAP (22.6 mg, 0.20 mmol, 10 mol%, in 0.4 mL dry pyridine) and 4,4´-dimethoxytriphenylmethyl chloride (752.1 mg, 2.20 mmol, 1.1 equiv., in 3.6 mL dry pyridine) at 0 °C under an argon atmosphere. The solution was allowed to warm up to 25 °C and stirred overnight. The solvent was removed under reduced pressure and the residue was solved in 100 mL dichloromethane. The organic layer was washed with ice-cold brine (3x 50 mL) and ice-cold water (1x 50 mL), dried over $MgSO_4$ and the solvent was removed under reduced pressure. The product was purified by silica gel column chromatography using methanol/ dichloromethane as eluent. Product **B** was obtained as colorless solid with 52% (637 mg, 1.05 mmol) yield.

^{1}H NMR (500 MHz, CD_2Cl_2) δ = 8.91 (s, 1H), 8.47 (s, 1H), 8.04 – 8.01 (m, 1H), 7.40 – 7.36 (m, 2H), 7.29 – 7.24 (m, 4H), 7.23 – 7.14 (m, 3H), 6.77 – 6.70 (m, 5H), 4.80 (dd, J = 10.9, 5.6 Hz, 1H), 4.03 (q, J = 5.5 Hz, 1H), 3.75 (s, 3H), 3.75 (s, 3H), 3.25 – 3.16 (m, 8H), 3.08 – 3.01 (m, 1H), 2.43 – 2.36 (m, 1H), 2.24 ppm (s, 1H); ^{13}C NMR (126 MHz, CD_2Cl_2) δ = 163.12, 159.04, 159.01, 158.15, 156.05, 155.66, 145.66, 136.58, 136.52, 134.15, 130.56, 130.48, 128.62, 128.23, 127.15, 113.47, 109.29, 86.58, 85.95, 84.50, 73.45, 66.22, 65.06, 55.70, 41.80, 38.89, 35.42 ppm.

Synthesis of N^6-DMF-2'-deoxy-5'-O-DMT-2'-7-deaza-8-aza-2'-deoxyadenosine 3'-CE phosphoramidite C

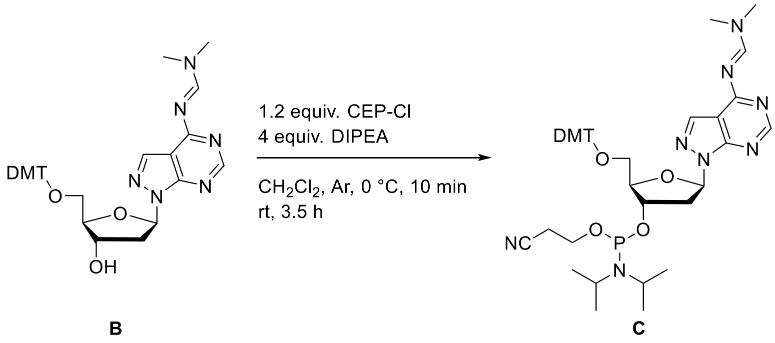

Prior to use, N^6-DMF-2'-deoxy-5'-O-DMT-2'-7-deaza-8-aza-2'-deoxyadenosine was dried in high *vacuo* overnight. To the stirred solution of *N*^{6}-DMF-2'-deoxy-5'-O-DMT-2'-7-deaza-8-aza-2'-deoxyadenosine (625 mg, 1.03 mmol) in dry dichloromethane (9 mL) and DIPEA (706 µL, 4.11 mmol, 4 equiv.) was added was added 2-cyanoethyl diisopropylphosphoramidochloridite (278 µL, 1.23 mmol, 1.2 equiv.) at 0 °C under a argon atmosphere. The cooling bath was removed after 10 minutes and the solution was stirred at ambient temperature for 3.5 hours. The solution was filtered and diluted with 15 mL dichloromethane. The organic phase was washed with a saturated aqueous solution of $NaHCO_3$ (2x 30 mL), brine (30 mL) then dried over anhydrous $MgSO_4$, filtered and concentrated in *vacuo*. Product **C** was obtained as colorless foam and as a diastereoisomeric mixture with 98% (814 mg, 1.01 mmol) yield. It was used without further purification for solid-phase oligonucleotide synthesis. ^{1}H NMR (600 MHz, CD^2Cl_2) δ = 8.92 (s, 2H), 8.49 (d, J = 1.4 Hz, 2H), 8.06 – 8.00 (m, 2H), 7.41 – 7.35 (m, 4H), 7.31 – 7.22 (m, 8H), 7.22 – 7.12 (m, 6H), 6.78 – 6.65 (m, 10H), 4.92 – 4.82 (m, 1H), 4.22 – 4.16 (m, 2H), 3.88 – 3.53 (m, 20H), 3.28 – 3.09 (m, 17H), 2.77 – 2.42 (m, 5H), 1.27 – 1.07 ppm (m, 30H); ^{13}C NMR (151 MHz, CD_2Cl_2) δ = 163.16, 159.03, 159.02, 159.00, 158.98, 158.14, 156.08, 155.79, 155.78, 145.75, 145.72, 136.74, 136.70, 136.62, 136.60, 134.14, 130.65, 130.62, 130.55, 130.52, 128.77, 128.71, 128.19, 128.17, 127.12, 127.08, 118.31, 118.23, 113.44, 109.42, 109.42, 86.48, 86.46, 85.76, 85.73, 85.56, 85.52, 84.92, 74.82, 74.70, 74.36, 74.25, 71.08, 64.82, 64.70, 59.14, 59.05, 59.02, 58.92, 58.63, 55.71, 55.69, 46.77, 46.74, 45.83, 45.79, 43.84, 43.81, 43.76, 43.73, 41.80, 38.19, 38.17, 38.06, 38.03, 35.43, 24.96, 24.93, 24.91, 24.89, 24.86, 24.84, 24.82, 23.22, 23.21, 22.84, 22.79, 20.95, 20.90, 20.79, 20.74 ppm.

NMR spectra

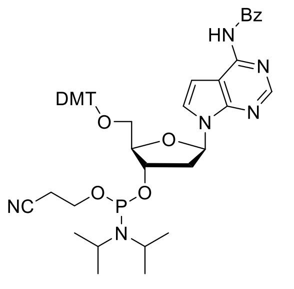

A

^{1}H NMR

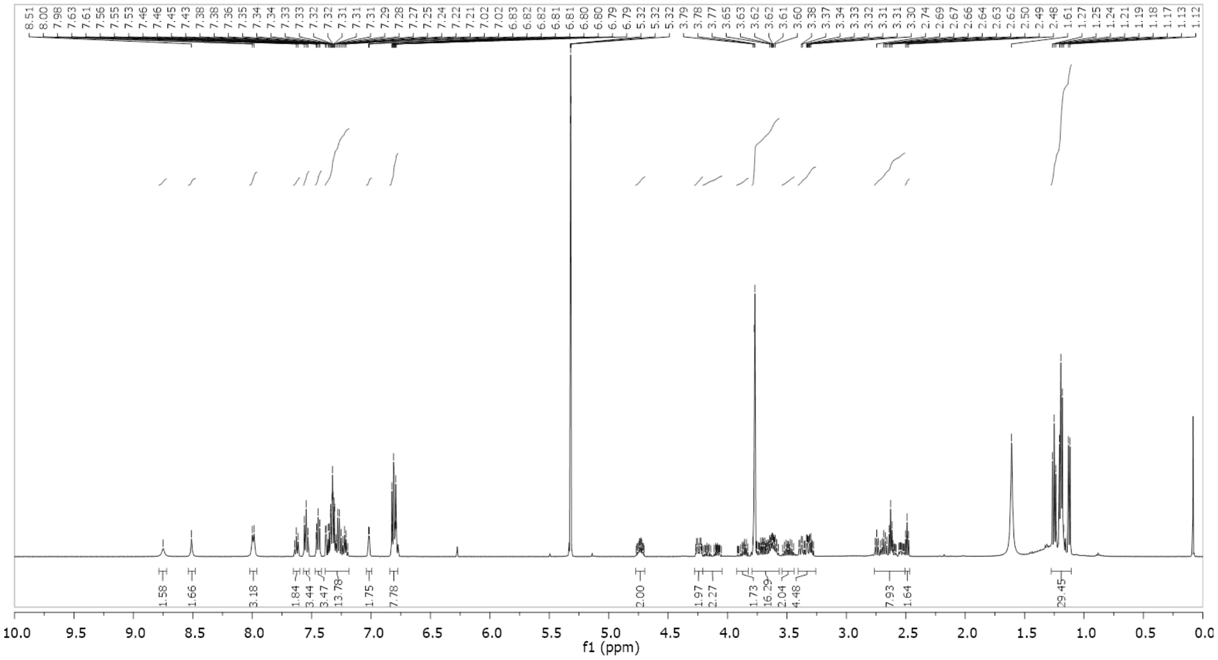

^{13}C NMR

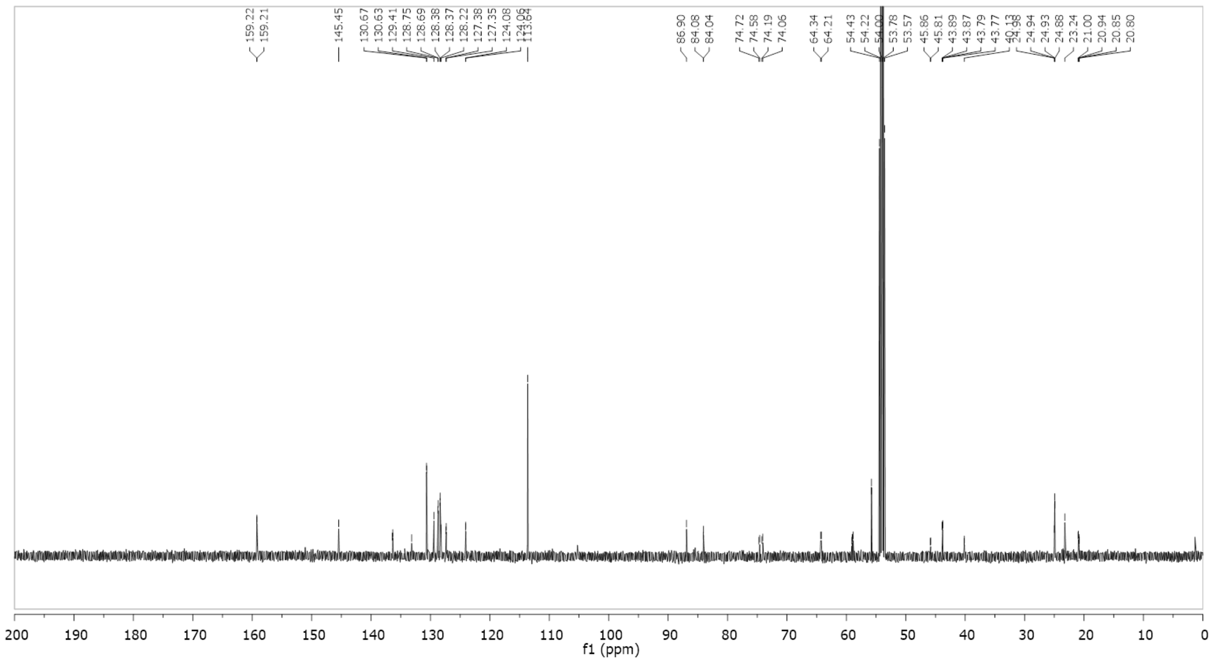

^{31}P NMR

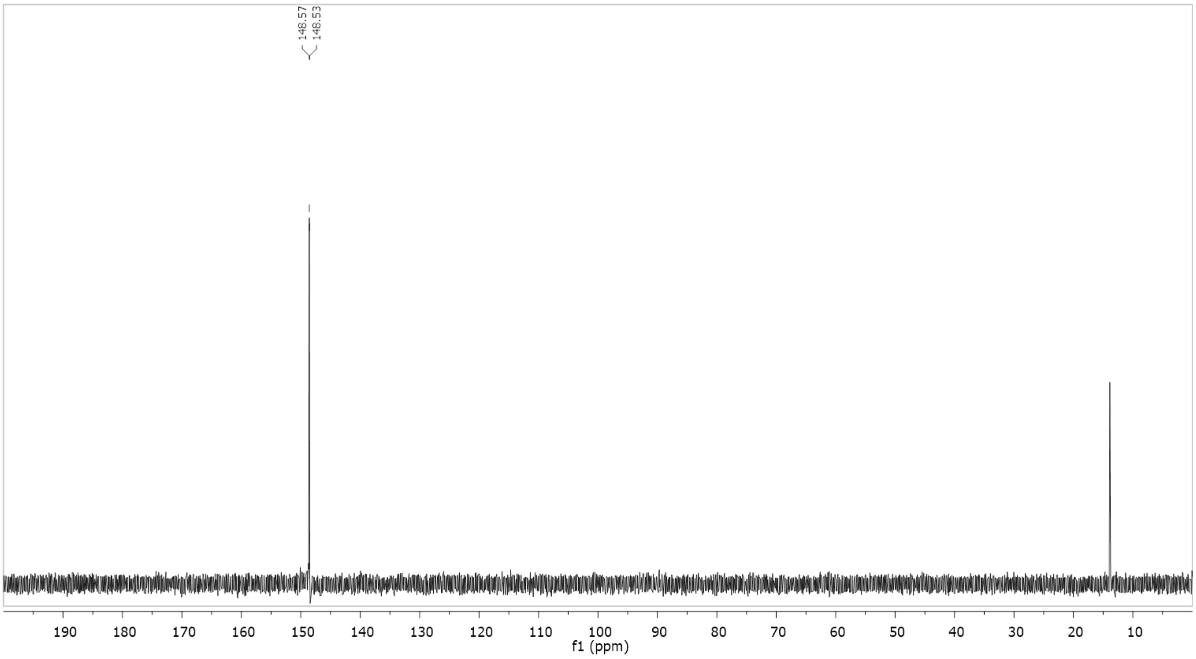

148.57
148.53
190 180 170 160 150 140 130 120 110 100 90 80 70 60 50 40 30 20 10
f1 (ppm)

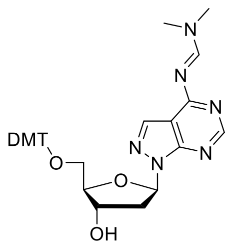

B

^{1}H NMR

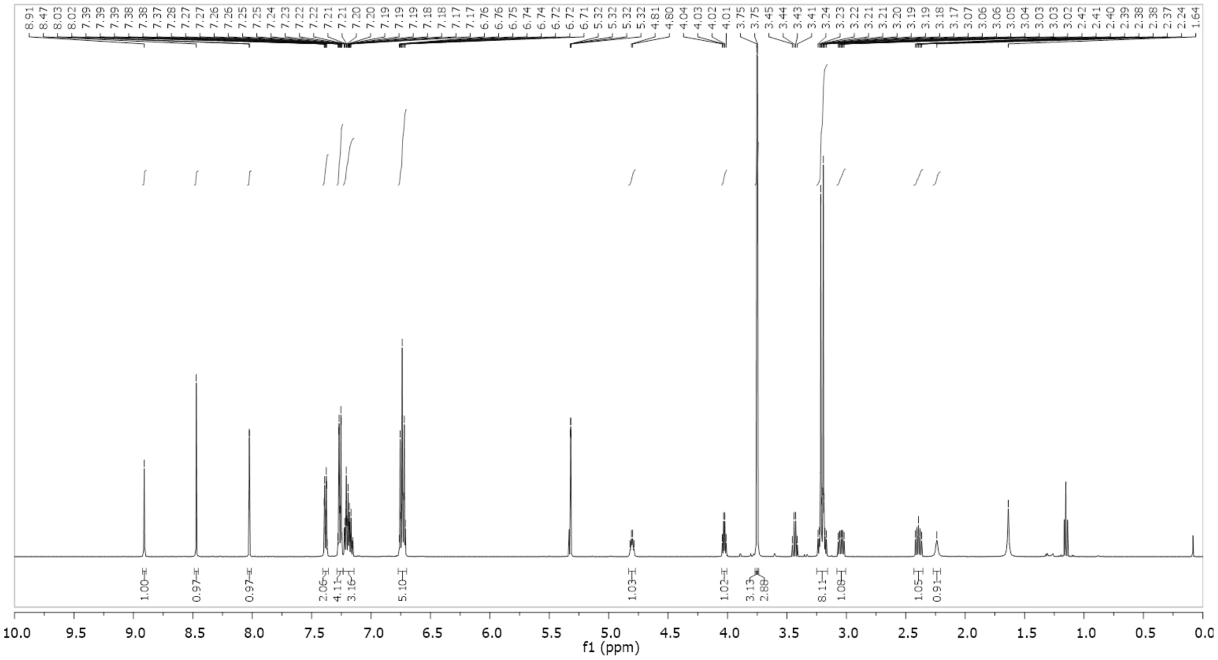

^{13}C NMR

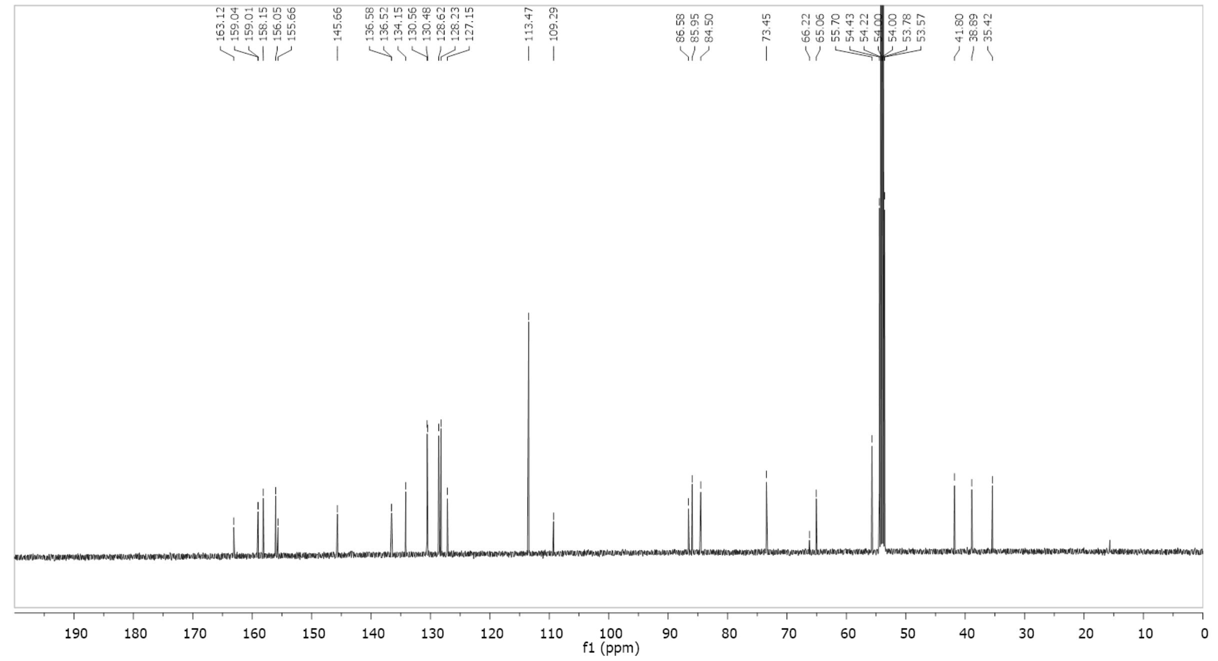

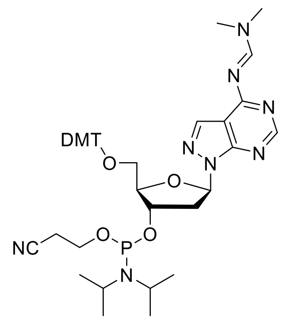

C

1H NMR

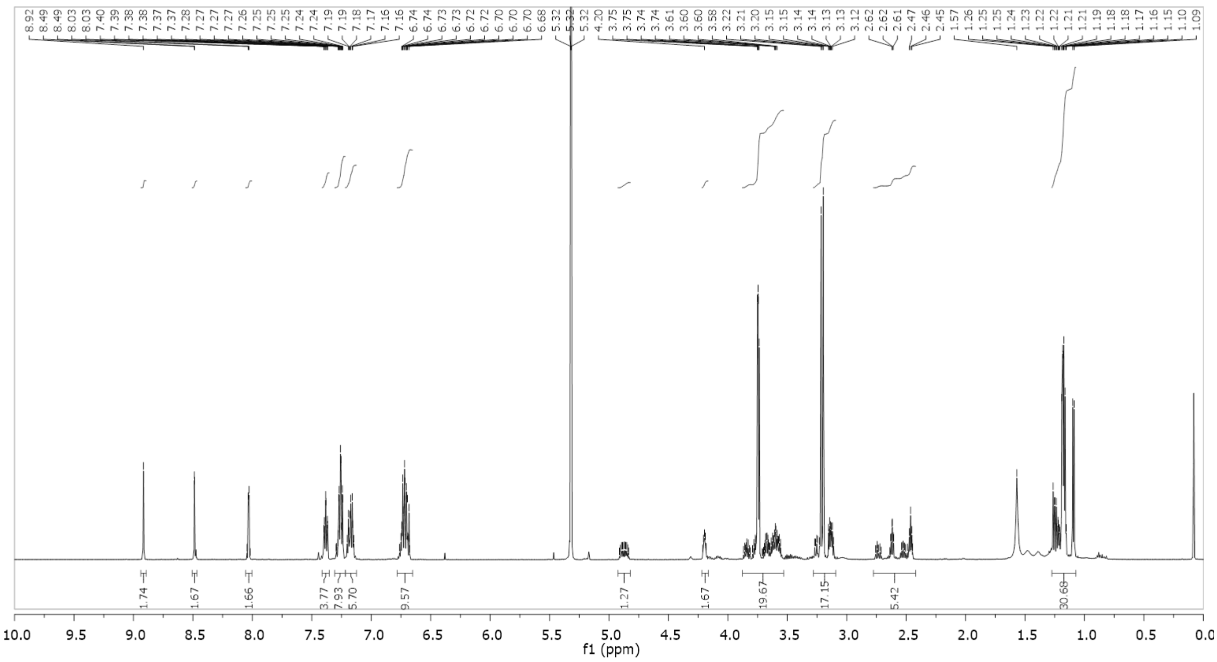

13C NMR

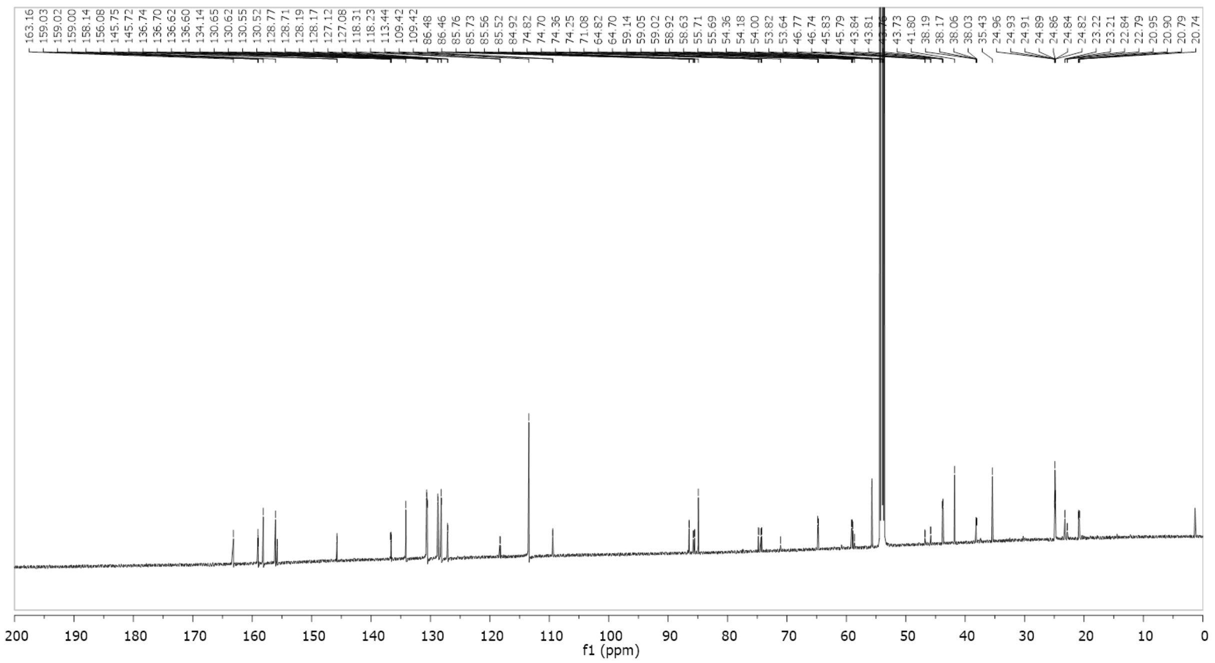

Chemical stability screening of DNA barcodes

Representative Procedures

Treatment of solid support-bound stabilized oligonucleotide with aqueous acids (RP-01)

DMT-cleavage: The DMT-protecting group of DNA strand bound to 1000 Å controlled pore glass (CPG) solid support (1 µmol, ~40 mg of 10mer 7De-dATC- and 7De8a-dATC-sequence **5** and **6**) was cleaved by addition of 200 µL 3% trichloroacetic acid in CH_2Cl_2 for 1 min. Orange coloring of the solution indicated successful removal of protecting group. The deprotection was repeated 3-5 times until no further coloring of the solution was observed. CPG-bound deprotected DNA was washed three times with each 200 µL of 1% TEA in ACN, DMF, MeOH, ACN and CH_2Cl_2 and dried *in vacuo*.

Investigation of stability: 20 nmol of CPG-bound stabilized oligonucleotide (0.75 mg) was treated with 50 µL aqueous acid. The suspension was shaken at ambient temperature for 22 h. Afterwards solution was removed under vacuum filtration, CPG was washed with excess of 1% TEA and three times with each 200 µL of 0.1 M $MgCl_2$ solution, water, DMF, MeOH, ACN and CH_2Cl_2 and dried *in vacuo*.

Cleavage and Analysis: DNA was deprotected and cleaved from CPG by shaking in 500 µL of an AMA solution (AMA = aqueous ammonia (30%)/ aqueous methylamine (40%), 1:1, vol/vol) for 4 h at room temperature. Afterwards 20 µL of 1 M Tris buffer (pH = 7.5) were added, the mixture was dried under reduced pressure (SpeedVac) and dissolved in 200 µL of distilled water. The product was analyzed by analytical RP-HPLC and MALDI-TOF-MS.

Treatment of solid support-bound stabilized oligonucleotides with metal salts or organic reagents (RP-02)

DMT-cleavage: DMT-protecting group of DNA strand bound to 1000 Å controlled pore glass (CPG) solid support (1 µmol, ~40 mg of 10mer 7-De-dATC- and 7-De8a-dATC-sequence) was cleaved by addition of 200 µL 3% trichloroacetic acid in CH_2Cl_2 for 1 min. Orange coloring of the solution indicated successful removal of protecting group. The deprotection was repeated 3-5 times until no further coloring of the solution was observed. CPG-bound deprotected DNA was washed three times with each 200 µL of 1% TEA in ACN, DMF, MeOH, ACN and CH_2Cl_2 and dried *in vacuo*.

Investigation of stability: 20 nmol of CPG-bound stabilized oligonucleotide (0.75 mg) were treated with 200 equiv. of metal salt/organic reagent (4 µmol) solved in 50 µL dry solvent. The suspension was shaken at ambient temperature for 22 h. Afterwards the solvent was removed under vacuum filtration, CPG was washed three times with each 200 µL of 0.1 M

EDTA solution, 0.1 M $MgCl_2$ solution, water, DMF, MeOH, ACN and CH_2Cl_2 and dried *in vacuo*.

Cleavage and Analysis: DNA was deprotected and cleaved from CPG by shaking in 500 µL of an AMA solution (AMA = aqueous ammonia (30%)/ aqueous methylamine (40%), 1:1, vol/vol) for 4 h at room temperature. Afterwards 20 µL of 1 M Tris buffer (pH = 7.5) were added, the mixture was dried under reduced pressure (SpeedVac) and DNA was dissolved in 200 µL distilled water. The product was analyzed by analytical RP-HPLC and MALDI-TOF-MS.

Table S5 – Stabilitiy of chemically modified oligonucleotides **5** and **6** in the presence of different metal salts, organocatalysts and acids [a]

Entry	Reagent	Solvent	7De-dATC 5	7De8a-dATC 6
1	10% TFA	H_2O	[green: 0-20%]	[green: 0-20%]
2	3.7% HCl	H_2O	[green: 0-20%]	[green: 0-20%]
3	$Bi(OTf)_3$	MeOH	[green: 0-20%]	[green: 0-20%]
4	$Ce(NH_4)_2(NO_3)_6$	MeOH	[green: 0-20%]	[green: 0-20%]
5	$Co(acac)_3$	ACN	[green: 0-20%]	[green: 0-20%]
6	$Cu(MeCN)_4PF_6$	ACN	[green: 0-20%]	[green: 0-20%]
7	$FeCl_2 \cdot 4\,H_2O$	ACN	[green: 0-20%]	[green: 0-20%]
8[b]	La(O*i*-Pr)$_3$	THF	[green: 0-20%]	[green: 0-20%]
9	LiBr	ACN	[green: 0-20%]	[green: 0-20%]
10	$Ni(acac)_2$	ACN	[green: 0-20%]	[green: 0-20%]
11	$Pd(OAc)_2$	ACN	[red: > 61%]	[red: > 61%]
12	$RuCl_3$	ACN	[light green: 21-40%]	[light green: 21-40%]
13	[Ru(*p*-cymene)Cl$_2$]$_2$	CH_2Cl_2	[light green: 21-40%]	[green: 0-20%]
14	Grubbs 1st Gen.	CH_2Cl_2	[green: 0-20%]	[green: 0-20%]
15	$Sc(OTf)_3$	ACN	[green: 0-20%]	[green: 0-20%]
16[c]	$Sc(OTf)_3$	ACN	[light green: 21-40%]	[light green: 21-40%]
17	SeO_2	MeOH	[green: 0-20%]	[green: 0-20%]
18	$VO(acac)_2$	MeOH	[green: 0-20%]	[green: 0-20%]
19	$ZnCl_2$	ACN	[green: 0-20%]	[green: 0-20%]
20	DDQ	EtOH	[green: 0-20%]	[green: 0-20%]
21	PIDA	ACN	[green: 0-20%]	[green: 0-20%]
22	TEMPO	ACN	[green: 0-20%]	[green: 0-20%]

[a] For each: 20 nmol DNA, aqueous acids or 200 equiv. transition metal salt or 200 equiv. organic reagent, 50 µL solvent, rt, 22 h. [b] Poor solubility, added as suspension. [c] Experiment was performed at 40 °C. ACN = acetonitrile, MeOH = methanol.

5 = 5'-TT7De-dA CT7De-dA CCT 7De-dA-3'-CPG

6 = 5'-TT7De8a-dA CT7De8a-dA CCT 7De8a-dA-3'-CPG

[green]	[light green]	[orange]	[red]	
0-20%	21-40%	41-60%	> 61%	degree of DNA degradation

HPLC traces and MALDI-MS spectra of metal ion screens

CPG-oligonucleotide + 10% TFA

According to the representative procedure RP-01 solid support-coupled oligonucleotide (20 nmol) was treated with 10% TFA.

CPG-oligonucleotide	Analytical data
10mer 7De-dATC **5**<br>Analytical RP-HPLC-trace	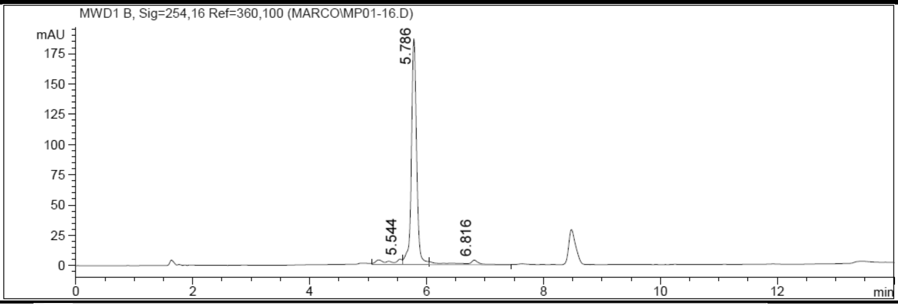
MALDI-MS spectrum	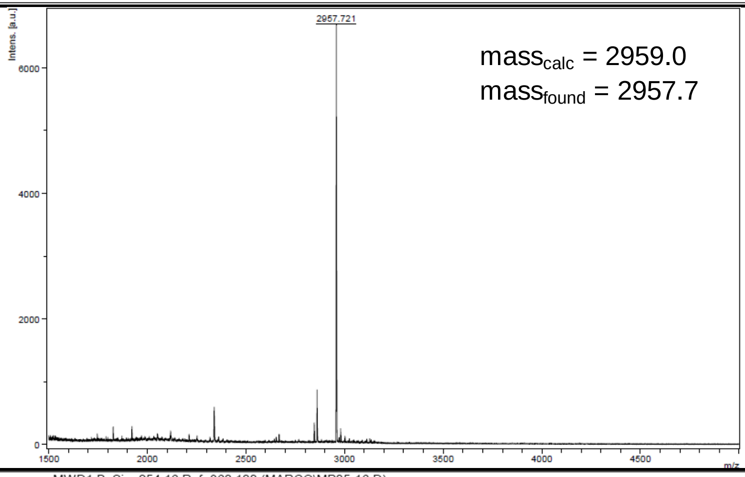
10mer 7De8a-dATC **6**<br>Analytical RP-HPLC-trace	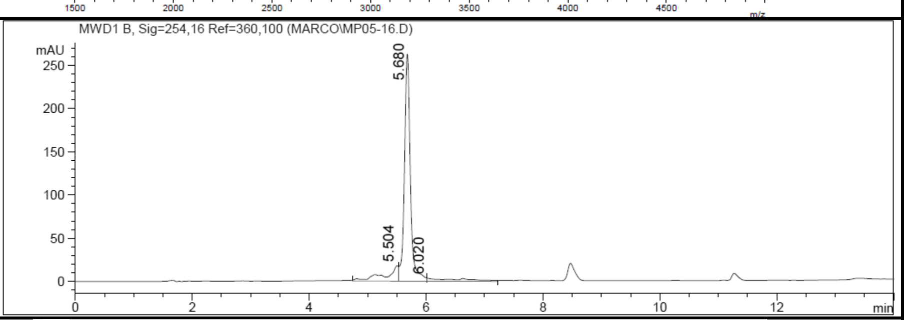
MALDI-MS spectrum	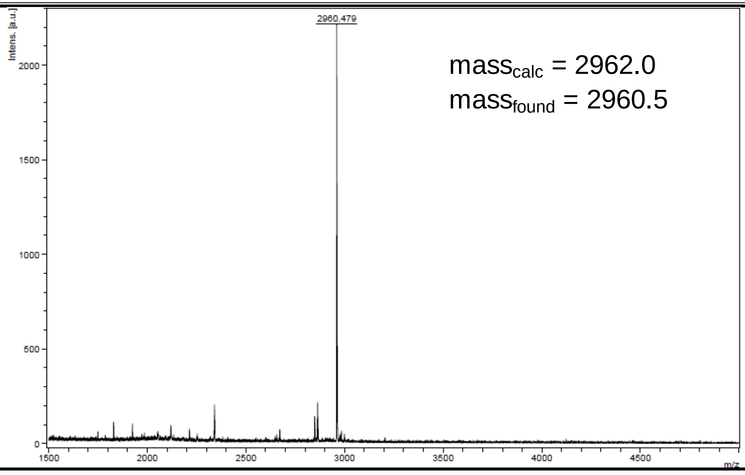

CPG-oligonucleotide + 3.7% HCl

According to the representative procedure RP-01 solid support-coupled oligonucleotide (20 nmol) was treated with 3.7% HCl.

CPG-oligonucleotide	Analytical data
10mer 7De-dATC **5**<br><br>Analytical RP-HPLC-trace	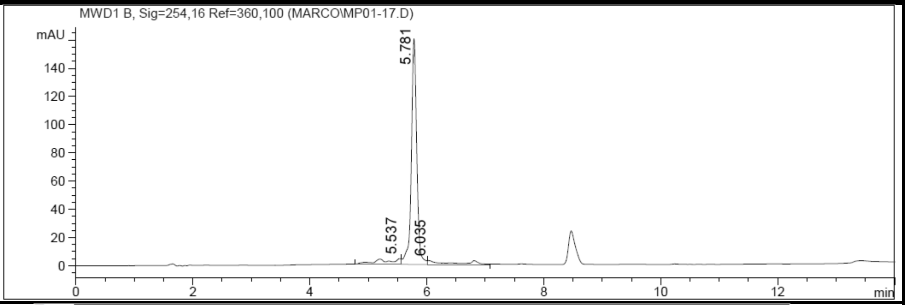
MALDI-MS spectrum	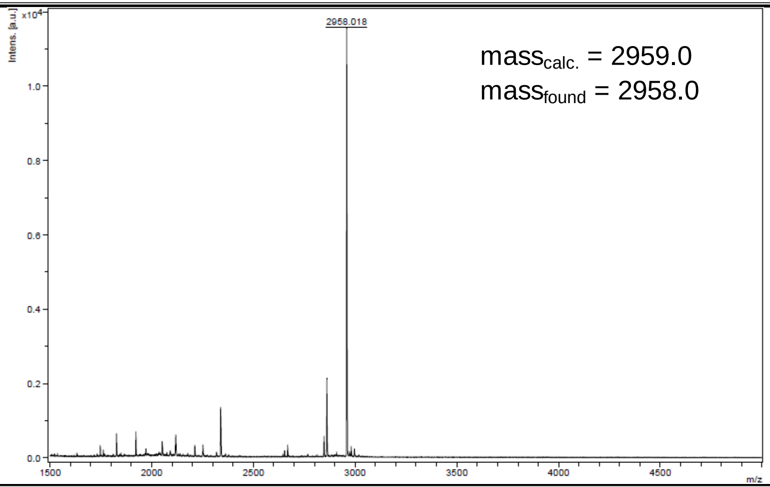
10mer 7De8a-dATC **6**<br><br>Analytical RP-HPLC-trace	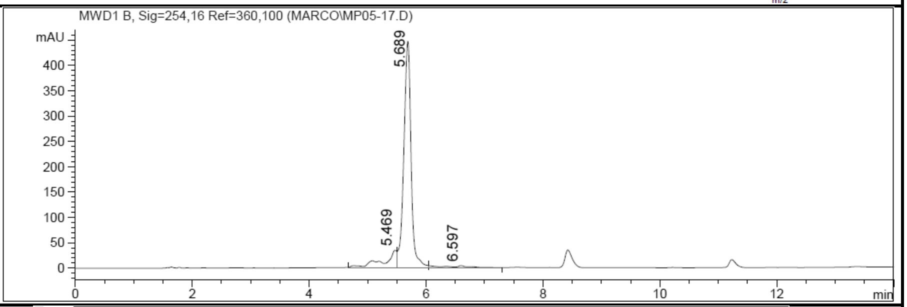
MALDI-MS spectrum	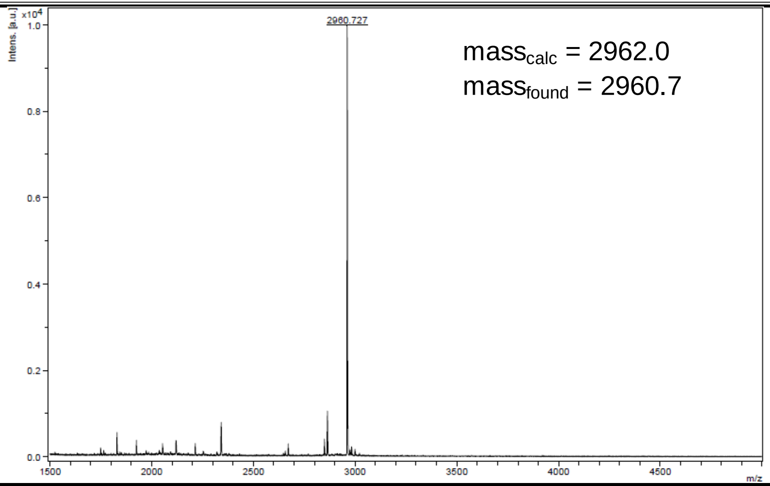

CPG-oligonucleotide + Bi(OTf)$_3$

According to the representative procedure RP-02 solid support-coupled oligonucleotide (20 nmol) was treated with Bi(OTf)$_3$.

CPG-oligonucleotide	Analytical data
10mer 7De-dATC **5**<br>Analytical RP-HPLC-trace	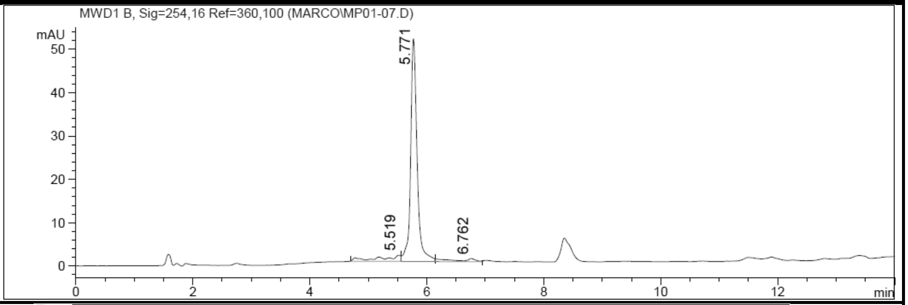
MALDI-MS spectrum	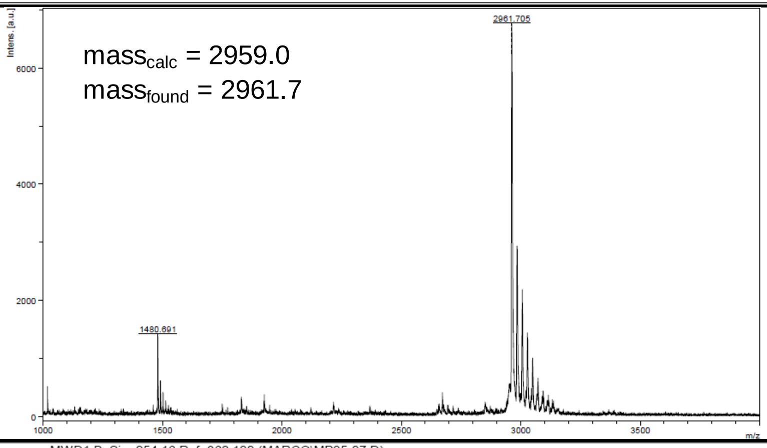
10mer 7De8a-dATC **6**<br>Analytical RP-HPLC-trace	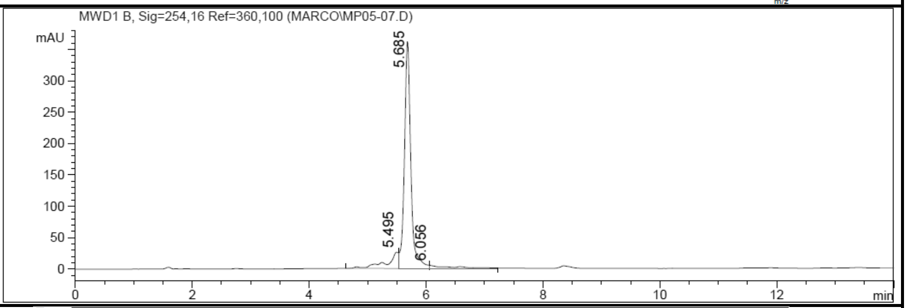
MALDI-MS spectrum	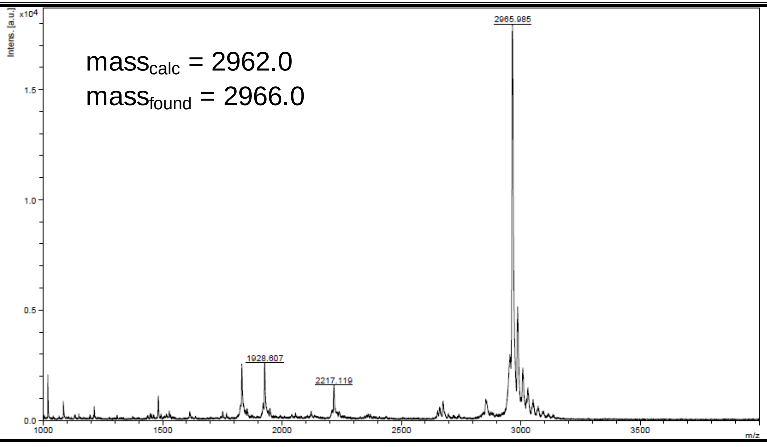

CPG-oligonucleotide + $Ce(NH_4)_2(NO_3)_6$

According to the representative procedure RP-02 solid support-coupled oligonucleotide (20 nmol) was treated with $Ce(NH_4)_2(NO_3)_6$.

CPG-oligonucleotide	Analytical data
10mer 7De-dATC **5**<br>Analytical RP-HPLC-trace	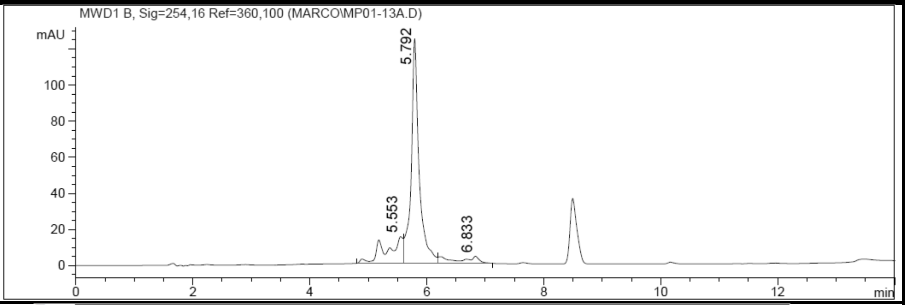
MALDI-MS spectrum	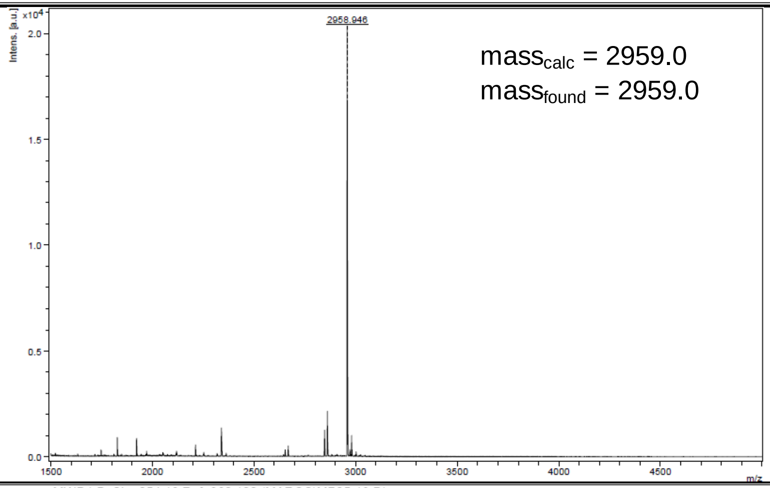
10mer 7De8a-dATC **6**<br>Analytical RP-HPLC-trace	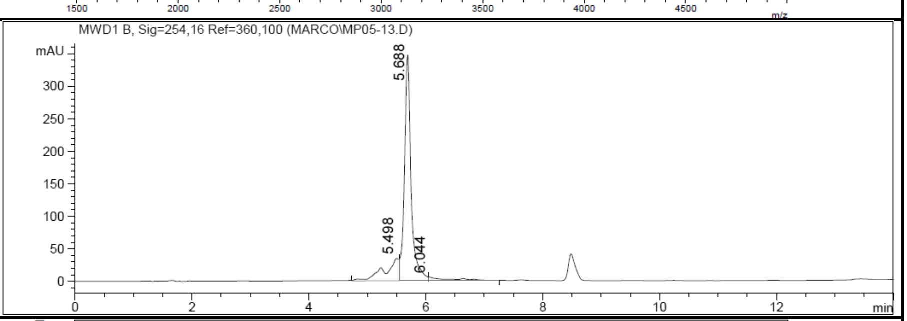
MALDI-MS spectrum	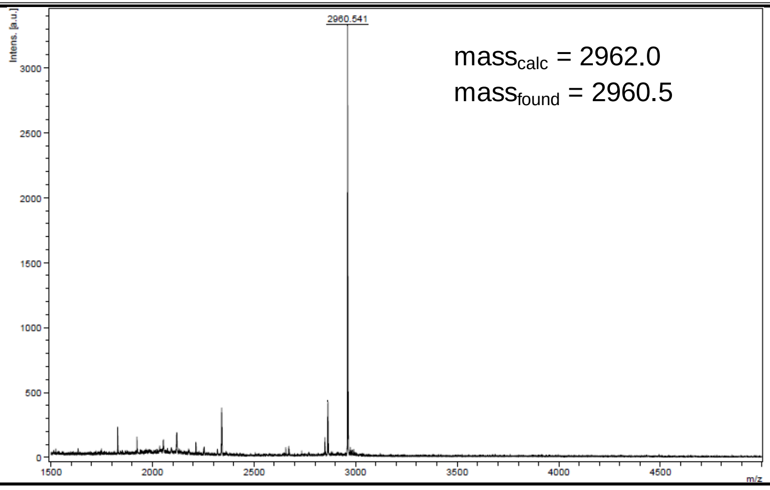

CPG-oligonucleotide + Co(acac)$_3$

According to the representative procedure RP-02 solid support-coupled oligonucleotide (20 nmol) was treated with Co(acac)$_3$.

CPG-oligonucleotide	Analytical data
10mer 7De-dATC **5**<br><br>Analytical RP-HPLC-trace	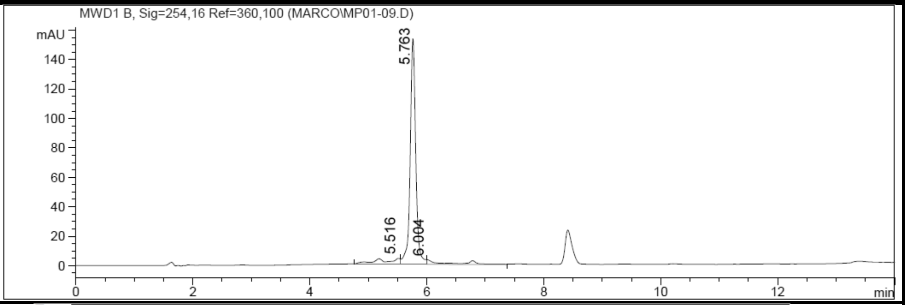
MALDI-MS spectrum	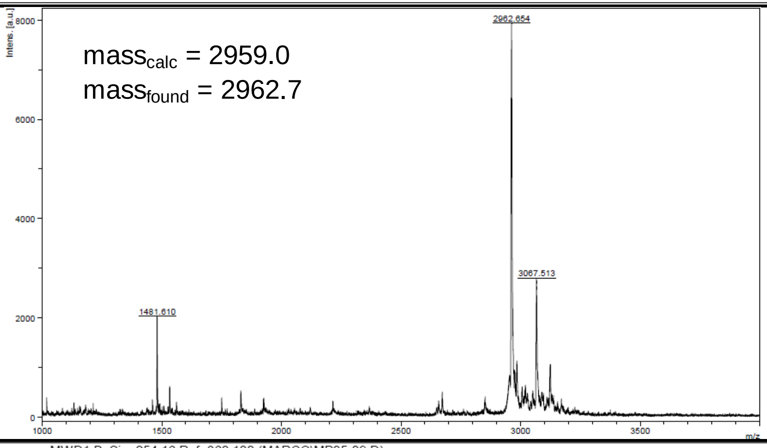
10mer 7De8a-dATC **6**<br><br>Analytical RP-HPLC-trace	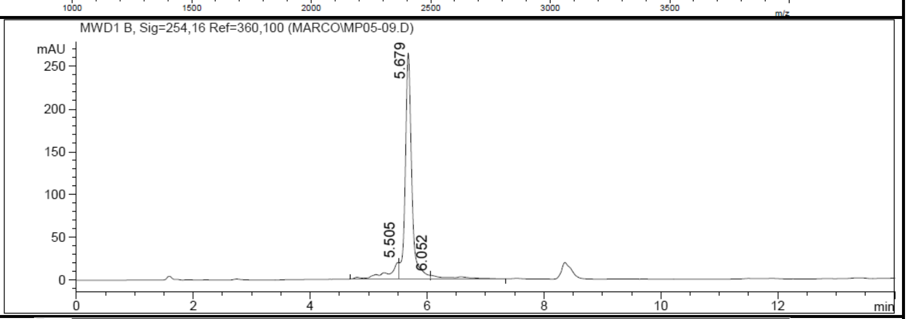
MALDI-MS spectrum	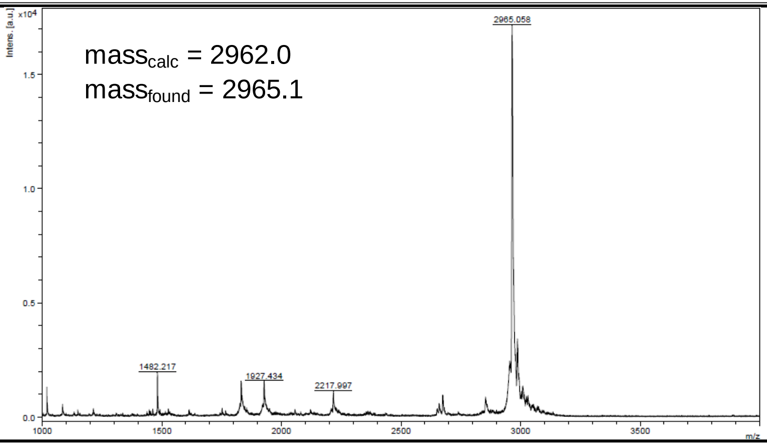

CPG-oligonucleotide + $Cu(MeCN)_4PF_6$

According to the representative procedure RP-02 solid support-coupled oligonucleotide (20 nmol) was treated with $Cu(MeCN)_4PF_6$.

CPG-oligonucleotide	Analytical data
10mer 7De-dATC **5**<br><br>Analytical RP-HPLC-trace	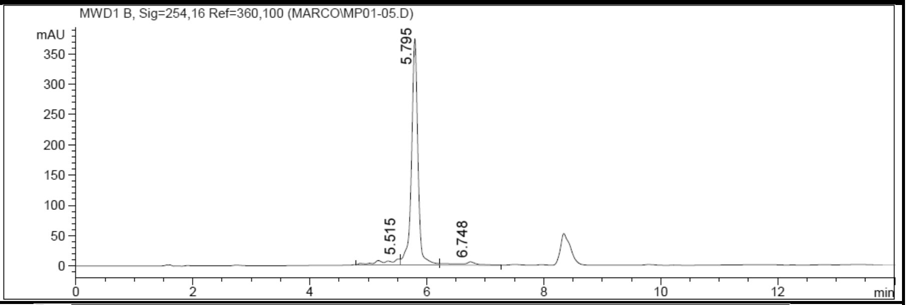
MALDI-MS spectrum	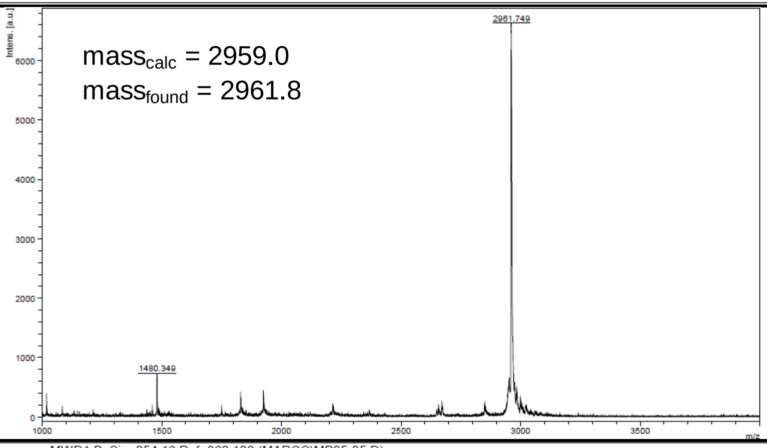
10mer 7De8a-dATC **6**<br><br>Analytical RP-HPLC-trace	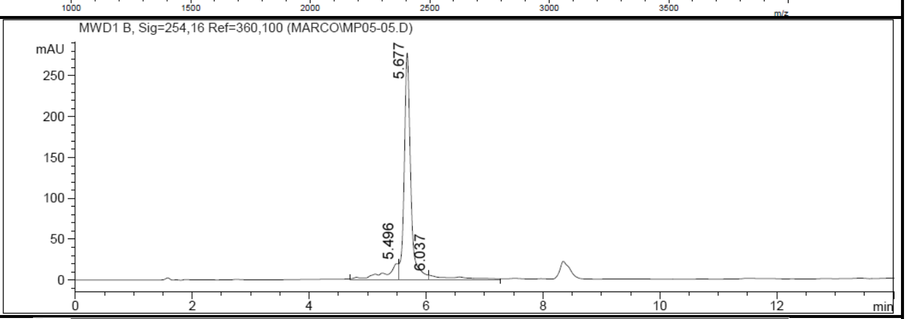
MALDI-MS spectrum	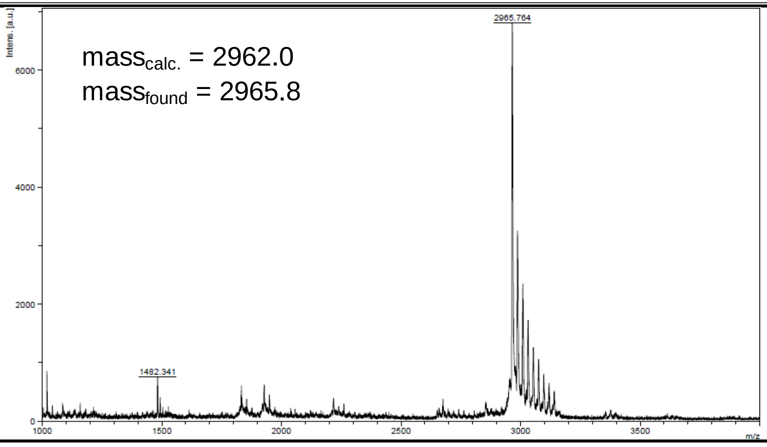

CPG-oligonucleotide + $FeCl_2 \cdot 4\ H_2O$

According to the representative procedure RP-02 solid support-coupled oligonucleotide (20 nmol) was treated with $FeCl_2 \cdot 4\ H_2O$.

CPG-oligonucleotide	Analytical data
10mer 7De-dATC **5**<br>Analytical RP-HPLC-trace	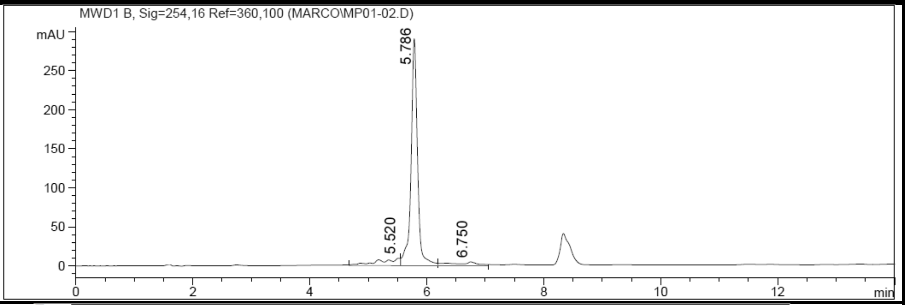
MALDI-MS spectrum	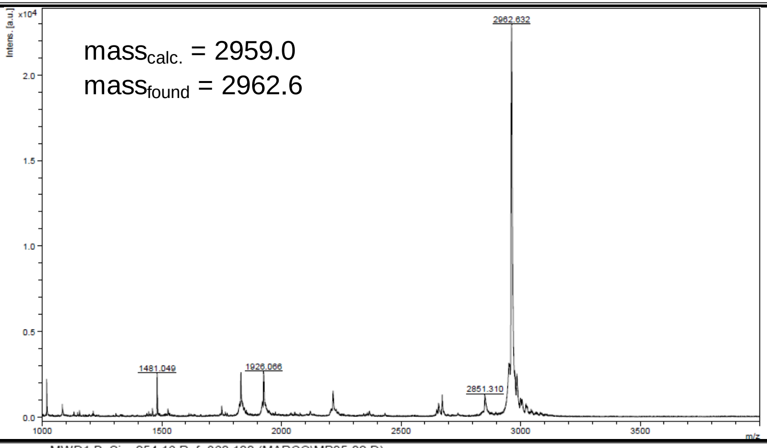<br>$mass_{calc.}$ = 2959.0<br>$mass_{found}$ = 2962.6
10mer 7De8a-dATC **6**<br>Analytical RP-HPLC-trace	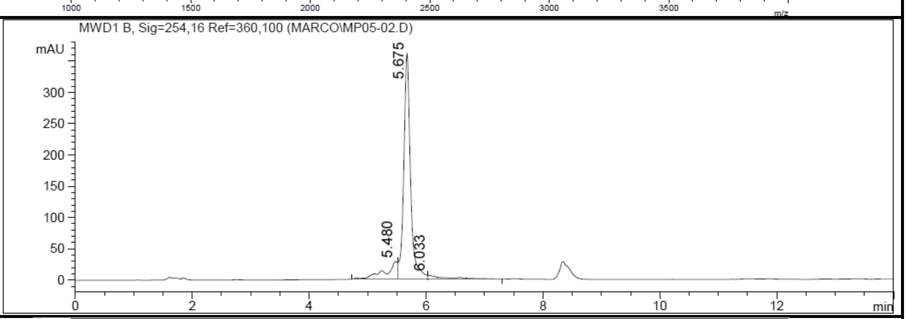
MALDI-MS spectrum	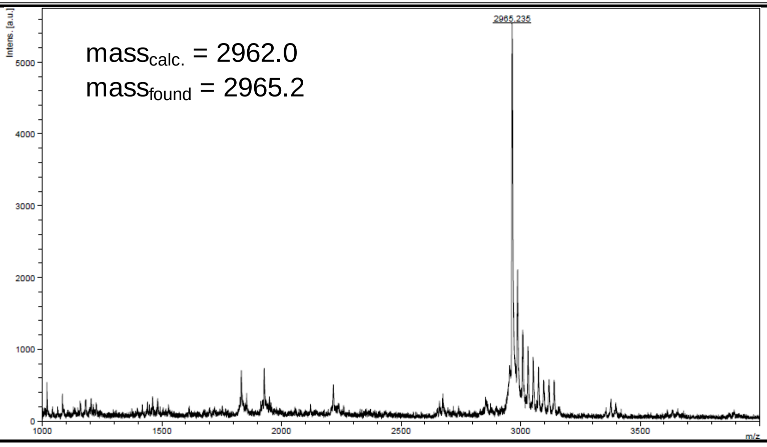<br>$mass_{calc.}$ = 2962.0<br>$mass_{found}$ = 2965.2

CPG-oligonucleotide + La(O*i*-Pr)$_3$

According to the representative procedure RP-02 solid support-coupled oligonucleotide (20 nmol) was treated with La(O*i*-Pr)$_3$.

CPG-oligonucleotide	Analytical data
10mer 7De-dATC **5**<br>Analytical RP-HPLC-trace	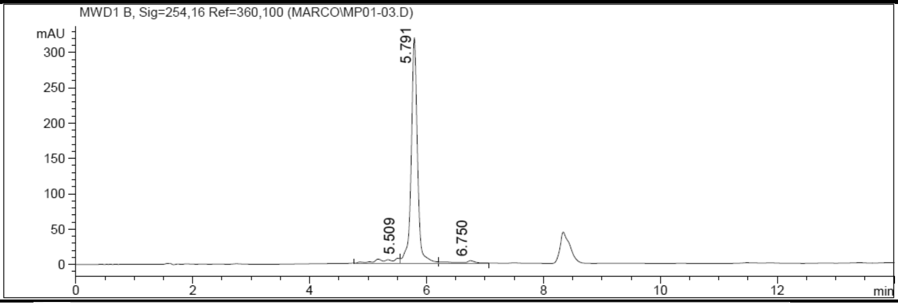
MALDI-MS spectrum	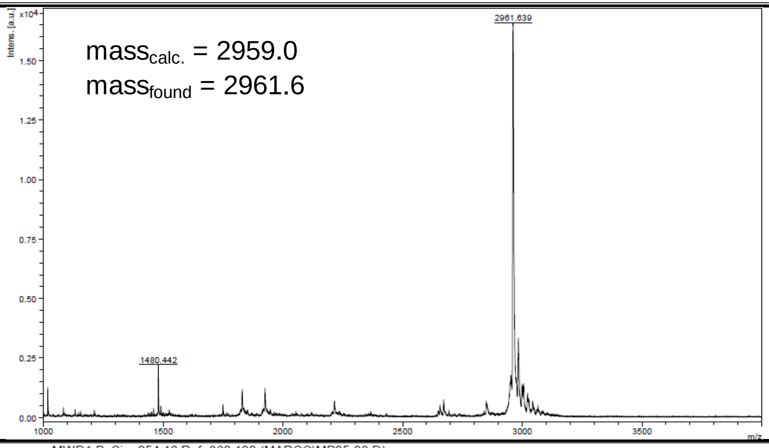
10mer 7De8a-dATC **6**<br>Analytical RP-HPLC-trace	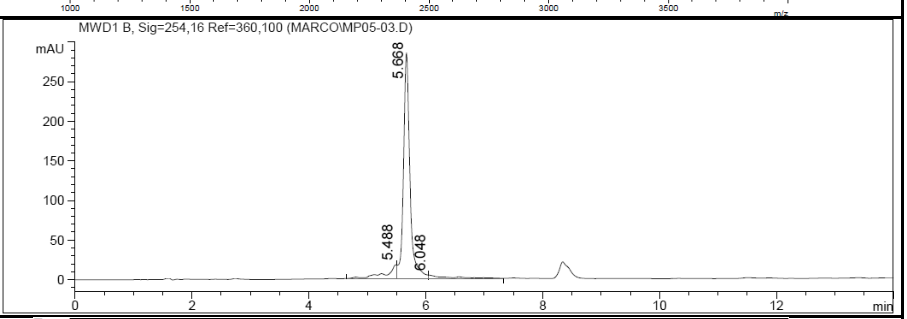
MALDI-MS spectrum	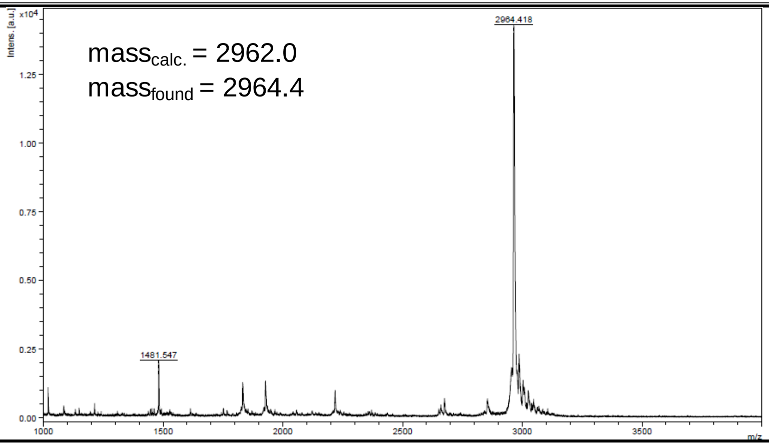

CPG-oligonucleotide + LiBr

According to the representative procedure RP-02 solid support-coupled oligonucleotide (20 nmol) was treated with LiBr.

CPG-oligonucleotide	Analytical data
10mer 7De-dATC **5**<br>Analytical RP-HPLC-trace	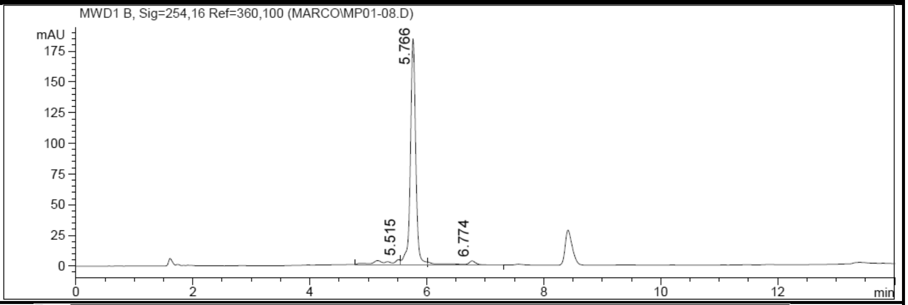
MALDI-MS spectrum	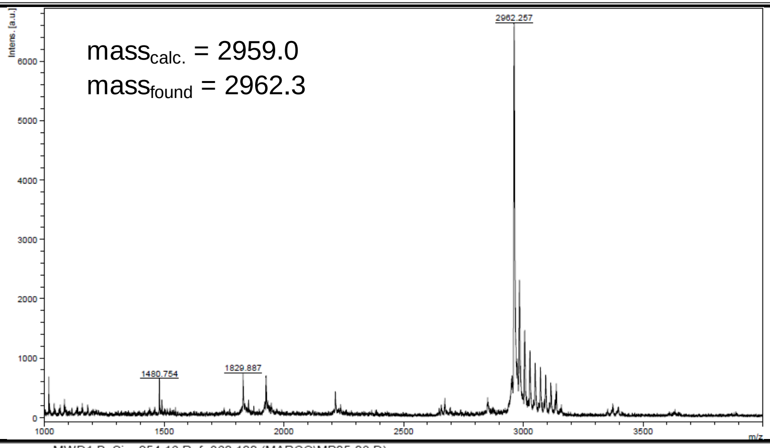
10mer 7De8a-dATC **6**<br>Analytical RP-HPLC-trace	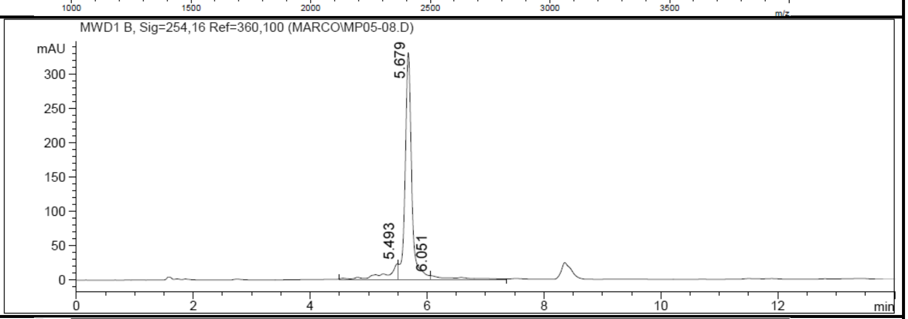
MALDI-MS spectrum	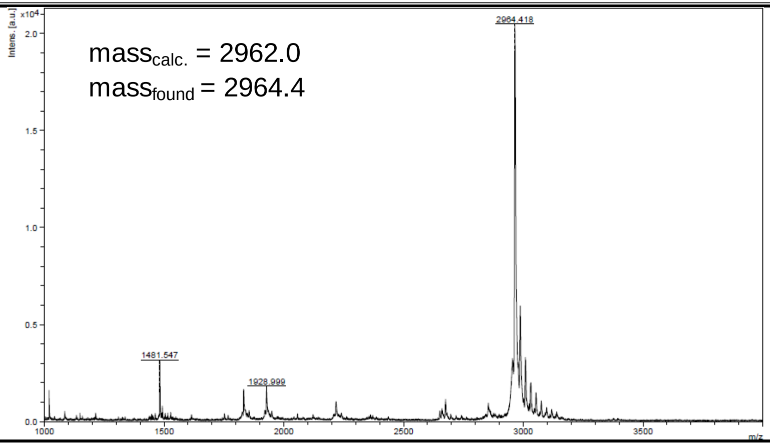

CPG-oligonucleotide + $Ni(acac)_2$

According to the representative procedure RP-02 solid support-coupled oligonucleotide (20 nmol) was treated with $Ni(acac)_2$.

CPG-oligonucleotide	Analytical data
10mer 7De-dATC **5**<br>Analytical RP-HPLC-trace	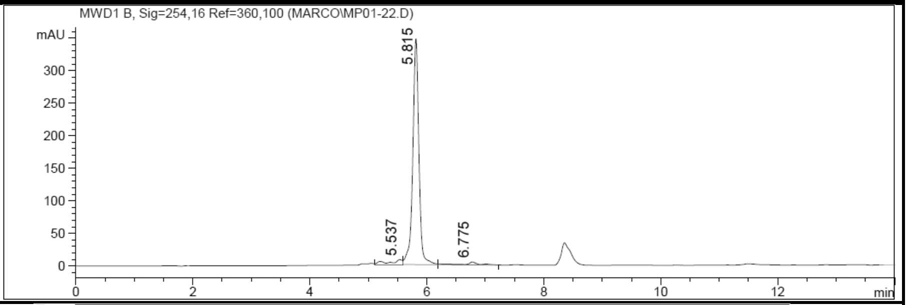
MALDI-MS spectrum	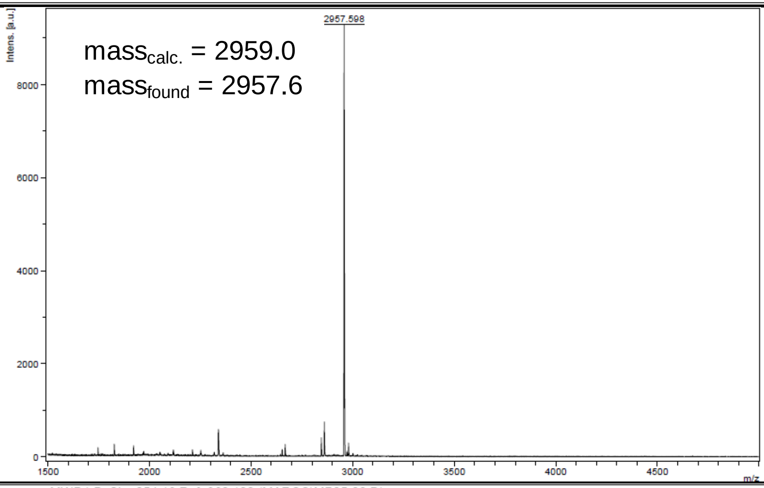<br>$mass_{calc.}$ = 2959.0<br>$mass_{found}$ = 2957.6
10mer 7De8a-dATC **6**<br>Analytical RP-HPLC-trace	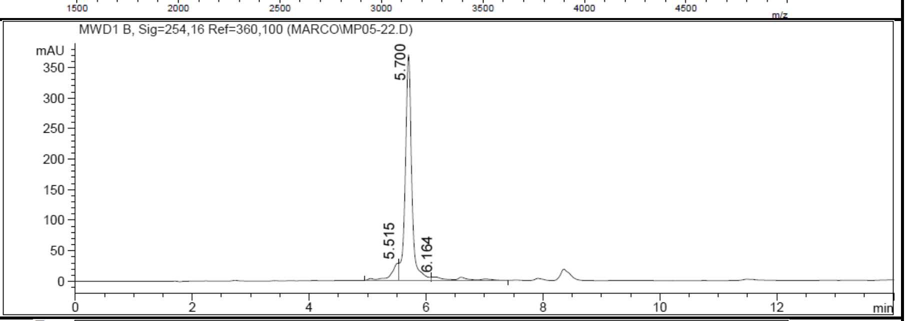
MALDI-MS spectrum	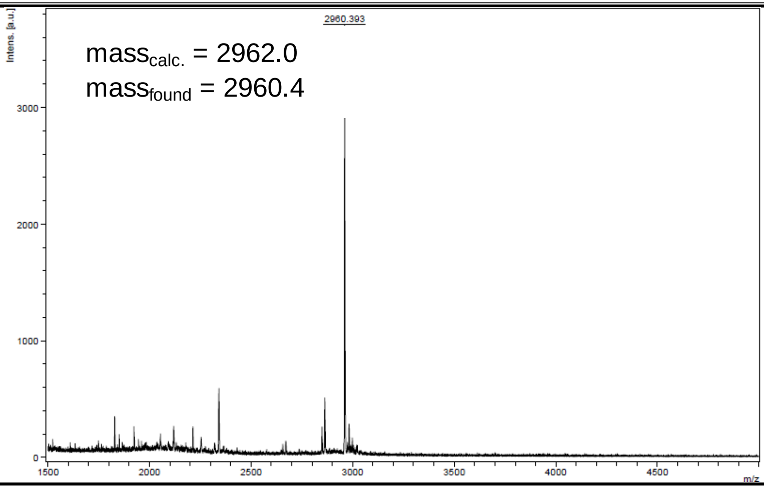<br>$mass_{calc.}$ = 2962.0<br>$mass_{found}$ = 2960.4

CPG-oligonucleotide + $PdOAc_2$

According to the representative procedure RP-02 solid support-coupled oligonucleotide (20 nmol) was treated with $PdOAc_2$.

CPG-oligonucleotide	Analytical data
10mer 7De-dATC **5**<br>Analytical RP-HPLC-trace	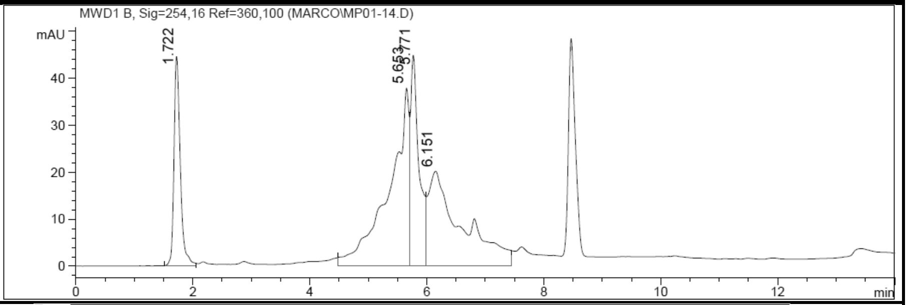
MALDI-MS spectrum	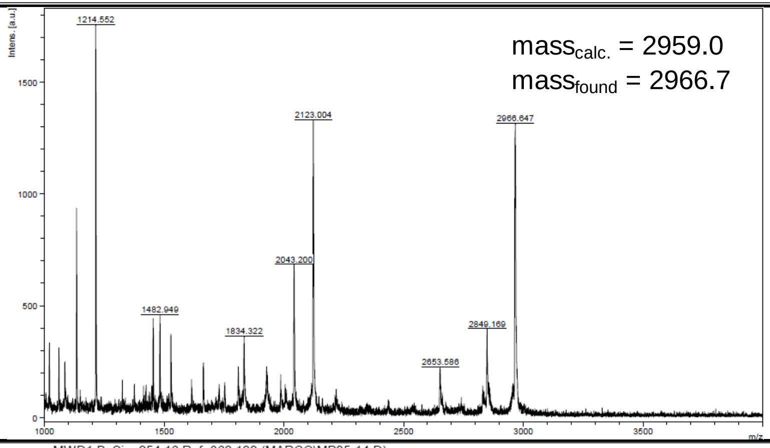
10mer 7De8a-dATC **6**<br>Analytical RP-HPLC-trace	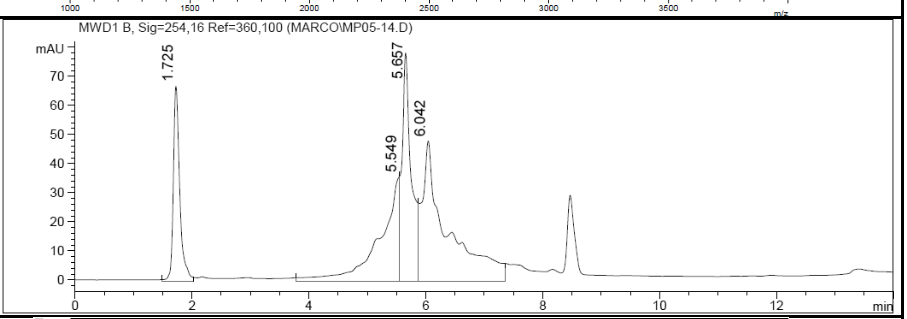
MALDI-MS spectrum	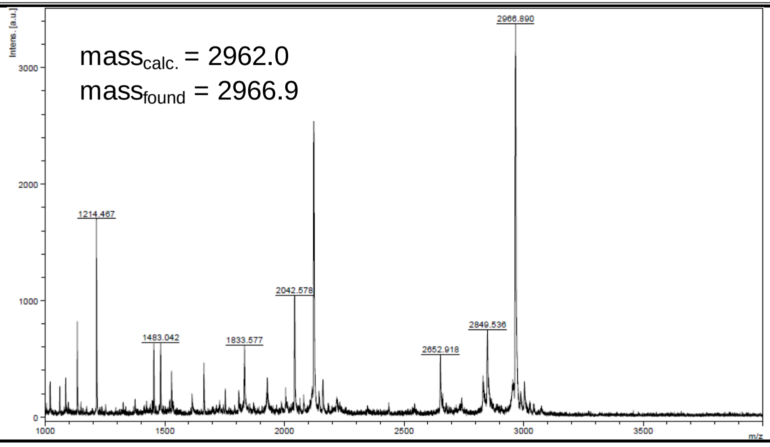

CPG-oligonucleotide + $RuCl_3$

According to the representative procedure RP-02 solid support-coupled oligonucleotide (20 nmol) was treated with $RuCl_3$.

<table>
<tr><th>CPG-oligonucleotide</th><th>Analytical data</th></tr>
<tr><td>10mer 7De-dATC 5

Analytical RP-HPLC-trace</td><td>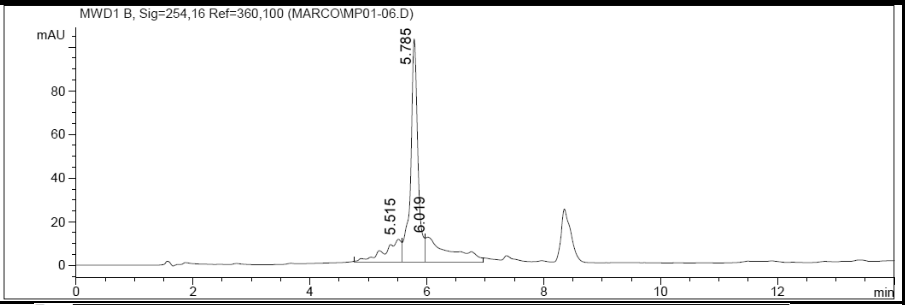
</td></tr>
<tr><td>MALDI-MS spectrum</td><td>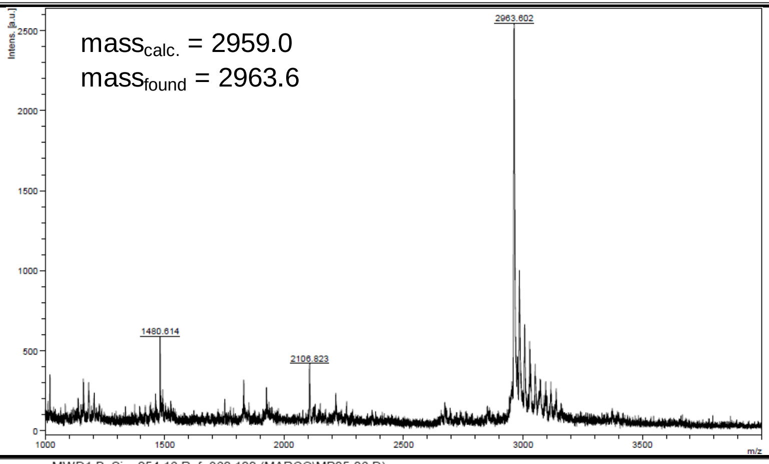
</td></tr>
<tr><td>10mer 7De8a-dATC 6

Analytical RP-HPLC-trace</td><td>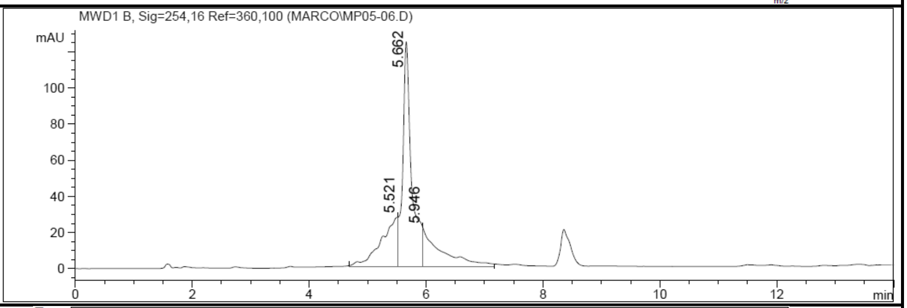
</td></tr>
<tr><td>MALDI-MS spectrum</td><td>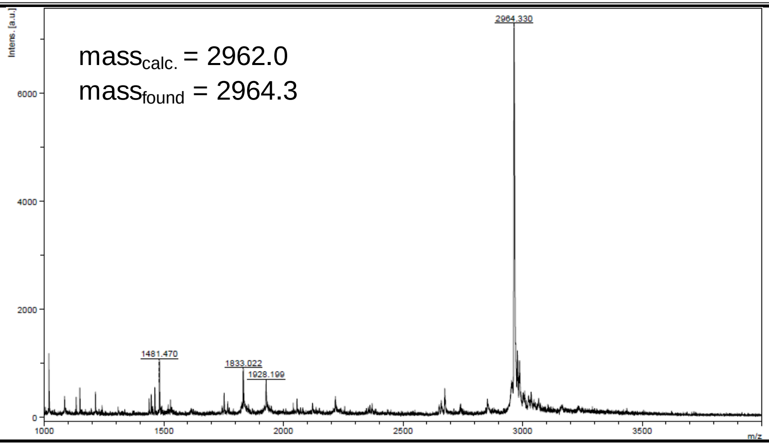
</td></tr>
</table>

CPG-oligonucleotide + $[Ru(p\text{-cymene})Cl_2]_2$

According to the representative procedure RP-02 solid support-coupled oligonucleotide (20 nmol) was treated with $[Ru(p\text{-cymene})Cl_2]_2$.

CPG-oligonucleotide	Analytical data
10mer 7De-dATC **5**<br>Analytical RP-HPLC-trace	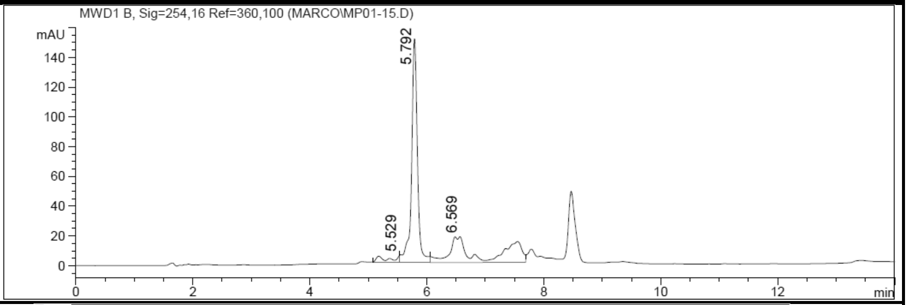
MALDI-MS spectrum	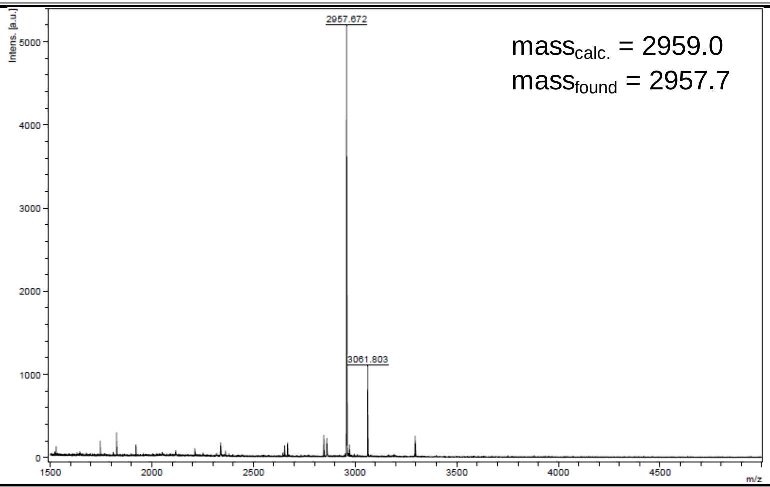
10mer 7De8a-dATC **6**<br>Analytical RP-HPLC-trace	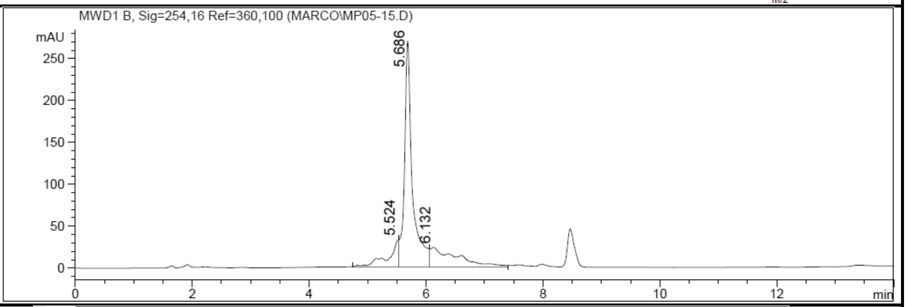
MALDI-MS spectrum	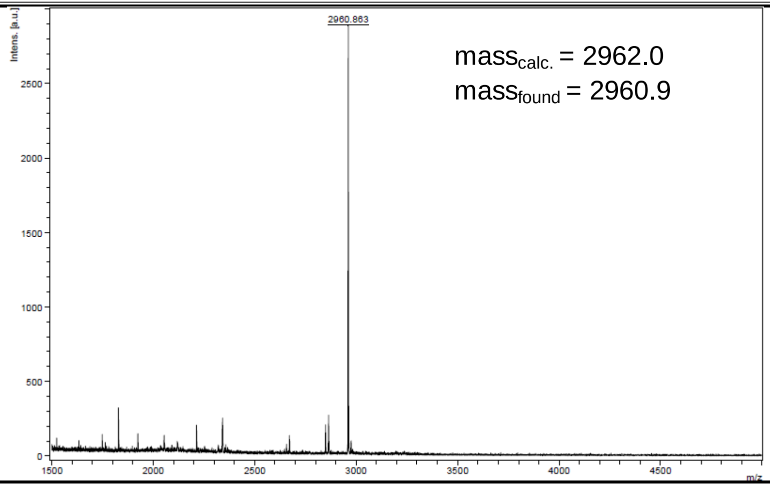

CPG-oligonucleotide + Grubbs 1st Gen.

According to the representative procedure RP-02 solid support-coupled oligonucleotide (20 nmol) was treated with Grubbs 1st Gen..

CPG-oligonucleotide	Analytical data
10mer 7De-dATC **5**<br>Analytical RP-HPLC-trace	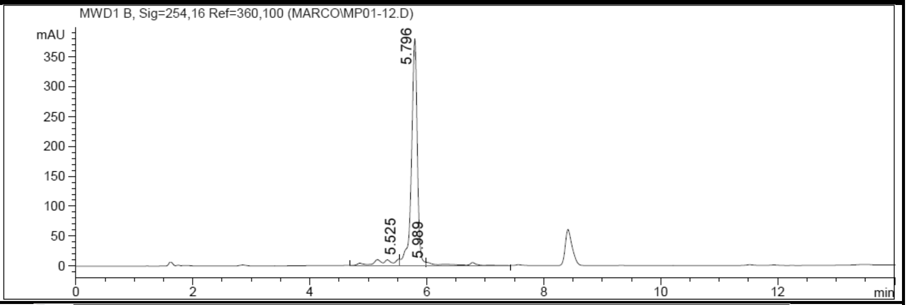
MALDI-MS spectrum	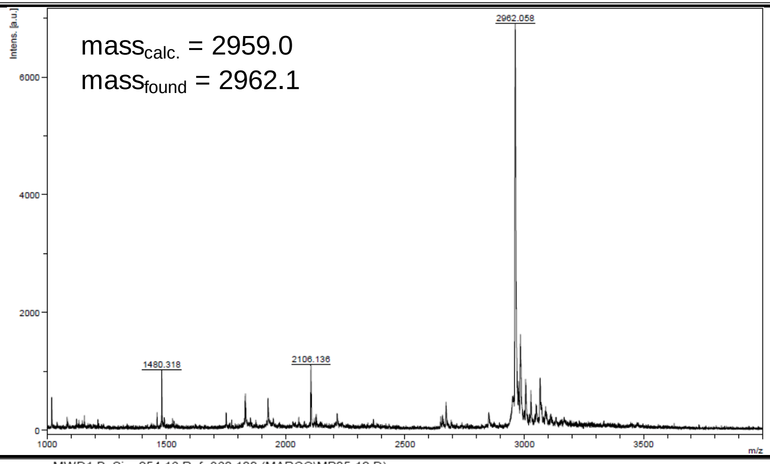
10mer 7De8a-dATC **6**<br>Analytical RP-HPLC-trace	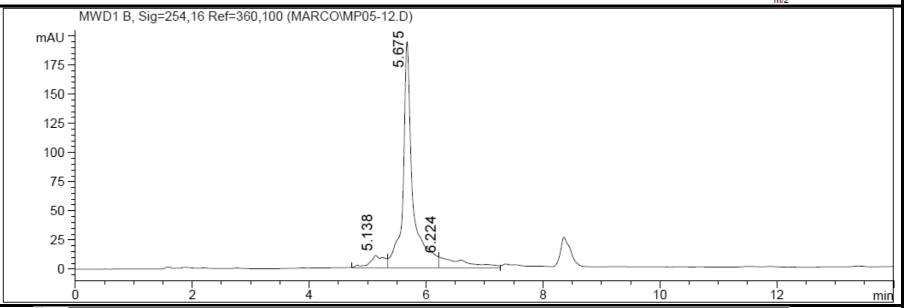
MALDI-MS spectrum	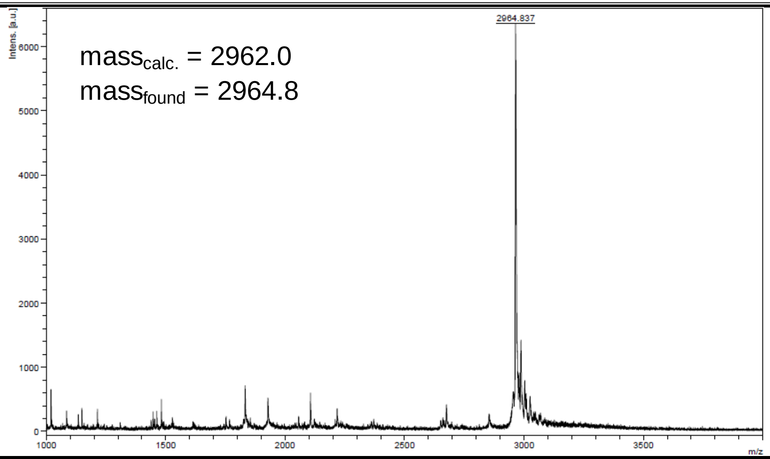

CPG-oligonucleotide + $Sc(OTf)_3$

According to the representative procedure RP-02 solid support-coupled oligonucleotide (20 nmol) was treated with $Sc(OTf)_3$.

CPG-oligonucleotide	Analytical data
10mer 7De-dATC **5**<br><br>Analytical RP-HPLC-trace	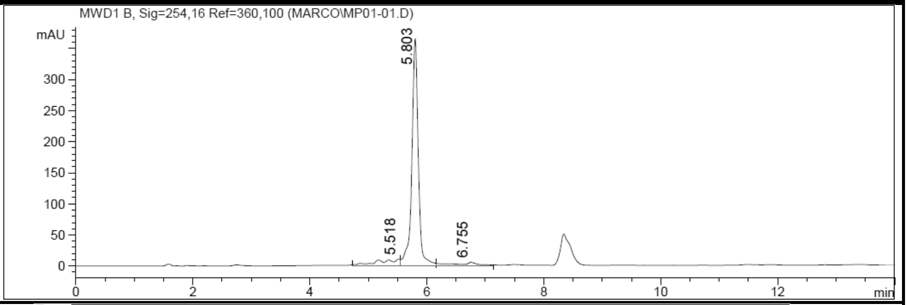
MALDI-MS spectrum	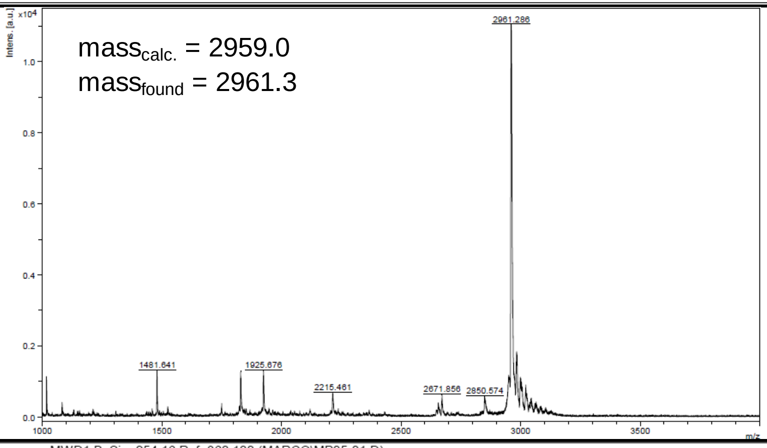
10mer 7De8a-dATC **6**<br><br>Analytical RP-HPLC-trace	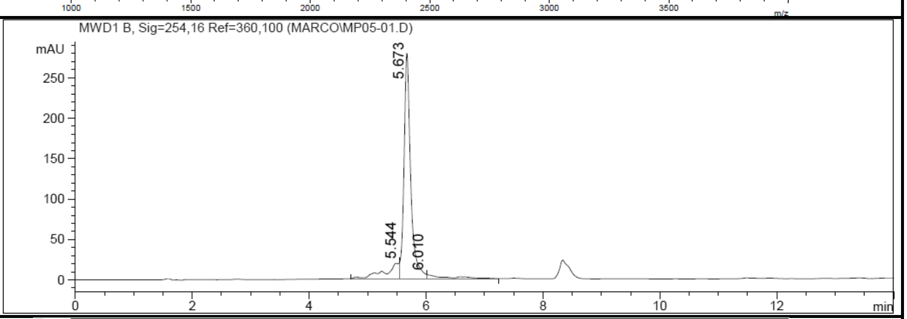
MALDI-MS spectrum	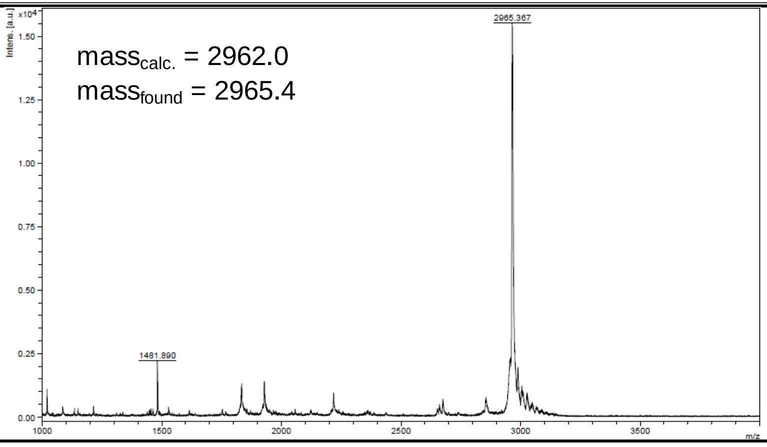

CPG-oligonucleotide + $Sc(OTf)_3$

According to the representative procedure RP-02 solid support-coupled oligonucleotide (20 nmol) was treated with $Sc(OTf)_3$ at 40 °C.

CPG-oligonucleotide	Analytical data
10mer 7De-dATC **5**<br>Analytical RP-HPLC-trace	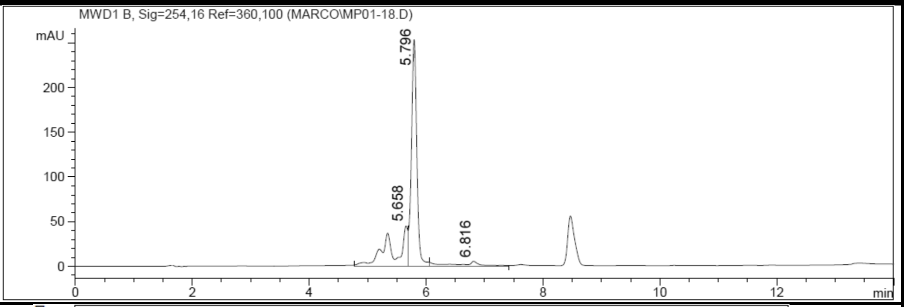
MALDI-MS spectrum	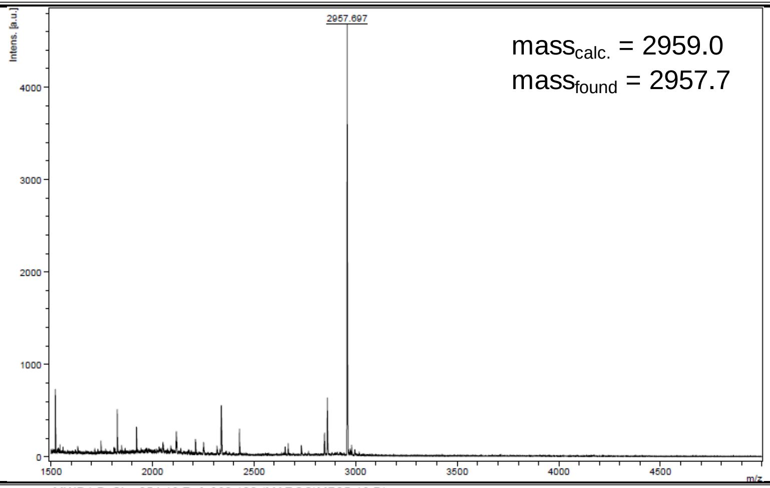
10mer 7De8a-dATC **6**<br>Analytical RP-HPLC-trace	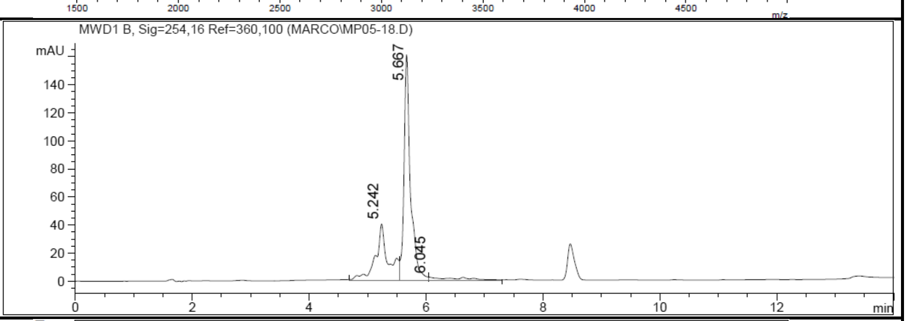
MALDI-MS spectrum	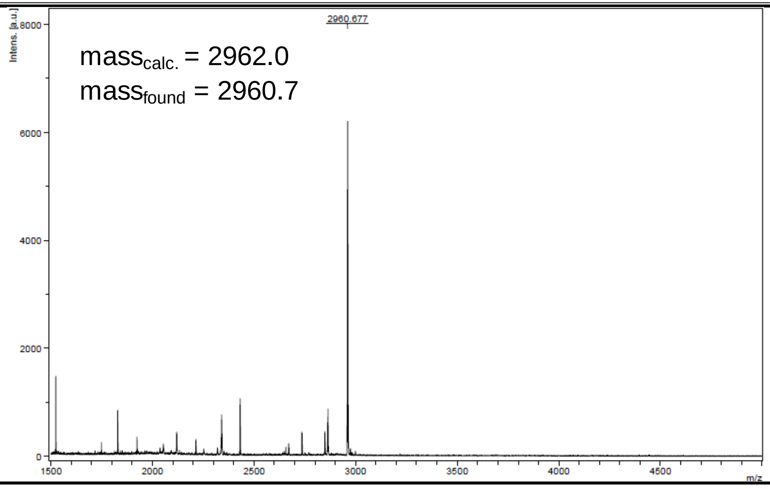

CPG-oligonucleotide + SeO_2

According to the representative procedure RP-02 solid support-coupled oligonucleotide (20 nmol) was treated with SeO_2.

CPG-oligonucleotide	Analytical data
10mer 7De-dATC **5**<br>Analytical RP-HPLC-trace	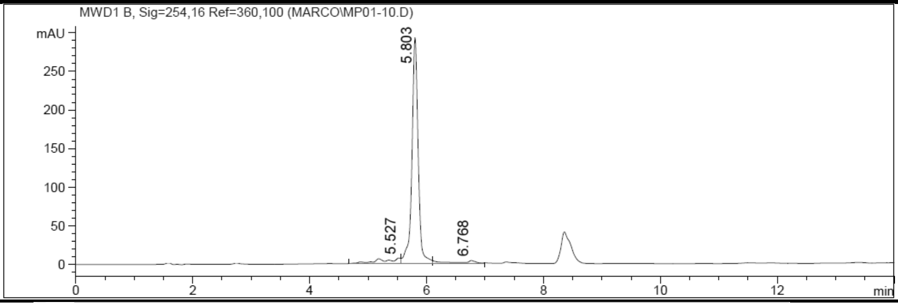
MALDI-MS spectrum	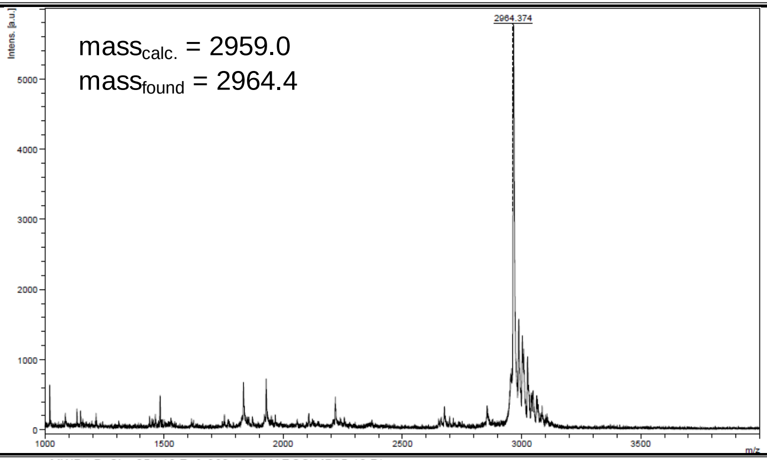<br>$mass_{calc.}$ = 2959.0<br>$mass_{found}$ = 2964.4
10mer 7De8a-dATC **6**<br>Analytical RP-HPLC-trace	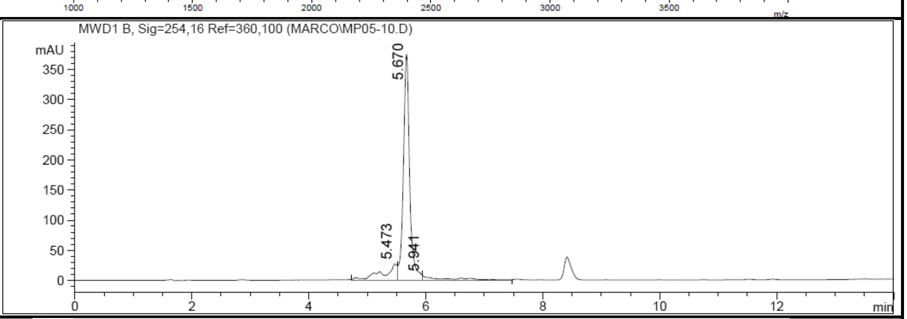
MALDI-MS spectrum	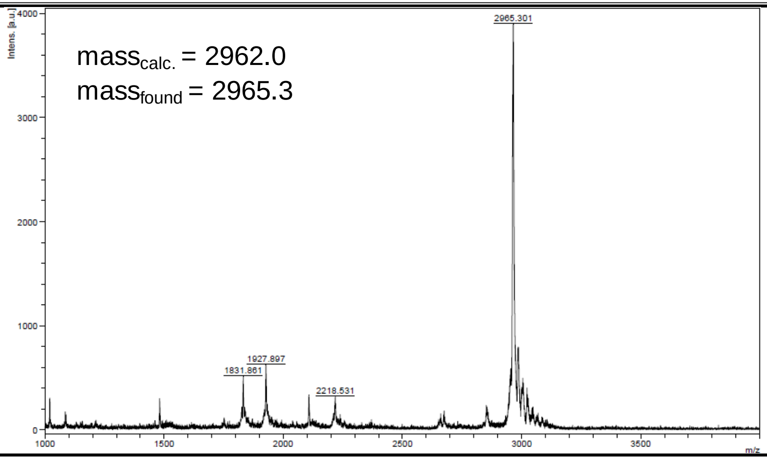<br>$mass_{calc.}$ = 2962.0<br>$mass_{found}$ = 2965.3

CPG-oligonucleotide + $VO(acac)_2$

According to the representative procedure RP-02 solid support-coupled oligonucleotide (20 nmol) was treated with $VO(acac)_2$.

<table>
<tr><th>CPG-oligonucleotide</th><th>Analytical data</th></tr>
<tr><td>10mer 7De-dATC 5

Analytical RP-HPLC-trace</td><td>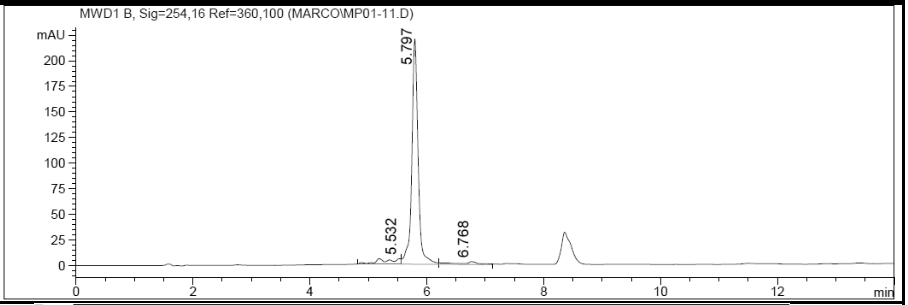
</td></tr>
<tr><td>MALDI-MS spectrum</td><td>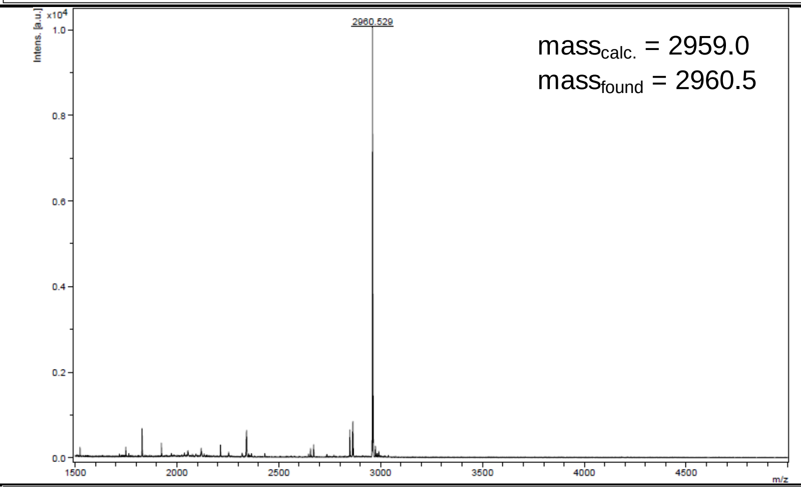
</td></tr>
<tr><td>10mer 7De8a-dATC 6

Analytical RP-HPLC-trace</td><td>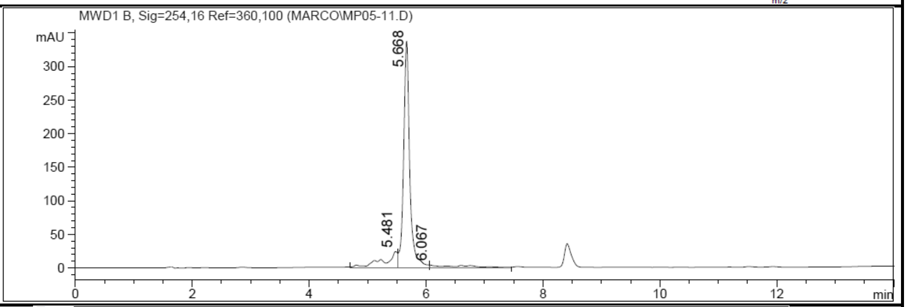
</td></tr>
<tr><td>MALDI-MS spectrum</td><td>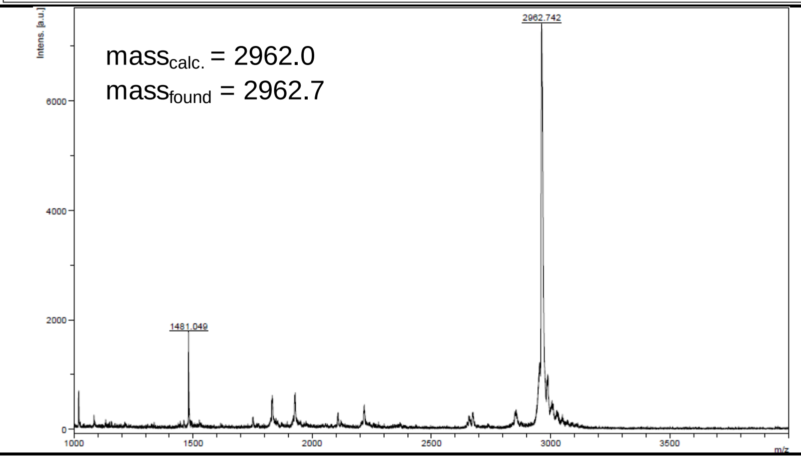
</td></tr>
</table>

CPG-oligonucleotide + $ZnCl_2$

According to the representative procedure RP-02 solid support-coupled oligonucleotide (20 nmol) was treated with $ZnCl_2$.

CPG-oligonucleotide	Analytical data
10mer 7De-dATC **5**<br><br>Analytical RP-HPLC-trace	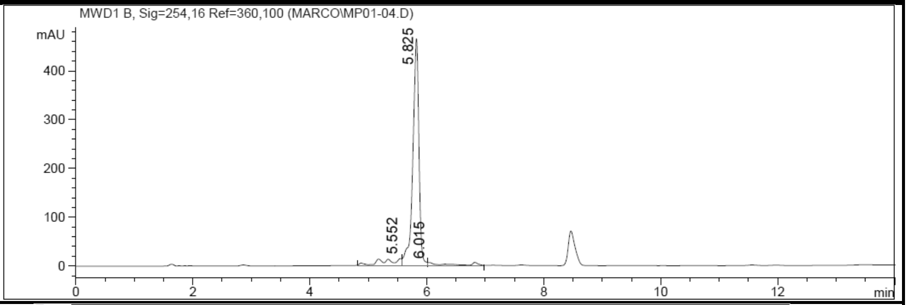
MALDI-MS spectrum	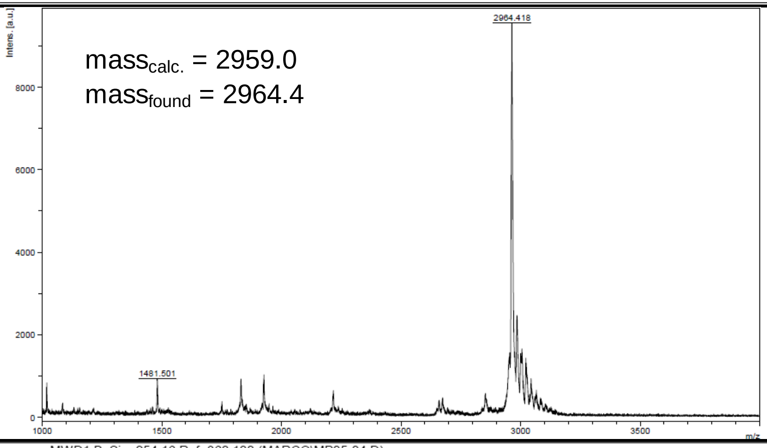
10mer 7De8a-dATC **6**<br><br>Analytical RP-HPLC-trace	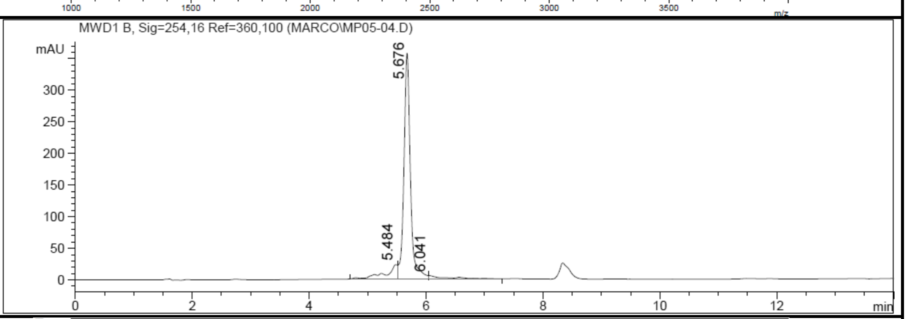
MALDI-MS spectrum	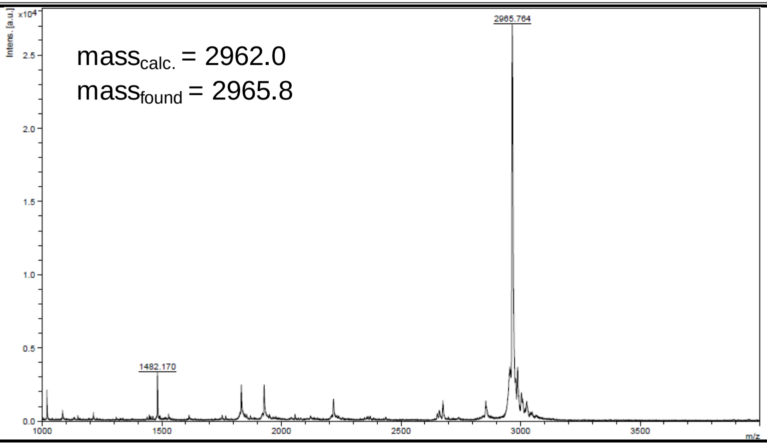

CPG-oligonucleotide + DDQ

According to the representative procedure RP-02 solid support-coupled oligonucleotide (20 nmol) was treated with DDQ.

CPG-oligonucleotide	Analytical data
10mer 7De-dATC **5**<br>Analytical RP-HPLC-trace	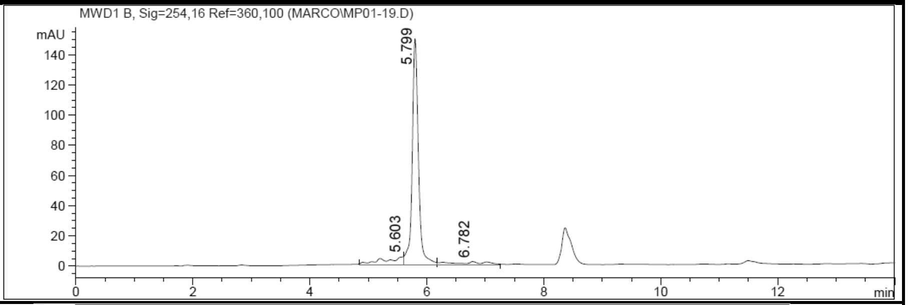
MALDI-MS spectrum	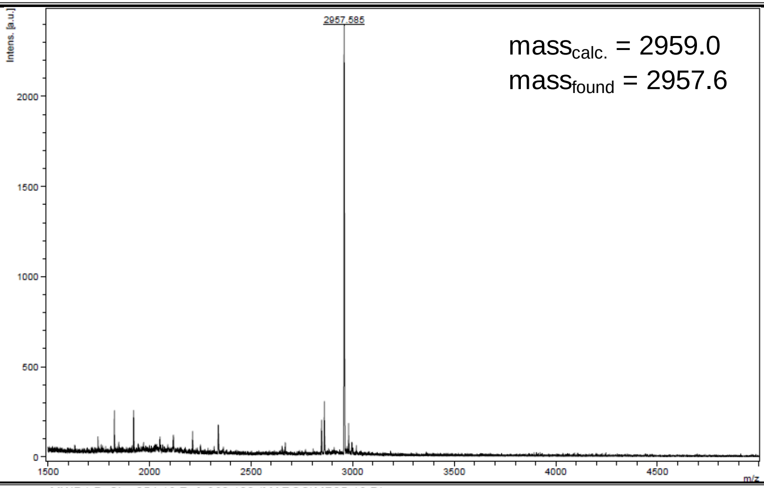
10mer 7De8a-dATC **6**<br>Analytical RP-HPLC-trace	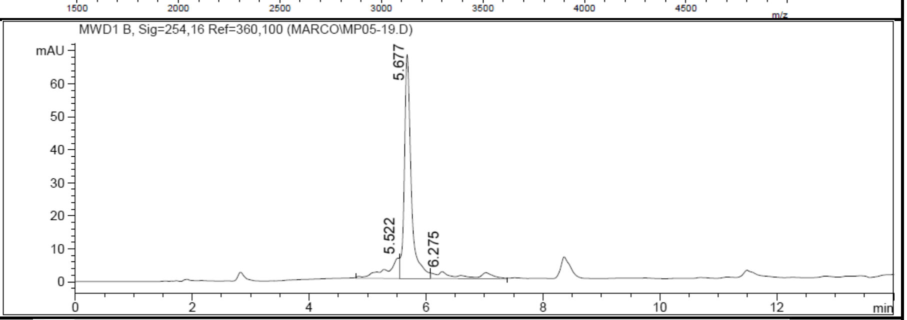
MALDI-MS spectrum	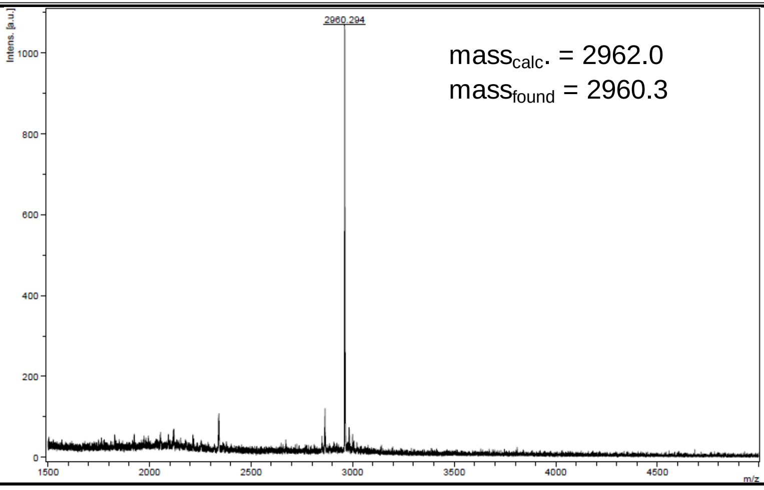

CPG-oligonucleotide + PIDA

According to the representative procedure RP-02 solid support-coupled oligonucleotide (20 nmol) was treated with PIDA.

<table>
<tr><th>CPG-oligonucleotide</th><th>Analytical data</th></tr>
<tr><td>10mer 7De-dATC **5**

Analytical RP-HPLC-trace</td><td>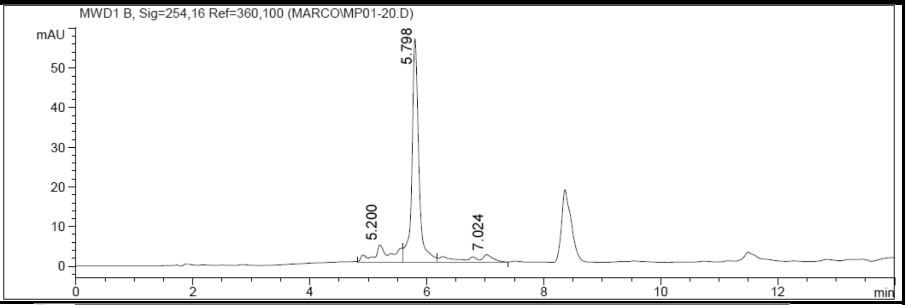
</td></tr>
<tr><td>MALDI-MS spectrum</td><td>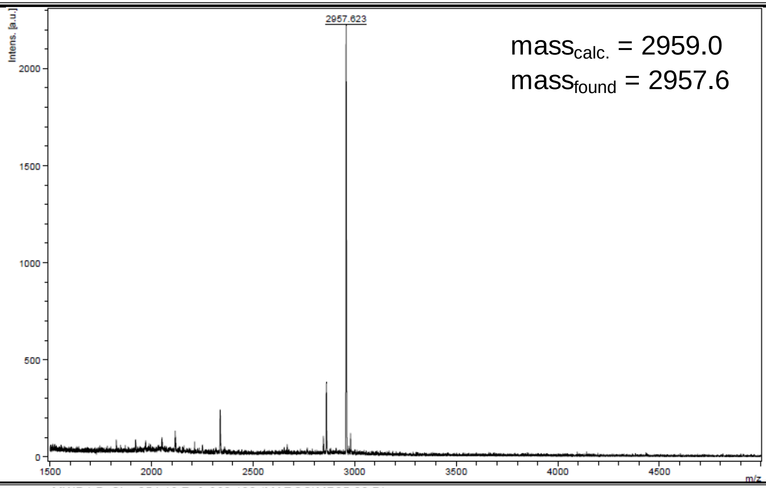
</td></tr>
<tr><td>10mer 7De8a-dATC **6**

Analytical RP-HPLC-trace</td><td>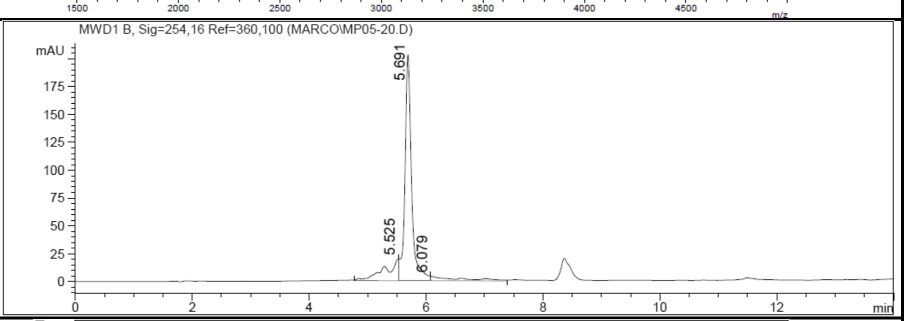
</td></tr>
<tr><td>MALDI-MS spectrum</td><td>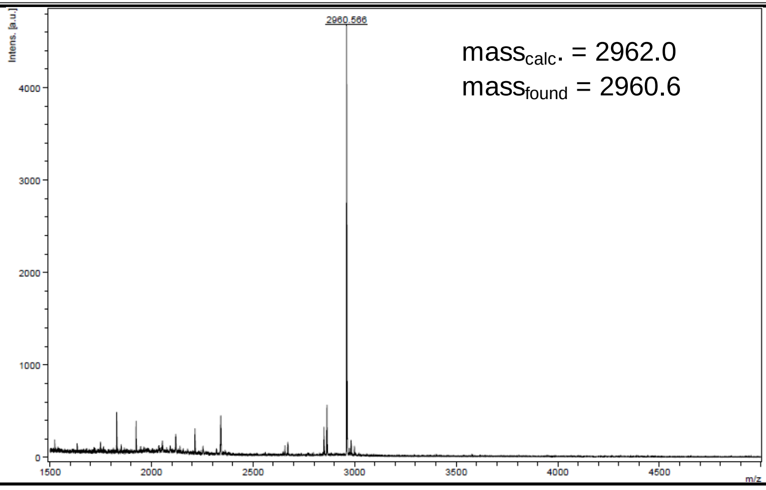
</td></tr>
</table>

CPG-oligonucleotide + TEMPO

According to the representative procedure RP-02 solid support-coupled oligonucleotide (20 nmol) was treated with TEMPO.

<table>
<tr><th>CPG-oligonucleotide</th><th>Analytical data</th></tr>
<tr><td>10mer 7De-dATC 5

Analytical RP-HPLC-trace</td><td>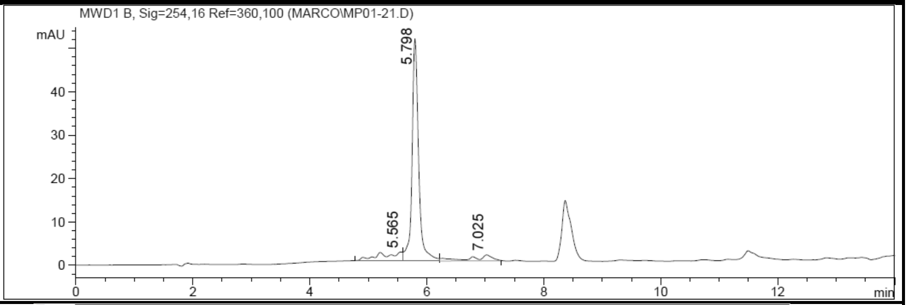
</td></tr>
<tr><td>MALDI-MS spectrum</td><td>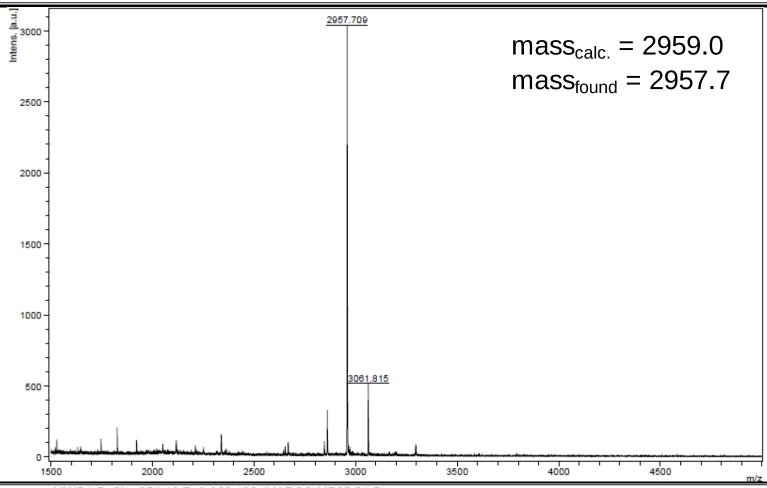

mass$_{calc.}$ = 2959.0
mass$_{found}$ = 2957.7</td></tr>
<tr><td>10mer 7De8a-dATC 6

Analytical RP-HPLC-trace</td><td>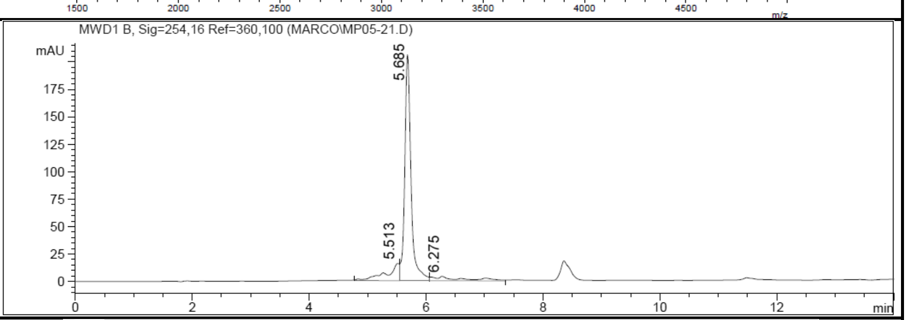
</td></tr>
<tr><td>MALDI-MS spectrum</td><td>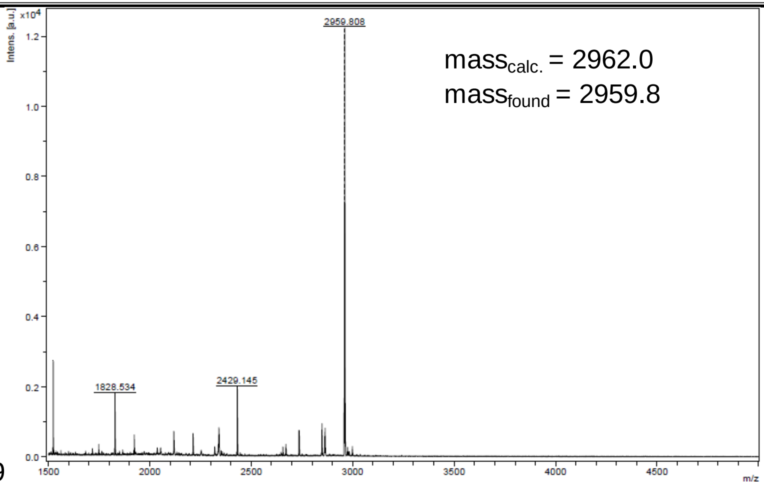

mass$_{calc.}$ = 2962.0
mass$_{found}$ = 2959.8
59</td></tr>
</table>

Biological experiments

T4 ligation and amplification by PCR

Table S6 – Sequences of DNA oligonucleotides *I* – *III*/*III'* and used primer sequences.

DNA	Sequence (5'-3')
HP	CAAATCCGTTCASAGGTCGGTGTGAACGGATTTGAGTC
I (a-f)	CT*CTCT7De-dATCT7De-dAT7De-dACCT<br>CT*CTTC7De-dA7De-dATTCC7De-dACCT<br>CT*CT7De-dAC7De-dACTTT7De-dA7De-dACCT<br>CT*CTC7De-dA7De-dATT7De-dAC7De-dA7De-dACCT<br>CT*CT7De-dACCT7De-dACTT7De-dACCT<br>CT*CTCTTT7De-dA 7De-dACT7De-dACCT
I'	TAG G AG GTi aai iaa iAG AGG ACT
II	GTATCAAGCAGG
II'	TAGGCCTGCTTG
III	CCTACTCTCGTATGACCTCAACTACATGGTCTACA
III'	TGTAGACCATGTAGTTGAGGTCATACGAGAG
forward primer	TCGTCGGCAGCGTCAGATGTGTATAAGAGACAGAGGTCGGTGTGAACGGATTTG
reverse primer	GTCTCGTGGGCTCGGAGATGTGTATAAGAGACAGTGTAGACCATGTAGTTGAGGTCA
forward primer (qPCR)	AGGTCGGTGTGAACGGATTTGAG
reverse primer (qPCR)	GTAGACCATGTAGTTGAGGTCA

S = C_9-Spacer, T* = Ethynyl-dU, i = inosine; a = abasic site.

5'-phosphorylation of DNA

For 5'-phosphorylation of 280 pmol DNA in a total reaction volume of 20 µL,10 units of T4 polynucleotide kinase (T4 PNK, *Thermo Fisher Scientific*), 1x PNK Buffer A (50 mM Tris-HCl, 10 mM $MgCl_2$, 5 mM DTT, 0.1 mM spermidine, pH = 7.6 at 25 °C, *Thermo Fisher Scientific*) and 1 mM ATP (*Thermo Fisher Scientific*) were used. Reaction mixtures were incubated at 37 °C for 20 min, then heat-inactivated at 75 °C for 15 min and slowly cooled down to 4 °C.

Ligation of DNA

Prior to enzymatic ligation of DNA, the oligonucleotides were annealed by incubation at 85 °C for 10 min and cooling down to 4 °C. For ligation (40 µL scale), 100 pmol of each oligonucleotide, 600 units of T4 DNA Ligase (T4 DNA ligase, *New England Biolabs*) and 1x T4 DNA Ligase Buffer (50 mM Tris-HCl, 10 mM $MgCl_2$, 10 mM DTT, 1 mM ATP, pH = 7.5

at 25 °C , *New England Biolabs*) were mixed. Ligation reactions were performed at 25 °C for 16 h, then stopped by heat inactivation at 75 °C for 15 min and cooled down to 4 °C.

Analysis of DNA ligation

For analysis of DNA ligation reactions, agarose gel electrophoresis was performed using a 3% or 4% agarose gel. Electrophoresis was carried out in TBE buffer (89 mM Tris-borate, 2 mM EDTA, pH = 8.3) at 100 V constant voltage for 15 min and then 150 V constant voltage for about 45 min. For staining of the DNA, Midori Green Direct (*NIPPON Genetics*) and as a reference, GeneRuler Ultra Low Range DNA Ladder (*Thermo Fisher Scientific*) was used. Imaging of the gels was performed using the *Bio-Rad* Gel Doc™ XR system.

Purification of DNA by ethanol precipitation

After the first and second ligation, the DNA was precipitated by adding 1/10 volume of 3 M aq. sodium acetate (pH = 5.2) and 3 volumes of 100% ethanol and incubating this solution for about 4 h or overnight at -80 °C. Afterwards the samples were centrifuged at 4 °C for 30 min (13200 rpm; Centrifuge 5415 R, *Eppendorf*), the supernatant was taken off, additional 100 µL of 100% ethanol were added and the solution was incubated for 1 h at -80 °C. Afterwards the samples were centrifuged at 4 °C for 30 min (13200 rpm; Centrifuge 5415R, *Eppendorf*), the supernatant was taken off, and the DNA pellets were dried at 37 °C. The DNA samples were dissolved in ddH_2O.

Purification of DNA by gel extraction

After the third ligation, the DNA samples were gel extracted using the “QIAquick Gel Extraction Kit” (*Qiagen*) according to the manufacturer protocol.

PCR amplification

Following the third ligation, fully encoded DNA was amplified by PCR. Thereby, 5 µL of gel extracted DNA, 5 U of Taq DNA polymerase (*Thermo Fisher Scientific*), 1x Taq Buffer (10 mM Tris-HCl, 50 mM KCl, 0.08% (v/v) Nonidet P40, pH = 8.8 at 25 °C, *Thermo Fisher Scientific*), 3 mM $MgCl_2$, 0.625 mM of each dNTP (dATP, dGTP, dTTP, and dCTP, corresponding to 2.5 mM of the mixture of dNTPs) and 1 µM of the reverse primer in a reaction volume of 39 µL. The PCR program started with pre-denaturation at 95 °C for 3 min, followed by denaturation for 30 s at 95 °C, annealing for 30 s at 55 °C, and elongation for 30 s at 72 °C. After 10 cycles, 1 µM of the forward primer was added and additional 20 cycles were performed. After PCR, the time for elongation was prolonged to 5 min. The PCR products were analyzed by agarose gel electrophoresis.

Silica-membrane-based purification of DNA

PCR products were purified using the “QIAquick PCR Purification Kit” (*Qiagen*) according to the manufacturer´s protocol.

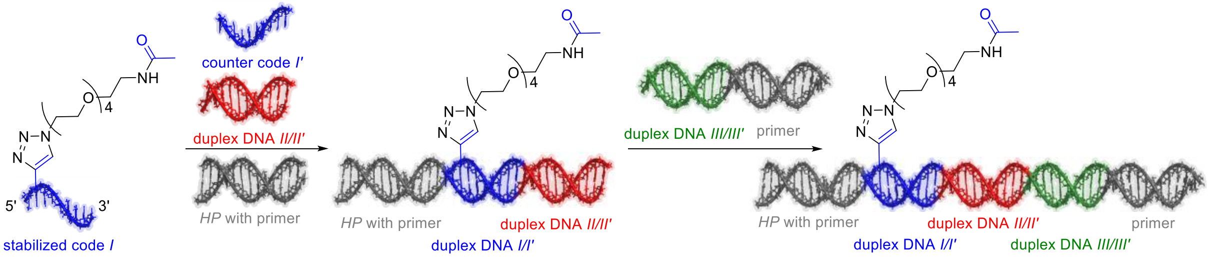

Figure S1 – Encoding scheme for test ligations with chemically stabilized barcodes *I*.

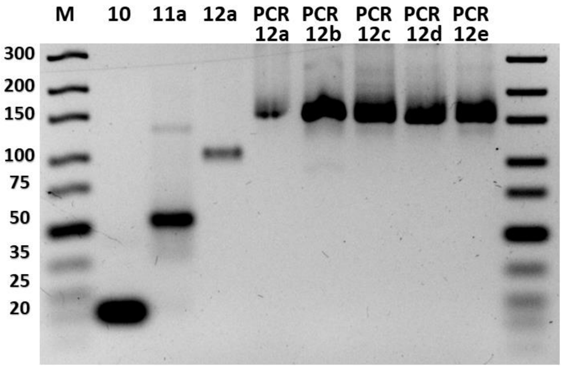

Figure S2 – Agarose gel (4%) of hairpin-based encoding strategy and PCR amplification of fully encoded DNA using stabilized DNA in barcode *I*. Stabilized DNA barcode *I* was ligated to hairpin *HP*, DNA *I'* and to DNA duplexes *II/II'* in one pot to encode the first building block and the heterocyclic scaffold yielding the duplex *HP-I-II/I'-II'.* Then, DNA duplex *HP-I-II/I'-II'* was ligated to DNA duplexes *III/III'* to encode acid building blocks. Lane 1: hairpin *HP*, lane 2: ligation of stabilized DNA barcode *I* to hairpin *HP* and DNA duplexes *II/II'*, lane 3: ligation of DNA duplex *HP-I-II/I'-II'* to DNA duplexes *III/III'*, lane 4: PCR amplification of DNA duplex *HP-I-II-III/I'-II'-III'.*

Sanger sequencing

Sanger sequencing of purified PCR products was performed by *Microsynth Seqlab GmbH* (Göttingen, Germany). The sequencing of DNA sequences containing adjacent adenosines using the forward primer did not lead to two defined peaks in the chromatogram. This was observed for oligonucleotides with chemically modified adenosines as well as for the native DNA oligonucleotides. However, sequencing of the same DNA sequences using the reverse primer gave the expected results. The sequencing data was analyzed with *Benchling* [Biology Software] (2020). Retrieved from https://benchling.com.

Table S7 – Sanger sequencing results of the PCR products of encoded sequences containing five different stabilized codes *Ia-e* (T* = Ethynyl-dU)

Code *Ia* **5'-CT*C TCT 7De-dATC T7De-dAT 7De-dACC T-3'**

forward primer

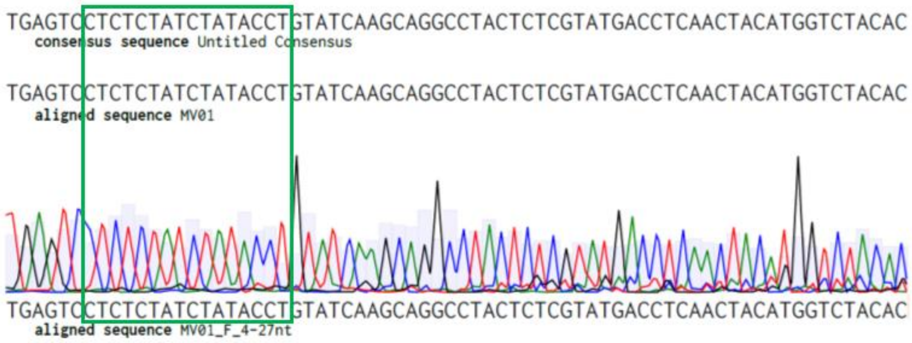

reverse primer

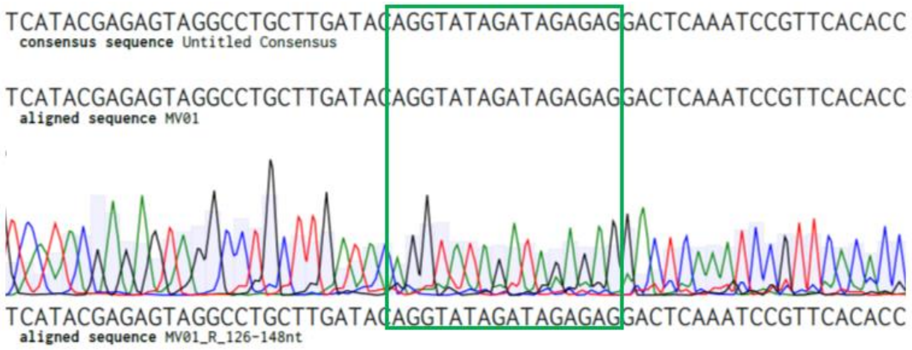

Code *Ib* **5'-CT*C TTC 7De-dA 7De-dAT TCC 7De-dACC T-3'**

forward primer

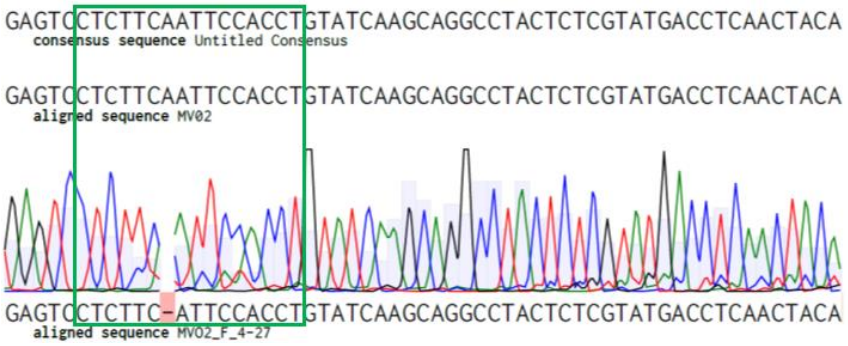

reverse primer

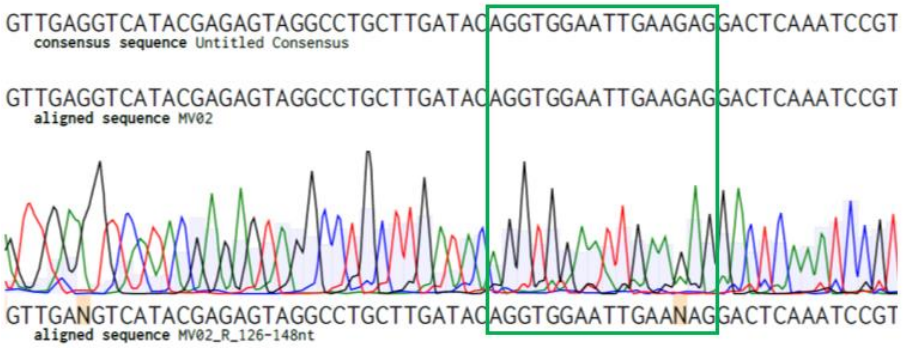

**Code *Ic***	**5'-CT*C T7De-dAC 7De-dACT TT7De-dA 7De-dACC T-3'**

forward primer

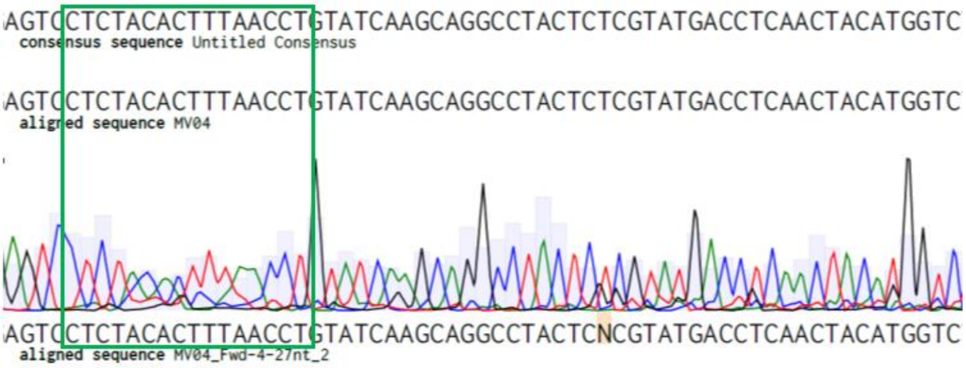

reverse primer

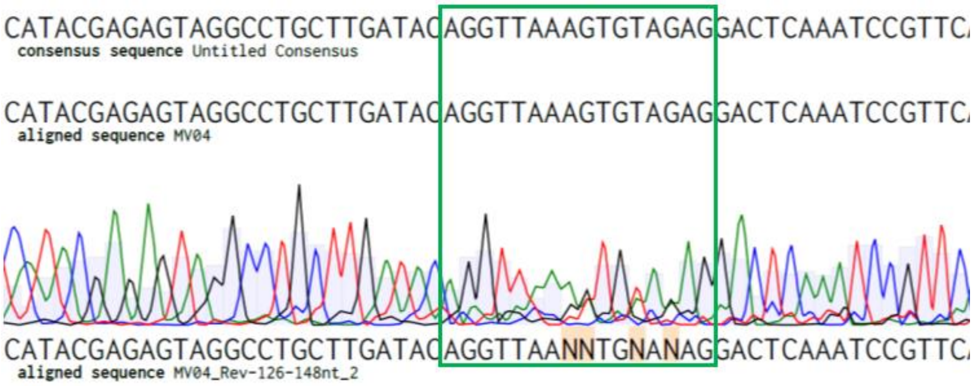

**Code *Id***	**5'-CT*C TC7De-dA 7De-dATT 7De-dAC7De-dA 7De-dACC T-3'**

forward primer

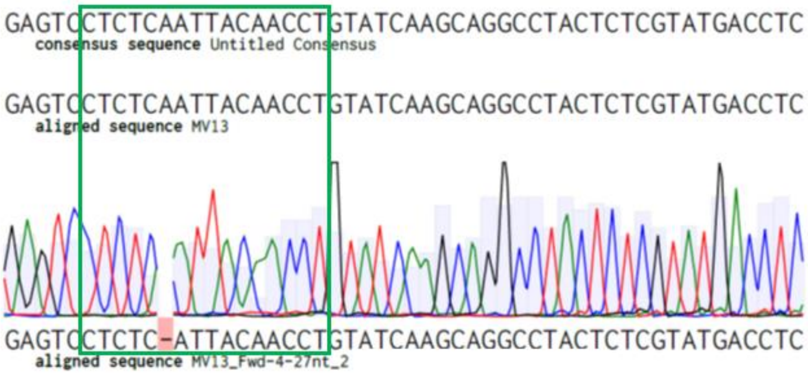

reverse primer

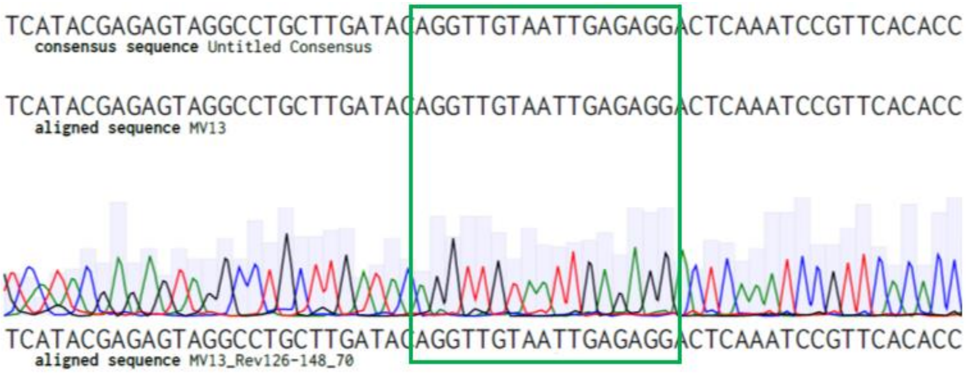

**Code *le***	**5'-CT*C T7De-dAC CT7De-dA CTT 7De-dACC T-3'**

forward primer

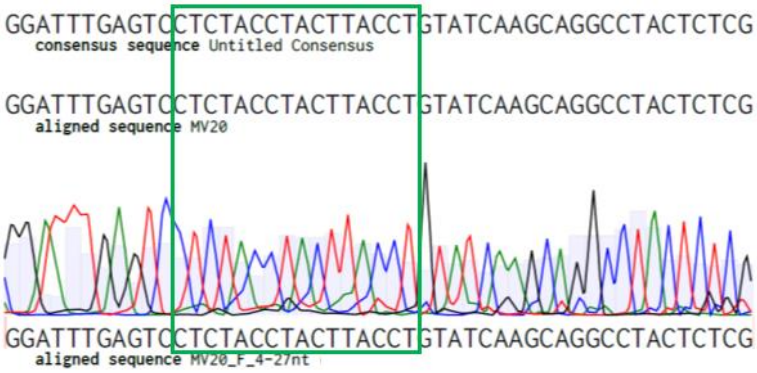

reverse primer

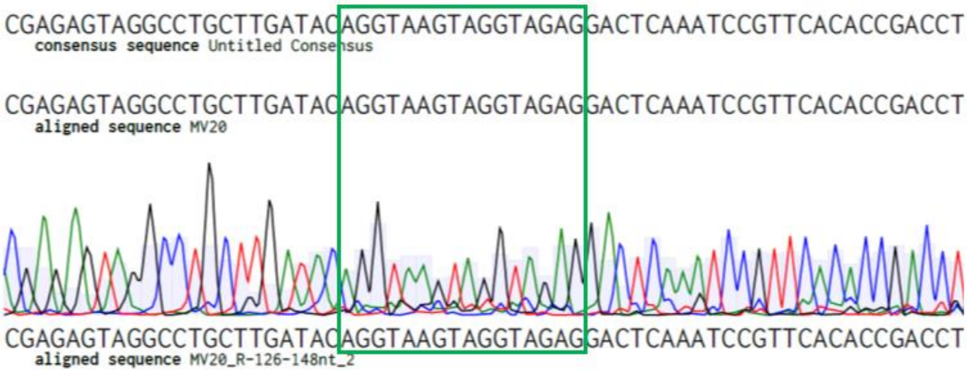

qPCR

For qPCR experiments the following were combined in PCR plate wells (GK480K-50, *Kisker*) in a total volume of 20 µL: DNA template (5 µL, ligation product 3), 200 nM forward primer (0.8 µL, 5 µM stock), 200 nM reverse primer (0.8 µL, 5 µM stock), SsoAdvanced universal SYBR® Green supermix (10 µL, *Bio-Rad*) and H_2O (3.4 µL).

For all qPCR experiments the following amplification method using the LightCycler® 480 II system from *Roche* was performed: hot start at 95 °C for 30 s, then 35 cycles of 95 °C for 15 s (denaturation), 60 °C for 30 s (annealing) and 72 °C for 30 s (elongation).

The specificity of the PCR amplification was analysed by melting curve measurements. Analysis was done with the LightCycler® 480 – software version 1.5.

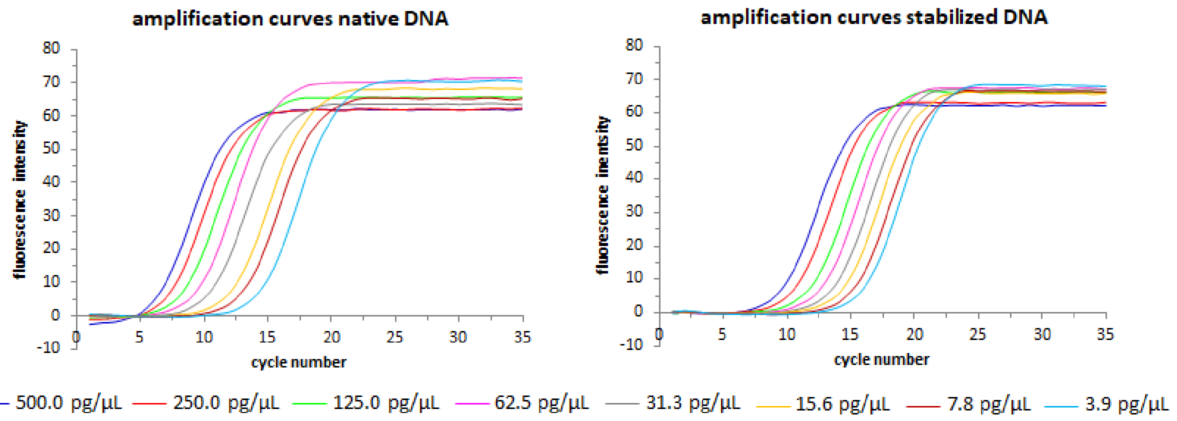

Figure S3 – Amplification curves (qPCR) using different concentrations of DNA sequences containing native or stabilized DNA barcodes *Id* (5'-CT*C TC7De-dA 7De-dATT 7De-dAC7De-dA 7De-dACC T-3').

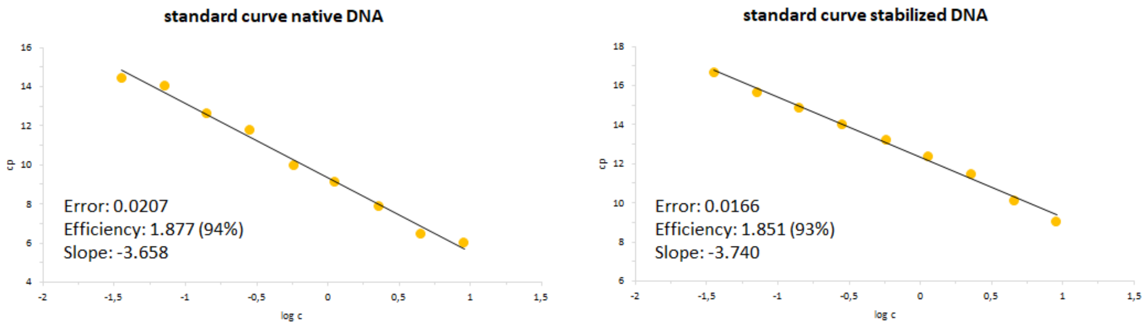

Figure S4 – Standard curve (qPCR) using different concentrations of DNA sequences containing native or stabilized DNA barcodes *Id* (5'-CT*C TC7De-dA 7De-dATT 7De-dAC7De-dA 7De-dACC T-3').

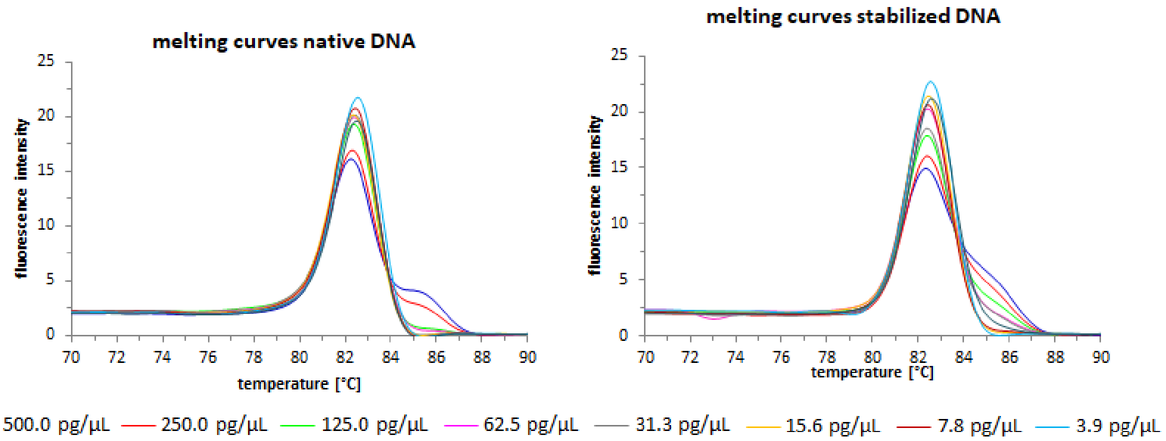

Figure S5 – Melting curves of the PCR products after qPCR of different concentrations of DNA sequences containing native or stabilized DNA barcodes *Id* (5'-CT*C TC7De-dA 7De-dATT 7De-dAC7De-dA 7De-dACC T-3').

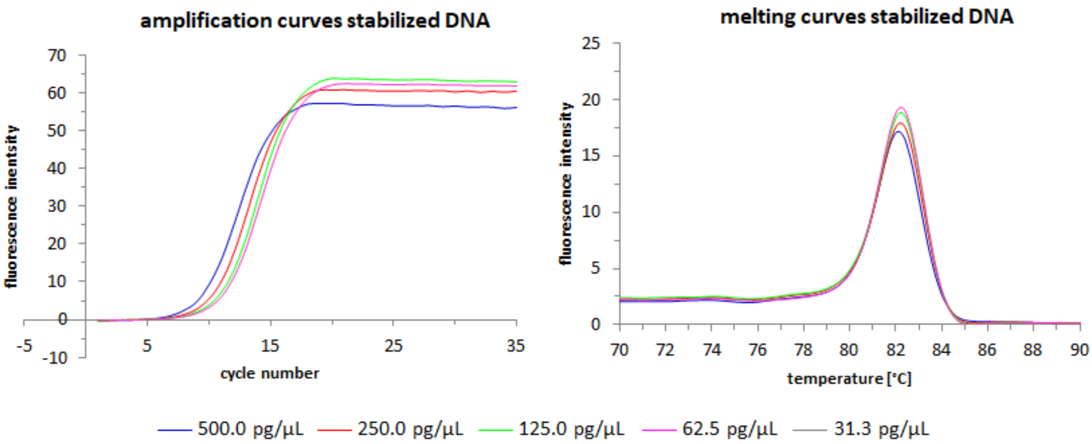

Figure S6 – Amplification and melting curves (qPCR) using different concentrations of DNA sequence containing stabilized DNA barcode *Ia* (5'-CT*C TCT 7De-dATC T7De-dAT 7De-dACC T-3').

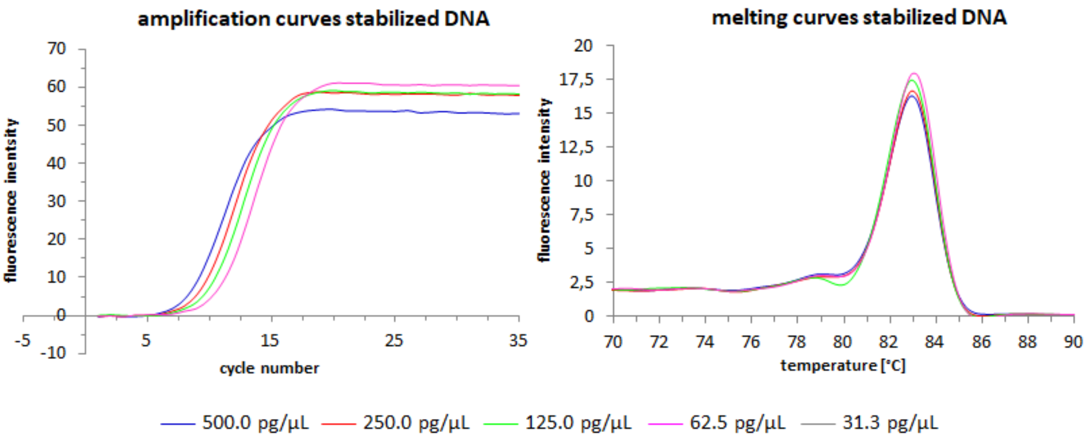

Figure S7 – Amplification and melting curves (qPCR) using different concentrations of DNA sequences containing stabilized DNA barcode *Ib* (5'-CT*C TTC 7De-dA 7De-dAT TCC 7De-dACC T-3').

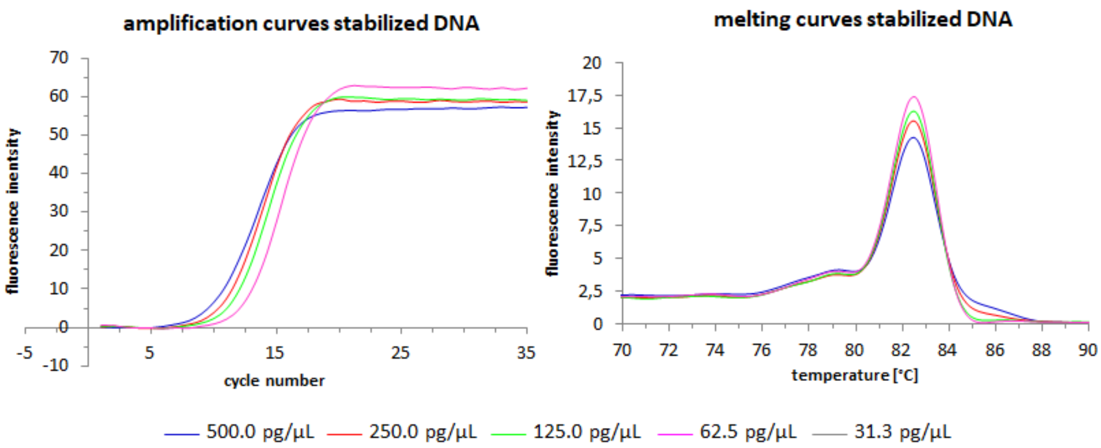

Figure S8 – Amplification and melting curves (qPCR) using different concentrations of DNA sequences containing stabilized DNA barcode Ic (5'-CT*C T7De-dAC 7De-dACT TT7De-dA 7De-dACC T-3').

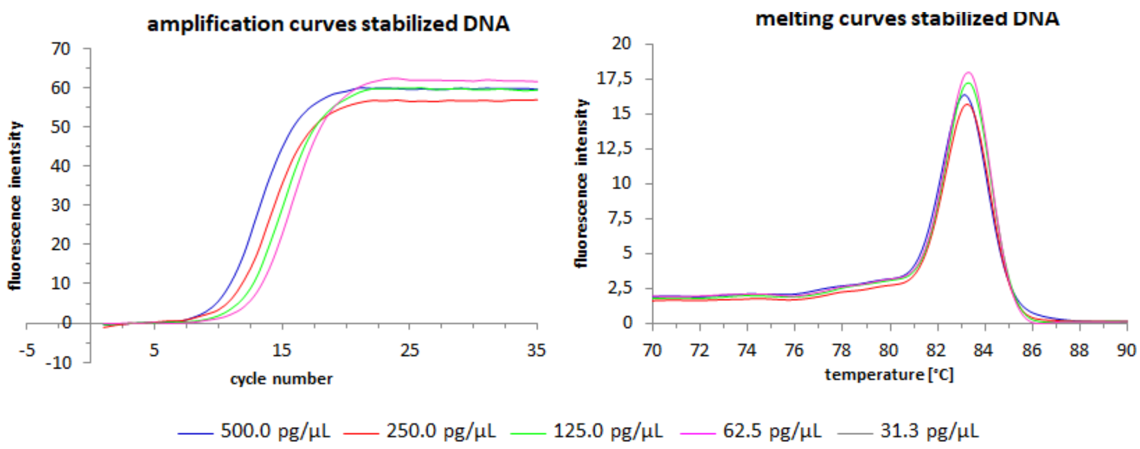

Figure S9 – Amplification and melting curves (qPCR) using different concentrations of DNA sequences containing stabilized DNA barcode Id (5'-CT*C T7De-dAC CT7De-dA CTT 7De-dACC T-3').

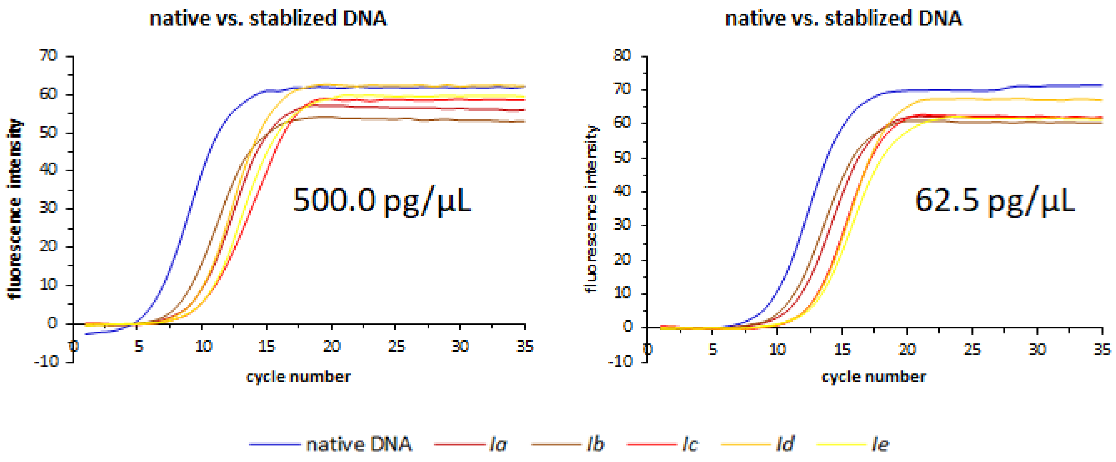

Figure S10 – Differences in the amplification rate of different DNA sequences containing stabilized DNA barcodes *(Ia-e)* and native DNA (*Ia*).

DNA-encoded chemistries

Representative Procedures

The syntheses followed published procedures.[26-32]

Copper(I)-promoted alkyne-azide cycloaddition (RP-03)

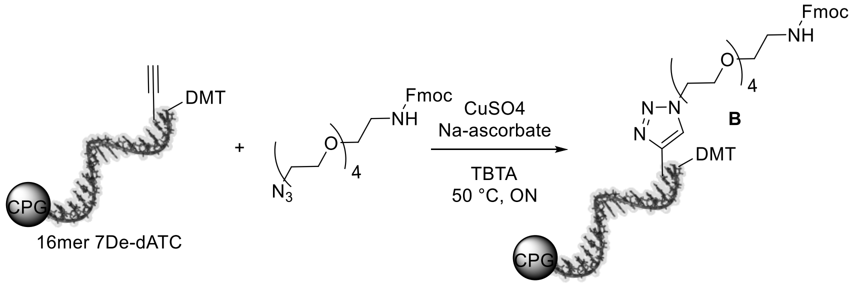

The CPG-bound oligonucleotide-alkyne conjugate (400 nmol) was suspended in 280 µL of H_2O/MeOH (1:1). Subsequently, the azide (33.60 µmol, 84 equiv.) dissolved in 100 µL of DMF, TBTA (16.80 µmol, 42 equiv.) dissolved in 120 µL of DMF, Na-ascorbate (16.80 µmol, 42 equiv.) dissolved in 10 µL of H_2O, and $CuSO_4{\cdot}5H_2O$ (1.68 µmol, 4.2 equiv.) dissolved in 10 µL of H_2O were added to the suspension in this order. Stock solutions of all reactants were prepared before the reaction was started. The reaction mixtures were shaken at 50 °C overnight. Then the CPG-bound conjugate was filtered over a filter column and washed three times with each 200 µL of 0.1 M EDTA solution, 0.1 M $MgCl_2$ solution, water, DMF, MeOH, ACN and CH_2Cl_2 and dried *in vacuo*.

The completeness of the reaction was controlled by cleavage of a small portion (~20 nmol) of CPG-bound oligonucleotide conjugate with 500 µL AMA (AMA = aqueous ammonia (30%)/ aqueous methylamine (40%), 1:1, vol/vol) for 4 h at ambient temperature. To this solution 20 µL of 1 M Tris buffer (pH = 7.5) were added, the mixture was dried in a SpeedVac, and dissolved in 200 µL of distilled water. The crude was analyzed by analytical RP-HPLC and MALDI-MS.

Amide coupling (RP-04)

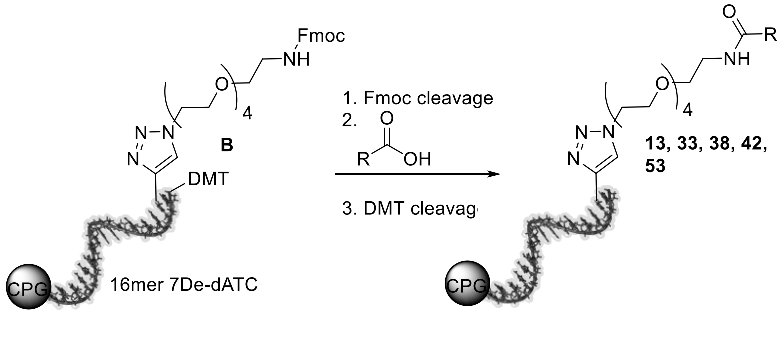

Step 1: The Fmoc-protecting group of the CPG-bound oligonucleotide (250 nmol, 9-10 mg) was cleaved off by addition of 200 µL 20% piperidine in dry DMF and shaking for 5 min. Afterwards, the CPG-bound deprotected oligonucleotide was washed three times with each 200 µL of DMF, MeOH, ACN and CH_2Cl_2 and then dried *in vacuo*.

Step 2: CPG-bound oligonucleotide, carboxylic acid and HATU were dried *in vacuo* for 15 min. Stock solutions of all reactants in dry DMF were prepared before the reaction was started. To the solution of carboxylic acid (25 µmol, 100 equiv.) in 75 µL dry DMF, HATU (25 µmol, 100 equiv.) dissolved in 75 µL dry DMF and DIPEA (62.5 µmol, 250 equiv.) were added. The mixture was shaken for 5 min and added to CPG-bound DNA suspended in 75 µL dry DMF (250 nmol, 1 equiv.). The amide coupling reaction was shaken at ambient temperature for 2 hours. Next, CPG-bound conjugate was filtered over a filter column, washed three times with each 200 µL of DMF, MeOH, ACN and CH_2Cl_2 and dried *in vacuo*. Amide coupling was repeated two times.

Completeness of amide coupling was controlled by cleaving off a small portion of CPG-bound oligonucleotide conjugate (0.7–0.9 mg, ~20 nmol) with 500 µL AMA (AMA = aqueous ammonia (30%)/ aqueous methylamine (40%), 1:1, vol/vol) 1 h (TC-sequences) or 4 h (ATGC- and 7De-dATC-sequences) at ambient temperature. Afterwards 20 µL of 1 M Tris buffer (pH = 7.5) were added, the mixture was dried under reduced pressure (SpeedVac) and DNA was dissolved in 200 µL distilled water. Crude reaction mixture was analyzed by analytical RP-HPLC and MALDI-MS. In case of uncompleted coupling (<90%) the reaction was repeated a third time.

Unreacted amines were capped with acetic acid anhydride (three times 200 µL, 30 s, 1:1 mixture of THF/methylimidazole, 9:1, vol/vol, and THF/pyridine/acetic acid anhydride 8:1:1, vol/vol). Capped CPG-bound oligonucleotide conjugate was washed three times with each 200 µL of DMF, MeOH, ACN and CH_2Cl_2 and dried *in vacuo*.

Step 3: DMT-protecting group of CPG-bound oligonucleotide (250 nmol, 9-10 mg of solid phase material) was removed by addition of 200 µL 3% trichloroacetic acid in CH_2Cl_2 for 1 min. Orange coloring of the solution indicated successful removal of protecting group. The deprotection was repeated 3-5 times until no further coloring of the solution was observed. CPG-bound deprotected DNA was washed three times with each 200 µL of 1% TEA in ACN, DMF, MeOH, ACN and CH_2Cl_2 and dried *in vacuo*.

Ugi four-component reaction on CPG-bound oligonucleotides (RP-05)

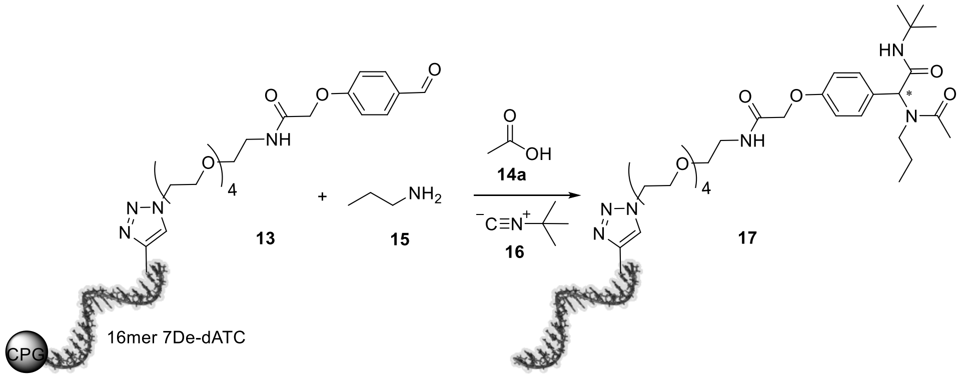

Prior to use, CPG-bound oligonucleotide aldehyde conjugate was dried *in vacuo* for 15 min.[26] A solution of propargylamine **15** (1000 equiv., 20 µmol) in 50 µL MeOH was added to the CPG-bound DNA-aldehyde conjugate **13**. The reaction mixture was shaken at ambient temperature for 3 h to effect imine formation. Afterwards, acetic acid **14a** (1000 equiv., 20 µmol, solid acids were dissolved in 15 µL MeOH) was pipetted to the reaction mixture, followed by the addition of *tert*-butylisocyanide **16** (1000 equiv., 20 µmol). The reaction mixture was shaken for 16 h at 50 °C. The CPG-bound conjugate was filtered over a filter column, washed three times with each 200 µL of DMF, MeOH, ACN and CH_2Cl_2 and dried *in vacuo*. The CPG-bound DNA conjugate was cleaved from the solid phase and deprotected with 500 µL AMA solution for 4 h at ambient temperature. Afterwards the mixture was dried in a SpeedVac and the remaining DNA pellet was dissolved in 200 µL of distilled water. The crude was analyzed by analytical RP-HPLC and MALDI-MS. The product was isolated by preparative RP-HPLC.

Ugi-azide four-component reaction on CPG-bound oligonucleotides (RP-06)

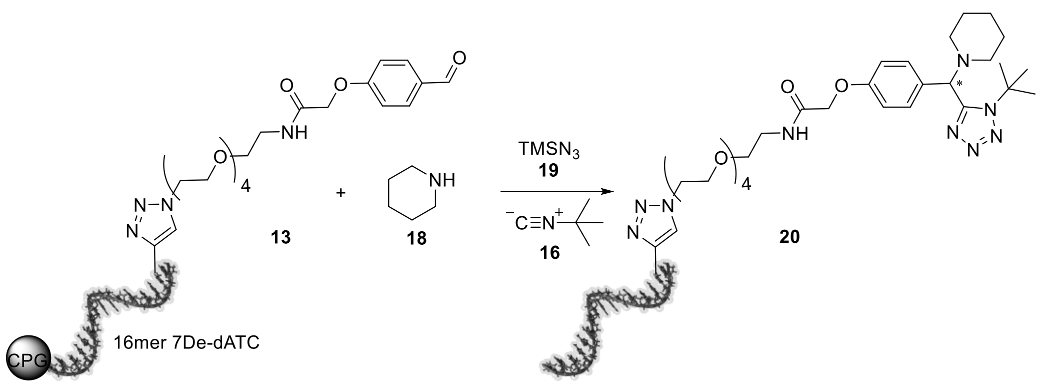

CPG-bound oligonucleotide was dried *in vacuo* for 15 min.[26] A solution of piperidine (1000 equiv., 20 µmol) in 50 µL MeOH was added to the CPG-bound DNA-aldehyde conjugate **13**. The reaction mixture was shaken at ambient temperature for 3 h to effect imine formation. Afterwards, *tert*-butylisocyanide **16** (1000 equiv., 20 µmol) was pipetted to the

reaction mixture, followed by the addition of azidotrimethylsilane (1000 equiv., 20 µmol). The reaction mixture was shaken for 16 h at 50 °C. The CPG-bound conjugate was filtered over a filter column, washed three times with each 200 µL of DMF, MeOH, ACN and CH_2Cl_2 and dried *in vacuo*. The CPG-bound DNA conjugate was cleaved from the solid phase and deprotected with 500 µL AMA solution for 4 h at ambient temperature. Afterwards the mixture was dried in a SpeedVac and the remaining DNA pellet was dissolved in 200 µL of distilled water. The crude was analyzed by analytical RP-HPLC and MALDI-MS. The product was isolated by preparative RP-HPLC.

Groebke-Blackburn-Bienaymé three-component reaction on CPG-bound oligonucleotides (RP-07)

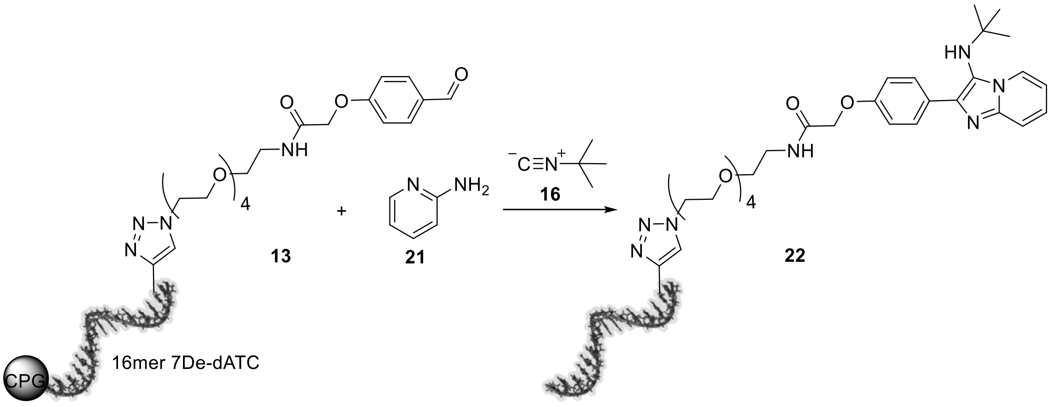

Prior to use, CPG-bound oligonucleotide aldehyde conjugate and 2-aminopyridine **21** were dried *in vacuo* for 15 min.[26] 2-aminopyridine (1000 equiv., 20 µmol) was added to the CPG-bound DNA-aldehyde conjugate **13** in 50 µL MeOH. The reaction mixture was shaken at ambient temperature for 6 h to effect imine formation. Afterwards, *tert*-butylisocyanide **16** (1000 equiv., 20 µmol) was pipetted to the reaction mixture, followed by the addition of acetic acid as Brønsted acid (final volume: 80 µL, acid concentration: 1%). The reaction mixture was shaken for 16 h at ambient temperature. The CPG-bound conjugate was filtered over a filter column, washed three times with each 200 µL of DMF, MeOH, ACN and CH_2Cl_2 and dried *in vacuo*. The CPG-bound DNA conjugate **22** was cleaved from the solid phase and deprotected with 500 µL AMA solution for 4 h at ambient temperature. Afterwards the mixture was dried in a SpeedVac and the remaining DNA pellet was dissolved in 200 µL of distilled water. The crude was analyzed by analytical RP-HPLC and MALDI-MS. The product was isolated by preparative RP-HPLC.

Ugi four-component/aza-Wittig reaction on CPG-bound oligonucleotides (RP-08)

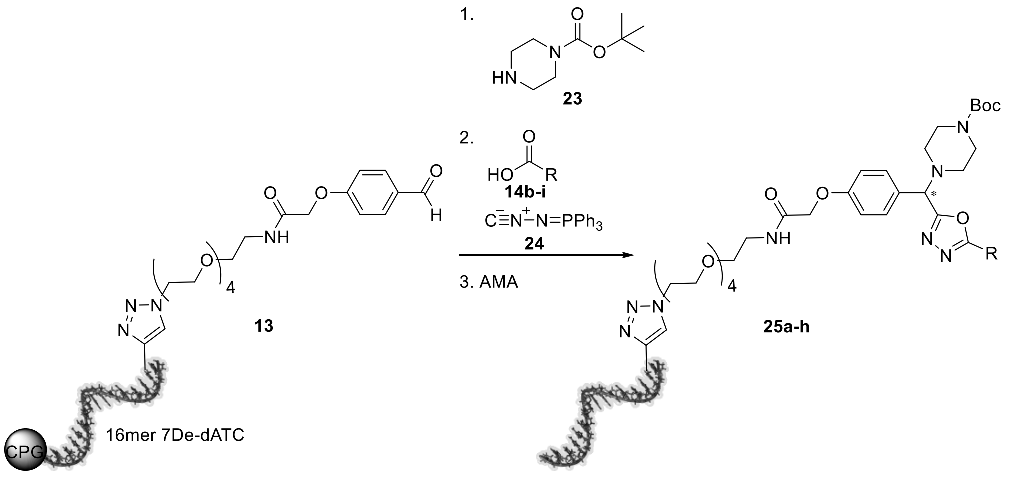

CPG-bound oligonucleotide, *N*-Boc-piperazine **23**, (isocyanoimino)triphenylphosphorane **24** and solid acids were dried *in vacuo* for 15 min.[26] *N*-Boc-piperazine **23** (1000 equiv., 20 µmol) was added to the CPG-bound DNA-aldehyde conjugate **13** in 30 µL 1,2-dichloroethane. The reaction mixture was shaken at ambient temperature for 3 h to effect imine formation. Then, the acid **14** (1000 equiv., 20 µmol) was dissolved in 80 µL 1,2-dichloroethane, transferred to (isocyanoimino)triphenylphosphorane **24** (1000 equiv., 20 µmol) and this mixture was added to the CPG-bound conjugate. The reaction mixture was shaken for 16 h at 50 °C. The CPG-bound conjugate was filtered over a filter column, washed three times with each 200 µL of DMF, MeOH, ACN and CH_2Cl_2 and dried *in vacuo*. The CPG-bound DNA conjugate **25** was cleaved from the solid phase and deprotected with 500 µL AMA solution for 4 h at ambient temperature. Afterwards the mixture was dried in a SpeedVac and the remaining DNA pellet was dissolved in 200 µL of distilled water. The crude was analyzed by analytical RP-HPLC and MALDI-MS. The product was isolated by preparative RP-HPLC.

(*R*)-(-)-BNDHP-mediated Biginelli reaction onCPG-bound oligonucleotides (RP-09)

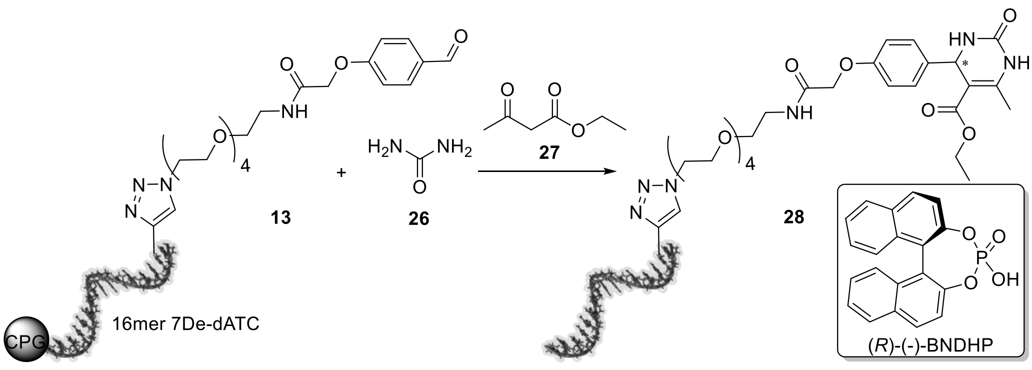

The CPG-bound oligonucleotide **13**, urea **26** and (*R*)-(-)-BNDHP were dried *in vacuo* for 15 min.[27] Urea **26** (10 µmol, 500 equiv.) and (*R*)-(-)-BNDHP (1 µmol, 50 equiv.) were

dissolved both in 30 µL ethanol. The solutions were added to CPG-coupled oligonucleotide-aldehyde conjugate **13** (20 nmol) followed by ethyl acetoacetate **27** (10 µmol, 500 equiv.). The reaction mixture was shaken at 50 °C for 20 h. Then the CPG-bound oligonucleotide conjugate **28** was filtered over a filter column, washed three times with each DMF, MeOH, ACN and CH_2Cl_2 and dried *in vacuo*. CPG-bound oligonucleotide conjugates **28** were cleaved from solid support and deprotected with 500 µL AMA at ambient temperature for 4 h. Afterwards 20 µL of 1 M Tris buffer (pH = 7.5) were added, the mixture was dried under reduced pressure (SpeedVac) and DNA was dissolved in 200 µL distilled water. The crude reaction mixture was analyzed by analytical RP-HPLC and MALDI-TOF-MS. The product was purified by preparative RP-HPLC.

(*R*)-(-)-BNDHP-mediated Povarov reaction on CPG-bound oligonucleotides (RP-10)

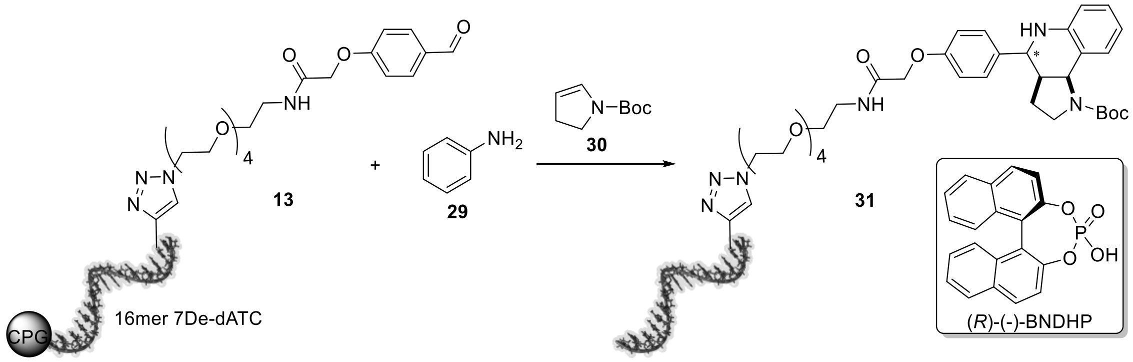

Prior to use, CPG-bound oligonucleotide, solid anilines and (*R*)-(-)-BNDHP were dried *in vacuo* for 15 min.[27] Aniline **29** (10 µmol, 500 equiv.) was dissolved in 24 µL ethanol. The solution was added to CPG-bound oligonucleotide-aldehyde conjugate **13** (20 nmol) suspended in 12 µL triethyl orthoformate. The suspension was shaken at ambient temperature for 4 h. Afterwards 30 µL of (*R*)-(-)-BNDHP (2 µmol, 100 equiv.) in ethanol followed by *N*-Boc-2,3-dihydro-1H-pyrrole **30** (10 µmol, 500 equiv.) was added. The reaction mixture was shaken at 50 °C for 16 h. Then the CPG-bound oligonucleotide conjugate was filtered over a filter column, washed three times with each DMF, MeOH, ACN and CH_2Cl_2 and dried *in vacuo*. CPG-bound oligonucleotide conjugates **31** were cleaved from solid support and deprotected with 500 µL AMA at ambient temperature for 4 h. Afterwards 20 µL of 1 M Tris buffer (pH = 7.5) were added, the mixture was dried under reduced pressure (SpeedVac) and DNA was dissolved in 200 µL distilled water. The crude reaction mixture was analyzed by analytical RP-HPLC and MALDI-TOF-MS. The product was purified by preparative RP-HPLC.

Trifluoroacetic acid-mediated Boc cleavage on CPG-bound oligonucleotides (RP-11)

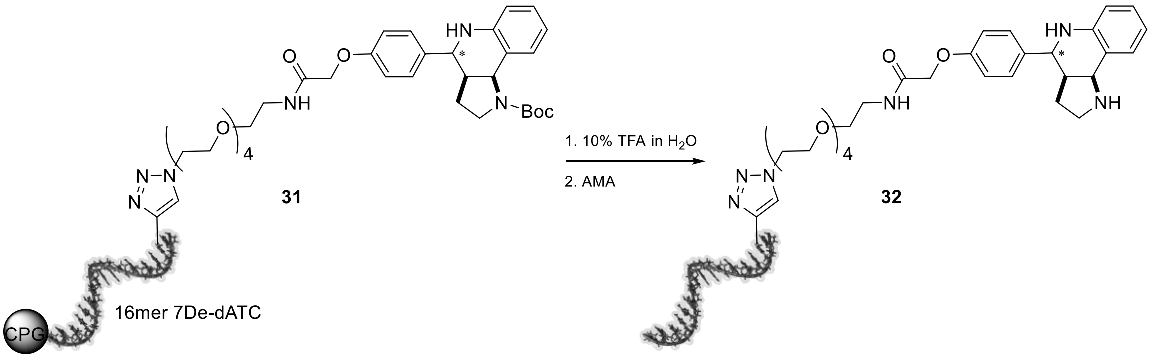

CPG-bound oligonucleotide conjugate **31** was treated with 10% trifluoroacetic acid in CH_2Cl_2 at ambient temperature for 4 h. Afterwards CPG bound DNA was filtered over a filter column, washed with excess of 1% TEA and three times with each 200 µL of DMF, MeOH, ACN and CH2Cl2 and dried in vacuo. CPG-bound oligonucleotide conjugates **32** were cleaved from solid support and deprotected with 500 µL AMA at ambient temperature for 4 h. Afterwards 20 µL of 1 M Tris buffer (pH = 7.5) were added, the mixture was dried under reduced pressure (SpeedVac) and DNA was dissolved in 200 µL distilled water. The crude reaction mixture was analyzed by analytical RP-HPLC and MALDI-TOF-MS. The product was purified by preparative RP-HPLC.

TFA-mediated Pictet-Spenger reaction on CPG-bound oligonucleotide-tryptophan conjugate 33 (RP-12)

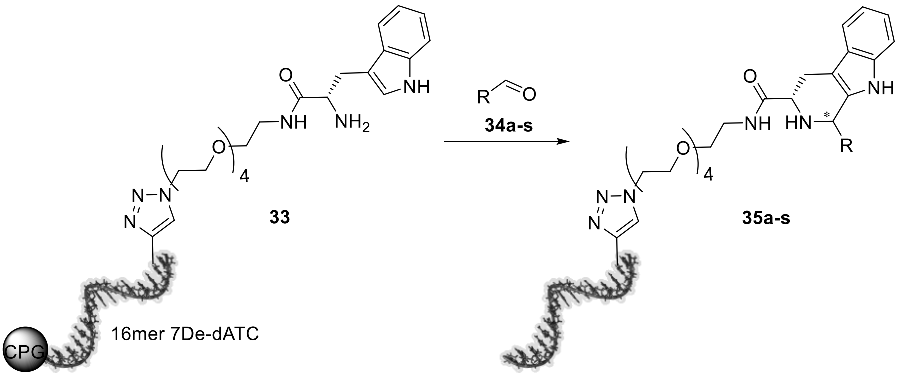

Prior to use, CPG-bound oligonucleotide **33** and the aldehyde **34** (for solids) were dried *in vacuo* for 15 min.[28,29] Aldehyde **34** (30 µmol, 1500 equiv.) was dissolved in 50 µL of a 5% trifluoroacetic acid in CH_2Cl_2 solution. This solution was added to CPG-bound oligonucleotide-tryptophan conjugate **33** (20 nmol) and the reaction mixture was shaken at ambient temperature for 20 h. Afterwards CPG bound DNA was filtered over a filter column, washed with excess of 1% TEA and three times with each 200 µL of DMF, MeOH, ACN and CH_2Cl_2 and dried in vacuo. CPG-bound oligonucleotide conjugates **35** were cleaved from solid support and deprotected with 500 µL AMA at ambient temperature for 4 h. Afterwards

20 µL of 1 M Tris buffer (pH = 7.5) were added, the mixture was dried under reduced pressure (SpeedVac) and DNA was dissolved in 200 µL distilled water. The crude reaction mixture was analyzed by analytical RP-HPLC and MALDI-TOF-MS. The product was purified by preparative RP-HPLC.

Zn(II)-mediated *aza*-Diels-Alder reaction on CPG-bound oligonucleotides 13 (RP-13)

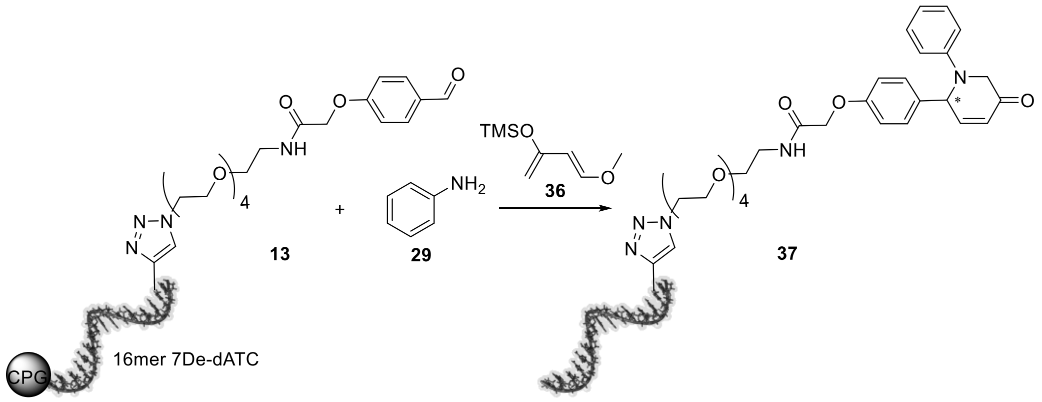

CPG-bound oligonucleotide **13**, and $ZnCl_2$ were dried *in vacuo* for 15 min.[27] Aniline **29** (10 µmol, 500 equiv.) was dissolved in 24 µL acetonitrile. The solution was added to CPG-coupled oligonucleotide-aldehyde conjugate **13** (20 nmol) suspended in 12 µL triethyl orthoformate. The suspension was shaken at ambient temperature for 4 h. Afterwards 30 µL of $ZnCl_2$ (2 µmol, 100 equiv.) in ACN followed by Danisheskys's diene **36** (20 µmol, 1000 equiv.) was added. The reaction mixture was shaken for 1 h at ambient temperature. Then the CPG-coupled oligonucleotide conjugate **37** was filtered over a filter column, washed three times with each 200 µL of 0.1 M EDTA solution, 0.1 M $MgCl_2$ solution, water, DMF, MeOH, ACN and CH_2Cl_2 and dried *in vacuo*. CPG-coupled oligonucleotide conjugate **37** was cleaved from solid support and deprotected with 200 µL aqueous ammonia (30%) at 50 °C for 6 h. Afterwards 20 µL of 1 M Tris buffer (pH = 7.5) were added, the mixture was dried under reduced pressure (SpeedVac) and DNA was dissolved in 200 µL distilled water. The crude reaction mixture was analyzed by analytical RP-HPLC and MALDI-TOF-MS. The product was purified by preparative RP-HPLC.

Cu(I)/bpy-mediated Petasis reaction on CPG-bound oligonucleotides 38 (RP-14)

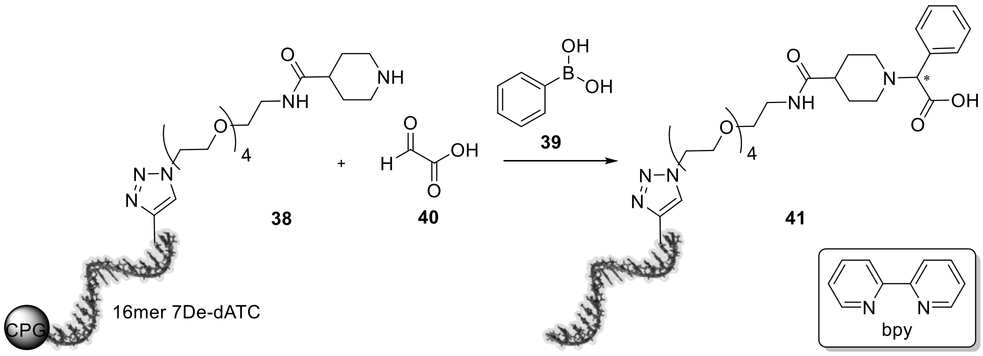

Prior to use all solid materials were dried in vacuo for 30 min.[30] CuCl (4.0 µmol, 200 equiv., 40 mM calculated for the final volume of 100 µL) and 2,2′-bipyridine (bpy, 4.0 µmol, 200 equiv., 40 mM calculated for the final volume of 100 µL) were dissolved in 48 µL DMF. The solution was shaken at 50 °C for 1 h. Phenylboronic acid **39** (50 µmol, 2500 equiv., 500 mM calculated for the final volume of 100 µL) were dissolved in the CuCl/bpy solution in DMF. 12 µL triethyl orthoformate and glyoxylic acid **40** (40 µmol, 2000 equiv., 400 mM calculated for the final volume of 100 µL) dissolved in 40 µL DMF were added. The solution was added to CPG-coupled-DNA-secondary amine conjugate **38** (20 nmol, 1 equiv.) and the suspension was shaken at 50 °C for 20 h. Then the CPG-bound DNA conjugate was filtered over a filter column, washed three times with each 200 µL of 0.1 M EDTA solution, 0.1 M $MgCl_2$ solution, water, DMF, MeOH, ACN and CH_2Cl_2 and dried in vacuo. CPG-bound oligonucleotide conjugated α-aryl glycine **41** were cleaved from solid support and deprotected with 500 µL AMA for 4 h at ambient temperature. Afterwards 20 µL of 1 M Tris buffer (pH = 7.5) were added, the mixture was dried under reduced pressure (SpeedVac) and the DNA was *dissolved in 200 µL distilled water. The crude reaction mixture was analyzed by analytical* RP-HPLC and MALDI-TOF-MS. The product was purified by preparative RP-HPLC.

Ag(I)-mediated 1,3-dipolar cycloaddition reaction on CPG-bound oligonucleotides 42 (RP-15)

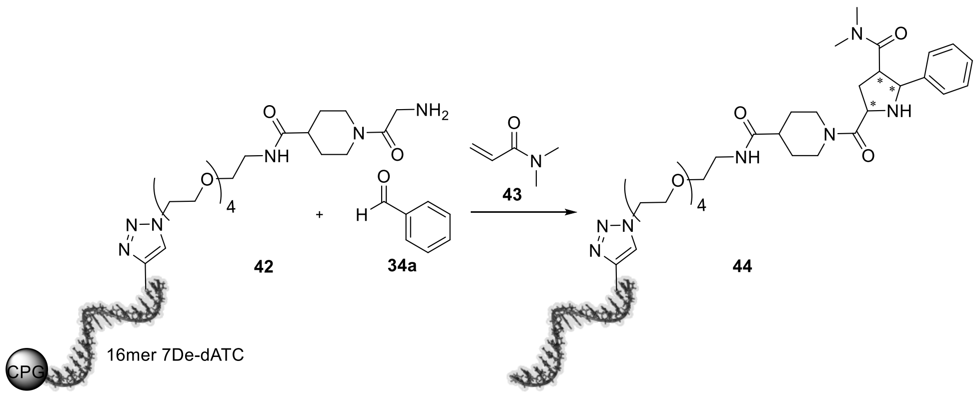

CPG-bound oligonucleotide **13** was dried *in vacuo* for 15 min.[28] Benzaldehyde **34a** (1000 equiv., 20 µmol) was added to the CPG-bound DNA-glycine conjugate **42** in 50 µL ACN/triethyl orthoformate (2:1). The reaction mixture was shaken at ambient temperature for 6 h. Afterwards, 30 µL of a suspension of AgOAc (100 equiv., 2 µmol) in ACN/triethyl orthoformate (2:1) was added followed by *N*,*N*-dimethylacrylamide **43** (4000 equiv., 80 µmol) and TEA (4000 equiv., 80 µmol). Prior addition to the reaction vessel, the AgOAc suspension was vortexed and pipetted up and down to obtain a homogeneous suspension. The reaction mixture was shaken for 16 h at 50 °C. Afterwards CPG bound DNA was filtered over a filter column, washed with excess of 1% TEA and three times with each 200 µL of 0.1 M EDTA solution, 0.1 M $MgCl_2$ solution, water, DMF, MeOH, ACN and CH_2Cl_2 and dried in vacuo. CPG-bound oligonucleotide conjugates **44** were cleaved from solid support and deprotected with 500 µL AMA at ambient temperature for 4 h. Afterwards 20 µL of 1 M Tris buffer (pH = 7.5) were added, the mixture was dried under reduced pressure (SpeedVac) and DNA was dissolved in 45 µL distilled water. 5 µL of 1,3,5-triazine-2,4,6-trithiol trisodium (TMT) salt solution (15% in H_2O) were added and the solution was shaken for 30 min at ambient temperature. Afterwards the sample was centrifuged at 4 °C for 30 min (13200 rpm; Centrifuge 5415 R, *Eppendorf*), the supernatant was taken off and diluted with 5 µL of a 3 M sodium acetate (pH = 5.2) and 200 µL 100% ethanol. The solution was incubated overnight at -80 °C. Afterwards the samples were centrifuged at 4 °C for 30 min (13200 rpm; Centrifuge 5415R, *Eppendorf*), the supernatant was taken off, additional 100 µL of 100% ethanol were added to the pellet and the solution was incubated again for 1 h at -80 °C. Afterwards the sample was centrifuged at 4 °C for 30 min (13200 rpm; Centrifuge 5415R, *Eppendorf*), the supernatant was taken off, and the DNA pellets were dried at 37 °C. The DNA samples were dissolved in 100 µL ddH_2O. The crude was analyzed by analytical RP-HPLC and MALDI-MS. The product was purified by preparative RP-HPLC.

Yb(III)-mediated Castagnoli-Cushman reaction on CPG-bound oligonucleotides 13 (RP-16)

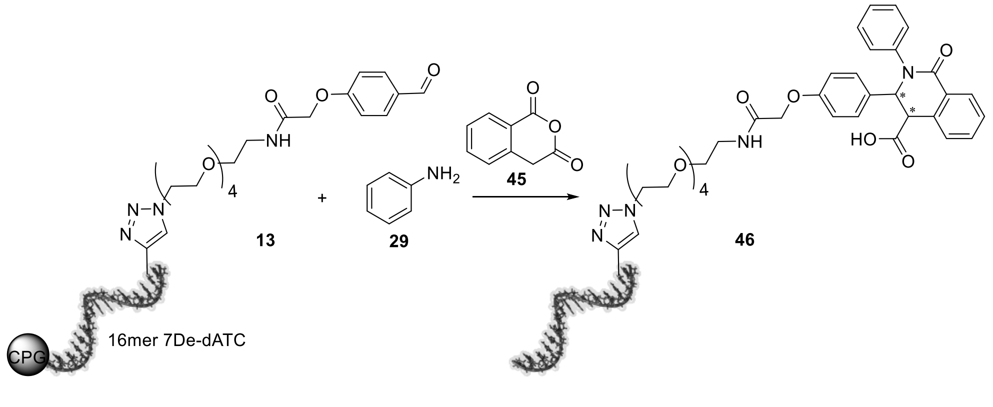

CPG-bound oligonucleotide **13**, homophtalic anhydride **44** and $Yb(OTf)_3$ were dried *in vacuo* for 15 min. [28] Aniline **29** (10 µmol, 500 equiv.) was dissolved in 24 µL CH_2Cl_2. The solution was added to the CPG-bound oligonucleotide-aldehyde conjugate **13** (20 nmol) suspended in 12 µL triethyl orthoformate. The suspension was shaken at ambient temperature for 4 h. Afterwards 30 µL of a suspension of $Yb(OTf)_3$ (1 µmol, 50 equiv.) in CH_2Cl_2 was added, followed by 30 µL of a suspension of homophtalic anhydride **45** (10 µmol, 500 equiv.) in CH_2Cl_2. Prior addition to the reaction vessel both suspensions were vortexed and pipetted up and down to obtain homogeneous suspensions. The reaction mixture was shaken for 1 h at ambient temperature. Then the CPG-bound conjugate was filtered over a filter column and washed three times with each 200 µL of 0.1 M EDTA solution, 0.1 M $MgCl_2$ solution, water, DMF, MeOH, ACN and CH_2Cl_2 and dried in *vacuo*. CPG-bound oligonucleotide conjugate **46** was then cleaved from the solid support and deprotected with 500 µL AMA solution for 4 h at ambient temperature. To this solution 20 µL of 1 M Tris buffer (pH = 7.5) were added, the mixture was dried in a SpeedVac and afterwards dissolved in 200 µL of distilled water. The crude was analyzed by analytical RP-HPLC and MALDI-MS. The product was purified by preparative RP-HPLC.

$Yb(PFO)_3$-mediated three-component synthesis of pyrazoles on CPG-bound oligo-nucleotides 13 (RP-17)

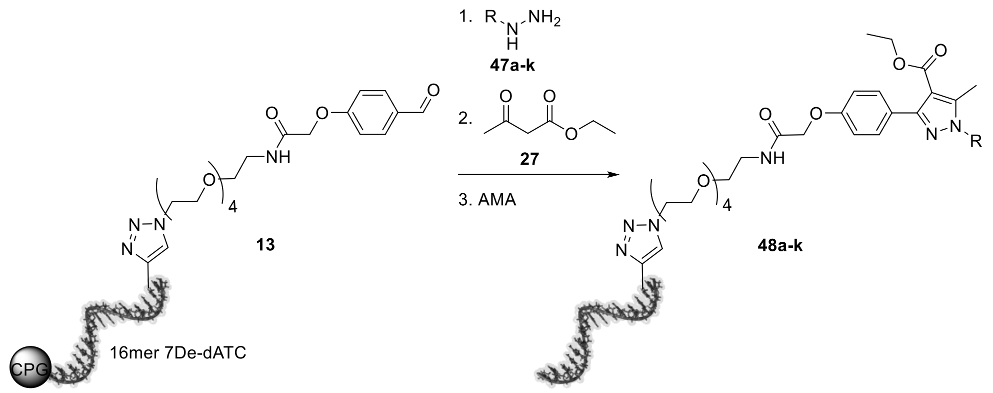

The catalyst $Yb(PFO)_3$ was prepared according to a published procedure.[31] Prior to the reaction, the hydrazine was extracted with diluted NH_3 solution and CH_2Cl_2, dried over $MgSO_4$ and finally dried *in vacuo* if the hydrazine was present as a hydrochloride salt. The hydrazine **47** (250 equiv., 5 µmol), dissolved in 30 µL toluene was added to the CPG-bound DNA-aldehyde conjugate **13** and the reaction mixture was shaken at ambient temperature for 0.5 h. Afterwards, ethyl acetoacetate **27** (3000 equiv., 60 µmol) and 50 µL of a suspension of $Yb(PFO)_3$ (250 equiv., 5 µmol) in toluene was added. Prior addition to the reaction vessel the $Yb(PFO)_3$ suspension was vortexed and pipetted up and down to obtain a homogeneous suspension. The reaction mixture was shaken at 50 °C for 16 h. The CPG-bound conjugate

was filtered over a filter column and washed with each 3x 200 µL of 0.1 M EDTA solution, 0.1 M $MgCl_2$ solution, water, DMF, MeOH, ACN and CH_2Cl_2 and then dried *in vacuo*. The CPG-bound DNA conjugate **48** was cleaved from the solid phase and deprotected by adding 500 µL AMA solution and shaking for 1 h (TC-sequences) or 4 hours (ATCG- and 7De-dATC-sequences) at ambient temperature. Afterwards the mixture was dried in a SpeedVac and the remaining DNA pellet was dissolved in 200 µL of distilled water.

Au(I)/Ag(I)-promoted pyrazoline-containing spiroheterocycle synthesis on CPG-bound oligonucleotides 13 (RP-18)

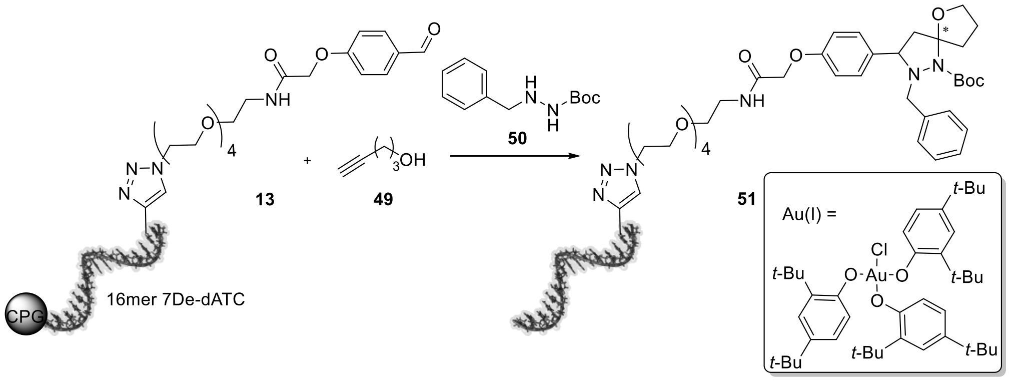

CPG-bound oligonucleotide **13**, *tert*-butyl 2-benzylhydrazinecarboxylate **50**, [Tris(2,4-di-*tert*-butylphenyl)phosphite]gold chloride and $AgSbF_6$ were dried *in vacuo* for 15 min.[32] The solution of *tert*-butyl 2-benzylhydrazine-carboxylate **50** (500 equiv., 15 µmol) in 20 µL THF and pent-4-yn-1-ol **49** (1000 equiv., 30 µmol) were added to CPG- bound DNA-aldehyde conjugate **13** (30 nmol) followed by equimolar mixture of Au(I)/$AgSbF_6$ (250 equiv., 7.5 µmol) suspended in 30 µL THF. Prior addition to the reaction vessel the mixture was vortexed and pipetted up and down. The reaction mixture was shaken at room temperature for 20 h. Then the CPG-bound conjugate was filtered over a filter column and washed three times with each 200 µL of 0.1 M EDTA solution, 0.1 M $MgCl_2$ solution, water, DMF, MeOH, ACN and CH_2Cl_2 and dried in *vacuo*. CPG-bound oligonucleotide conjugate **51** was then cleaved from the solid support and deprotected with 500 µL AMA solution for 4 h at ambient temperature. To this solution 20 µL of 1 M Tris buffer (pH = 7.5) were added, the mixture was dried in a SpeedVac and afterwards dissolved in 45 µL of distilled water. 5 µL of 1,3,5-triazine-2,4,6-trithiol trisodium salt solution (15% in H_2O) were added and the solution was shaken for 30 min at ambient temperature. Afterwards the sample was centrifuged at 4 °C for 30 min (13200 rpm; Centrifuge 5415 R, *Eppendorf*), the supernatant was taken off and diluted with 5 µL of a 3 M sodium acetate (pH = 5.2) and 200 µL 100% ethanol. The solution was incubated overnight at -80 °C. Afterwards the samples were centrifuged at 4 °C for 30 min (13200 rpm; Centrifuge 5415 R, *Eppendorf*), the supernatant was taken off, additional 100 µL of 100% ethanol were added to the pellet and the solution was incubated again for 1 h at -80

°C. Afterwards the sample was centrifuged at 4 °C for 30 min (13200 rpm; Centrifuge 5415 R, *Eppendorf*), the supernatant was taken off, and the DNA pellets were dried at 37 °C. The DNA samples were dissolved in 100 µL ddH_2O. The crude was analyzed by analytical RP-HPLC and MALDI-MS. The product was purified by preparative RP-HPLC.

Au(I)/Ag(I)-promoted pyrazoline synthesis on CPG-bound oligonucleotides 53 (RP-19)

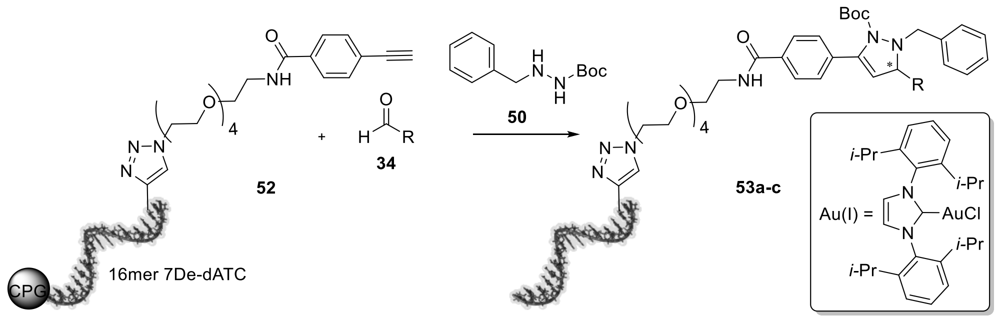

CPG-bound oligonucleotide **52**, *tert*-butyl 2-benzylhydrazinecarboxylate **50**, chloro[1,3-bis(2,6-diisopropylphenyl)imidazol-2-ylidene] gold(I) and AgOTf were dried *in vacuo* for 15 min.[29] The solution of *tert*-butyl 2-benzylhydrazine-carboxylate **50** (1000 equiv., 20 µmol) in 20 µL dry acetonitrile and aliphatic aldehyde **34** (1000 equiv., 20 µmol) were added to CPG- bound DNA-alkyne conjugate **52** (20 nmol) followed by equimolar mixture of Au(I)/AgOTf (250 equiv., 5 µmol) suspended in dry 30 µL acetonitrile. Prior addition to the reaction vessel the mixture was vortexed and pipetted up and down. The reaction mixture was shaken at room temperature for 20 h. Then the CPG-bound conjugate was filtered over a filter column and washed three times with each 200 µL of 0.1 M EDTA solution, 0.1 M $MgCl_2$ solution, water, DMF, MeOH, ACN and CH_2Cl_2 and dried *in vacuo*. CPG-bound oligonucleotide conjugate **53** was then cleaved from the solid support and deprotected with 500 µL AMA solution for 4 h at 50 °C. To this solution 20 µL of 1 M Tris buffer (pH = 7.5) were added, the mixture was dried in a SpeedVac and afterwards dissolved in 45 µL of distilled water. 5 µL of 1,3,5-triazine-2,4,6-trithiol trisodium salt solution (15% in H_2O) were added and the solution was shaken for 30 min at ambient temperature. Afterwards the sample was centrifuged at 4 °C for 30 min (13200 rpm; Centrifuge 5415 R, *Eppendorf*), the supernatant was taken off and diluted with 5 µL of a 3 M sodium acetate (pH = 5.2) and 200 µL 100% ethanol. The solution was incubated overnight at -80 °C. Afterwards the samples were centrifuged at 4 °C for 30 min (13200 rpm; Centrifuge 5415 R, *Eppendorf*), the supernatant was taken off, additional 100 µL of 100% ethanol were added to the pellet and the solution was incubated again for 1 h at -80 °C. Afterwards the sample was centrifuged at 4 °C for 30 min (13200 rpm; Centrifuge 5415 R, Eppendorf), the supernatant was taken off, and the DNA pellets were dried at 37 °C. The DNA samples were dissolved in 100 µL ddH_2O. The crude was analyzed by analytical RP-HPLC and MALDI-MS. The product was

purified by preparative RP-HPLC. *For **aromatic aldehydes 34** glacial acetic acid was used instead of acetonitrile as solvent.*

Au(I)/Ag(I)-promoted pyrazole synthesis on CPG-bound oligonucleotides 53 (RP-20)

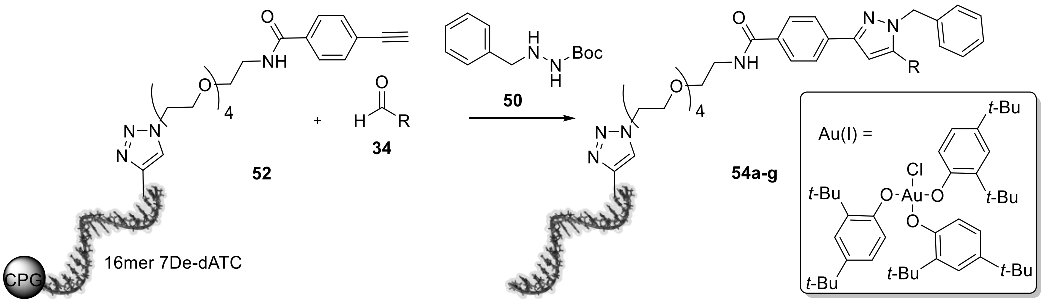

CPG-bound oligonucleotide **52**, *tert*-butyl 2-benzylhydrazinecarboxylate **50**, [tris(2,4-di-tert-butylphenyl)phosphite]gold chloride and AgOTf were dried *in vacuo* for 15 min.[29] The solution of *tert*-butyl 2-benzylhydrazine-carboxylate **51** (1000 equiv., 20 µmol) in 20 µL glacial acetic acid and aldehyde **34** (1000 equiv., 20 µmol) were added to CPG- bound DNA-alkyne conjugate **52** (20 nmol) followed by equimolar mixture of Au(I)/AgOTf (250 equiv., 5 µmol) suspended in dry 30 µL glacial acetic acid. Prior addition to the reaction vessel the mixture was vortexed and pipetted up and down. The reaction mixture was shaken at 60 °C for 20 h. Then the CPG-bound conjugate was filtered over a filter column and washed three times with each 200 µL of 0.1 M EDTA solution, 0.1 M $MgCl_2$ solution, water, DMF, MeOH, ACN and CH_2Cl_2 and dried in *vacuo*. CPG-bound oligonucleotide conjugate **54** was then cleaved from the solid support and deprotected with 500 µL AMA solution for 4 h at ambient temperature. To this solution 20 µL of 1 M Tris buffer (pH = 7.5) were added, the mixture was dried in a SpeedVac and afterwards dissolved in 45 µL of distilled water. 5 µL of 1,3,5-triazine-2,4,6-trithiol trisodium salt solution (15% in H_2O) were added and the solution was shaken for 30 min at ambient temperature. Afterwards the sample was centrifuged at 4 °C for 30 min (13200 rpm; Centrifuge 5415 R, *Eppendorf*), the supernatant was taken off and diluted with 5 µL of a 3 M sodium acetate (pH = 5.2) and 200 µL 100% ethanol. The solution was incubated overnight at -80 °C. Afterwards the samples were centrifuged at 4 °C for 30 min (13200 rpm; Centrifuge 5415 R, *Eppendorf*), the supernatant was taken off, additional 100 µL of 100% ethanol were added to the pellet and the solution was incubated again for 1 h at -80 °C. Afterwards the sample was centrifuged at 4 °C for 30 min (13200 rpm; Centrifuge 5415 R, *Eppendorf*), the supernatant was taken off, and the DNA pellets were dried at 37 °C. The DNA samples were dissolved in 100 µL ddH_2O. The crude was analyzed by analytical RP-HPLC and MALDI-MS. The product was purified by preparative RP-HPLC.

Trifluoroacetic acid-mediated Boc cleavage on oligonucleotide scaffold conjugates in solution (RP-21)

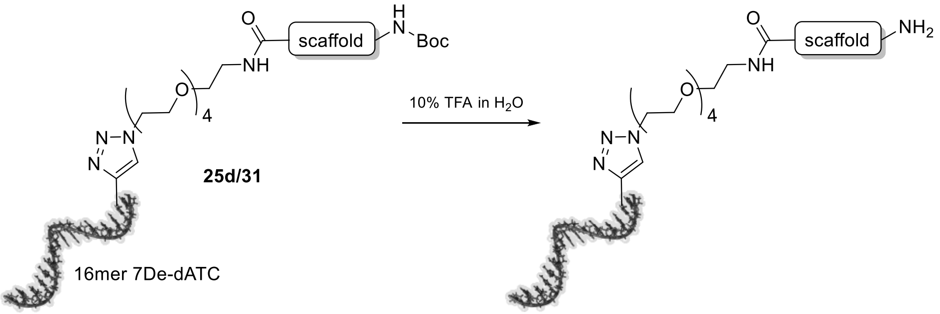

An isolated pellet of Boc-protected oligonucleotide conjugate **25d** or **31** was dissolved in 20 µL of a 10% trifluoroacetic acid in H_2O solution. The solution was shaken at ambient temperature for 4 h. The Boc deprotected oligonucleotide conjugate was precipitated by adding 2 µL of a 3 M sodium acetate (pH = 5.2) and 80 µL of 100% ethanol and storing this solution for overnight at -80 °C. Afterwards, the samples were centrifuged at 4 °C for 30 min (13200 rpm; Centrifuge 5415 R, *Eppendorf*), the supernatant was taken off and the DNA pellets were dried. Oligonucleotide conjugate was dissolved in ddH_2O and analyzed by analytical RP-HPLC and MALDI-TOF-MS

Table S8 – Overview of diverse chemical reactions on CPG-bound stabilized barcode.

Entry	Reaction	Conditions
1	Ugi four-component reaction (**RP-05**)[26] 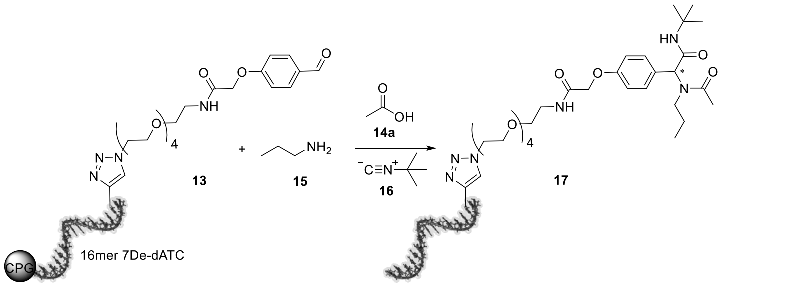	1. MeOH, 50 °C<br>2. AMA, rt
2	Ugi-azide four-component reaction (**RP-06**)[26] 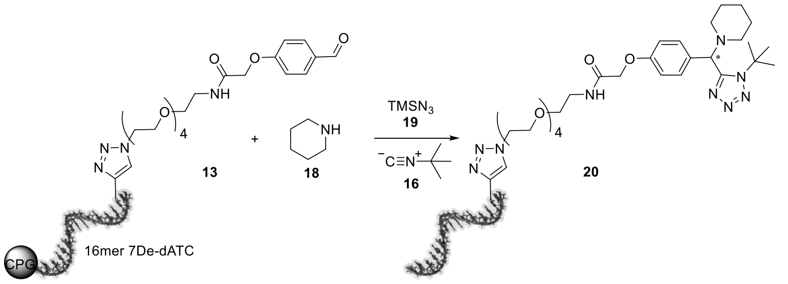	1. MeOH, 50 °C<br>2. AMA, rt
3	Groebke-Blackburn-Bienaymé reaction (**RP-07**)[26] 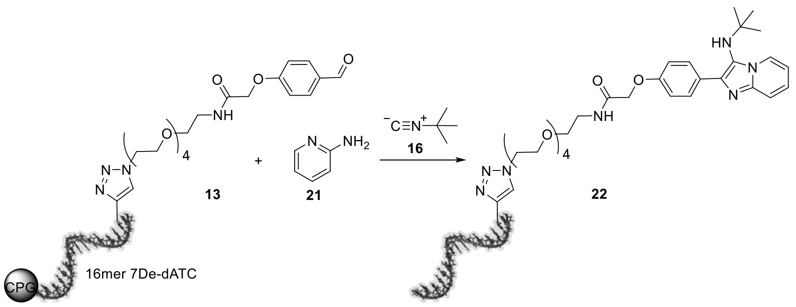	1. 1% acetic acid in MeOH, rt<br>2. AMA, rt
4	Ugi/*aza*-Wittig reaction (**RP-08**)[26] 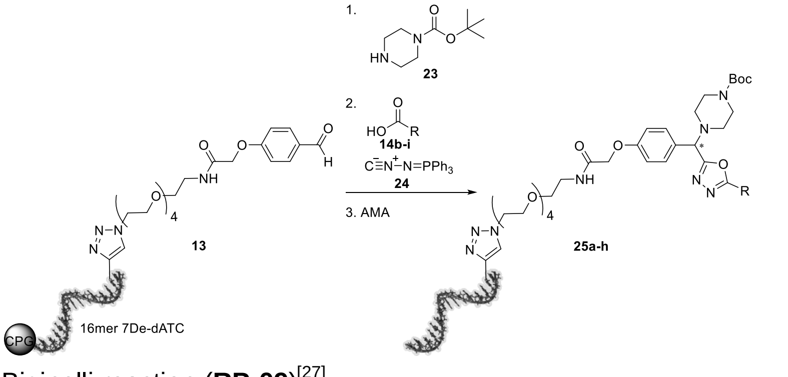	1. MeOH, 50 °C<br>2. AMA, rt
5	Biginelli reaction (**RP-09**)[27] 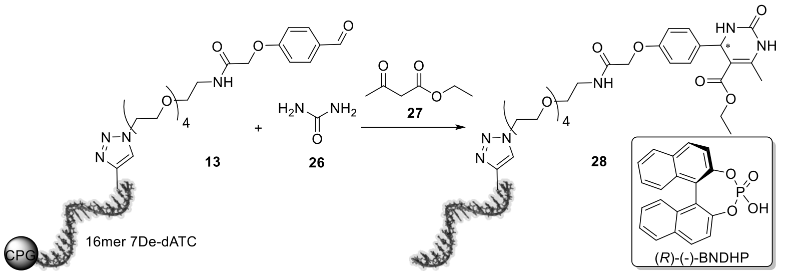	1. (*R*)-(-)-BNDHP EtOH 50 °C<br>2. AMA, rt

6 Povarov reaction (**RP-10**)[27]

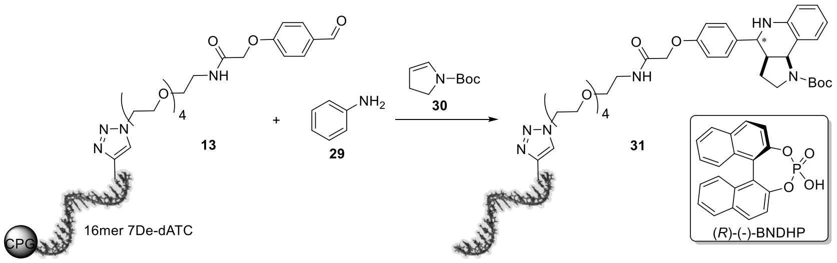

1. (*R*)-(-)-BNDHP
   EtOH/TEOF, 50 °C
2. AMA, rt

7 Boc cleavage (**RP-11**)

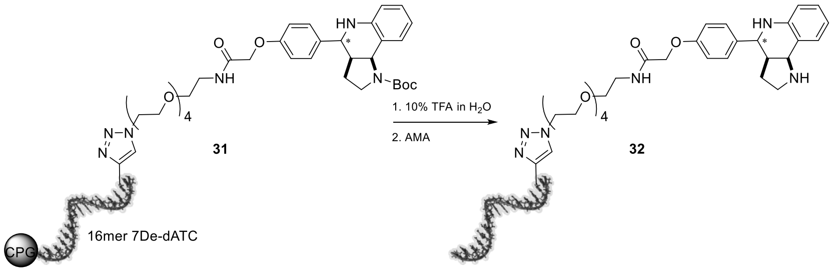

1. 10% TFA in H_2O
   4 h, rt
2. AMA, rt

8 Pictet-Spengler reaction (**RP-12**)[28,29]

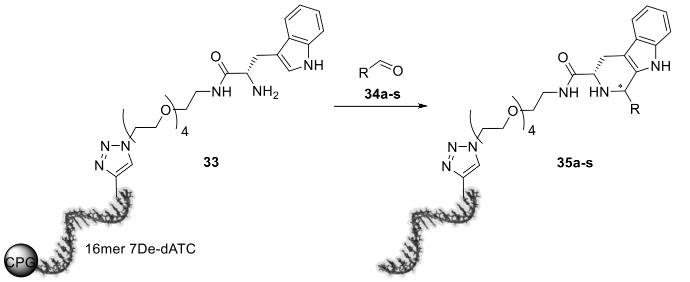

1. TFA
   CH_2Cl_2, rt
2. AMA, rt

9 aza-Diels-Alder reaction (**RP-13**)[27]

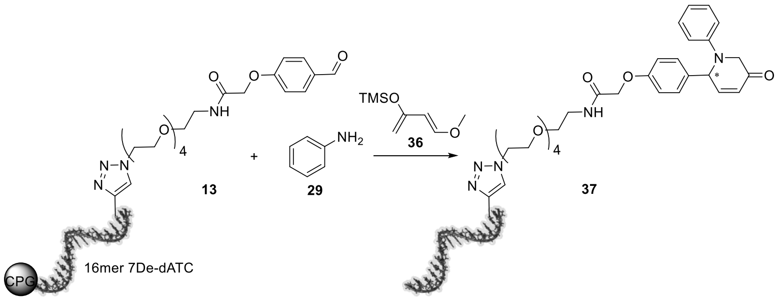

1. $ZnCl_2$
   ACN/TEOF, rt
2. aq. NH_3, 50 °C

10 Petasis reaction (**RP-14**)[30]

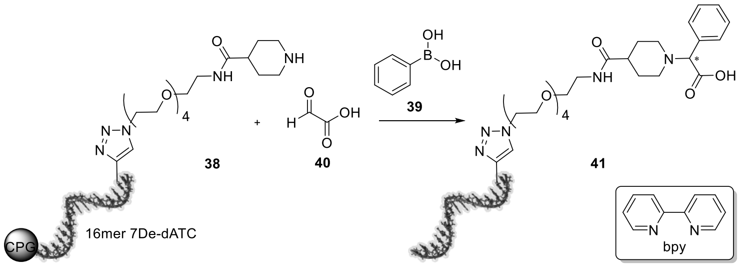

1. CuCl/bpy
   DMF/TEOF, 50 °C
2. AMA, rt

11 1,3-Dipolar cycloaddition (**RP-15**)[28]

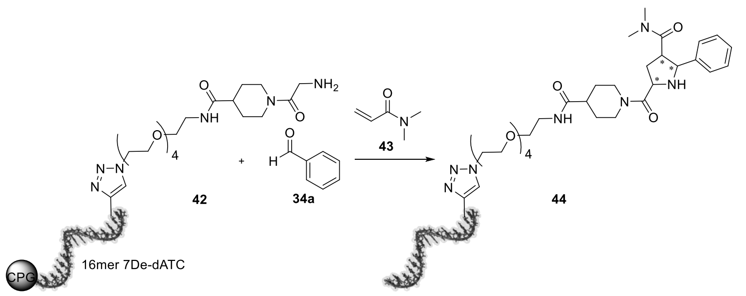

1. AgOAc
   ACN/TEOF, 50 °C
2. AMA, rt

12 Castagnoli-Cushman reaction (**RP-16**)[28]

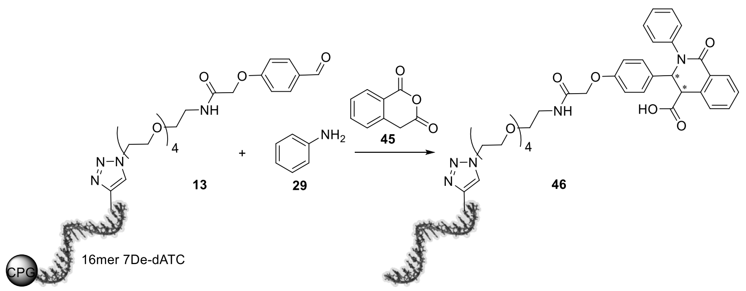

1. $Yb(OTf)_3$
   CH_2Cl_2/TEOF,
   rt
2. AMA, rt

13 Three-component pyrazole synthesis (**RP-17**)

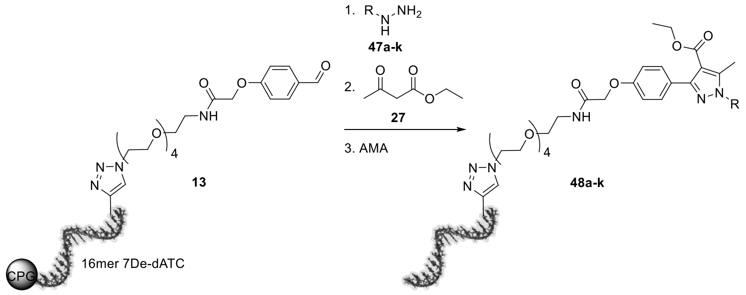

1. $Yb(PFO)_3$
   toluene, 50 °C
2. AMA, rt

14 Pyrazoline-containing spiroheterocycle synthesis (**RP-18**)[32]

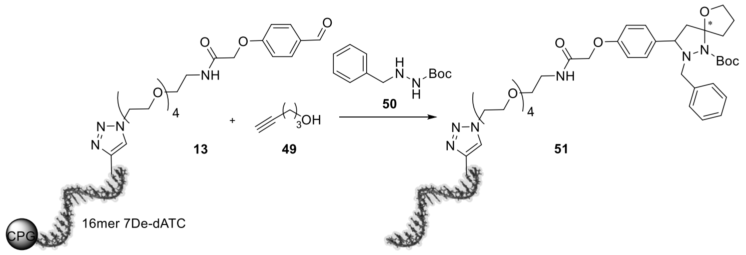

1. Au(I)/Ag(I)
   THF, rt
2. AMA, rt

15 Pyrazoline synthesis (**RP-19**)[29]

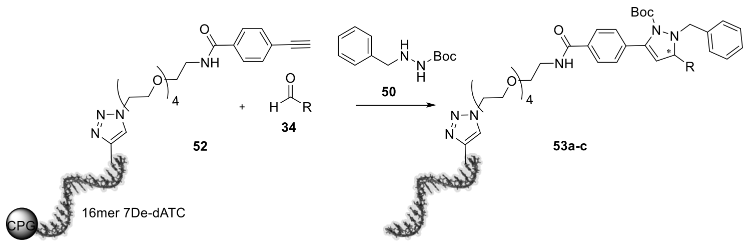

1. Au(I)/Ag(I)
   MeCN, 50 °C
2. AMA, rt

R = aliphatic

16 Pyrazole synthesis (**RP-20**)[29]

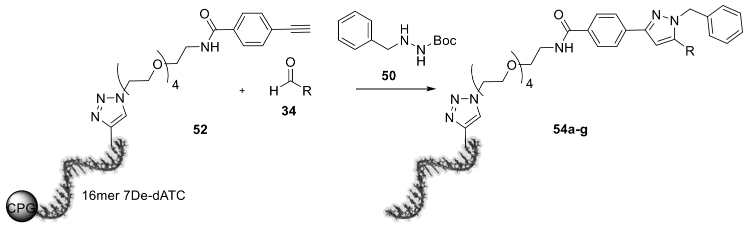

1. Au(I)/Ag(I)
   AcOH, 60 °C
2. AMA, rt

R = aromatic, aliphatic

HPLC traces and MALDI-MS spectrum

CPG-bound DNA-starting material conjugates

CPG-bound 16mer 7De-dATC-alkyne conjugate was reacted with Boc-*N*-amido-PEG(4)-azide according to RP-03.

HPLC trace of crude reaction mixture (analytical RP-HPLC)

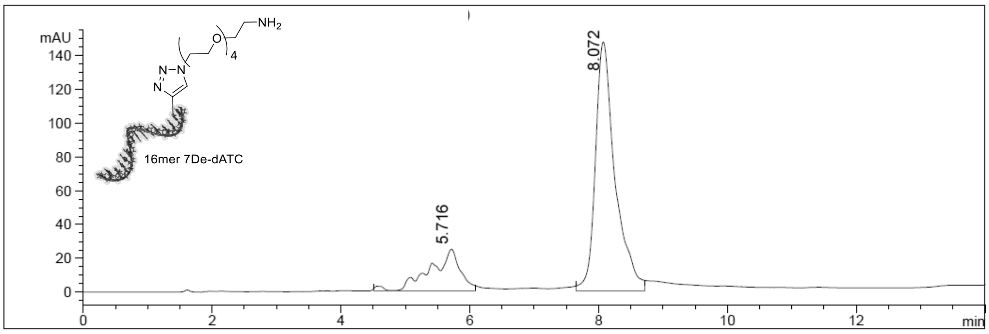

Peak list:

Ret. Time	Width min	Height	Area	Area %
5.716	0.534	24.523	786.225	20.195
8.072	0.352	147.020	3106.938	79.805

MALDI-MS spectrum of crude reaction mixture

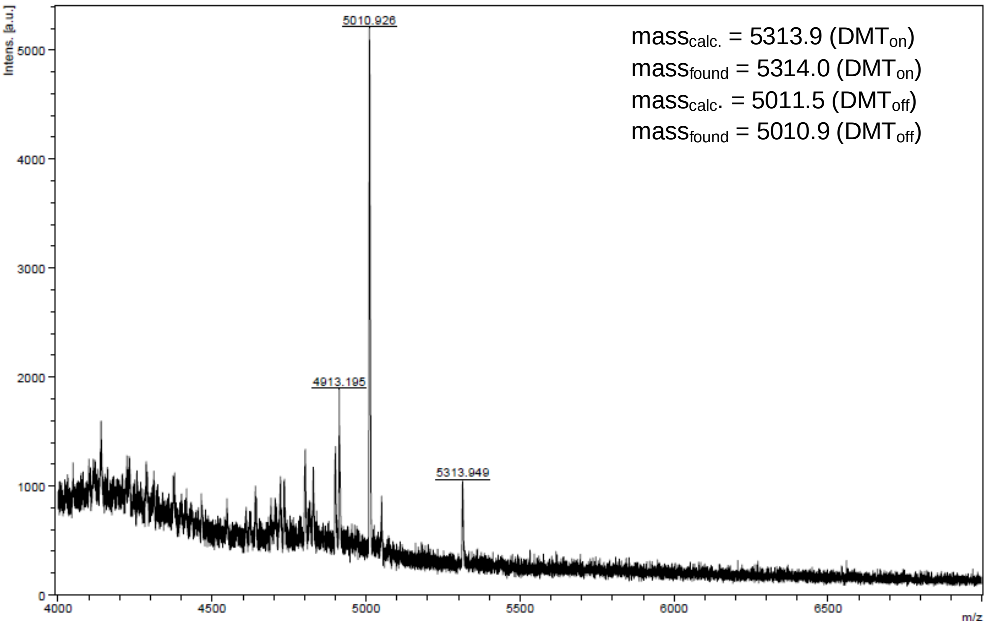

DNA conjugate 13: CPG-bound 16mer 7De-dATC-PEG(4)-NH_2 conjugate was reacted with 4-formyl-phenoxyacetic acid according to RP-04.

HPLC trace of crude reaction mixture **13** (Batch A, analytical RP-HPLC)

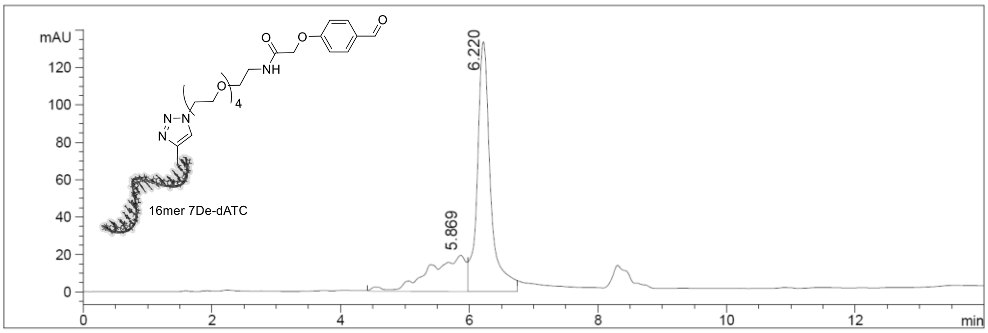

Peak list:

Ret. Time	Width min	Height	Area	Area %
5.869	0.662	19.392	770.620	28.875
6.220	0.236	133.784	1898.235	71.125

HPLC trace of crude reaction mixture **13** (Batch B, analytical RP-HPLC)

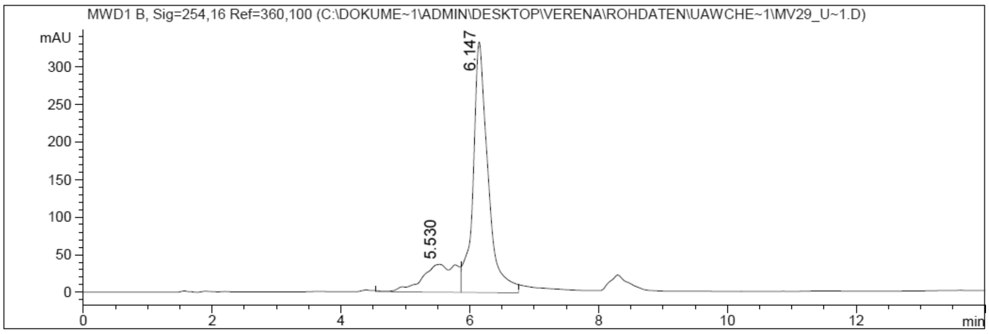

Peak list:

Ret. Time	Width min	Height	Area	Area %
5.530	0.626	37.105	1394.601	20.737
6.147	0.267	333.327	5330.540	79.263

HPLC trace of crude reaction mixture **13** (Batch C, analytical RP-HPLC)

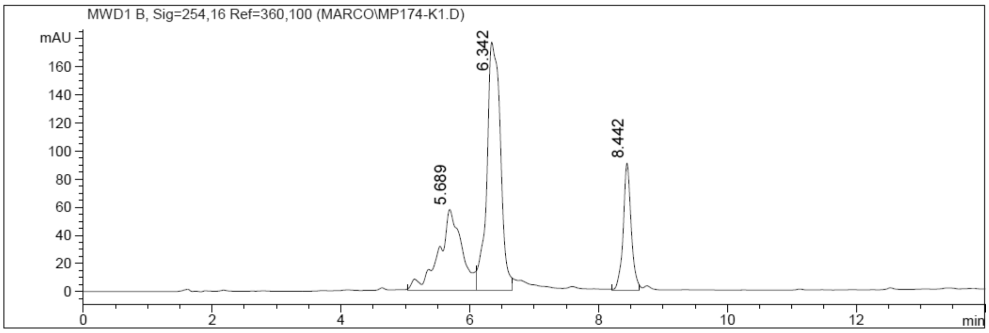

Peak list:

Ret. Time	Width min	Height	Area	Area %
5.689	0.418	57.653	1444.642	30.115
6.342	0.243	176.812	2573.615	53.650
8.442	0.143	90.691	778.797	16.235

HPLC trace of crude reaction mixture **13** (Batch D, analytical RP-HPLC)

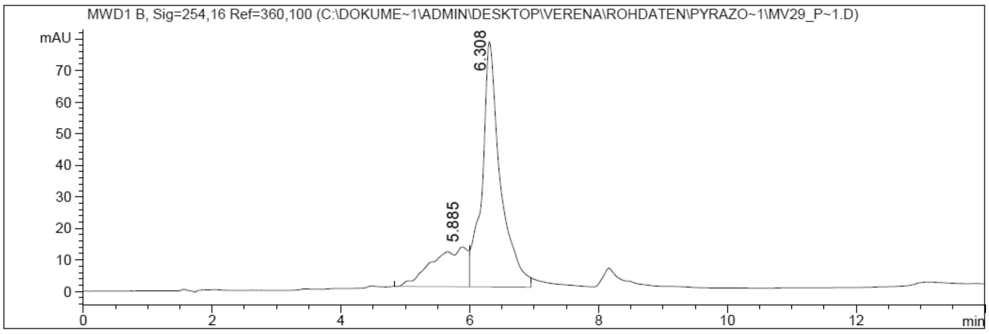

Peak list:

Ret. Time	Width min	Height	Area	Area %
5.885	0.628	12.621	475.272	23.565
6.308	0.331	77.614	1541.567	76.435

MALDI-MS spectrum of crude reaction mixture **13**

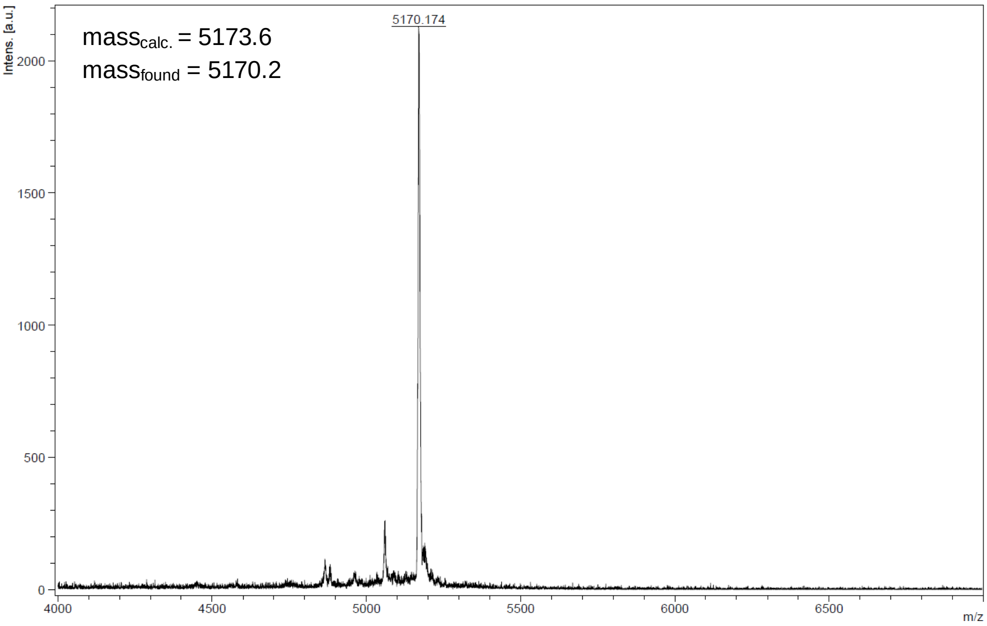

DNA conjugate 33: CPG-bound 16mer 7De-dATC-PEG(4)-NH_2 conjugate was reacted with *N*-Boc-tryptophan according to RP-04.

HPLC trace of crude reaction mixture **33** (analytical RP-HPLC)

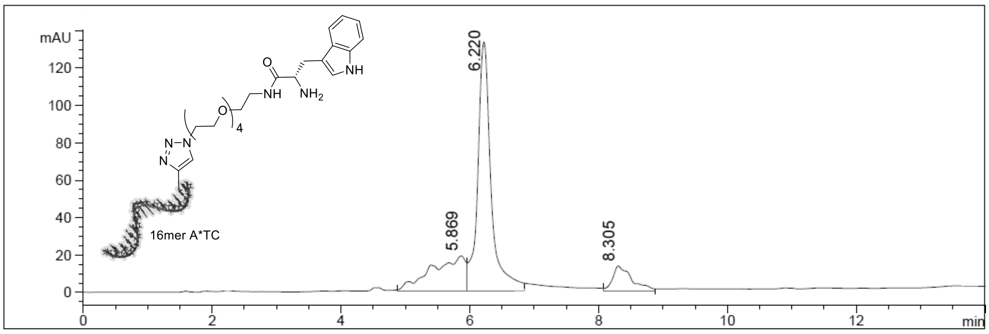

Peak list:

Ret. Time	Width min	Height	Area	Area %
5.869	0.602	18.840	680.488	23.719
6.220	0.240	133.165	1919.546	66.909
8.305	0.338	13.239	268.864	9.372

MALDI-MS spectrum of crude reaction mixture **33**

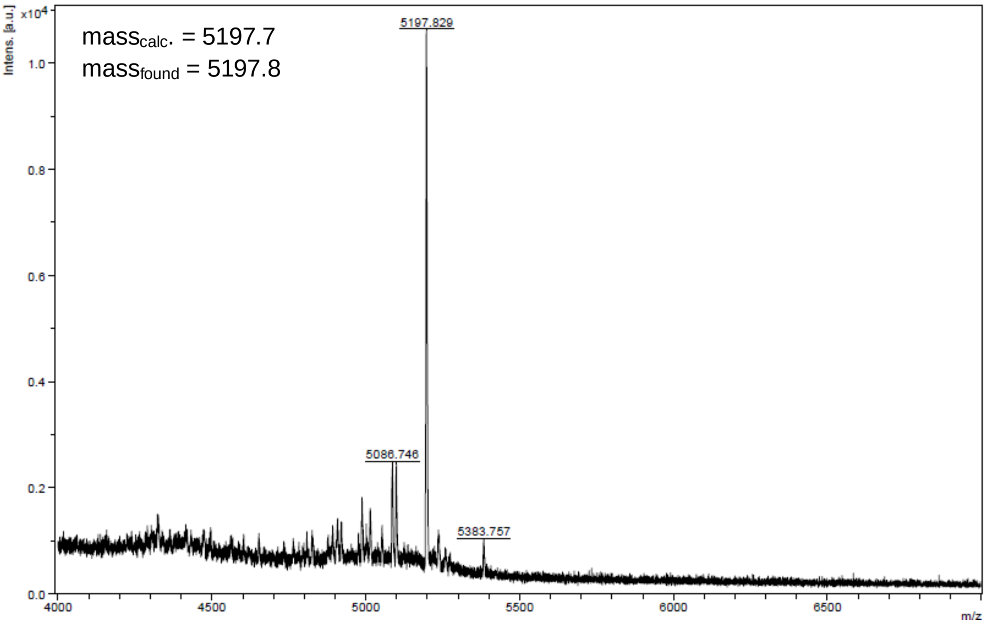

DNA conjugate 38: CPG-bound 16mer 7De-dATC-PEG(4)-NH_2 conjugate was reacted with *N*-Fmoc-piperidine-4-carboxylic acid according to RP-04.

HPLC trace of crude reaction mixture **38** (analytical RP-HPLC)

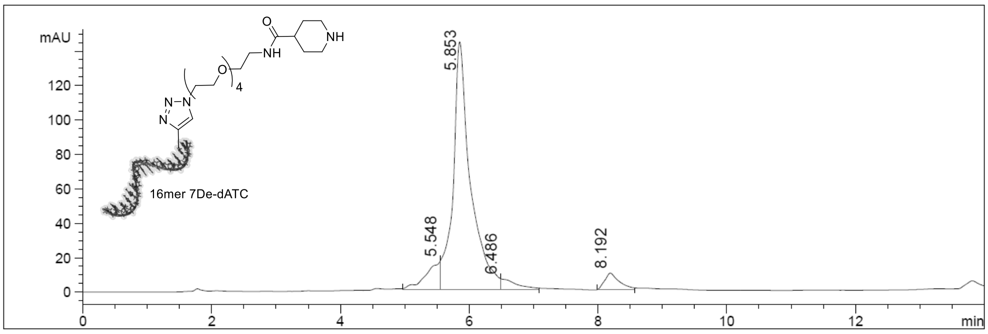

MALDI-MS spectrum of crude reaction mixture **38**

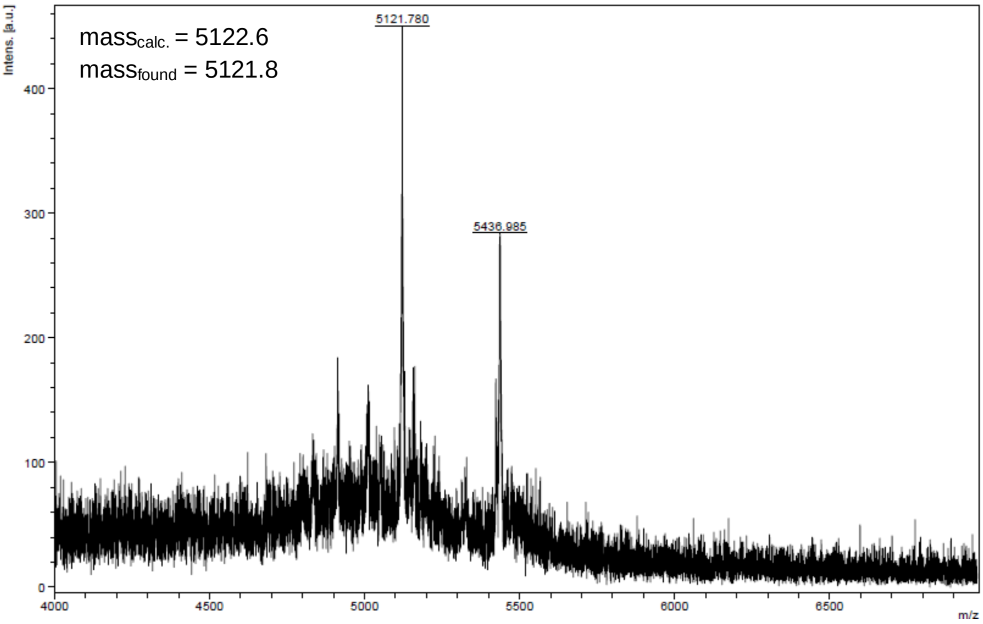

DNA conjugate 42: CPG-bound 16mer 7De-dATC-piperidine conjugate **38** was reacted with *N*-Fmoc-glycine according to RP-04.

HPLC trace of crude reaction mixture **42** (analytical RP-HPLC)

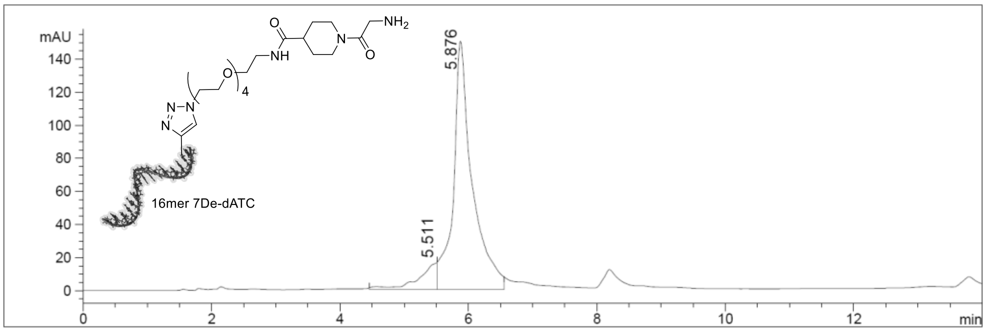

Peak list:

Ret. Time	Width min	Height	Area	Area %
5.511	0.332	16.547	329.330	9.763
5.876	0.338	150.227	3043.931	90.237

MALDI-MS spectrum of crude reaction mixture **42**

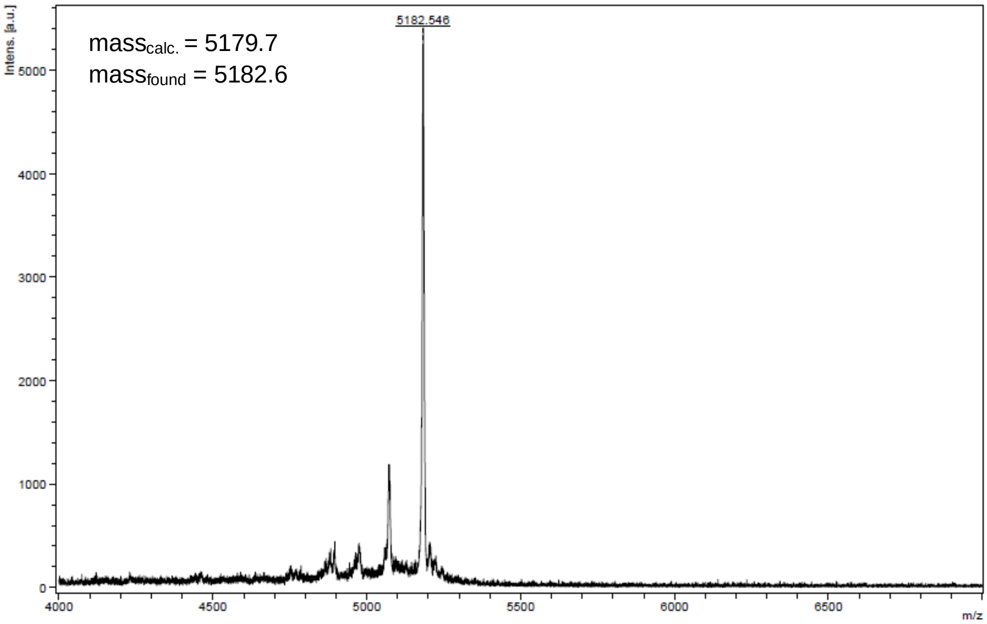

DNA conjugate 52: CPG-bound 16mer 7De-dATC-PEG(4)-NH_2 conjugate was reacted with 4-ethynylbenzoic acid according to RP-04.

HPLC trace of crude reaction mixture **52** (analytical RP-HPLC)

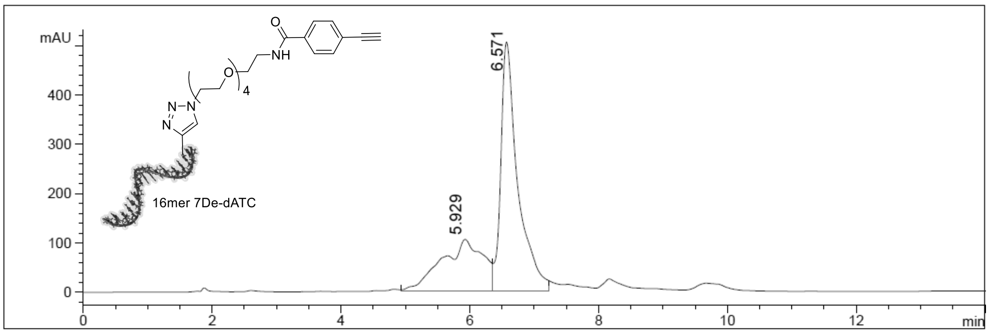

Peak list:

Ret. Time	Width min	Height	Area	Area %
5.929	0.743	104.156	4646.222	33.665
6.571	0.302	504.528	9155.146	66.335

MALDI-MS spectrum of crude reaction mixture **52**

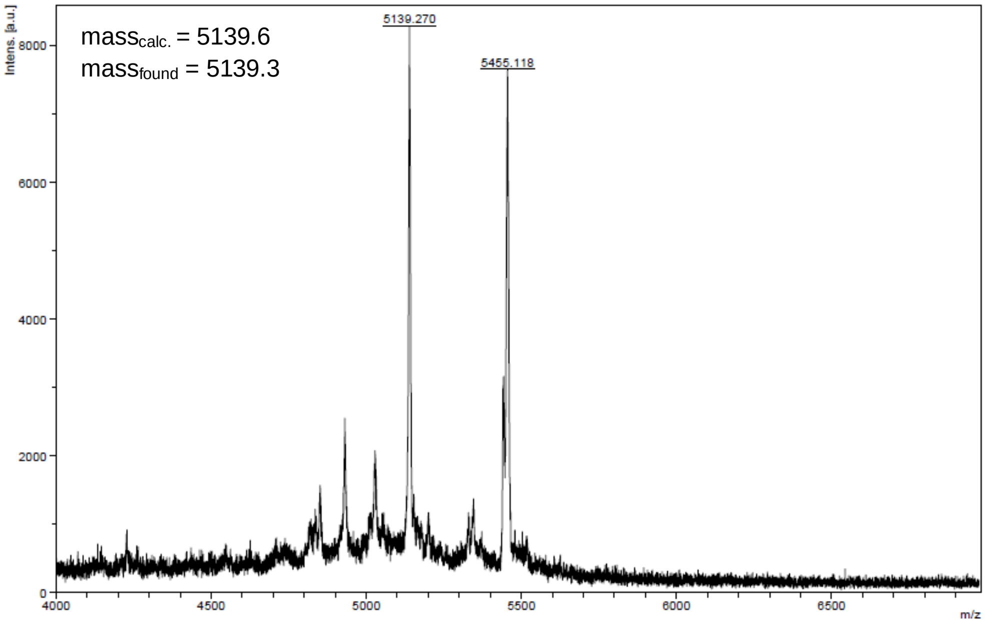

Isocyanide multicomponent reactions

Ugi four-component reaction

DNA conjugate 17: CPG-bound 16mer 7De-dATC-aldehyde conjugate **13** (Batch A) was reacted with acetic acid **14a**, propargylamine **15** and *tert*-butylisocyanide **16** according to RP-05.

HPLC trace of crude reaction mixture **17** (analytical RP-HPLC)

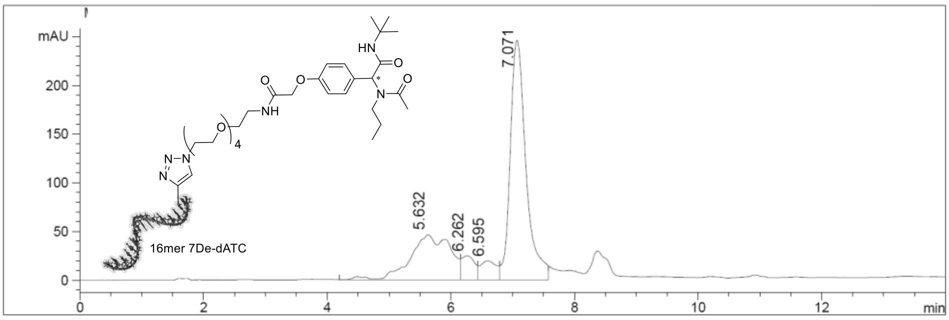

Peak list:

Ret. Time	Width min	Height	Area	Area %
5.632	0.742	46.341	2061.850	29.083
6.262	0.243	24.522	356.809	5.033
6.595	0.299	19.315	346.382	4.886
7.071	0.293	245.685	4324.537	60.999

HPLC trace of isolated product **17** (analytical RP-HPLC)

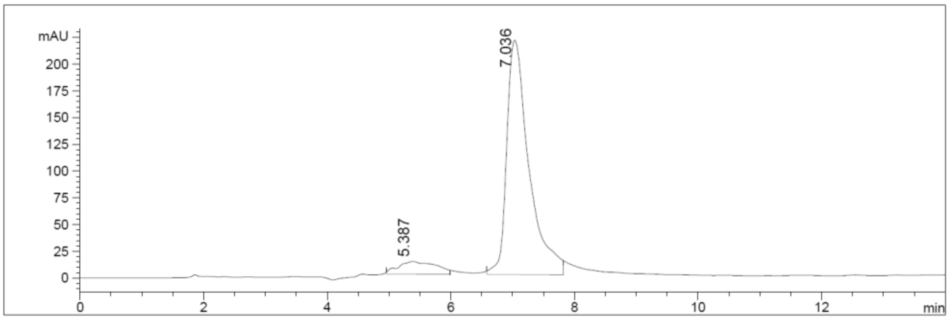

Peak list:

Ret. Time	Width min	Height	Area	Area %
5.387	0.705	12.043	509.625	8.520
7.036	0.416	219.312	5472.196	91.480

MALDI-MS spectrum of isolated product **17**

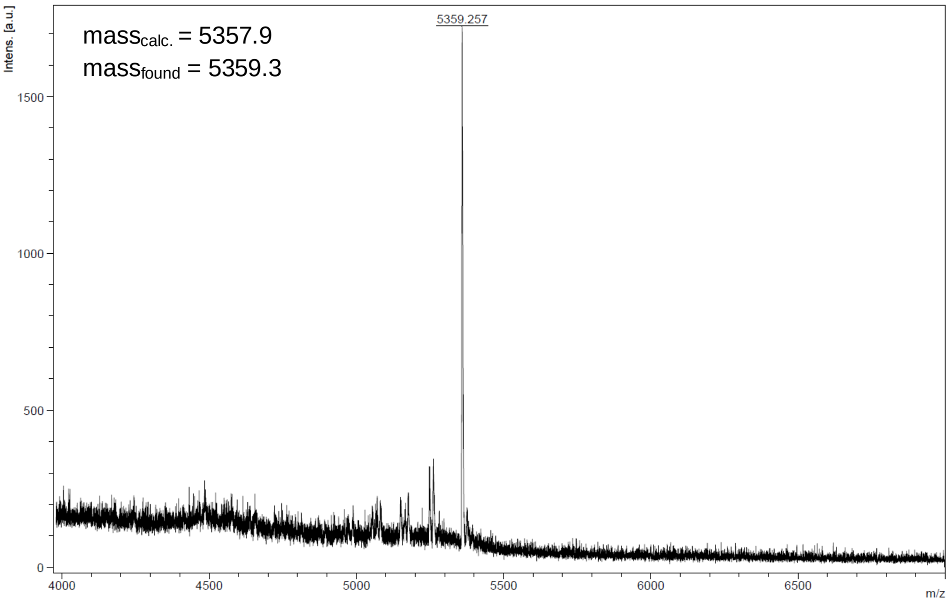

Ugi-azide three-component reaction

DNA conjugate 20: CPG-bound 16mer 7De-dATC-aldehyde conjugate **13** (Batch A) was reacted with piperidine **18**, *tert*-butylisocyanide **16** and trimethylsilyl azide **19** according to RP-06.

HPLC trace of crude reaction mixture **20** (analytical RP-HPLC)

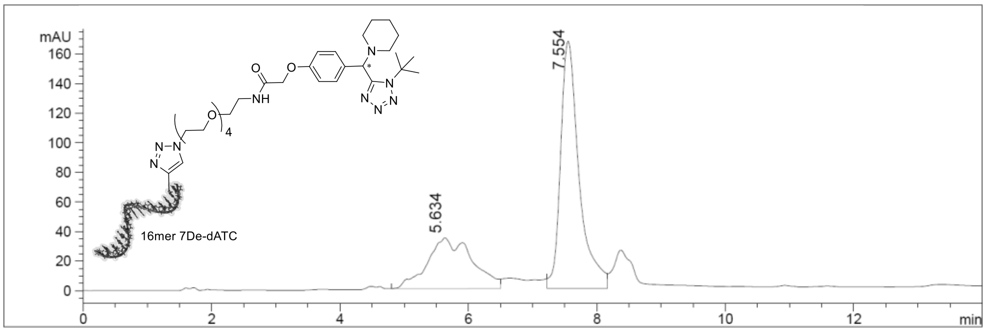

Peak list:

Ret. Time	Width min	Height	Area	Area %
5.634	0.810	34.364	1670.308	33.360
7.554	0.333	167.206	3336.638	66.640

HPLC trace of isolated product **20** (analytical RP-HPLC)

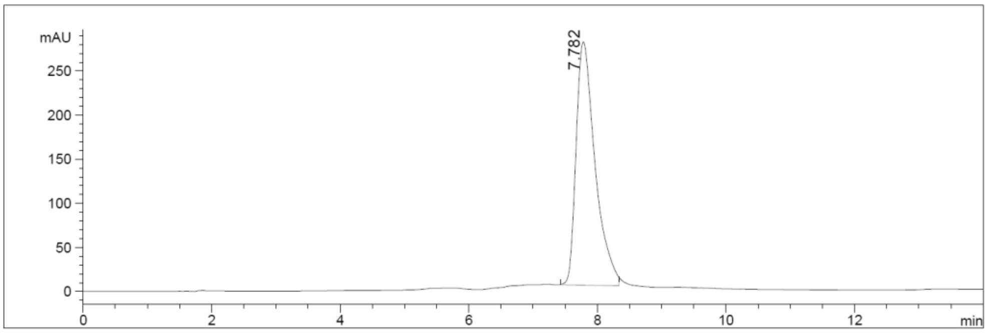

Peak list:

Ret. Time	Width min	Height	Area	Area %
7.782	0.341	276.131	5645.609	100.000

MALDI-MS spectrum of isolated product **20**

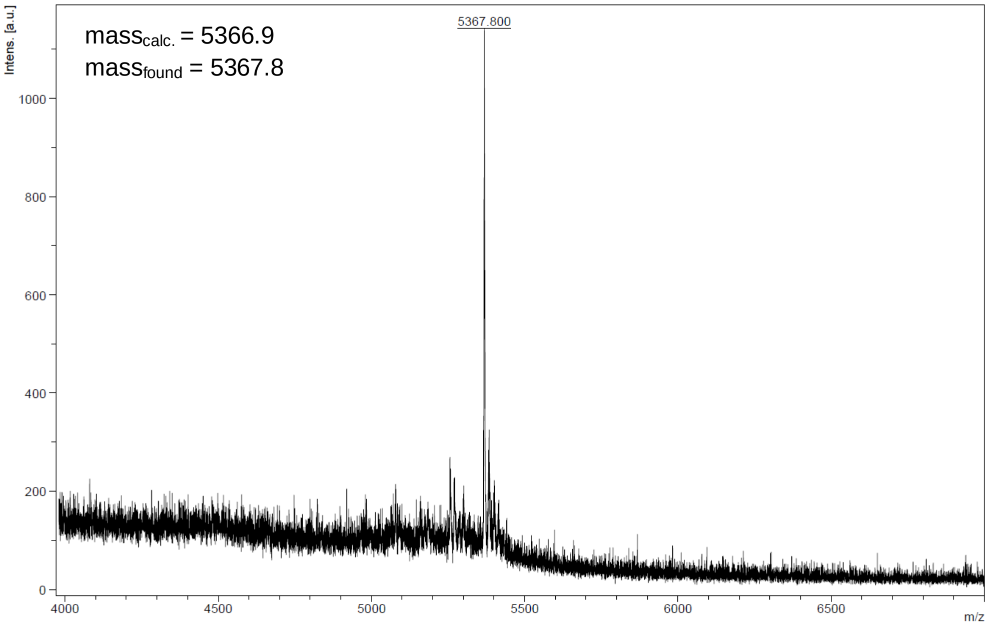

Groebke-Blackburn-Bienyamé three-component reaction

DNA conjugate 22: CPG-bound 16mer 7De-dATC-aldehyde conjugate **13** (Batch A) was reacted with 2-aminopyridine **21** and *tert*-butylisocyanide **16** according to RP-07.

HPLC trace of crude reaction mixture **22** (analytical RP-HPLC)

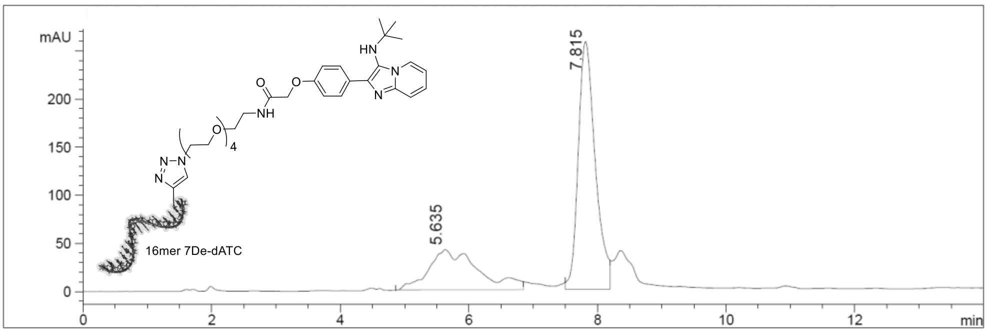

Peak list:

Ret. Time	Width min	Height	Area	Area %
5.635	0.896	41.781	2247.255	32.201
7.815	0.306	257.501	4731.628	67.799

HPLC trace of isolated product **22** (analytical RP-HPLC)

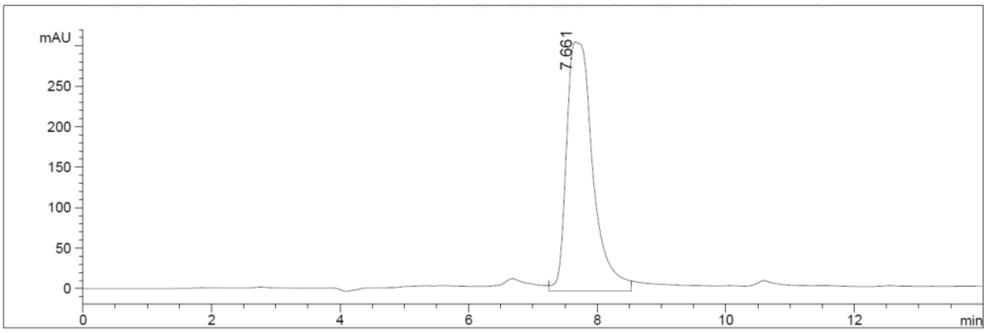

Peak list:

Ret. Time	Width min	Height	Area	Area %
7.661	0.476	307.232	8772.663	100.000

MALDI-MS spectrum of isolated product **22**

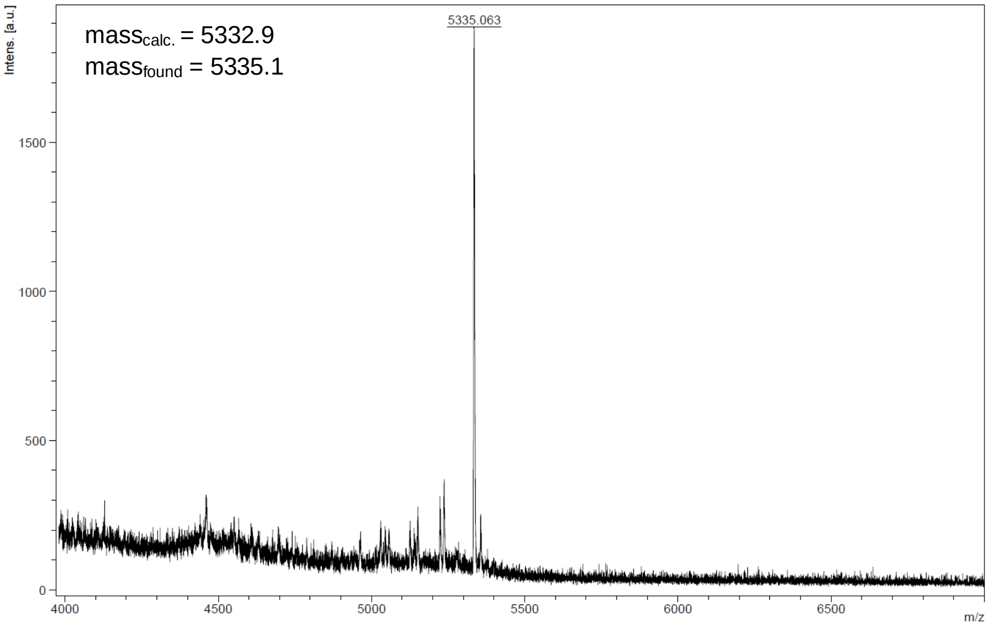

Ugi four-component/aza-Wittig reaction

Table S9 – HPLC traces for the U-4CR/aza-Wittig reaction on different DNA sequences.

**Oligonucleotide**	**HPLC traces**	**DNA degradation [%]**
10mer ATGC **57**	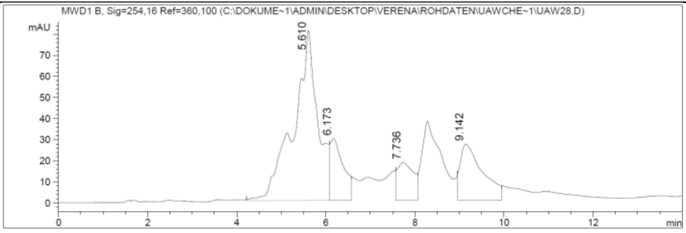	32
16mer 7De-dATC **25a**	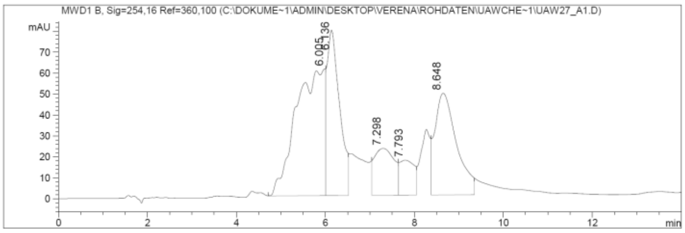	24

10mer ATGC = 5'-GTCATGATCT-3’, 16mer 7De-dATC = 5'-CT*C TCT TT7De-dA 7De-dACT 7De-dACC T-3'

Table S10 – Scope of the U-4CR/aza-Wittig reaction on CPG-bound 16mer 7De-dATC oligonucleotide-aldehyde conjugate **13** using different carboxylic acids **14**.[a]

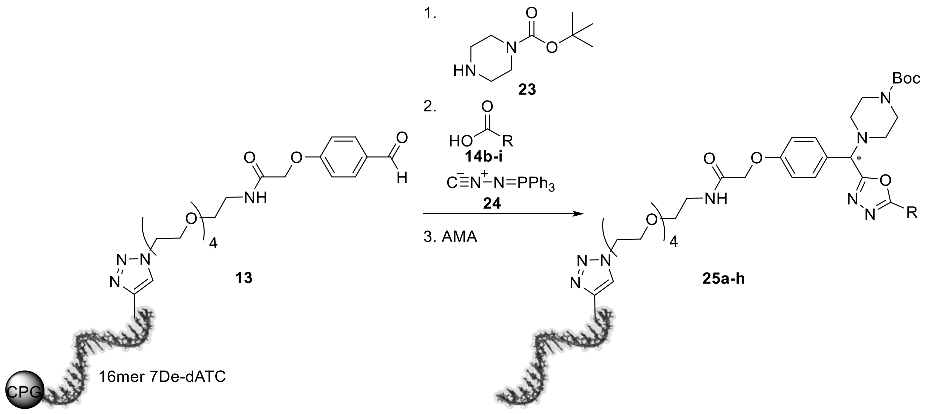

Entry	Product	Acid 14	Conversion [%][b]	DNA degradation [%][c]	$Mass_{calc.}$ $Mass_{found}$[d]
1	**25a**	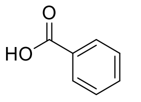 **14b**	39	24	5488.0<br>5489.1
2	**25b**	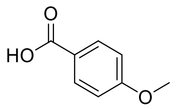 **14c**	39	28	5518.0<br>5518.4
3	**25c**	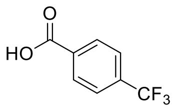 **14d**	32	22	5556.0<br>5557.2
4	**25d**	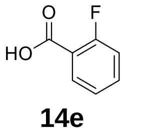 **14e**	41	23	5506.0<br>5506.9
5	**25e**	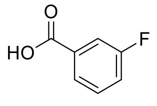 **14f**	33	14	5506.0<br>5507.7
6	**25f**	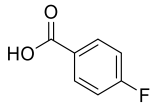 **14g**	49	18	5506.0<br>5507.5
7	**25g**	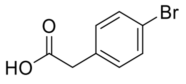 **14h**	41	35	5580.9<br>5581.9
8	**25h**	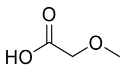 **14i**	49	29	5456.0<br>5457.0

[a] CPG-bound oligonucleotide conjugate **13** (20 nmol) with *N*-Boc-piperazine (1000 equiv.,20 µmol) in 30 µL in 1,2-dichloroethane at ambient temperature for 3 h, followed by addition of carboxylic acid **14** (1000 equiv., 20 µmol) and (isocyanoimino)triphenylphosphorane **24** (1000 equiv., 20 µmol) together suspended in 90 µL 1,2-dichloroethane and shaking of the reaction mixture at 50 °C for 16 h. DNA cleavage with AMA (30% aqueous ammonia / 40% aqueous methylamine, 1:1 (vol/vol)) at ambient temperature for 4 h. [b] Determined by analytical RP-HPLC analysis based on the ratios of **25** to **13**. [c] Determined by comparison of purities of the analytical RP-HPLC traces of starting material **13** and the crude reaction mixture **25**. [d] Measured by MALDI-MS. 16mer 7De-dATC = 5'-CT*C TCT TT7De-dA 7De-dACT 7De-dACC T-3'.

DNA conjugate 57: CPG-bound 10mer ATCG-aldehyde conjugate **56** was reacted with benzoic acid **14b** and *N*-Boc-piperazine **23** according to RP-08.

HPLC trace of starting material **56** (analytical RP-HPLC)

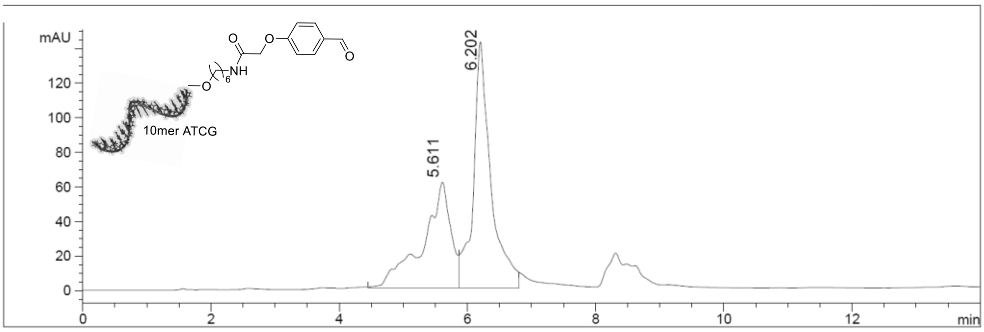

Peak list:

Ret. Time	Width min	Height	Area	Area %
5.611	0.511	61.380	1880.840	41.218
6.202	0.314	142.597	2682.314	58.782

HPLC trace of crude reaction mixture **57** (analytical RP-HPLC)

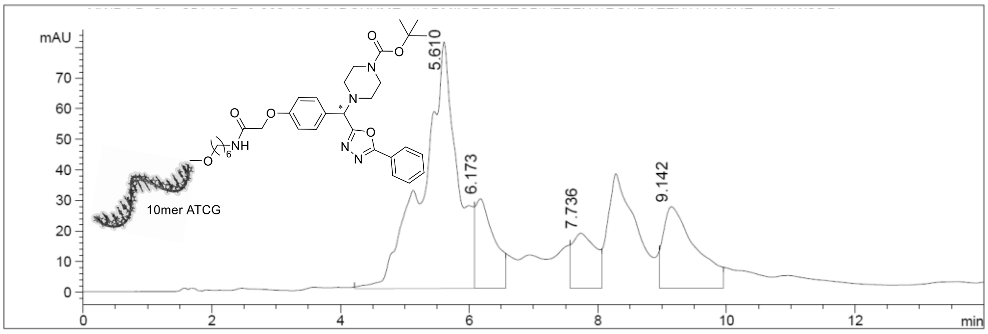

Peak list:

Ret. Time	Width min	Height	Area	Area %
5.610	0.624	80.652	3020.255	60.372
6.173	0.338	29.326	594.561	11.885
7.736	0.430	17.938	462.604	9.247
9.142	0.581	26.554	925.343	18.497

MALDI-MS spectrum of reaction crude **57**

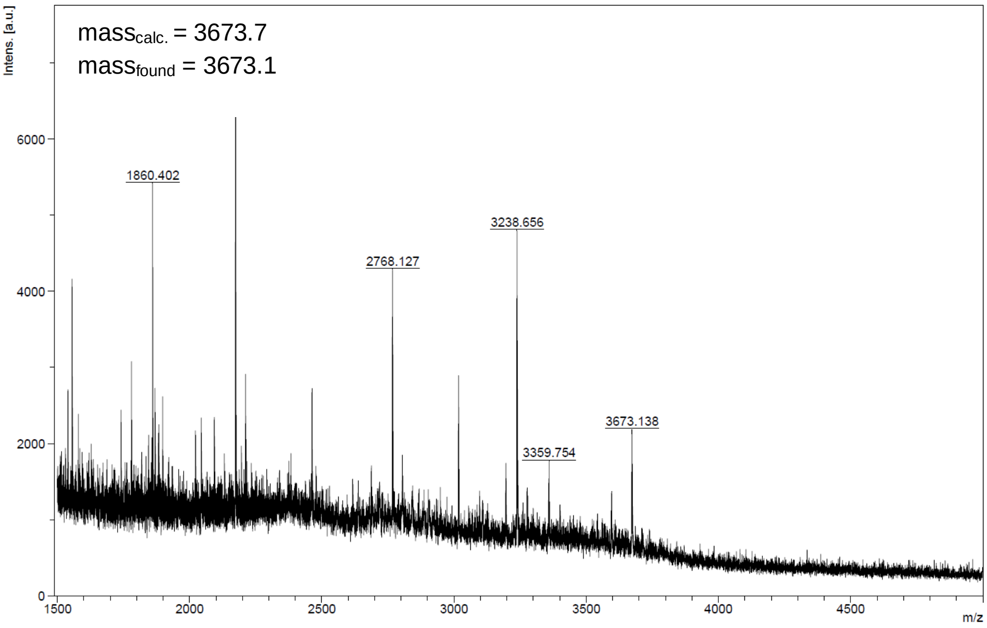

DNA conjugate 13: CPG-bound 16mer 7De-dATC-PEG(4)-NH_2 conjugate was reacted with 4-formyl-phenoxyacetic acid according to RP-04.

HPLC trace of crude reaction mixture **13** (Batch B, analytical RP-HPLC)

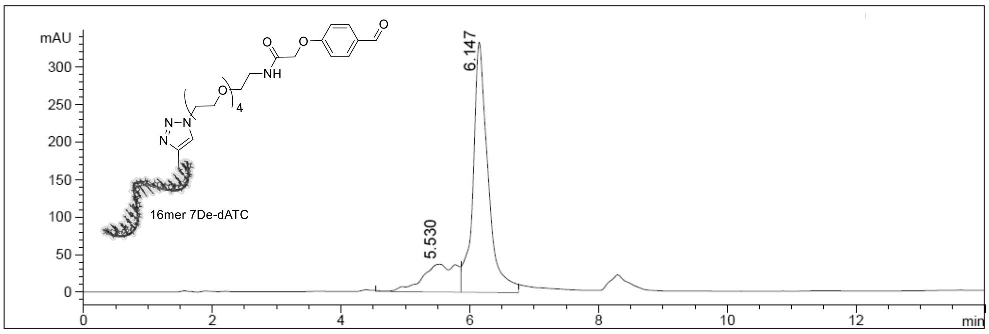

Peak list:

Ret. Time	Width min	Height	Area	Area %
5.530	0.626	37.105	1394.601	20.737
6.147	0.267	333.327	5330.540	79.263

MALDI-MS spectrum of crude reaction mixture **13**

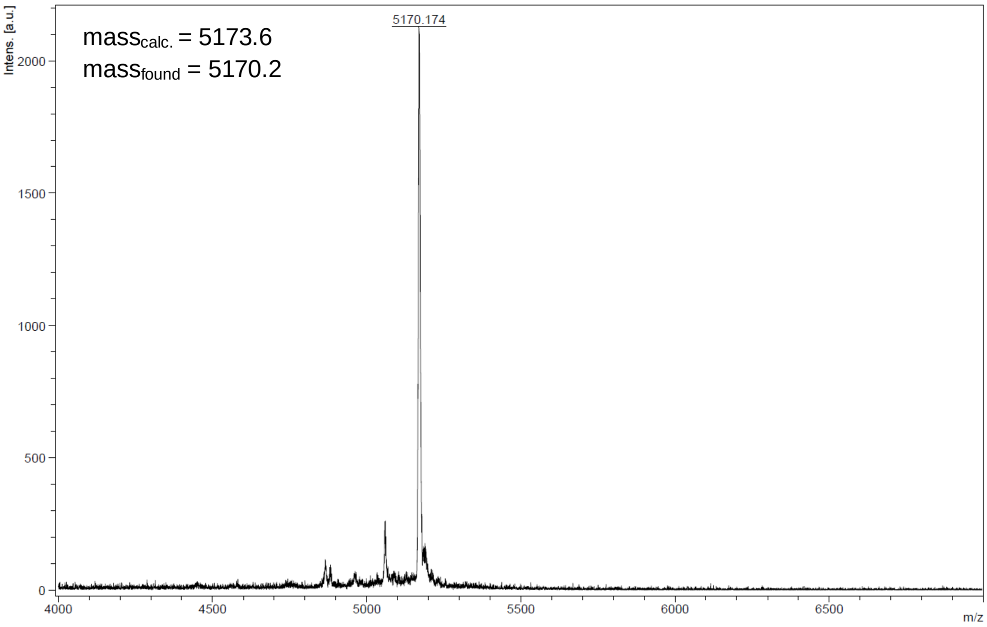

DNA conjugate 25a: CPG-bound 16mer 7De-dATC-aldehyde conjugate **13** (Batch B) was reacted with benzoic acid **14b,** *N*-Boc-piperazine **23** and (isocyanoimino)triphenylphosphorane **24** according to RP-08.

HPLC trace of crude reaction mixture **25a** (analytical RP-HPLC)

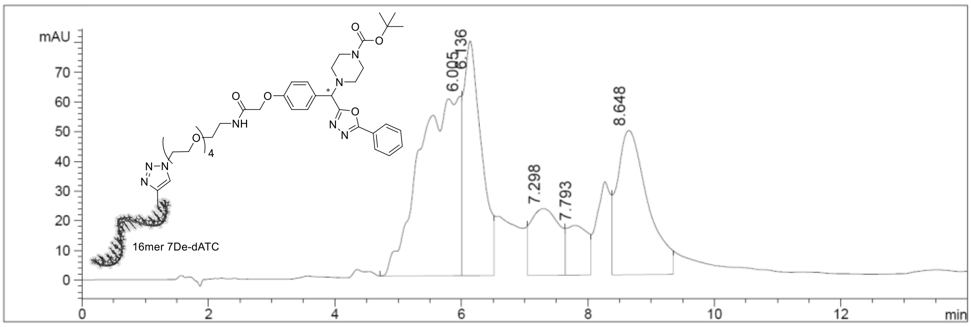

Peak list:

Ret. Time	Width min	Height	Area	Area %
6.005	0.748	60.481	2714.790	38.881
6.136	0.329	78.990	1557.483	22.306
7.298	0.523	22.384	702.436	10.060
7.793	0.378	16.715	379.348	5.433
8.648	0.559	48.532	1628.242	23.320

HPLC trace of isolated product **25a** (analytical RP-HPLC)

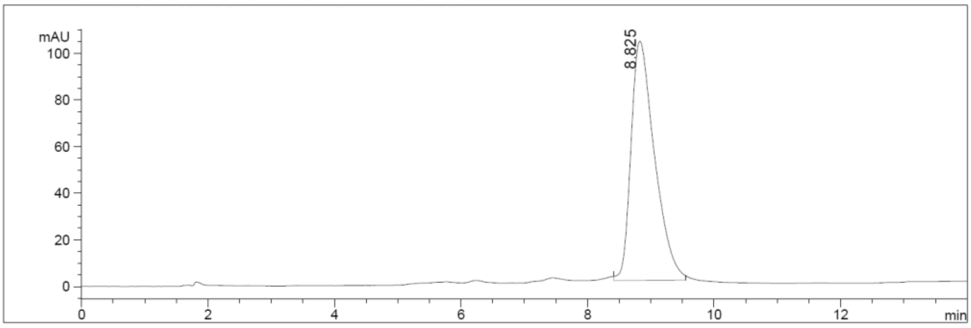

Peak list:

Ret. Time	Width min	Height	Area	Area %
8.825	0.433	102.565	2663.775	100.000

MALDI-MS spectrum of isolated product **25a**

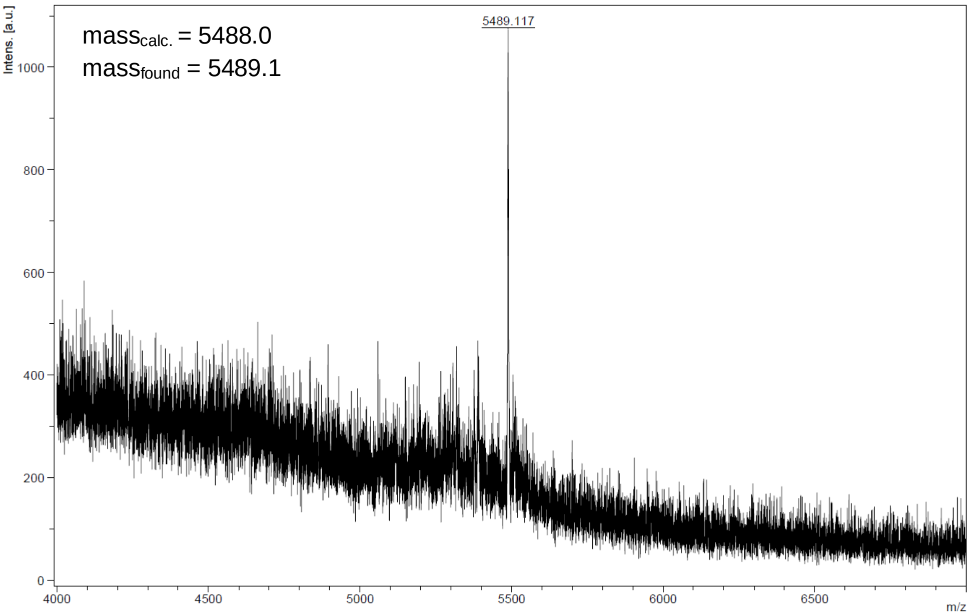

DNA conjugate 25b: CPG-bound 16mer 7De-dATC-aldehyde conjugate **13** (Batch B) was reacted with 4-methoxybenzoic acid **14c,** *N*-Boc-piperazine **23** and (isocyanoimino)triphenylphosphorane **24** according to RP-08.

HPLC trace of crude reaction mixture **25b** (analytical RP-HPLC)

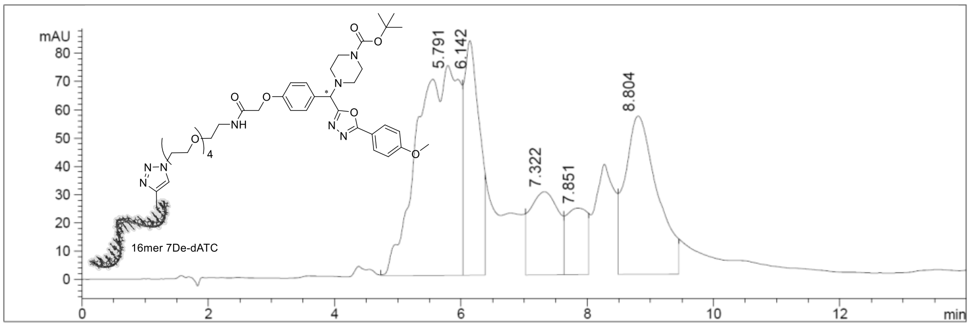

Peak list:

Ret. Time	Width min	Height	Area	Area %
5.791	0.783	74.237	3486.988	42.048
6.142	0.274	82.967	1362.656	16.432
7.322	0.531	29.371	935.881	11.285
7.851	0.372	23.545	525.018	6.331
8.804	0.590	56.038	1982.388	23.905

HPLC trace of isolated product **25b** a RP-HPLC)

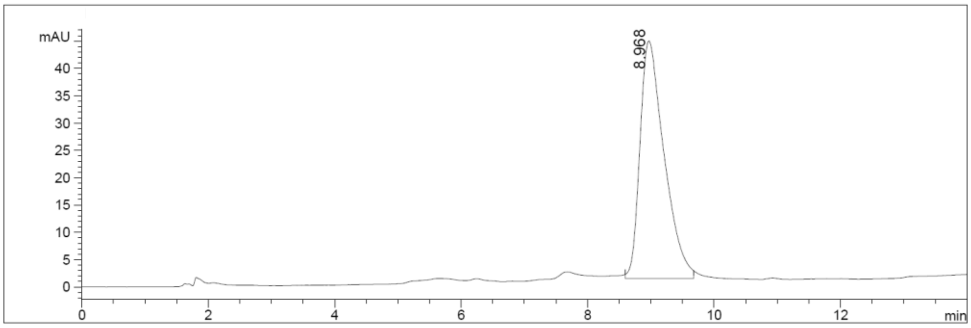

Peak list:

Ret. Time	Width min	Height	Area	Area %
8.968	0.445	43.491	1160.373	100.000

MALDI-MS spectrum of isolated product **25b**

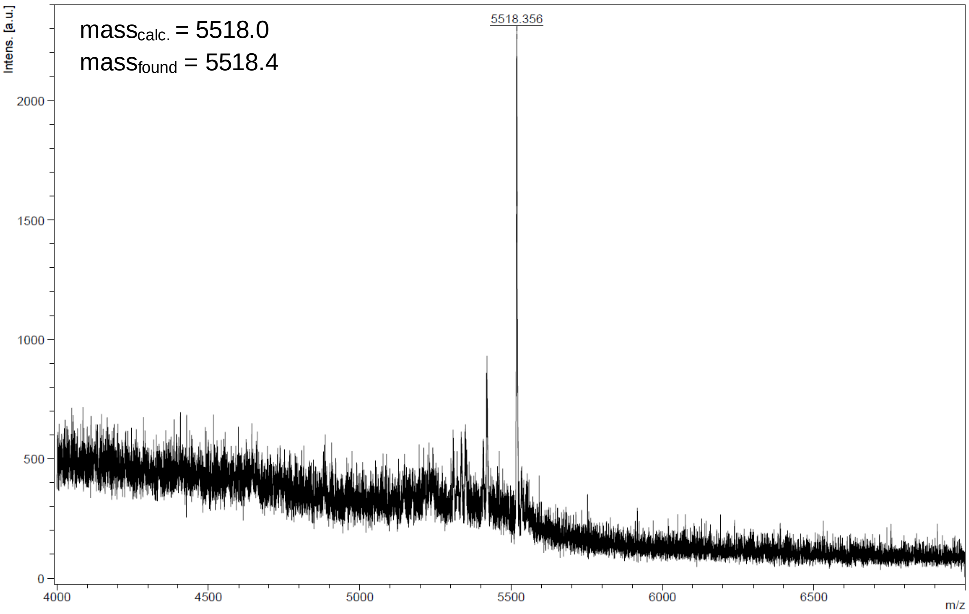

DNA conjugate 25c: CPG-bound 16mer 7De-dATC-aldehyde conjugate **13** (Batch B) was reacted with 4-(trifluoromethyl)benzoic acid **14d,** *N*-Boc-piperazine **23** and (isocyanoimino)triphenylphosphorane **24** according to RP-08.

HPLC trace of crude reaction mixture **25c** (analytical RP-HPLC)

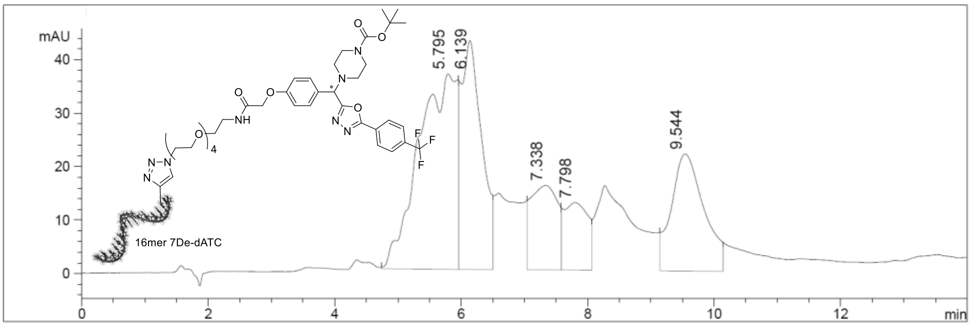

Peak list:

Ret. Time	Width min	Height	Area	Area %
5.795	0.702	36.506	1536.780	37.602
6.139	0.376	42.755	964.435	23.598
7.338	0.490	15.785	464.290	11.360
7.798	0.444	12.619	336.000	8.221
9.544	0.598	21.875	785.493	19.219

HPLC trace of isolated product **25c** (analytical RP-HPLC)

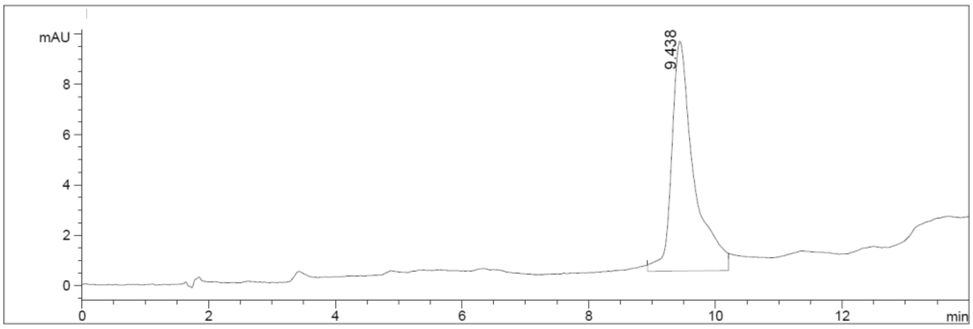

Peak list:

Ret. Time	Width min	Height	Area	Area %
9.438	0.407	9.118	222.668	100.000

MALDI-MS spectrum of isolated product **25c**

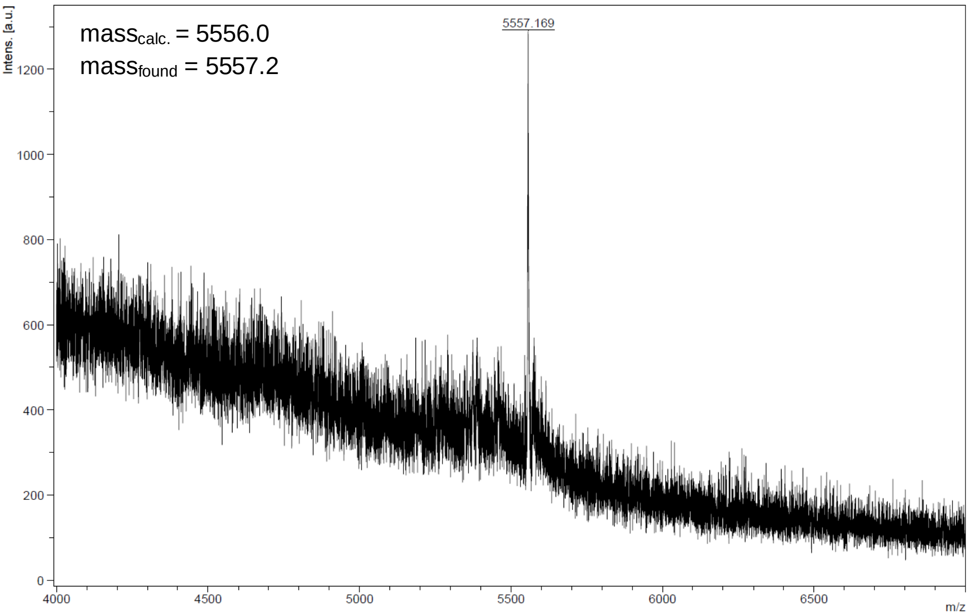

DNA conjugate 25d: CPG-bound 16mer 7De-dATC-aldehyde conjugate **13** (Batch B) was reacted with 2-fluorobenzoic acid **14e,** *N*-Boc-piperazine **23** and (isocyanoimino)triphenylphosphorane **24** according to RP-08.

HPLC trace of crude reaction mixture **25d** (analytical RP-HPLC)

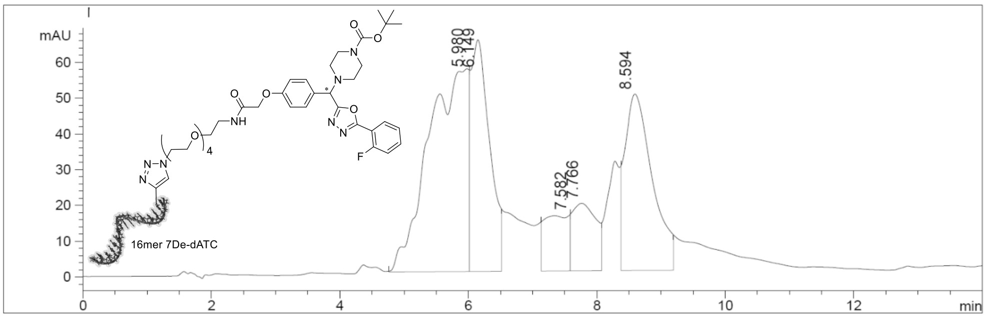

Peak list:

Ret. Time	Width min	Height	Area	Area %
5.980	0.713	56.621	2423.285	39.767
6.149	0.338	64.711	1314.006	21.563
7.582	0.433	15.730	408.723	6.707
7.766	0.431	18.854	487.767	8.004
8.594	0.493	49.305	1459.885	23.957

HPLC trace of isolated product **25d** (analytical RP-HPLC)

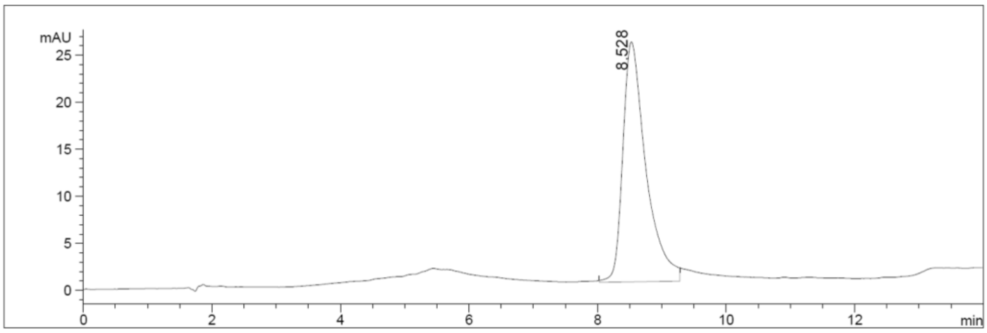

Peak list:

Ret. Time	Width min	Height	Area	Area %
8.528	0.418	25.499	639.469	100.000

MALDI-MS spectrum of isolated product **25d**

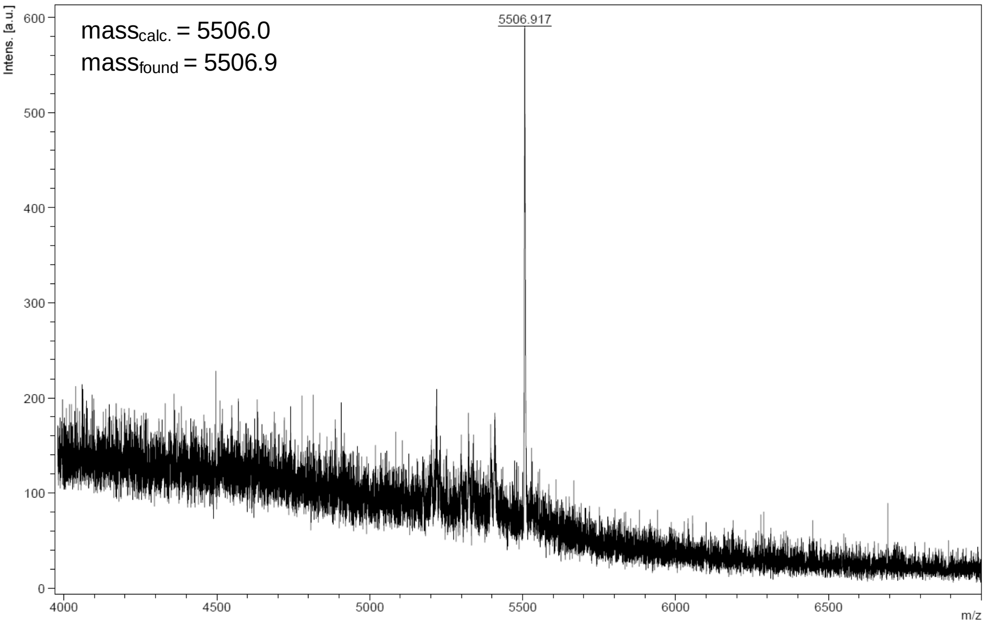

DNA conjugate 25e: CPG-bound 16mer 7De-dATC-aldehyde conjugate **13** (Batch B) was reacted with 3-fluorobenzoic acid **14f,** *N*-Boc-piperazine **23** and (isocyanoimino)triphenylphosphorane **24** according to RP-08.

HPLC trace of crude reaction mixture **25e** (analytical RP-HPLC)

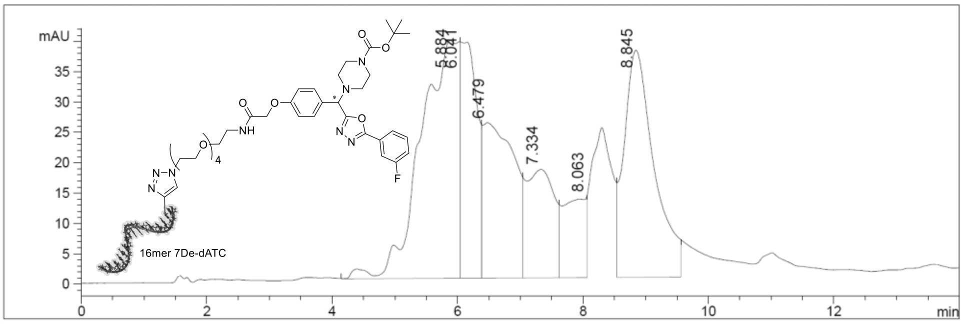

Peak list:

Ret. Time	Width min	Height	Area	Area %
5.884	0.699	39.184	1642.346	30.611
6.041	0.303	38.944	707.594	13.189
6.479	0.575	25.601	883.536	16.468
7.334	0.518	17.844	555.108	10.347
8.063	0.419	13.376	336.287	6.268
8.845	0.553	37.401	1240.281	23.117

HPLC trace of isolated product **25e** (analytical RP-HPLC)

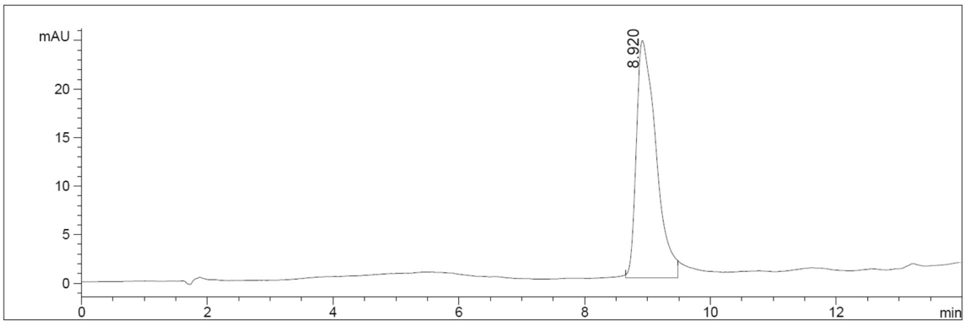

Peak list:

Ret. Time	Width min	Height	Area	Area %
8.920	0.362	24.406	529.737	100.000

MALDI-MS spectrum of isolated product **25e**

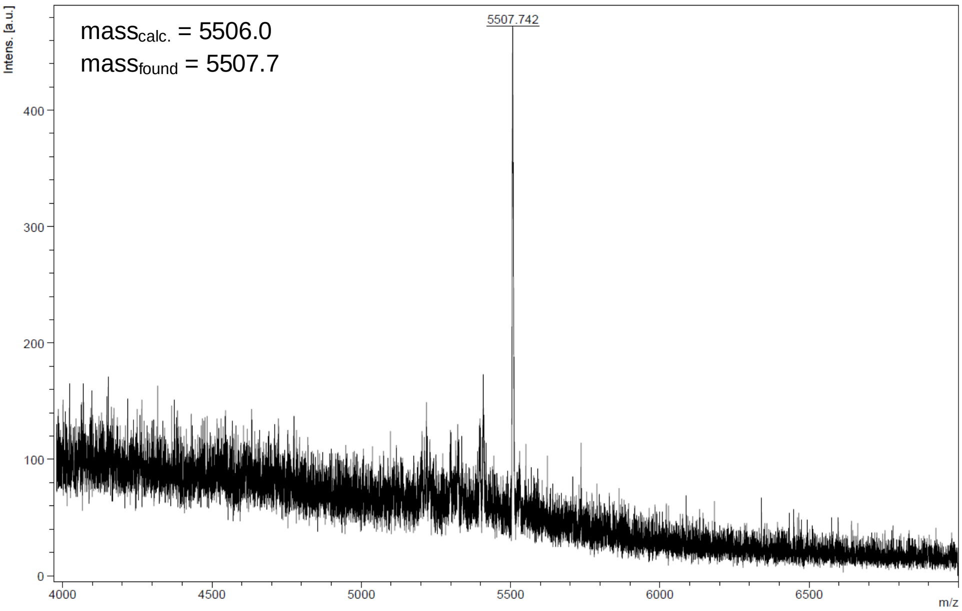

DNA conjugate 25f: CPG-bound 16mer 7De-dATC-aldehyde conjugate **13** (Batch B) was reacted with 4-fluorobenzoic acid **14g,** *N*-Boc-piperazine **23** and (isocyanoimino)triphenylphosphorane **24** according to RP-08.

HPLC trace of crude reaction mixture **25f** (analytical RP-HPLC)

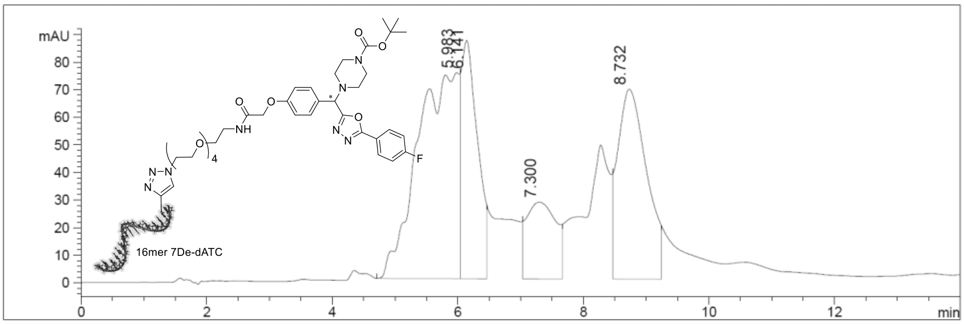

Peak list:

Ret. Time	Width min	Height	Area	Area %
5.983	0.762	74.637	3413.717	42.345
6.141	0.297	86.322	1540.295	19.106
7.300	0.552	27.786	920.575	11.419
8.732	0.529	68.859	2187.067	27.129

HPLC trace of isolated product **25f** (analytical RP-HPLC)

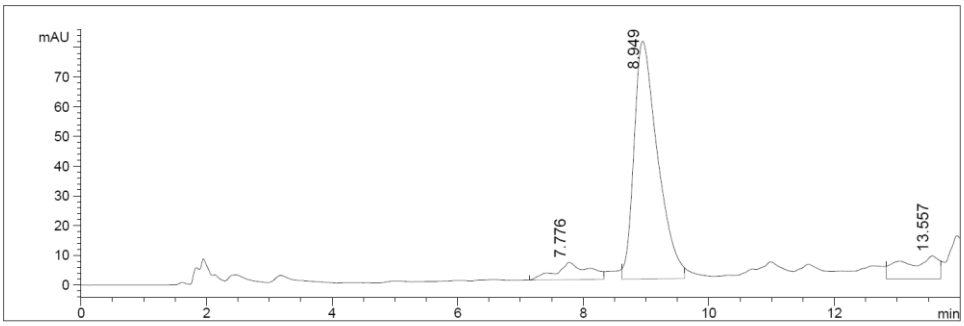

Peak list:

Ret. Time	Width min	Height	Area	Area %
7.776	0.602	5.820	210.306	8.121
8.949	0.437	79.905	2093.501	80.842
13.557	0.625	7.620	285.816	11.037

MALDI-MS spectrum of isolated product **25f**

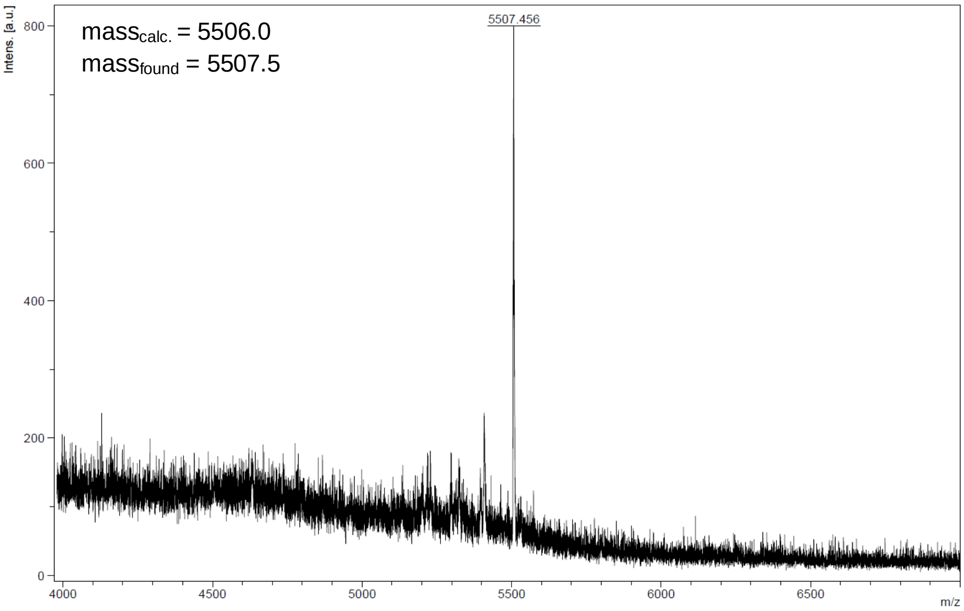

DNA conjugate 25g: CPG-bound 16mer 7De-dATC-aldehyde conjugate **13** (Batch B) was reacted with 2-(4-bromophenyl)acetic acid **14h,** *N*-Boc-piperazine **23** and (isocyanoimino)triphenylphosphorane **24** according to RP-08.

HPLC trace of crude reaction mixture **25g** (analytical RP-HPLC)

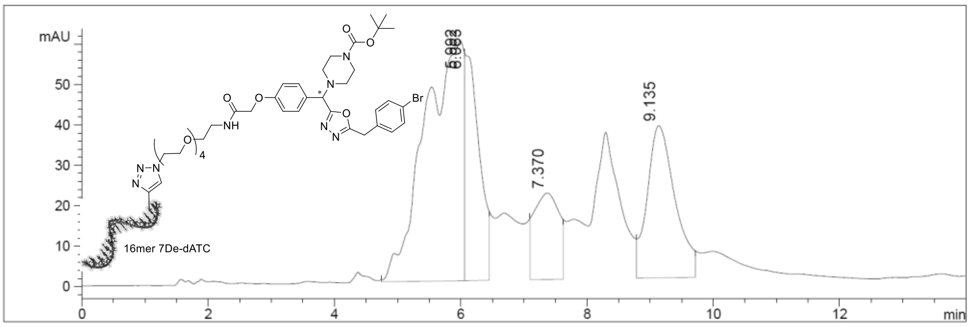

Peak list:

Ret. Time	Width min	Height	Area	Area %
5.992	0.708	59.360	2523.295	49.031
6.063	0.260	56.276	878.387	17.068
7.370	0.455	21.406	584.821	11.364
9.135	0.513	37.662	1159.805	22.537

HPLC trace of isolated product **25g** (analytical RP-HPLC)

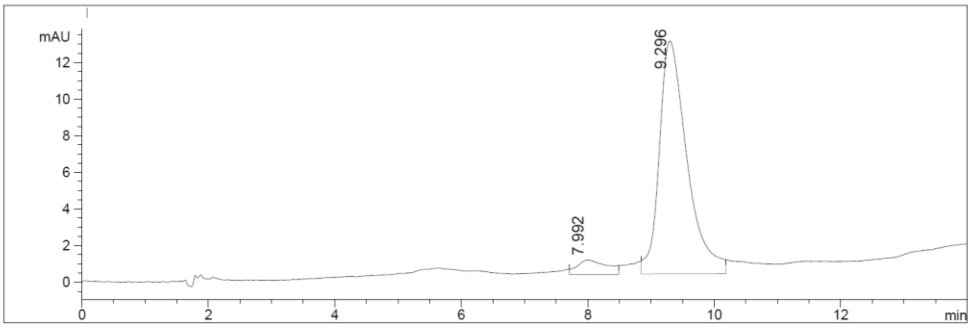

Peak list:

Ret. Time	Width min	Height	Area	Area %
7.992	0.551	0.775	25.606	6.205
9.296	0.507	12.723	387.062	93.795

MALDI-MS spectrum of isolated product **25g**

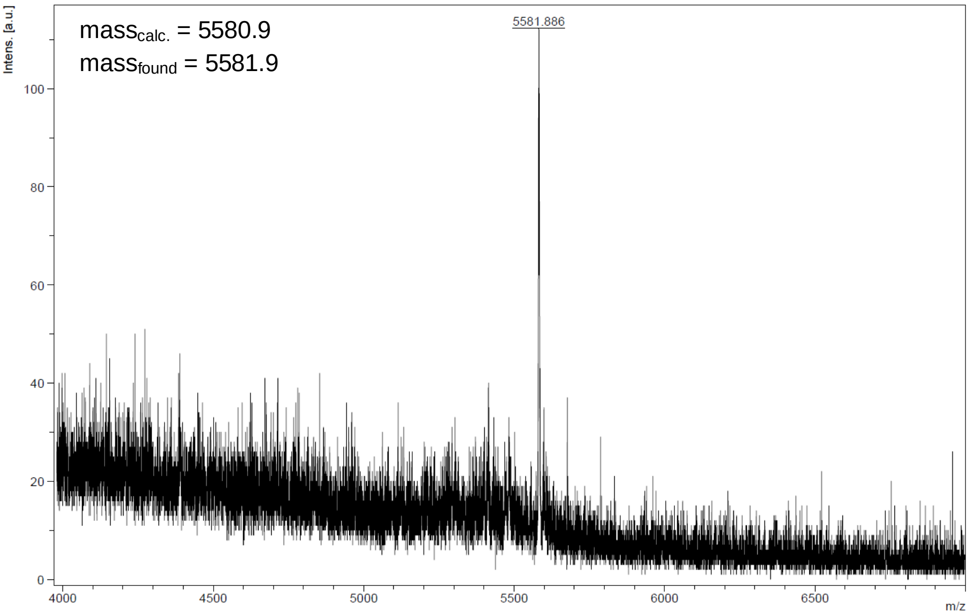

DNA conjugate 25h: CPG-bound 16mer 7De-dATC-aldehyde conjugate **13** (Batch B) was reacted with methoxy acetic acid **14i,** *N*-Boc-piperazine **23** and (isocyanoimino)triphenylphosphorane **24** according to RP-08.

HPLC trace of crude reaction mixture **25h** (analytical RP-HPLC)

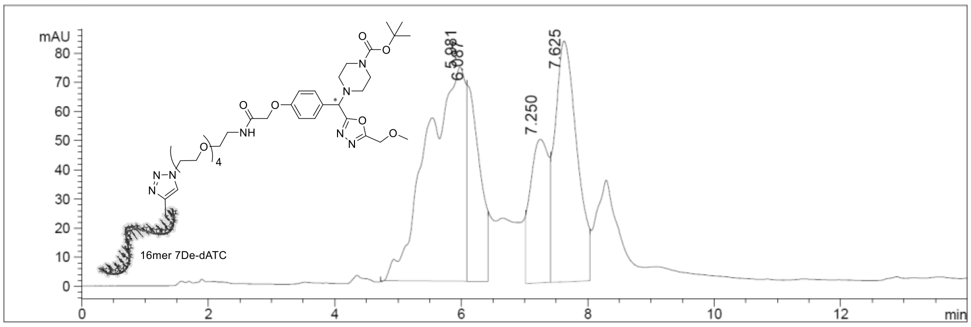

Peak list:

Ret. Time	Width min	Height	Area	Area %
5.981	0.698	73.412	3073.381	44.013
6.087	0.231	67.261	931.621	13.342
7.250	0.334	49.164	985.907	14.119
7.625	0.402	82.573	1991.958	28.526

HPLC trace of isolated product **25h** (analytical RP-HPLC)

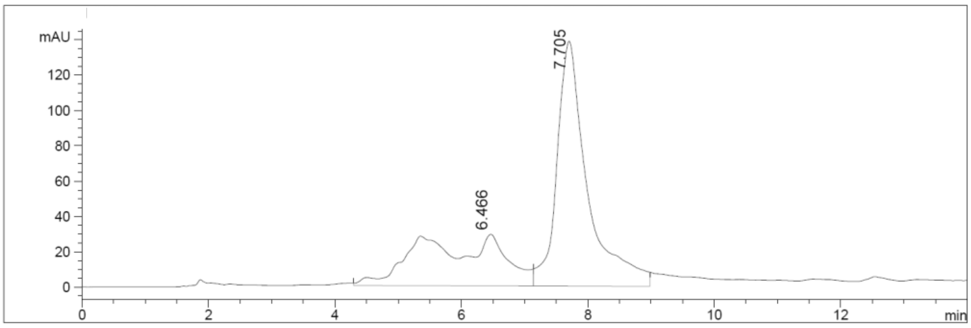

Peak list:

Ret. Time	Width min	Height	Area	Area %
6.466	1.490	29.220	2611.764	35.986
7.705	0.559	138.636	4645.936	64.014

MALDI-MS spectrum of isolated product **25h**

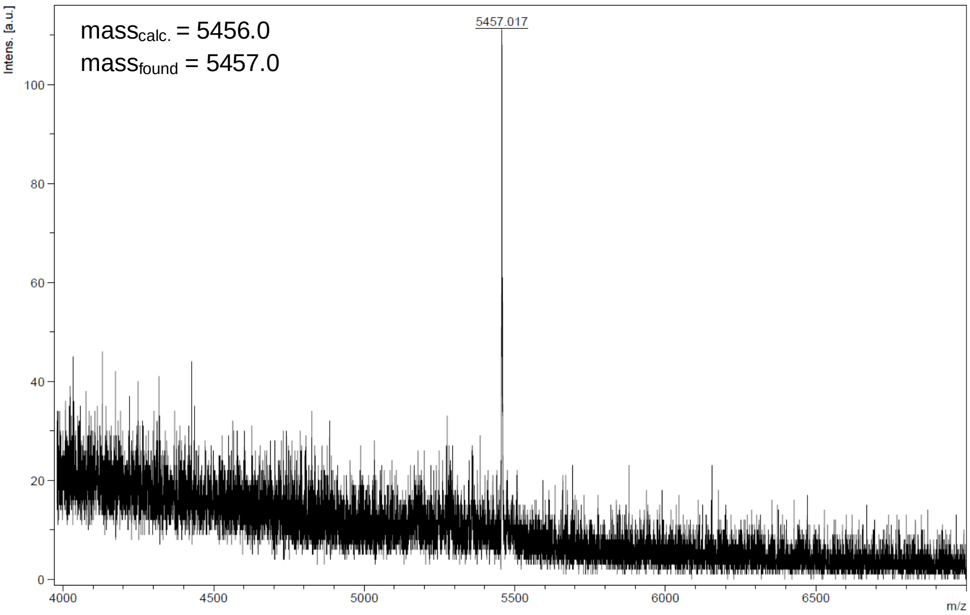

Brønsted acid-mediated reactions

Biginelli reaction

DNA conjugate 28: CPG-bound 16mer 7De-dATC-aldehyde conjugate **13** (Batch A) was reacted with urea **26,** and ethyl acetoacetate **27** according to RP-09.

HPLC trace of crude reaction mixture **28** (analytical RP-HPLC)

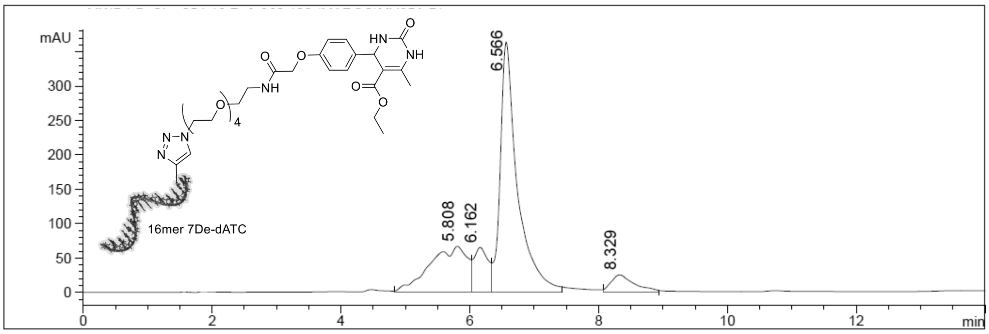

Peak list:

Ret. Time	Width min	Height	Area	Area %
5.808	0.674	66.684	2697.287	24.285
6.162	0.256	64.999	997.451	8.980
6.566	0.311	363.830	6779.593	61.040
8.329	0.422	25.004	632.552	5.695

HPLC trace of isolated product **28** (analytical RP-HPLC)

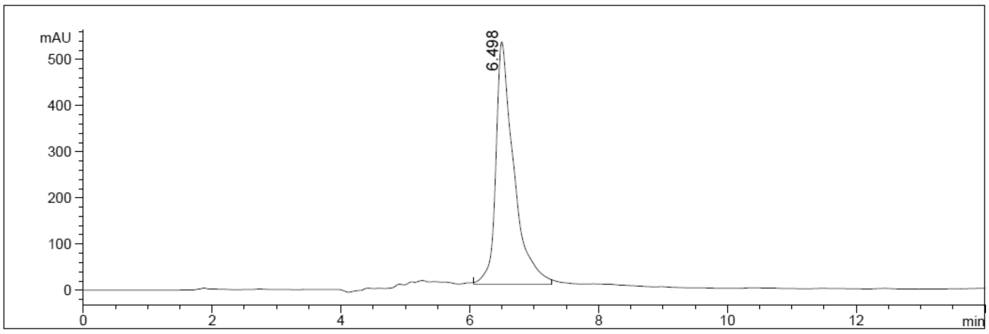

MALDI-MS spectrum of isolated product **28**

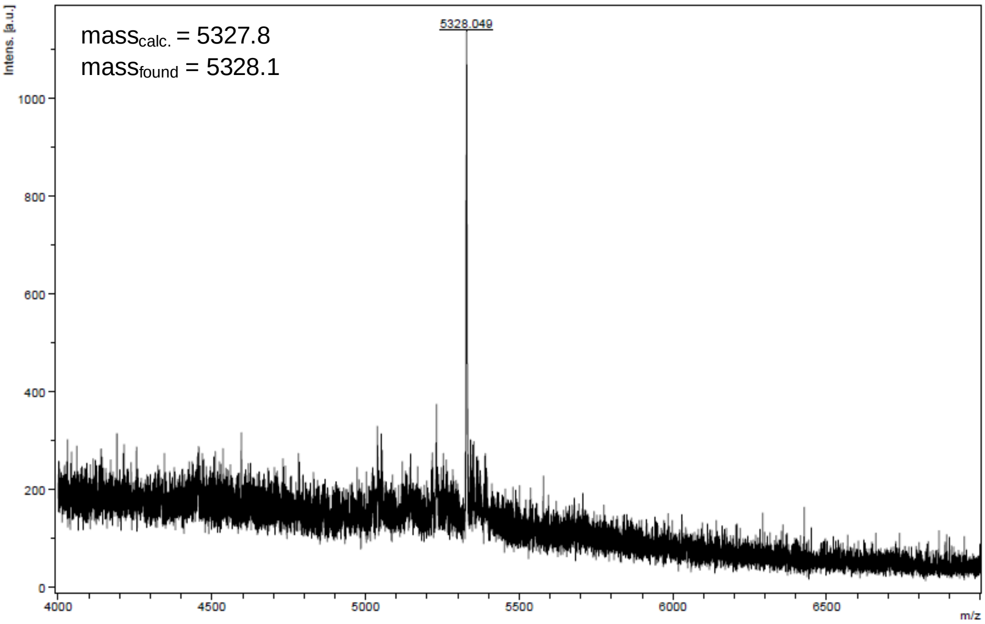

Povarov reaction

DNA conjugate 31: CPG-bound 16mer 7De-dATC-aldehyde conjugate **13** (Batch C) was reacted with aniline **29,** and *N*-Boc-2,3-dihydro-1*H*-pyrrole **30** according to RP-10.

HPLC trace of crude reaction mixture **31** (analytical RP-HPLC)

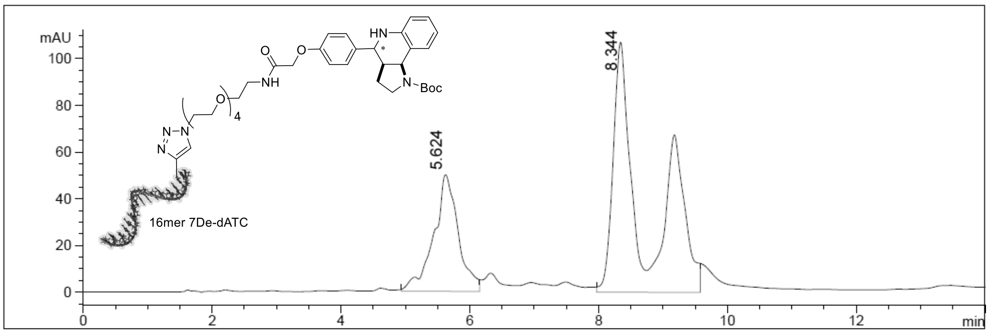

Peak list:

Ret. Time	Width min	Height	Area	Area %
5.624	0.442	49.956	1324.546	27.826
8.344	0.535	107.096	3435.576	72.174

HPLC trace of isolated product $\mathbf{31_{dia1}}$ (analytical RP-HPLC)

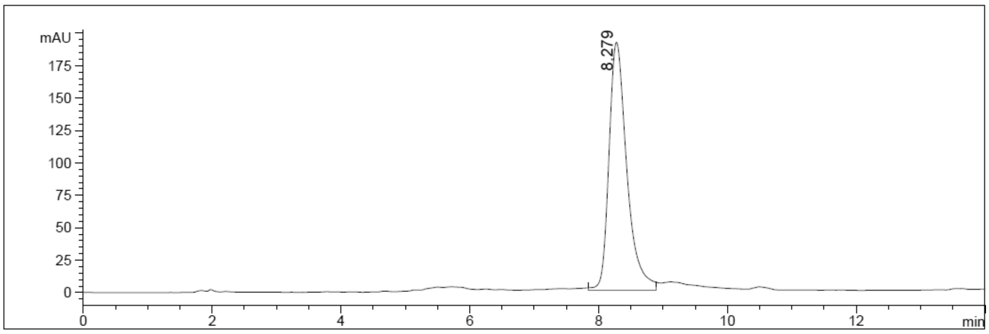

Peak list:

Ret. Time	Width min	Height	Area	Area %
8.279	0.319	190.998	3655.817	100.000

MALDI-MS spectrum of isolated product $\mathbf{31_{dia1}}$

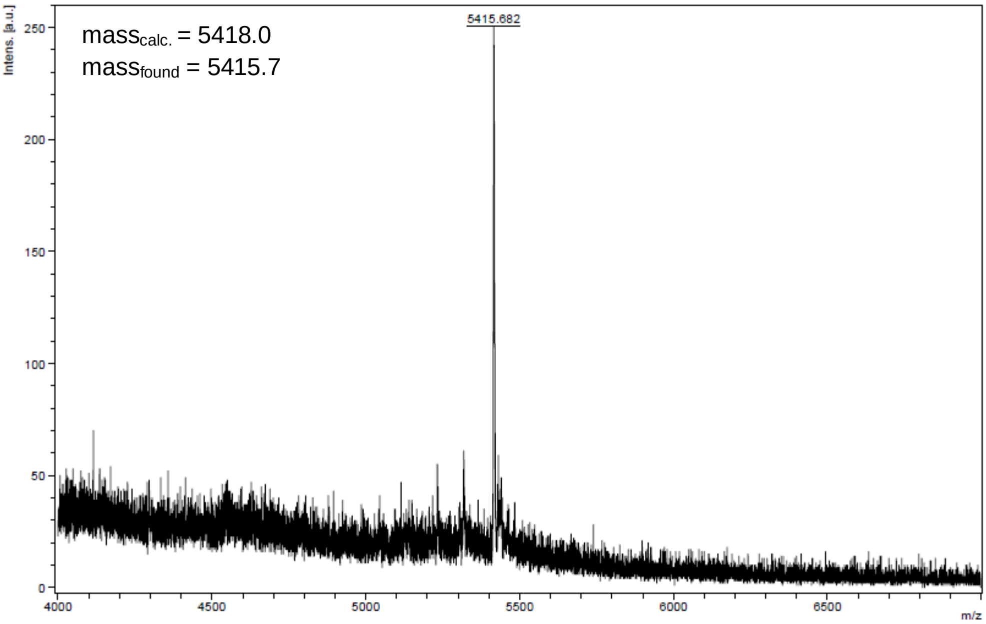

Boc cleavage on solid phase

DNA conjugate 32: CPG-bound 16mer 7De-dATC-Povarov conjugate **31** was Boc deprotected according to RP-11.

HPLC trace of crude reaction mixture **32** (analytical RP-HPLC)

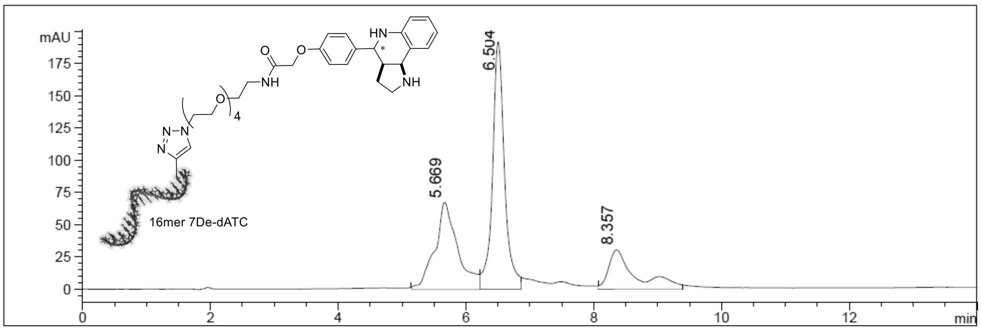

Peak list:

Ret. Time	Width min	Height	Area	Area %
5.669	0.416	67.588	1685.358	33.163
6.504	0.217	192.237	2503.403	49.260
8.357	0.489	30.466	893.277	17.577

HPLC trace of isolated product **32** (analytical RP-HPLC)

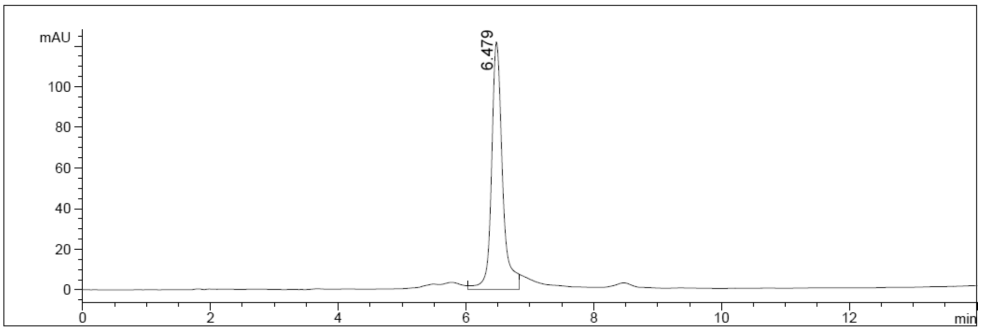

Peak list:

Ret. Time	Width min	Height	Area	Area %
6.479	0.183	122.075	1474.693	100.000

MALDI-MS spectrum of isolated product **32**

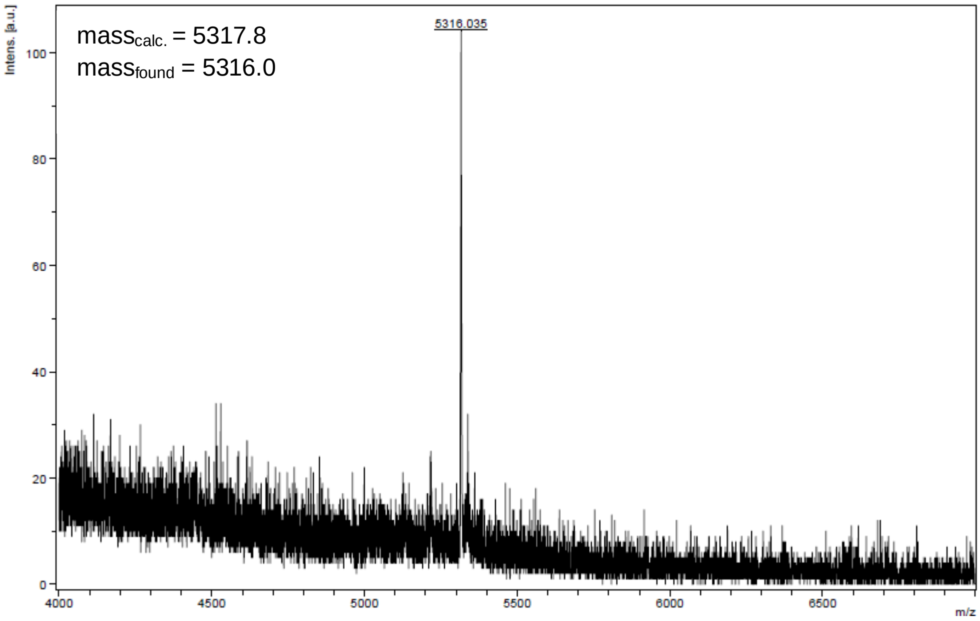

Pictet-Spengler reaction

Table S11 – Scope of TFA-mediated Pictet-Spenger reaction on CPG-bound 16mer 7De-dATC oligonucleotide-tryptophan conjugate **33** using different aldehydes **34**.[a]

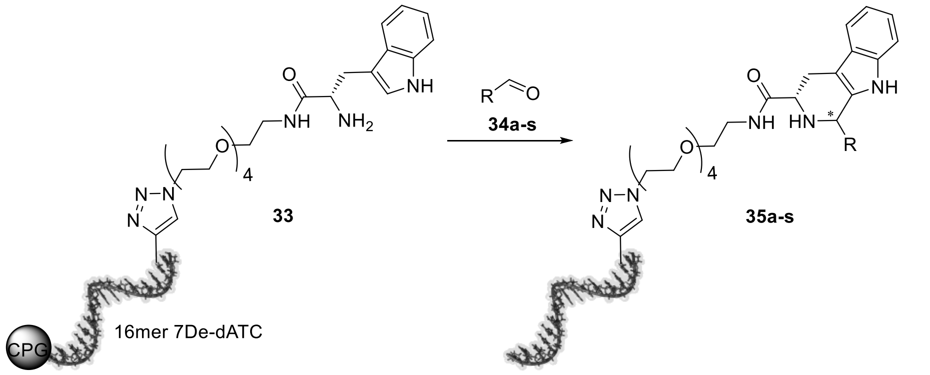

Entry	Product	Aldehyde	Conversion [%][b]	DNA degradation [%][c]	$Mass_{calc.}$ $Mass_{found}$[d]
1	**35a**	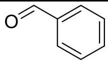 **34a**	93	<5	5285.8<br>5285.7
2	**35b**	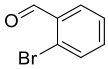 **34b**	>95	14	5364.7<br>5365.9
3	**35c**	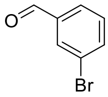 **34c**	>95	6	5364.7<br>5365.4
4	**35d**	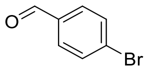 **34d**	>95	<5	5364.7<br>5364.7
5	**35e**	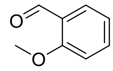 **34e**	>95	<5	5315.8<br>5315.8
6	**35f**	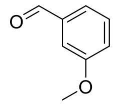 **34f**	>95	<5	5315.8<br>5315.7
7	**35g**	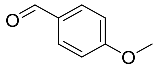 **34g**	>95	<5	5315.8<br>5317.0
8	**35h**	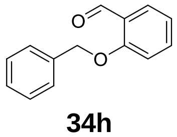 **34h**	64	<5	5391.9<br>5391.9
9	**35i**	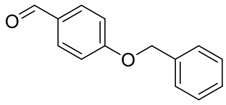 **34i**	>95	<5	5391.9<br>5392.3
10	**35j**	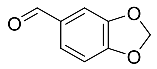 **34j**	>95	13	5329.8<br>5329.9

11	**35k**	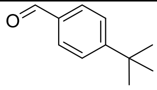 **34k**	>95	<5	5341.9<br>5341.8
12	**35l**	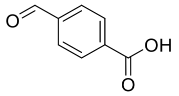 **34l**	>95	8	5329.8<br>5329.8
13	**35m**	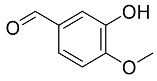 **34m**	>95	6	5331.8<br>5331.7
14	**35n**	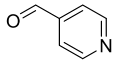 **34n**	85	<5	5286.8<br>5285.5
15	**35o**	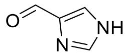 **34o**	>95	<5	5275.8<br>5272.4
16	**35p**	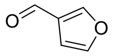 **34p**	85	<5	5275.8<br>5275.6
17	**35q**	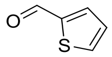 **34q**	>95	6	5291.8<br>5292.0
18	**35r**	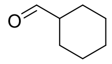 **34r**	>95	<5	5291.8<br>5291.8
19	**35s**	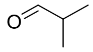 **34s**	>95	17	5251.8<br>5252.0

[a] CPG-bound oligonucleotide conjugate **33** (20 nmol) with aldehyde **34** (1500 equiv., 30 µmol) in 50 µL of 5% TFA in dichloromethane at ambient temperature for 20 h. DNA cleavage with AMA (30% aqueous ammonia / 40% aqueous methylamine, 1:1 (vol/vol)) at ambient temperature for 4 h. [b] Determined by analytical RP-HPLC analysis based on the ratios of **35** to **33**. [c] Determined by comparison of purities of the analytical RP-HPLC traces of starting material **33** and the crude reaction mixture **35**. [d] Measured by MALDI-MS. 16mer 7De-dATC = 5'-CT*C TCT TT7De-dA 7De-dACT 7De-dACC T-3'.

DNA conjugate 33: CPG-bound 16mer 7De-dATC-PEG(4)-NH_2 conjugate was reacted with *N*-Boc-tryptophan according to RP-04.

HPLC trace of crude reaction mixture **33** (analytical RP-HPLC)

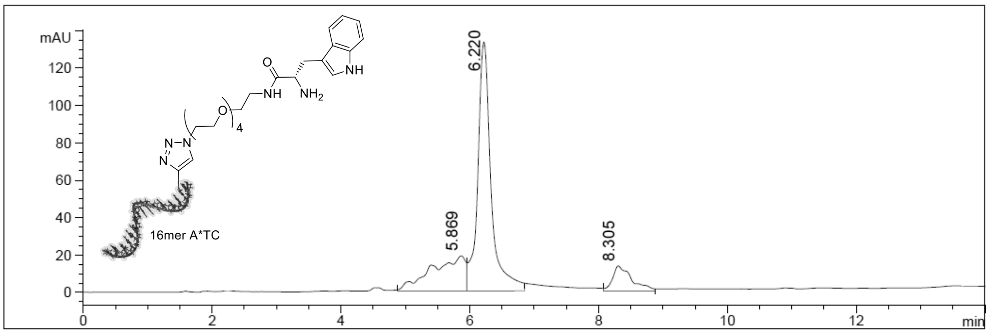

Peak list:

Ret. Time	Width min	Height	Area	Area %
5.869	0.602	18.840	680.488	23.719
6.220	0.240	133.165	1919.546	66.909
8.305	0.338	13.239	268.864	9.372

MALDI-MS spectrum of crude reaction mixture **33**

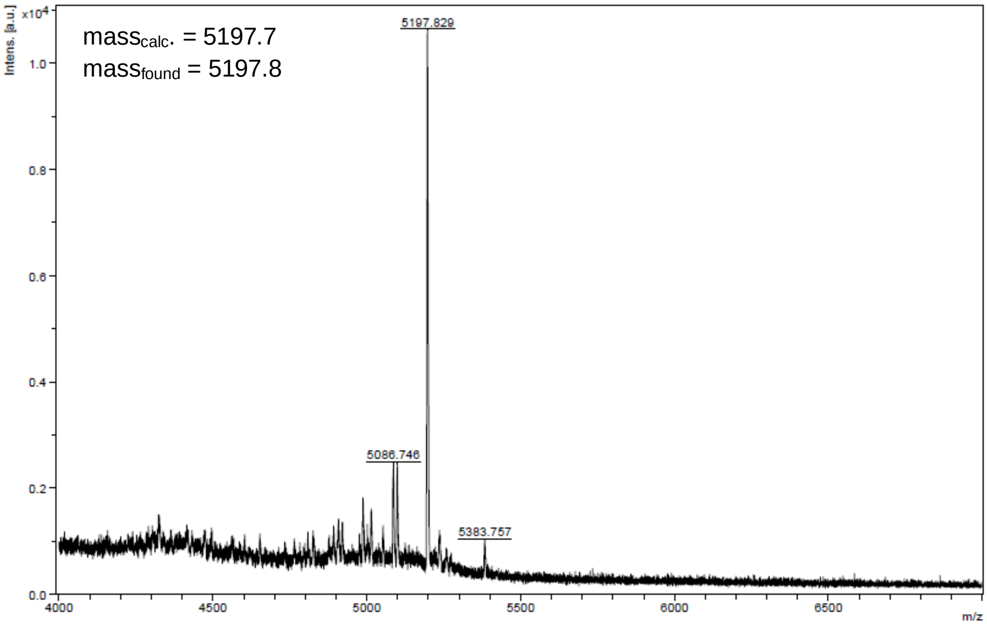

DNA conjugate 35a: CPG-bound 16mer 7De-dATC-tryptophan conjugate **33** was reacted with benzaldehyde **34a** according to RP-12.

HPLC trace of crude reaction mixture **35a** (analytical RP-HPLC)

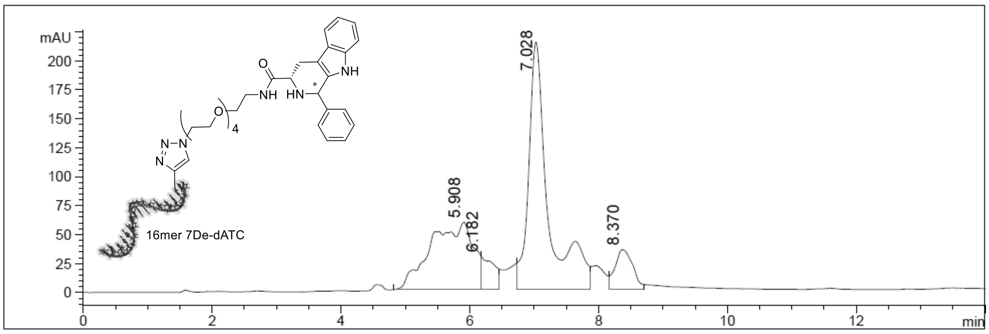

Peak list:

Ret. Time	Width min	Height	Area	Area %
5.908	0.771	57.620	2665.605	32.698
6.182	0.220	27.970	368.760	4.523
7.028	0.349	213.215	4468.176	54.810
8.370	0.319	33.927	649.569	7.968

HPLC trace of isolated product **35a** (analytical RP-HPLC)

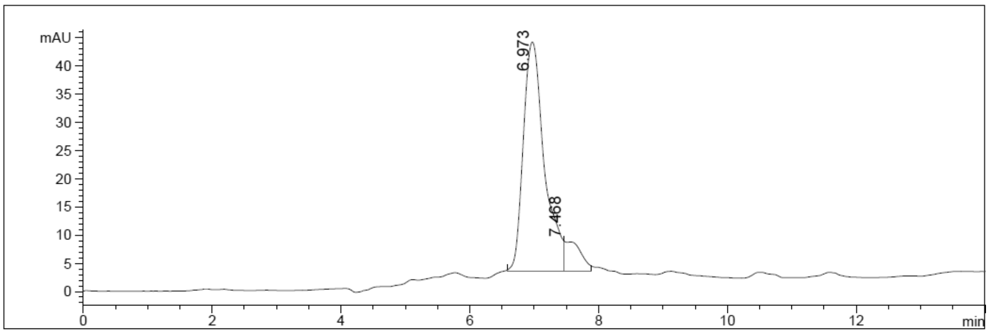

Peak list:

Ret. Time	Width min	Height	Area	Area %
6.973	0.380	40.656	926.310	90.897
7.468	0.259	5.367	92.768	9.103

MALDI-MS spectrum of isolated product **35a**

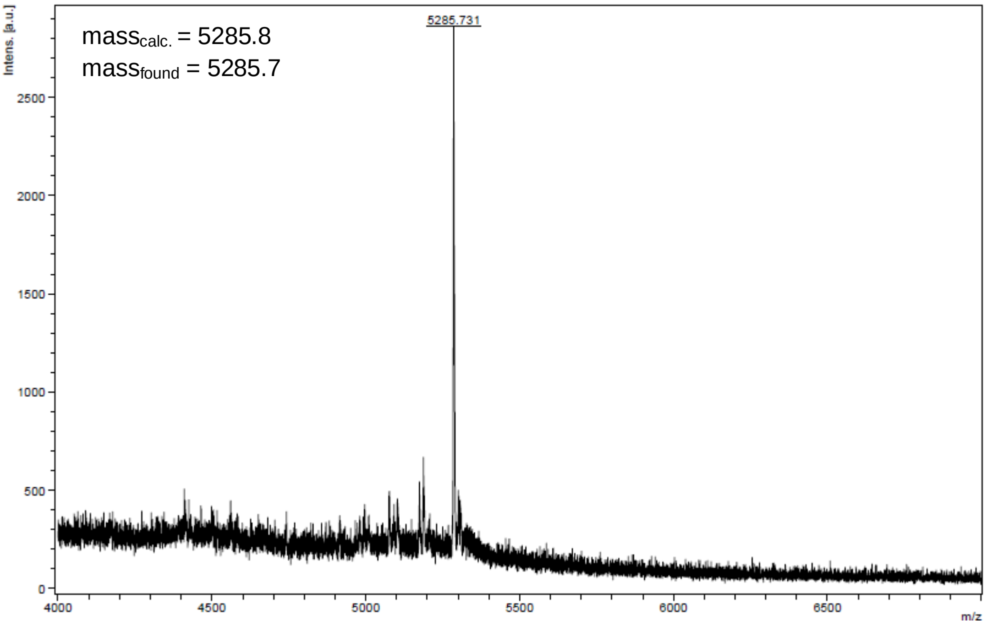

DNA conjugate 35b: CPG-bound 16mer 7De-dATC-tryptophan conjugate **33** was reacted with 2-bromoaldehyde **34b** according to RP-12.

HPLC trace of crude reaction mixture **35b** (analytical RP-HPLC)

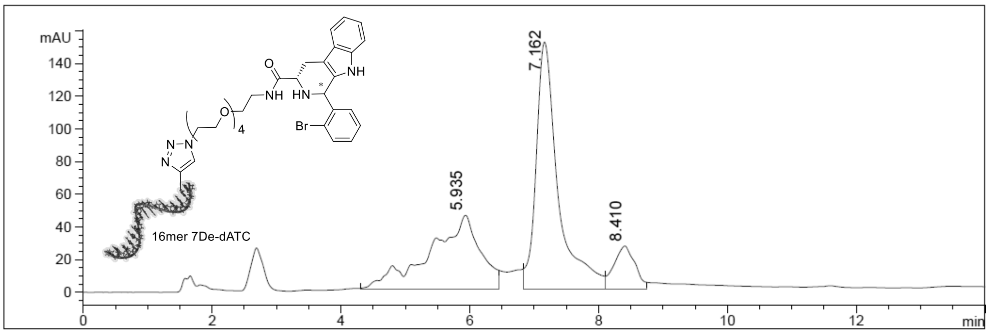

Peak list:

Ret. Time	Width min	Height	Area	Area %
5.935	0.909	45.212	2464.944	37.147
7.162	0.394	151.190	3573.426	53.852
8.410	0.376	26.451	597.312	9.002

HPLC trace of isolated product **35b** (analytical RP-HPLC)

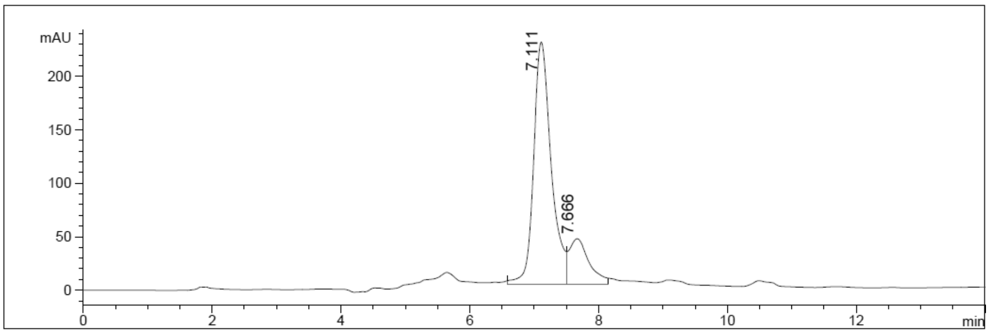

Peak list:

Ret. Time	Width min	Height	Area	Area %
7.111	0.318	226.843	4321.467	82.245
7.666	0.363	42.826	932.915	17.755

MALDI-MS spectrum of isolated product **35b**

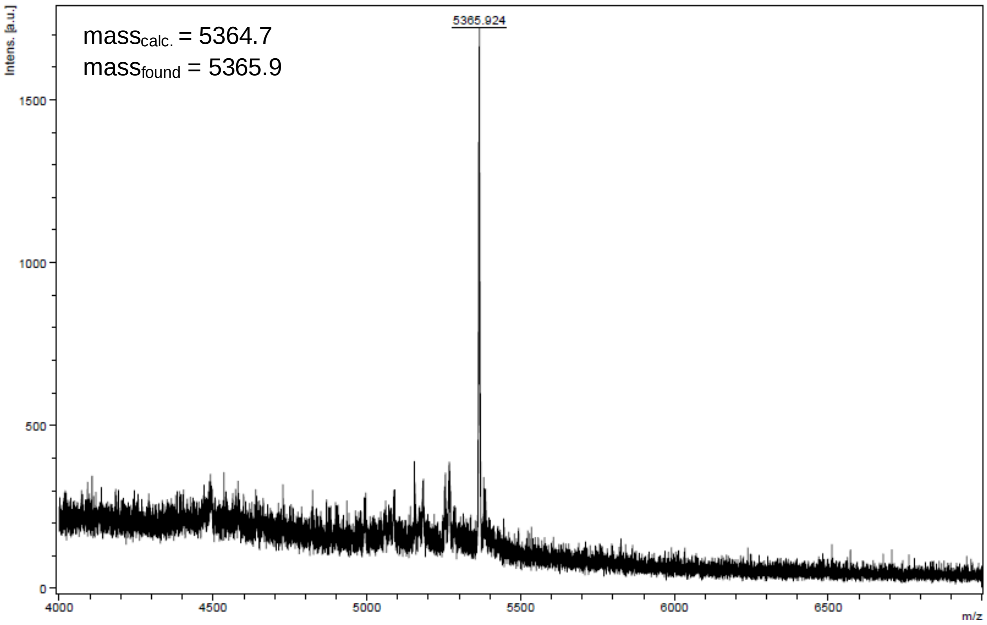

DNA conjugate 35c: CPG-bound 16mer 7De-dATC-tryptophan conjugate **33** was reacted with 3-bromoaldehyde **34c** according to RP-12.

HPLC trace of crude reaction mixture **35c** (analytical RP-HPLC)

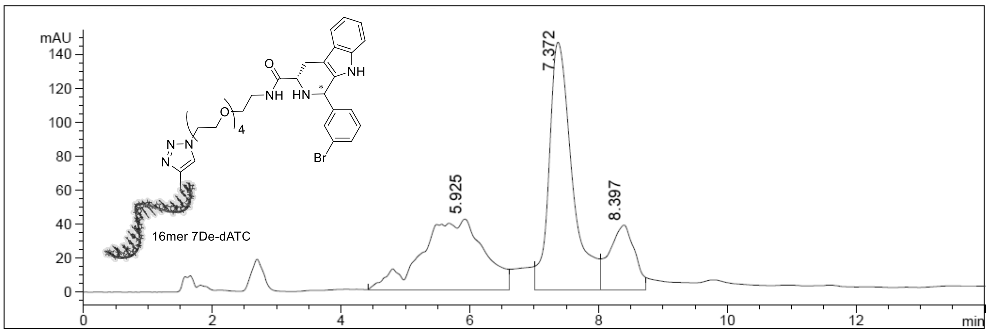

Peak list:

Ret. Time	Width min	Height	Area	Area %
5.925	1.129	41.612	2820.050	37.634
7.372	0.415	145.760	3632.841	48.481
8.397	0.455	38.091	1040.455	13.885

HPLC trace of isolated product **35c** (analytical RP-HPLC)

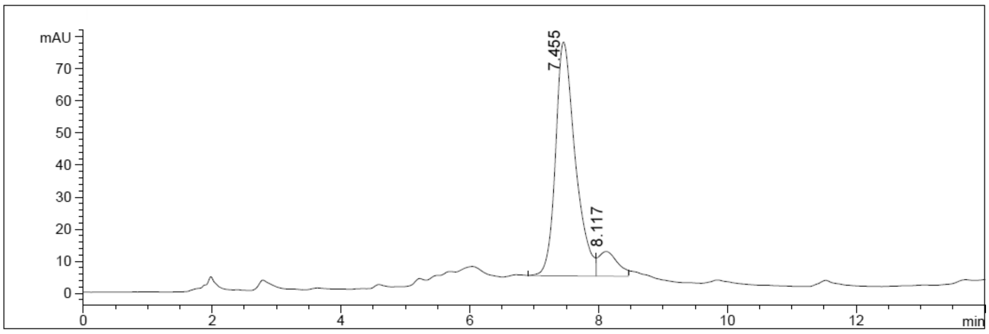

Peak list:

Ret. Time	Width min	Height	Area	Area %
7.455	0.354	72.904	1549.575	90.825
8.117	0.340	7.669	156.542	9.175

MALDI-MS spectrum of isolated product **35c**

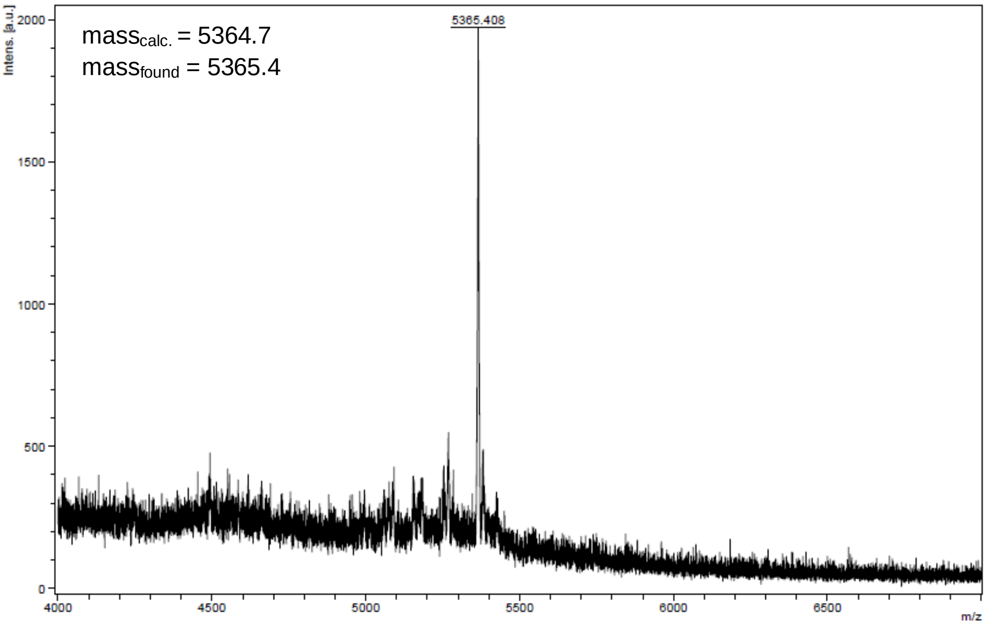

DNA conjugate 35d: CPG-bound 16mer 7De-dATC-tryptophan conjugate **33** was reacted with 4-bromoaldehyde **34d** according to RP-12.

HPLC trace of crude reaction mixture **35d** (analytical RP-HPLC)

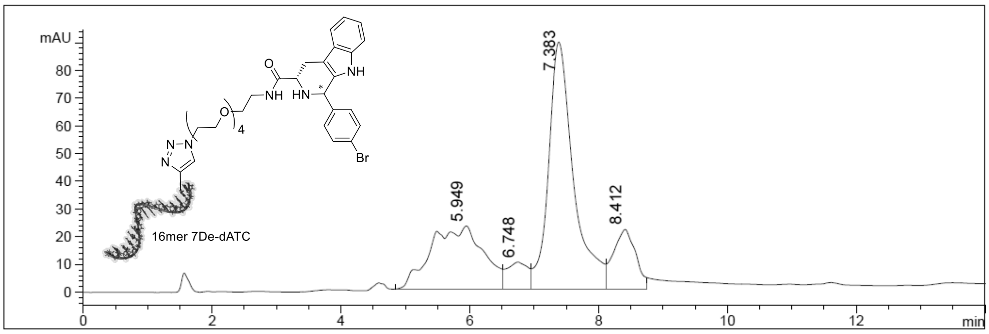


```
Peak list:

Ret. Time   Width min   Height      Area        Area %
------------------------------------------------------------
5.949       0.949       22.771      1296.702    29.250
6.748       0.379       9.777       222.584     5.021
7.383       0.448       89.136      2396.292    54.054
8.412       0.401       21.535      517.543     11.674
------------------------------------------------------------
```

HPLC trace of isolated product **35d** (analytical RP-HPLC)

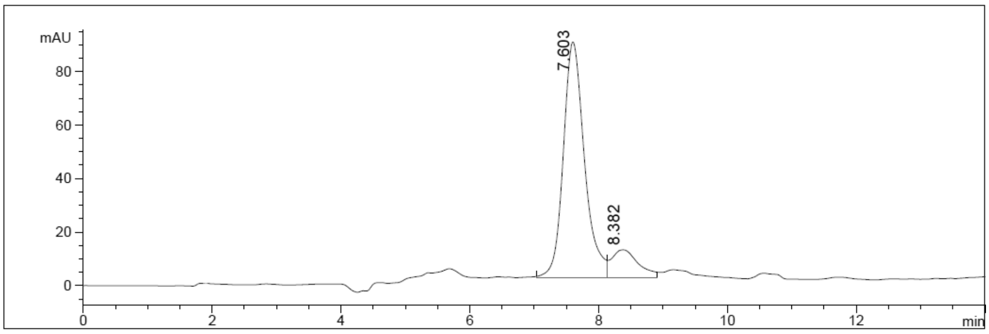


```
Peak list:

Ret. Time   Width min   Height      Area        Area %
------------------------------------------------------------
7.603       0.382       87.983      2018.067    87.132
8.382       0.479       10.370      298.037     12.868
------------------------------------------------------------
```

MALDI-MS spectrum of isolated product **35d**

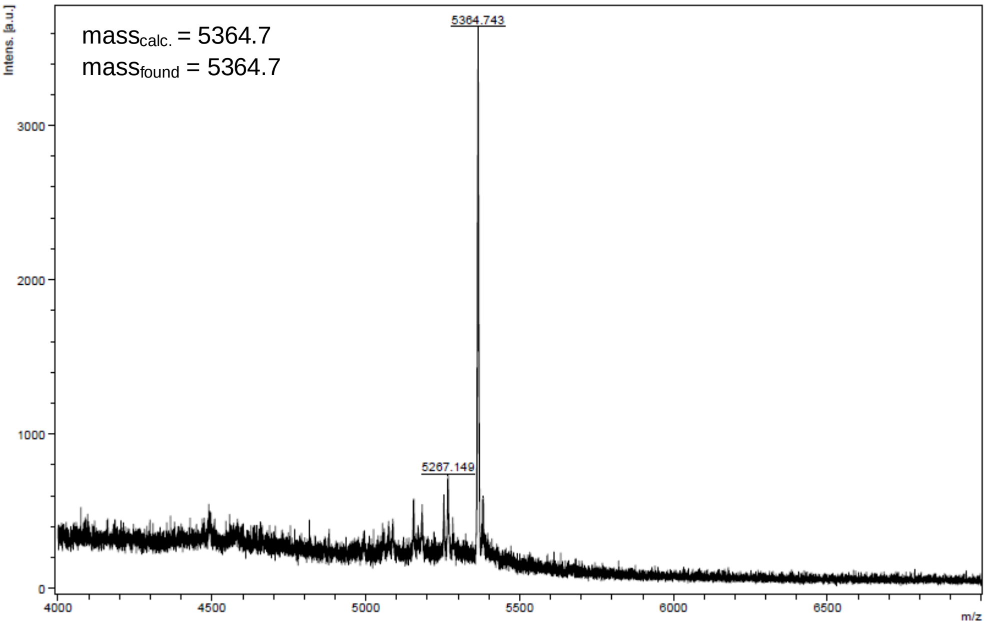

DNA conjugate 35e: CPG-bound 16mer 7De-dATC-tryptophan conjugate **33** was reacted with *o*-anisaldehyde **34e** according to RP-12.

HPLC trace of crude reaction mixture **35e** (analytical RP-HPLC)

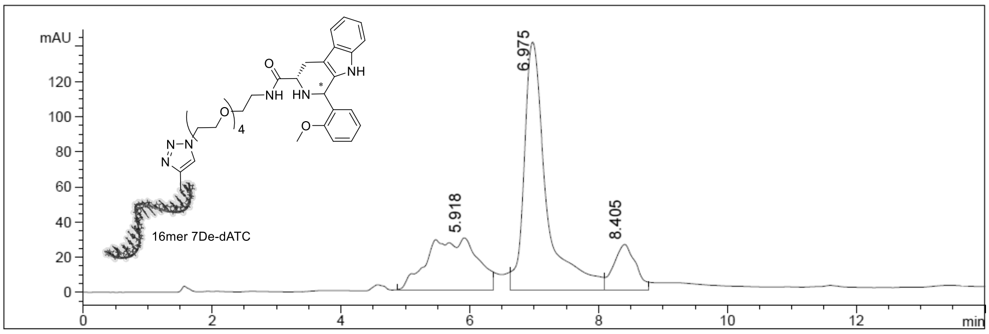

Peak list:

Ret. Time	Width min	Height	Area	Area %
5.918	0.907	29.841	1623.720	28.751
6.975	0.402	141.264	3405.354	60.298
8.405	0.395	26.106	618.482	10.951

HPLC trace of isolated product **35e** (analytical RP-HPLC)

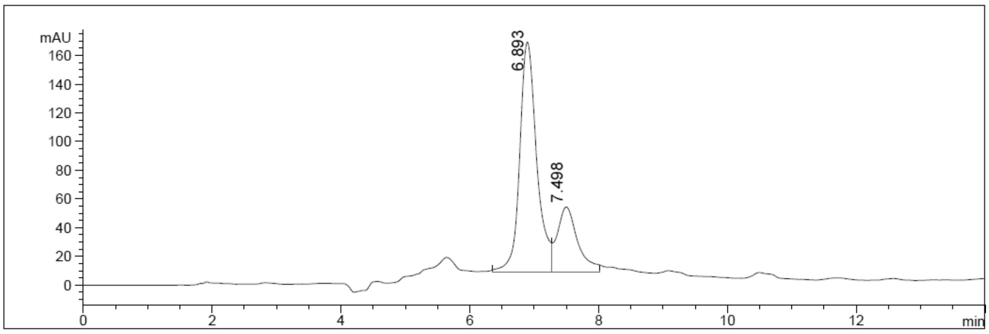

Peak list:

Ret. Time	Width min	Height	Area	Area %
6.893	0.308	160.389	2961.800	74.749
7.498	0.367	45.409	1000.505	25.251

MALDI-MS spectrum of isolated product **35e**

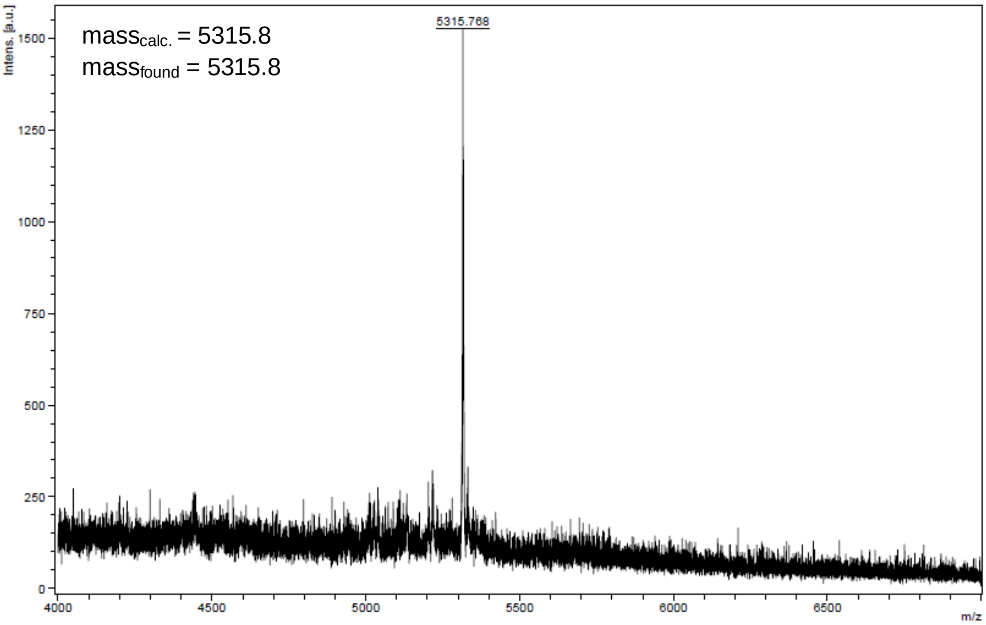

DNA conjugate 35f: CPG-bound 16mer 7De-dATC-tryptophan conjugate **33** was reacted with *m*-anisaldehyde **34f** according to RP-12.

HPLC trace of crude reaction mixture **35f** (analytical RP-HPLC)

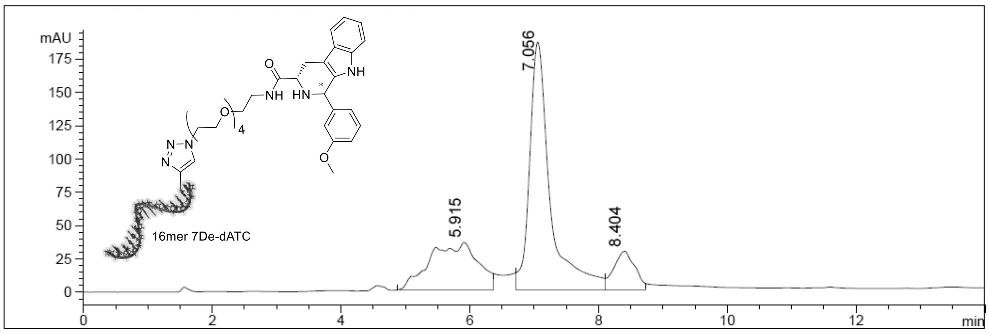

Peak list:

Ret. Time	Width min	Height	Area	Area %
5.915	0.869	35.656	1858.501	28.008
7.056	0.368	186.098	4111.922	61.967
8.404	0.380	29.173	665.240	10.025

HPLC trace of isolated product **35f** (analytical RP-HPLC)

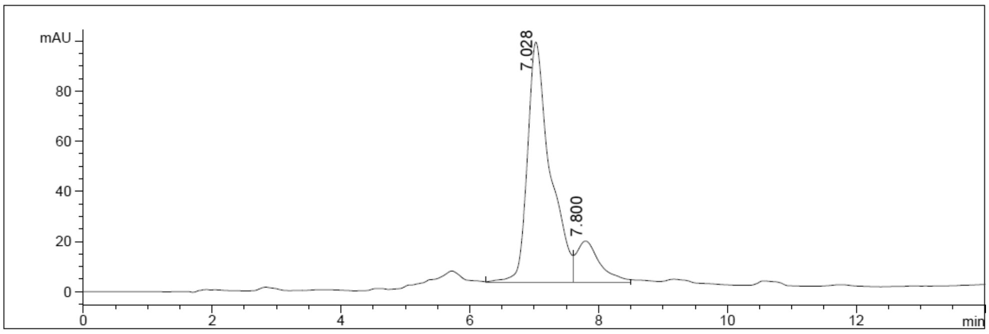

Peak list:

Ret. Time	Width min	Height	Area	Area %
7.028	0.409	95.715	2346.867	85.117
7.800	0.416	16.427	410.342	14.883

MALDI-MS spectrum of isolated product **35f**

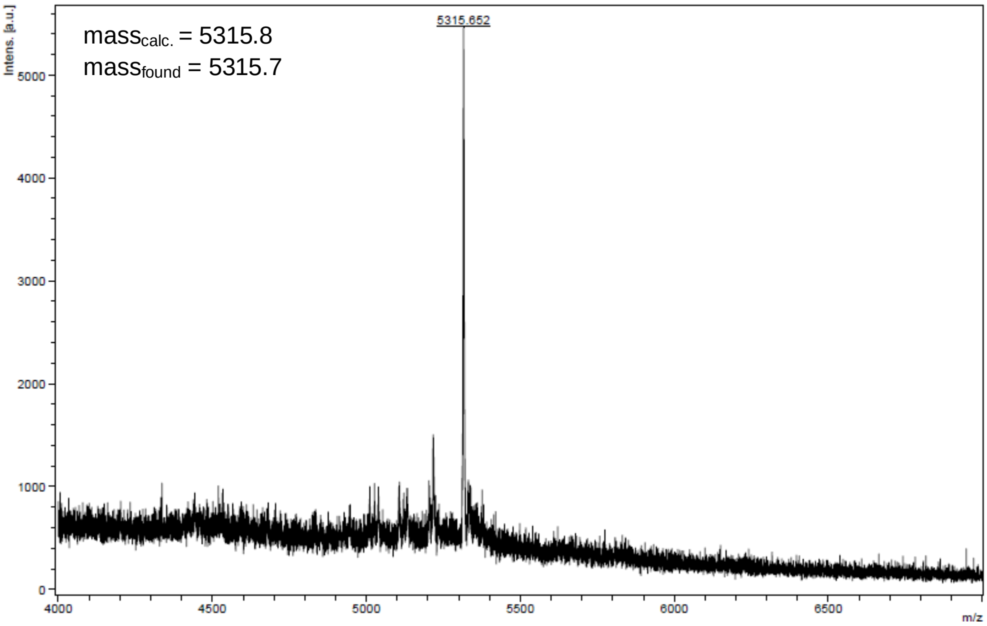

DNA conjugate 35g: CPG-bound 16mer 7De-dATC-tryptophan conjugate **33** was reacted with *p*-anisaldehyde **34g** according to RP-12.

HPLC trace of crude reaction mixture **35g** (analytical RP-HPLC)

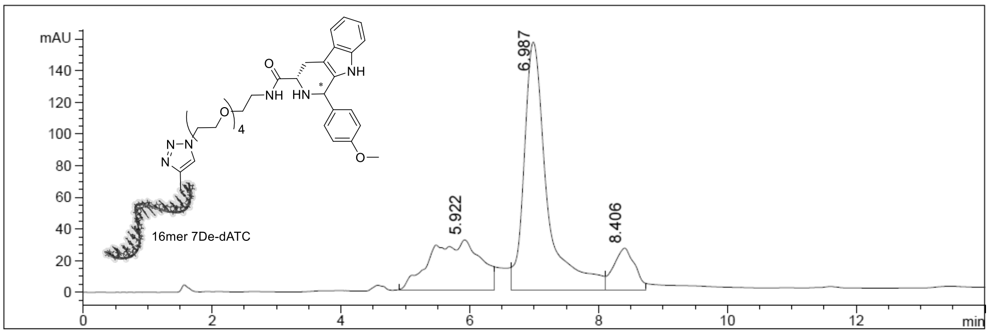


```
Peak list:

Ret. Time   Width min   Height      Area        Area %
--------------------------------------------------------
5.922       0.906       31.631      1718.760    27.690
6.987       0.414       156.621     3886.079    62.608
8.406       0.379       26.458      602.208     9.702
--------------------------------------------------------
```

HPLC trace of isolated product **35g** (analytical RP-HPLC)

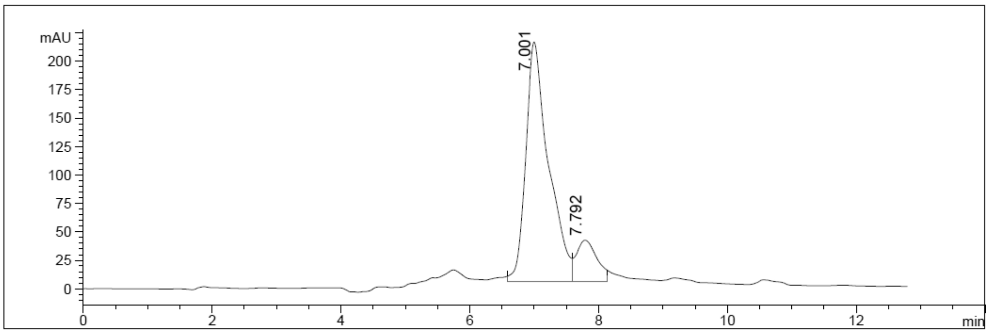


```
Peak list:

Ret. Time   Width min   Height      Area        Area %
--------------------------------------------------------
7.001       0.410       210.168     5164.978    86.600
7.792       0.370       36.031      799.209     13.400
--------------------------------------------------------
```

MALDI-MS spectrum of isolated product **35g**

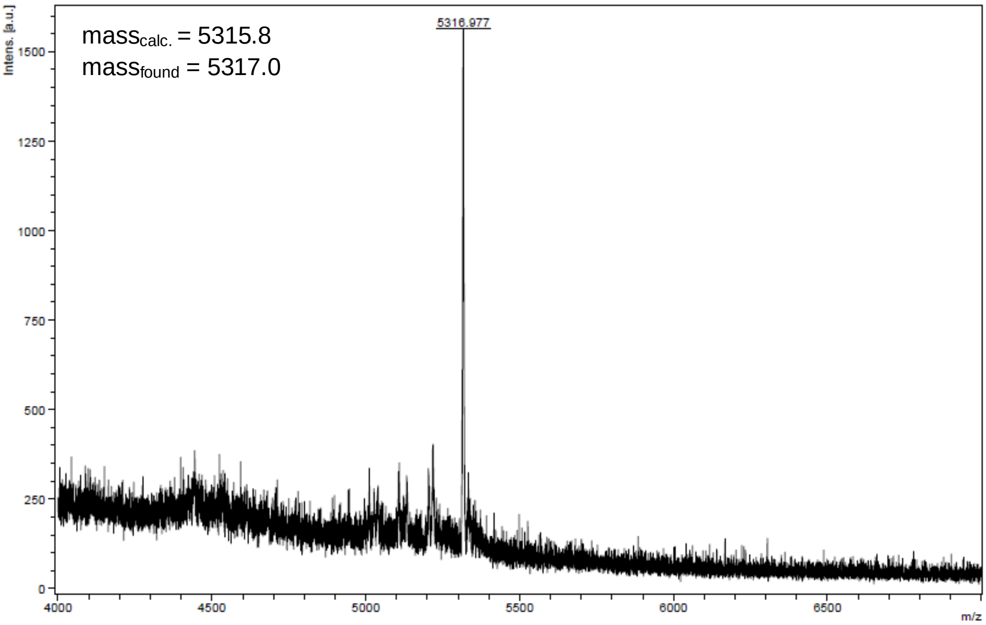

DNA conjugate 35h: CPG-bound 16mer 7De-dATC-tryptophan conjugate **33** was reacted with 2-benzyloxybenzaldehyde **34h** according to RP-12.

HPLC trace of crude reaction mixture **35h** (analytical RP-HPLC)

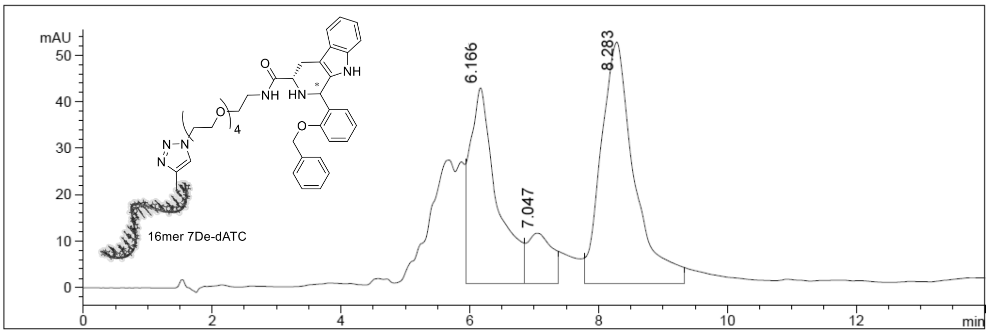

Peak list:

Ret. Time	Width min	Height	Area	Area %
6.166	0.478	42.165	1210.202	36.034
7.047	0.450	10.898	294.182	8.759
8.283	0.594	52.004	1854.158	55.207

HPLC trace of isolated product **35h** (analytical RP-HPLC)

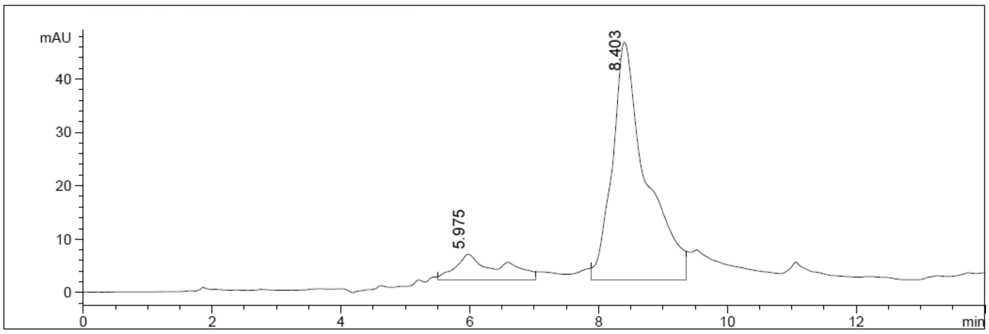

Peak list:

Ret. Time	Width min	Height	Area	Area %
5.975	0.802	4.851	233.293	12.609
8.403	0.605	44.545	1616.887	87.391

MALDI-MS spectrum of isolated product **35h**

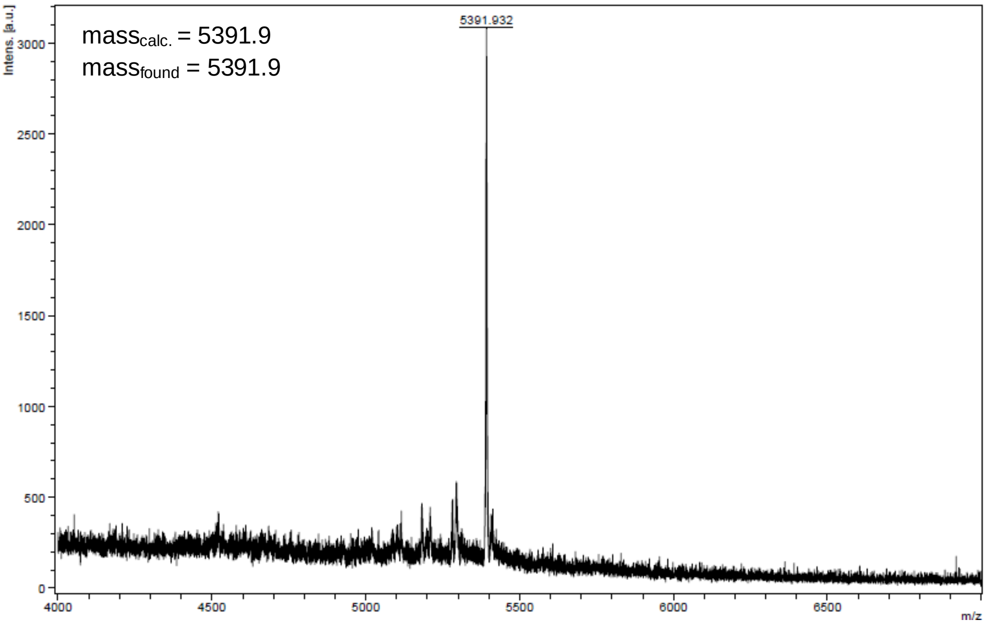

DNA conjugate 35i: CPG-bound 16mer 7De-dATC-tryptophan conjugate **33** was reacted with 4-benzyloxybenzaldehyde **34i** according to RP-12.

HPLC trace of crude reaction mixture **35i** (analytical RP-HPLC)

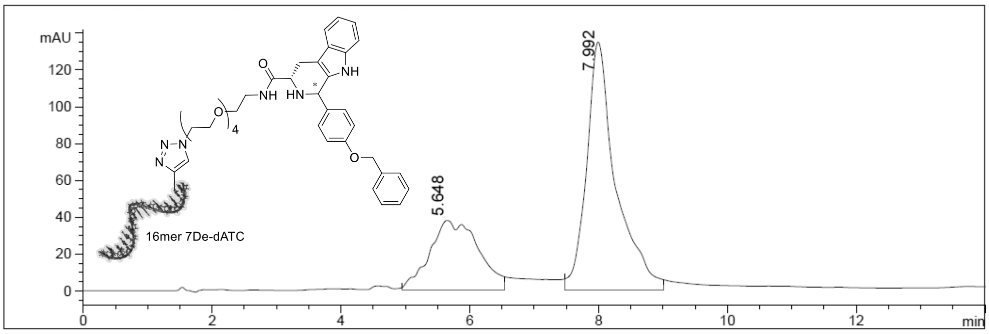

Peak list:

Ret. Time	Width min	Height	Area	Area %
5.648	0.875	37.585	1973.519	31.806
7.992	0.524	134.486	4231.256	68.194

HPLC trace of isolated product **35i** (analytical RP-HPLC)

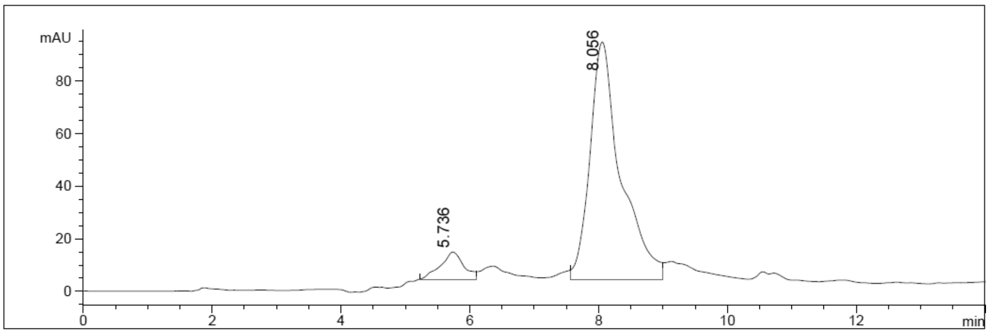

Peak list:

Ret. Time	Width min	Height	Area	Area %
5.736	0.422	10.488	265.408	8.110
8.056	0.555	90.371	3007.144	91.890

MALDI-MS spectrum of isolated product **35i**

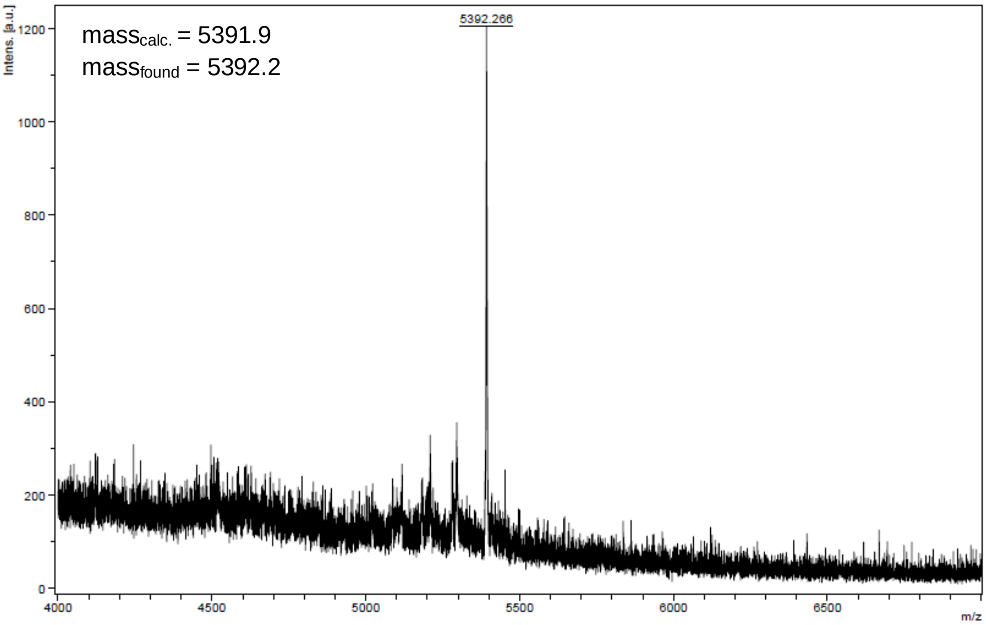

DNA conjugate 35j: CPG-bound 16mer 7De-dATC-tryptophan conjugate **33** was reacted with piperonal **34j** according to RP-12.

HPLC trace of crude reaction mixture **35j** (analytical RP-HPLC)

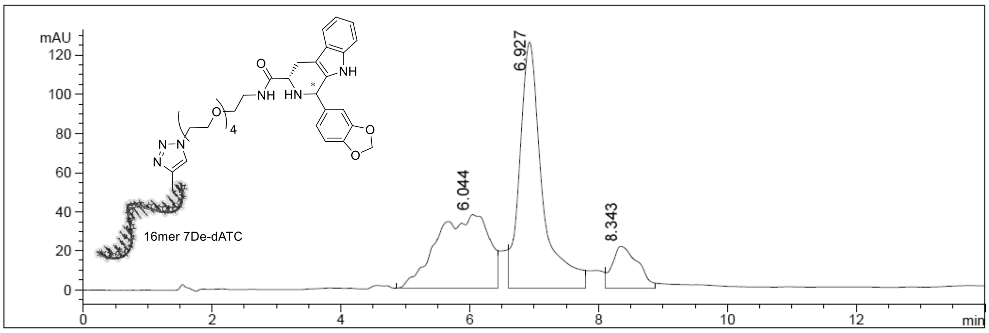

Peak list:

Ret. Time	Width min	Height	Area	Area %
6.044	0.944	37.948	2149.829	36.999
6.927	0.407	125.768	3069.250	52.823
8.343	0.463	21.293	591.384	10.178

HPLC trace of isolated product **35j** (analytical RP-HPLC)

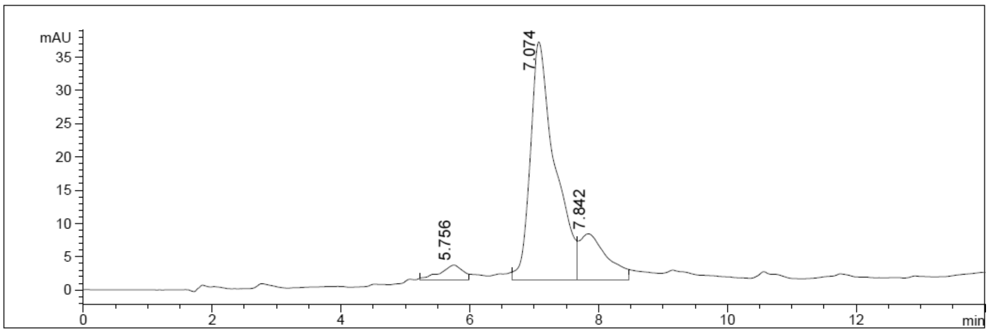

Peak list:

Ret. Time	Width min	Height	Area	Area %
5.756	0.410	2.241	55.120	4.545
7.074	0.444	35.782	952.570	78.541
7.842	0.493	6.942	205.137	16.914

MALDI-MS spectrum of isolated product **35j**

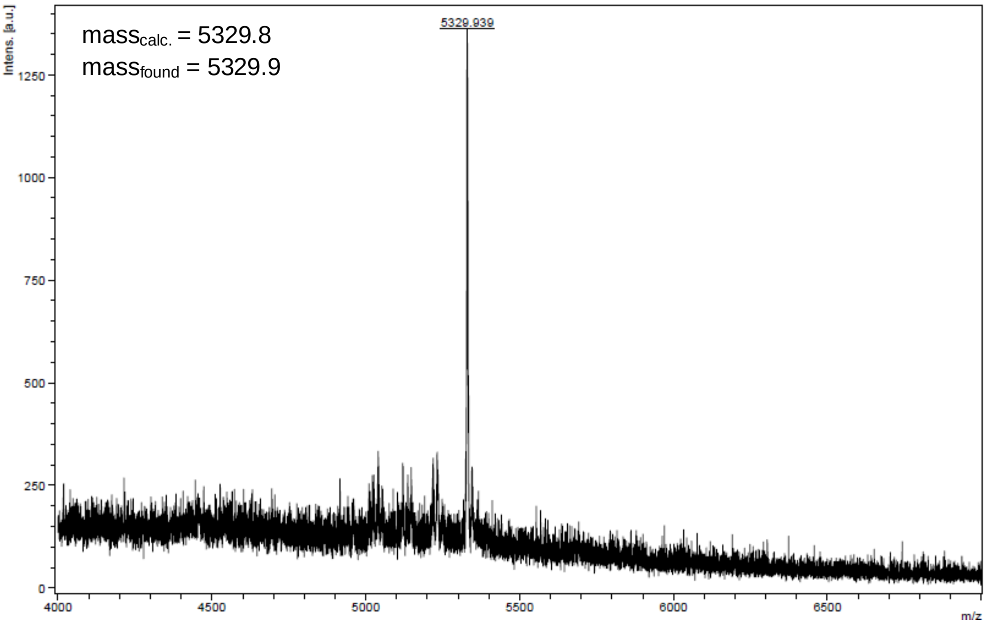

DNA conjugate 35k: CPG-bound 16mer 7De-dATC-tryptophan conjugate **33** was reacted with 4-*tert*-butylbenzaldehyde **34k** according to RP-12.

HPLC trace of crude reaction mixture **35k** (analytical RP-HPLC)

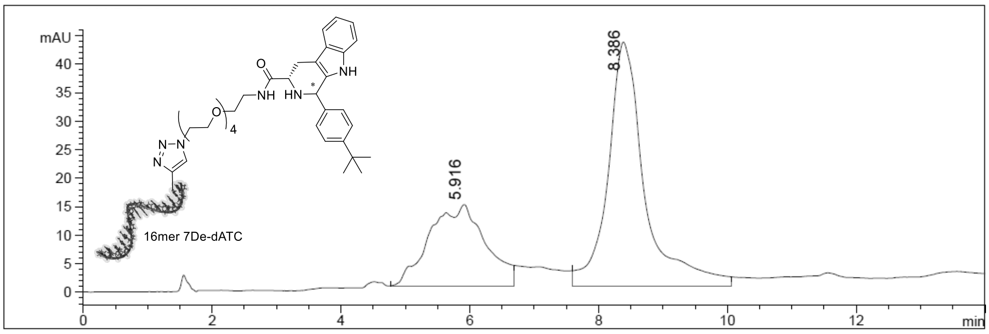

Peak list:

Ret. Time	Width min	Height	Area	Area %
5.916	1.004	14.279	860.122	33.682
8.386	0.660	42.766	1693.552	66.318

HPLC trace of isolated product **35k** (analytical RP-HPLC)

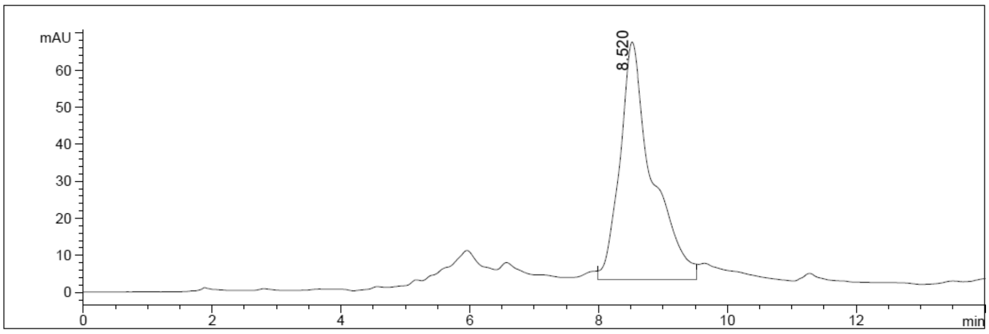

Peak list:

Ret. Time	Width min	Height	Area	Area %
8.520	0.578	64.121	2225.209	100.000

MALDI-MS spectrum of isolated product **35k**

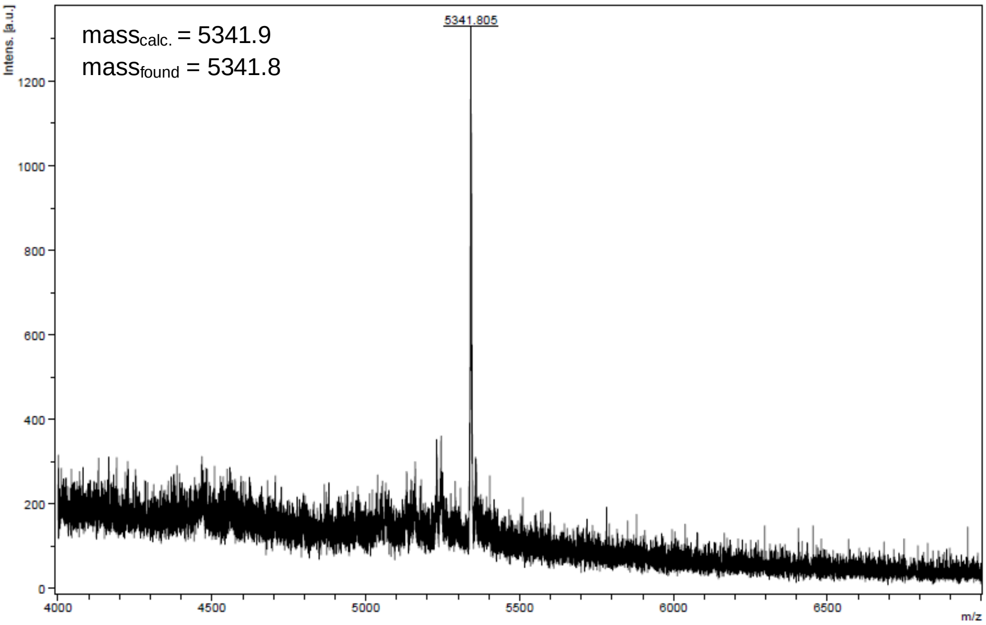

DNA conjugate 35l: CPG-bound 16mer 7De-dATC-tryptophan conjugate **33** was reacted with 4-formylbenzoic acid **34l** according to RP-12.

HPLC trace of crude reaction mixture **35l** (analytical RP-HPLC)

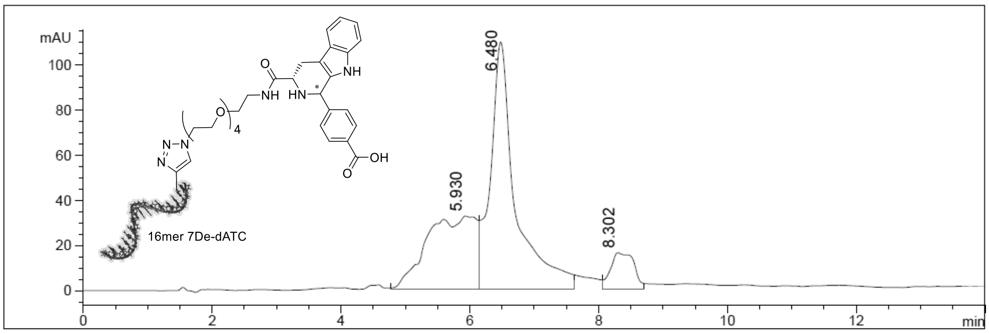

Peak list:

Ret. Time	Width min	Height	Area	Area %
5.930	0.891	32.175	1719.534	34.128
6.480	0.445	109.273	2914.885	57.852
8.302	0.421	15.984	404.072	8.020

HPLC trace of isolated product **35l** (analytical RP-HPLC)

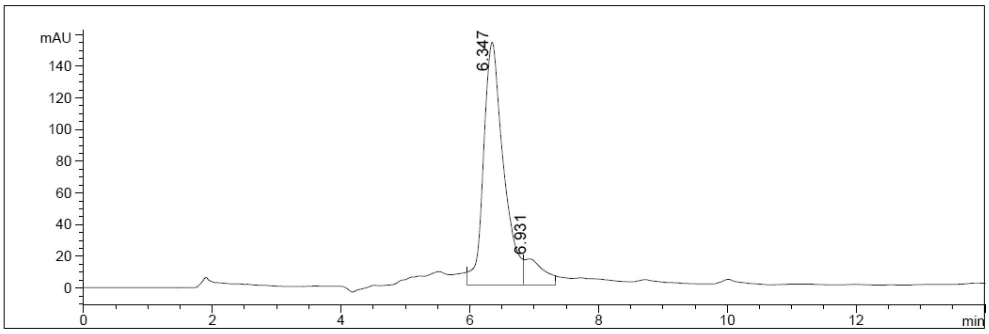

MALDI-MS spectrum of isolated product **35l**

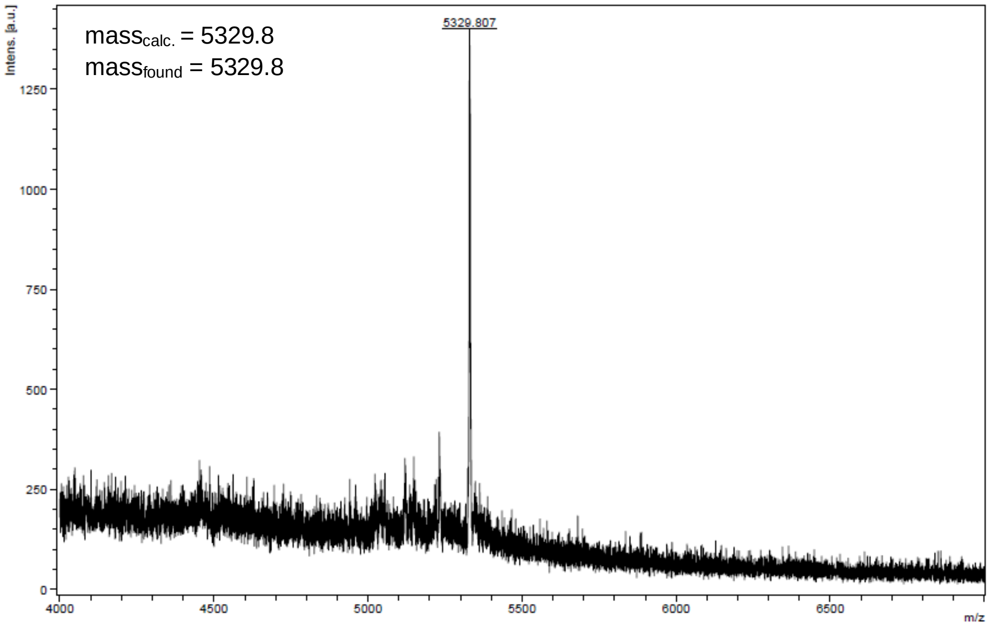

DNA conjugate 35m: CPG-bound 16mer 7De-dATC-tryptophan conjugate **33** was reacted with isovanilline **34m** according to RP-12.

HPLC trace of crude reaction mixture **35m** (analytical RP-HPLC)

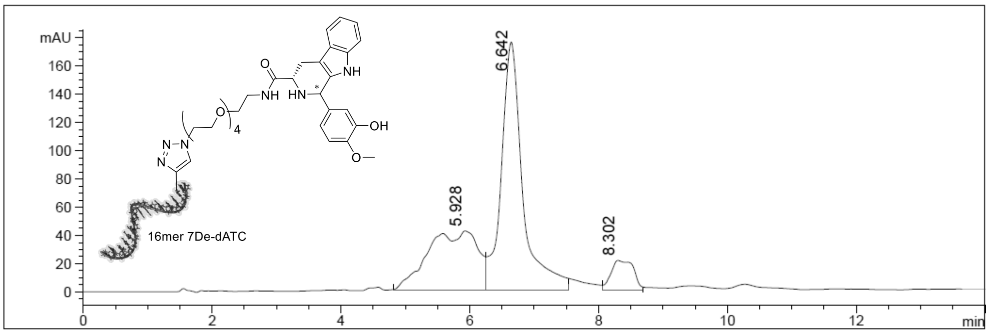


```
Peak list:

Ret. Time   Width min   Height     Area       Area %
--------------------------------------------------------
5.928       0.902       41.892     2267.198   33.170
6.642       0.385       175.706    4060.763   59.410
8.302       0.404       20.913     507.215    7.421
--------------------------------------------------------
```

HPLC trace of isolated product **35m** (analytical RP-HPLC)

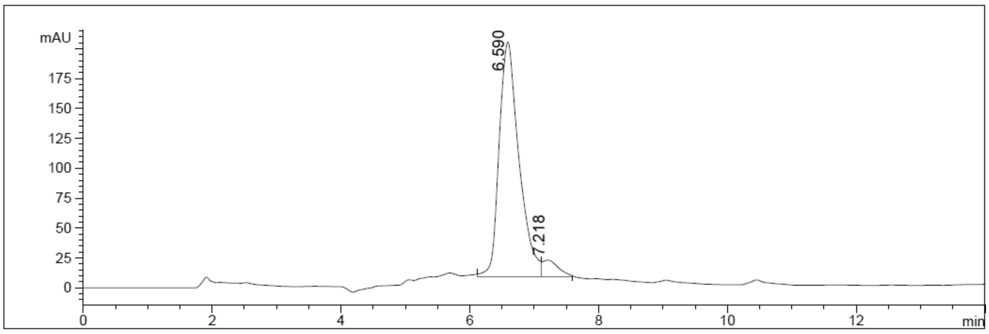

MALDI-MS spectrum of isolated product **35m**

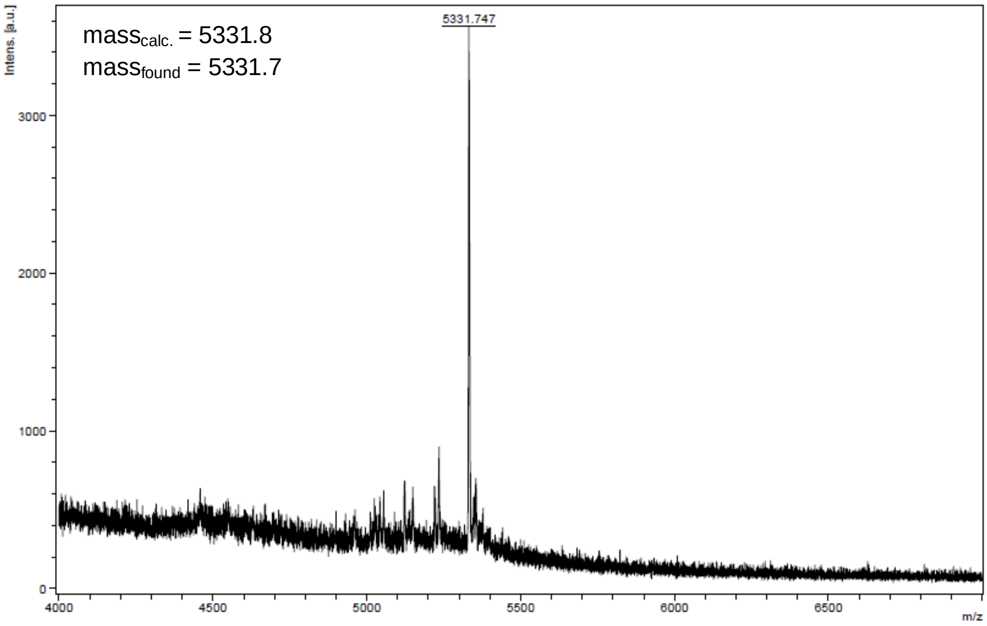

DNA conjugate 35n: CPG-bound 16mer 7De-dATC-tryptophan conjugate **33** was reacted with isonicotinaldehyde **34n** according to RP-12.

HPLC trace of crude reaction mixture **35n** (analytical RP-HPLC)

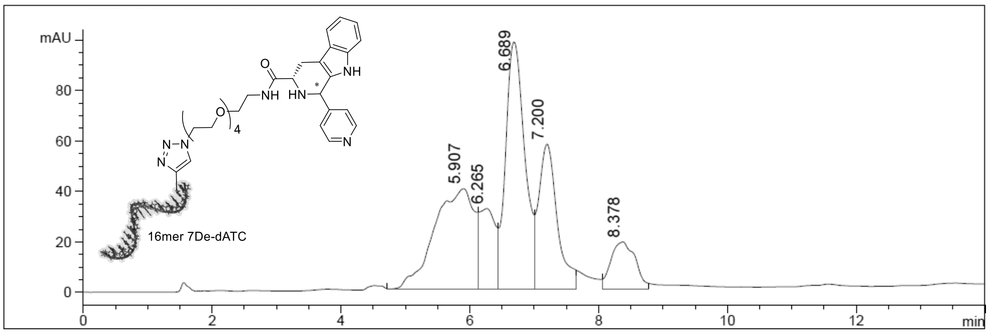

Peak list:

Ret. Time	Width min	Height	Area	Area %
5.907	0.710	39.872	1698.023	28.593
6.265	0.293	32.029	562.468	9.472
6.689	0.345	97.994	2027.659	34.144
7.200	0.335	57.514	1154.942	19.448
8.378	0.438	18.843	495.423	8.343

HPLC trace of isolated product **35n** (analytical RP-HPLC)

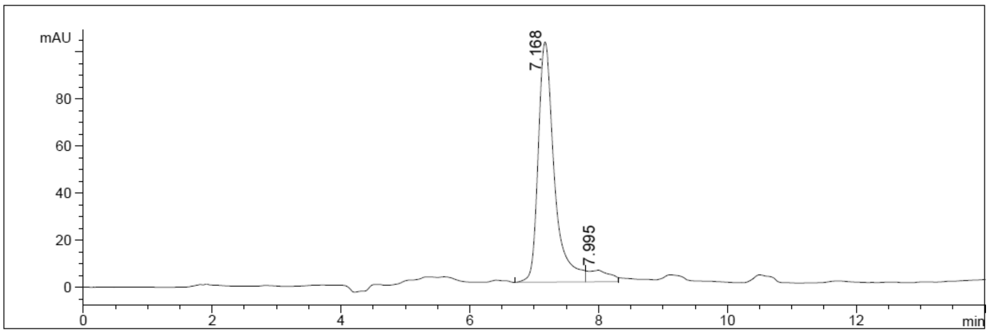

MALDI-MS spectrum of isolated product **35n**

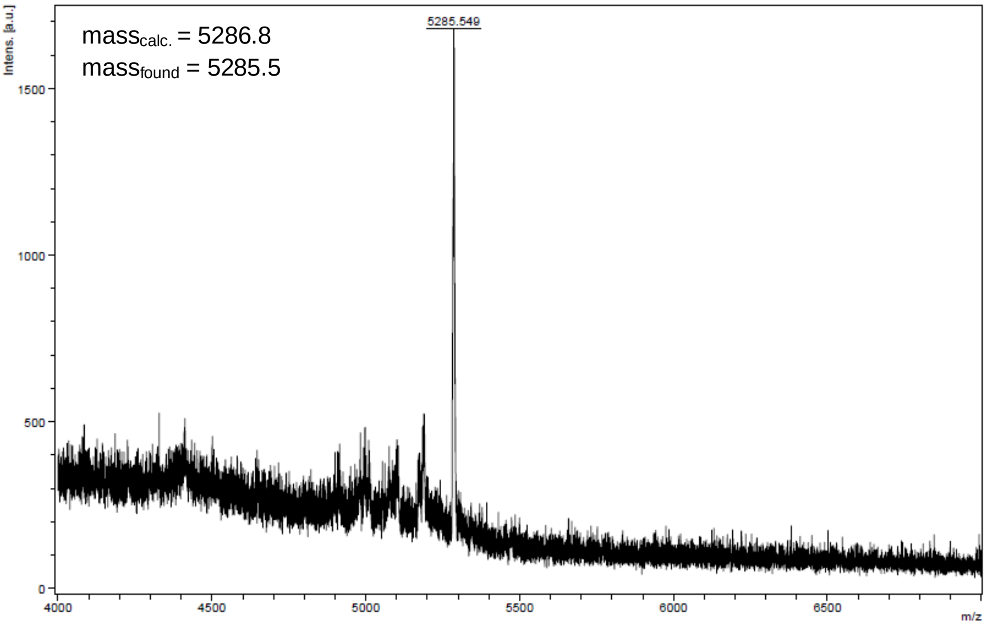

DNA conjugate 35o: CPG-bound 16mer 7De-dATC-tryptophan conjugate **33** was reacted with 4-imidazolecarboxaldehyde **34o** according to RP-12.

HPLC trace of crude reaction mixture **35o** (analytical RP-HPLC)

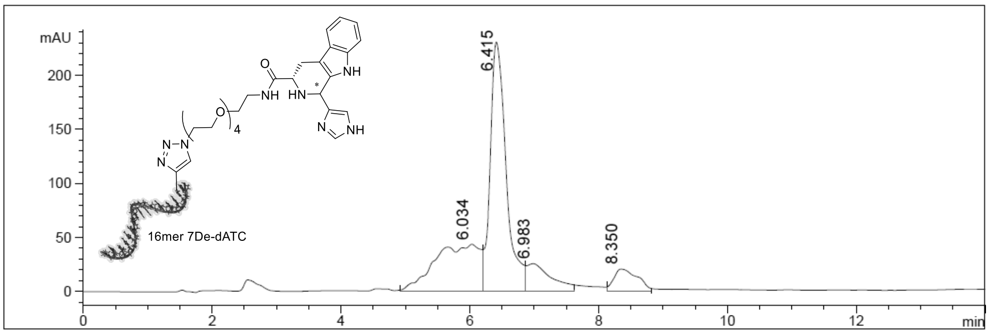

Peak list:

Ret. Time	Width min	Height	Area	Area %
6.034	0.809	43.313	2102.121	28.920
6.415	0.287	230.325	3968.849	54.602
6.983	0.443	25.403	674.466	9.279
8.350	0.428	20.369	523.313	7.199

HPLC trace of isolated product **35o** (analytical RP-HPLC)

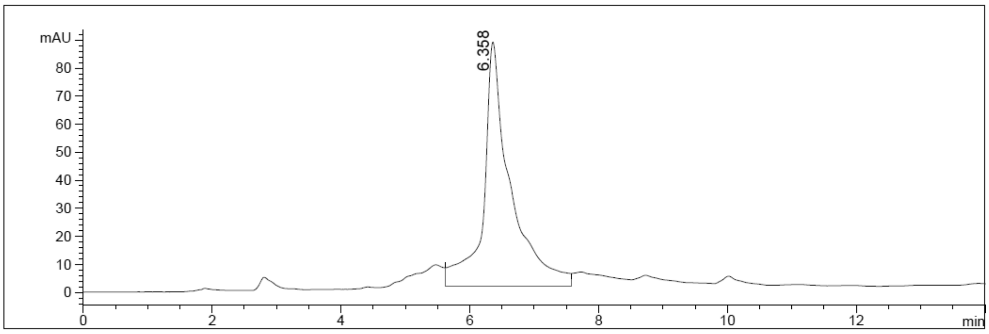

MALDI-MS spectrum of isolated product **35o**

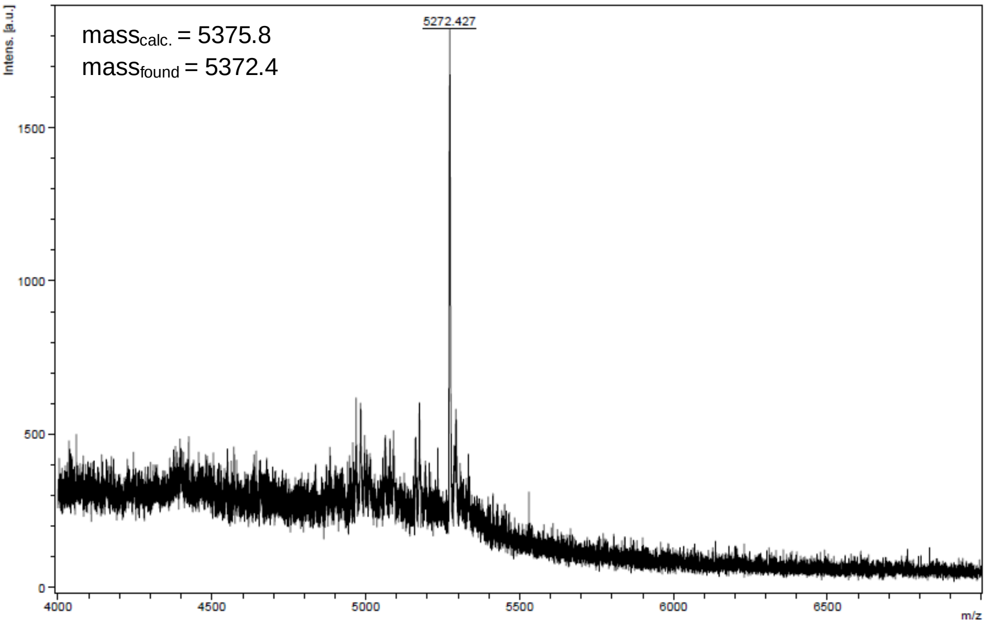

DNA conjugate 35p: CPG-bound 16mer 7De-dATC-tryptophan conjugate **33** was reacted with 3-furancarboxaldehyde **34p** according to RP-12.

HPLC trace of crude reaction mixture **35p** (analytical RP-HPLC)

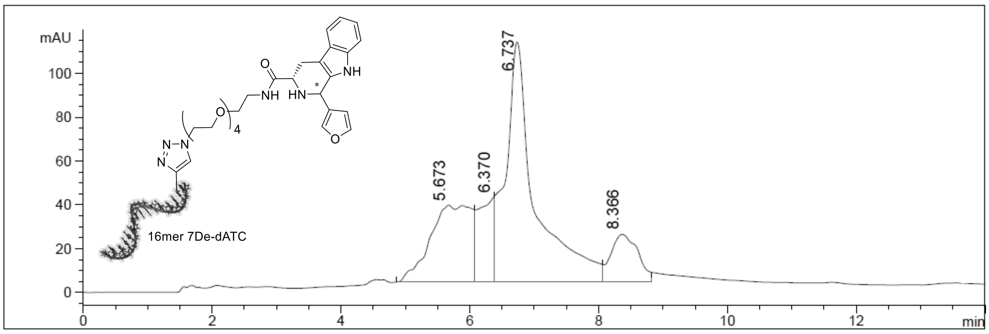

Peak list:

Ret. Time	Width min	Height	Area	Area %
5.673	0.740	35.183	1561.373	24.722
6.370	0.269	38.805	625.678	9.906
6.737	0.531	109.581	3489.398	55.248
8.366	0.488	21.832	639.385	10.124

HPLC trace of isolated product **35p** (analytical RP-HPLC)

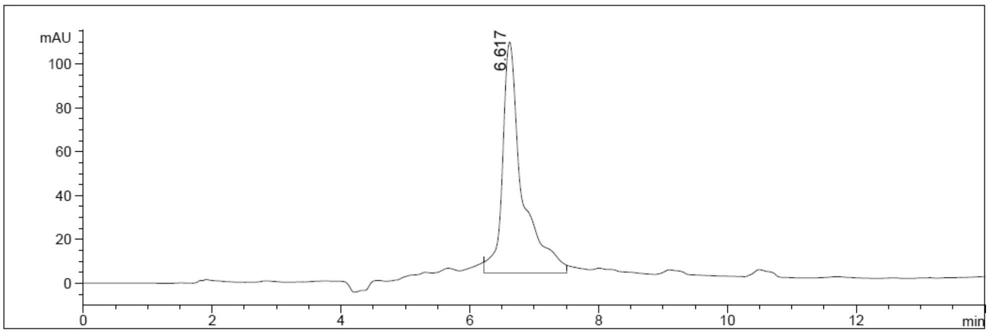

MALDI-MS spectrum of isolated product **35p**

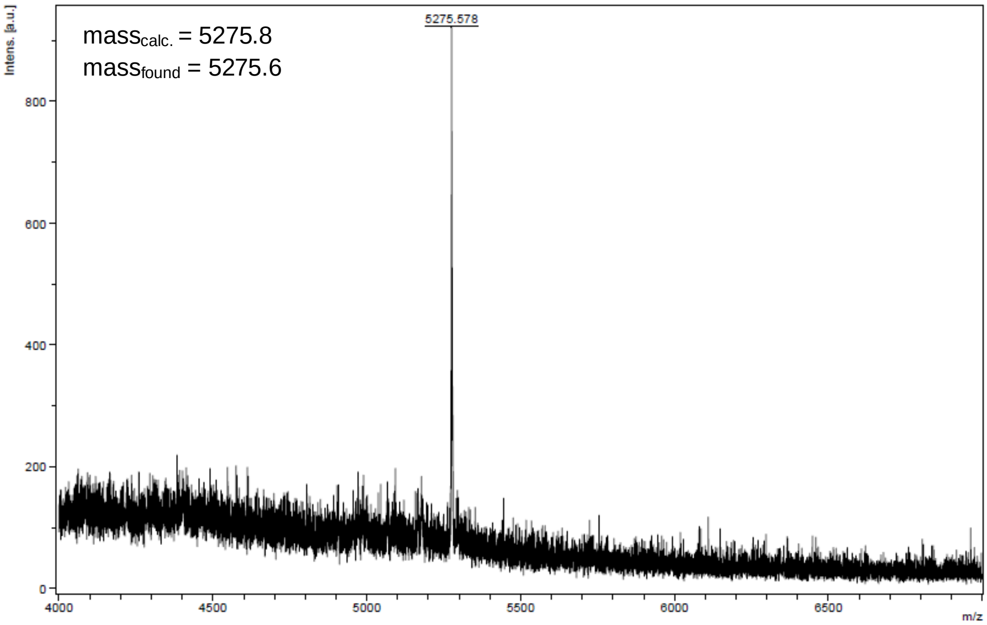

DNA conjugate 35q: CPG-bound 16mer 7De-dATC-tryptophan conjugate **33** was reacted with 2-thiophenecarboxaldehyde **34q** according to RP-12.

HPLC trace of crude reaction mixture **35q** (analytical RP-HPLC)

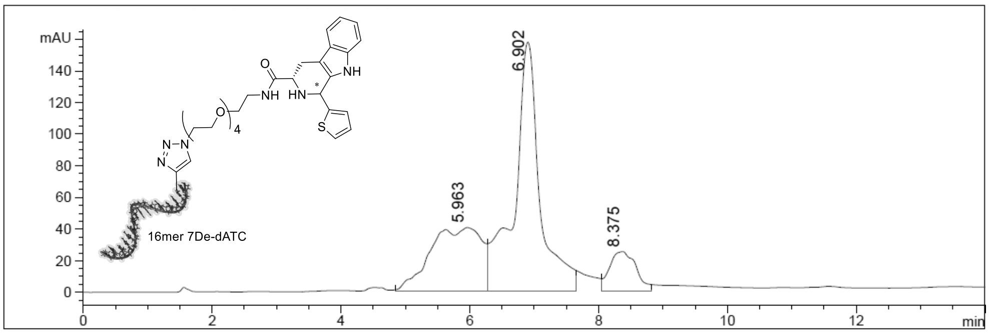

Peak list:

Ret. Time	Width min	Height	Area	Area %
5.963	0.927	39.937	2220.238	30.730
6.902	0.456	157.138	4298.798	59.499
8.375	0.475	24.758	705.940	9.771

HPLC trace of isolated product **35q** (analytical RP-HPLC)

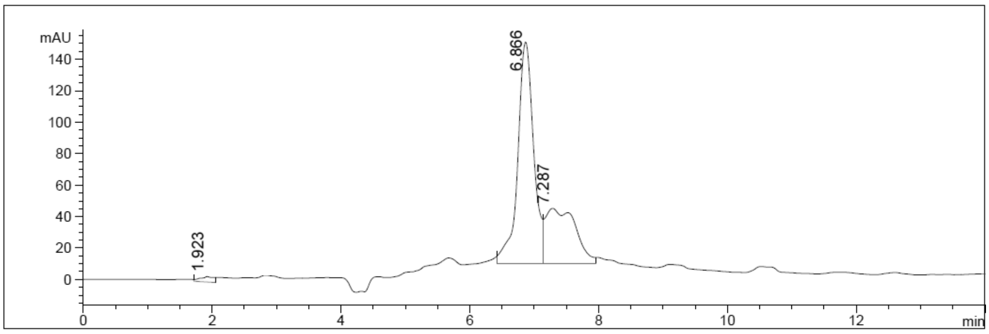

MALDI-MS spectrum of isolated product **35q**

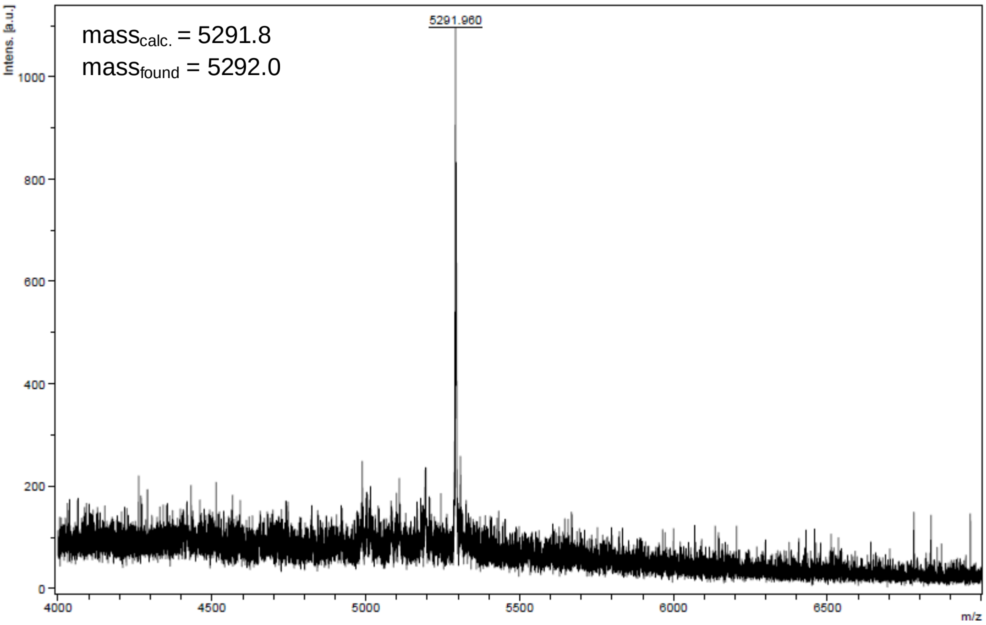

DNA conjugate 35r: CPG-bound 16mer 7De-dATC-tryptophan conjugate **33** was reacted with cyclohexanecarboxaldehyde **34r** according to RP-12.

HPLC trace of crude reaction mixture **35r** (analytical RP-HPLC)

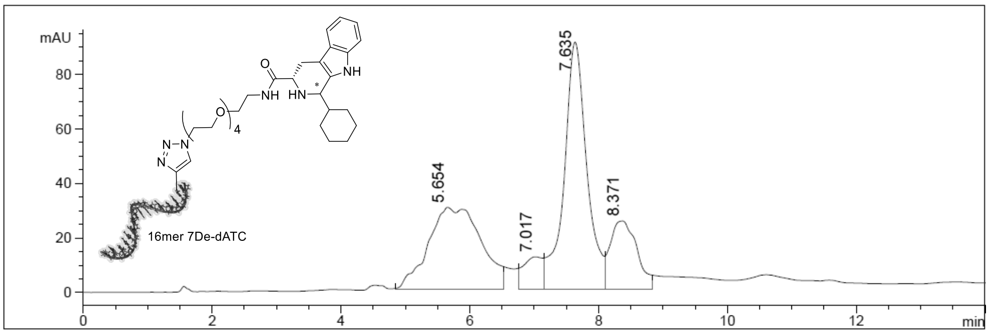


```
Peak list:

Ret. Time   Width min   Height      Area        Area %
----------------------------------------------------------
5.654       0.954       30.190      1728.326    35.159
7.017       0.349       12.004      251.036     5.107
7.635       0.406       90.834      2214.527    45.050
8.371       0.479       25.135      721.807     14.684
----------------------------------------------------------
```

HPLC trace of isolated product **35r** (analytical RP-HPLC)

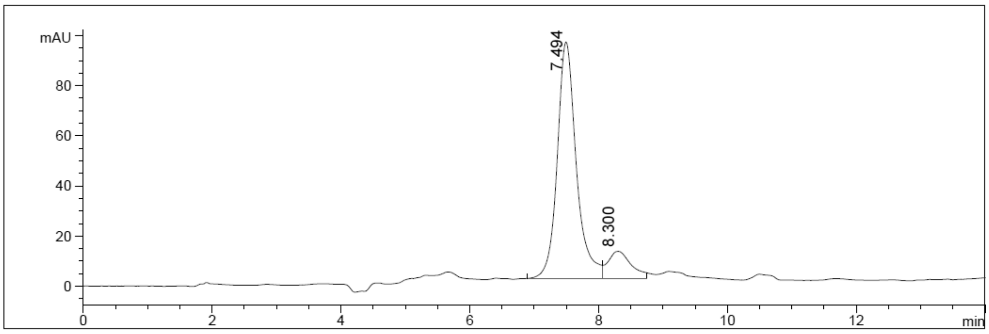

MALDI-MS spectrum of isolated product **35r**

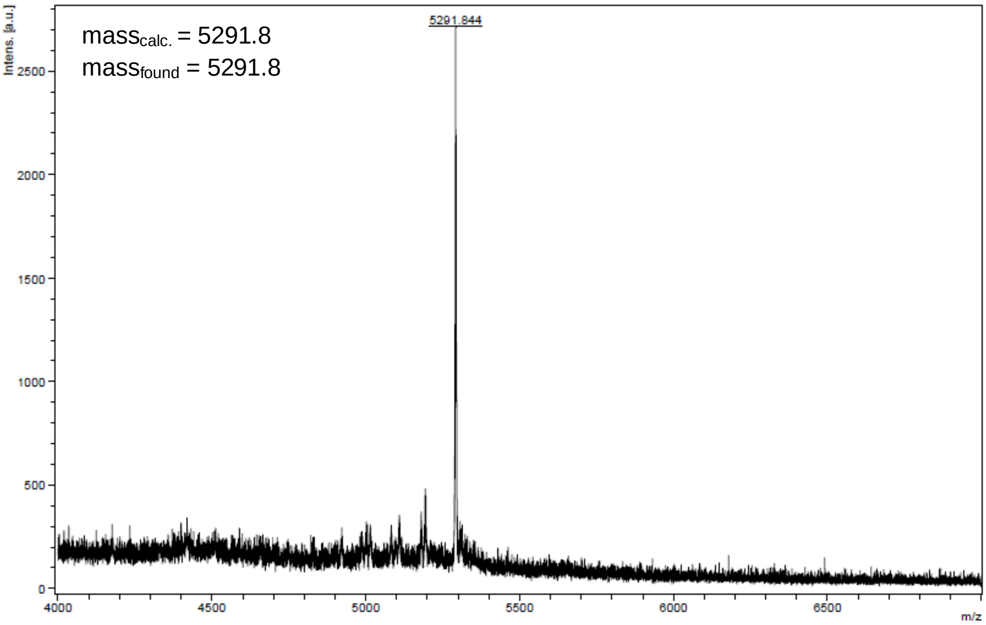

DNA conjugate 35s: CPG-bound 16mer 7De-dATC-tryptophan conjugate **33** was reacted with isobutyraldehyde **34s** according to RP-12.

HPLC trace of crude reaction mixture **35s** (analytical RP-HPLC)

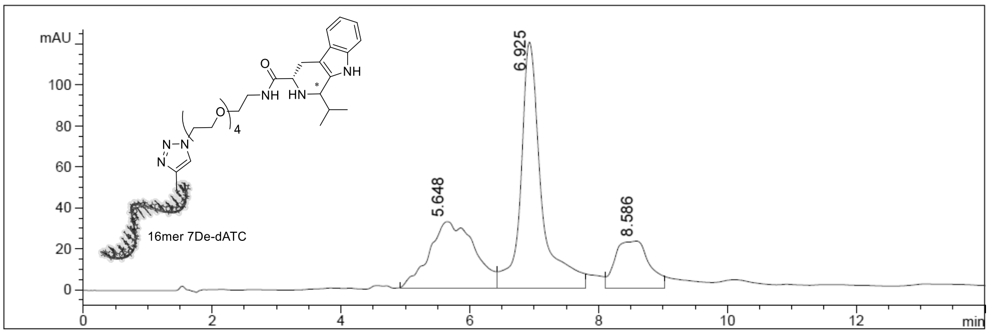


```
Peak list:

Ret. Time   Width min   Height      Area        Area %
-------------------------------------------------------
5.648       0.819       32.304      1587.856    31.197
6.925       0.370       119.977     2661.492    52.290
8.586       0.612       22.906      840.478     16.513
-------------------------------------------------------
```

HPLC trace of isolated product **35s** (analytical RP-HPLC)

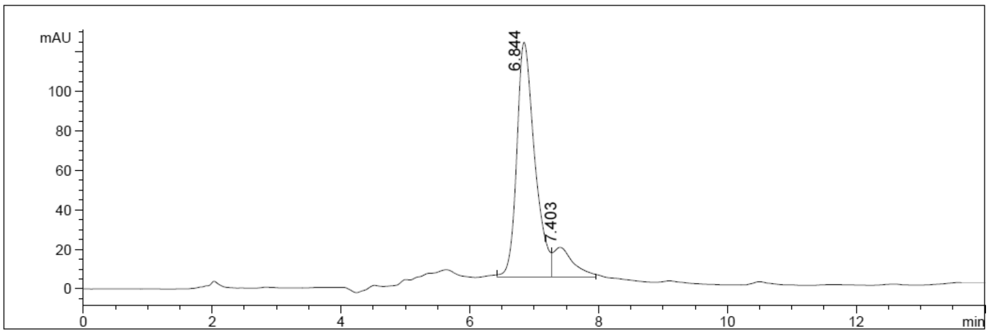


```
Peak list:

Ret. Time   Width min   Height      Area        Area %
-------------------------------------------------------
6.844       0.322       118.829     2297.196    87.509
7.403       0.364       15.022      327.896     12.491
-------------------------------------------------------
```

MALDI-MS spectrum of isolated product **35s**

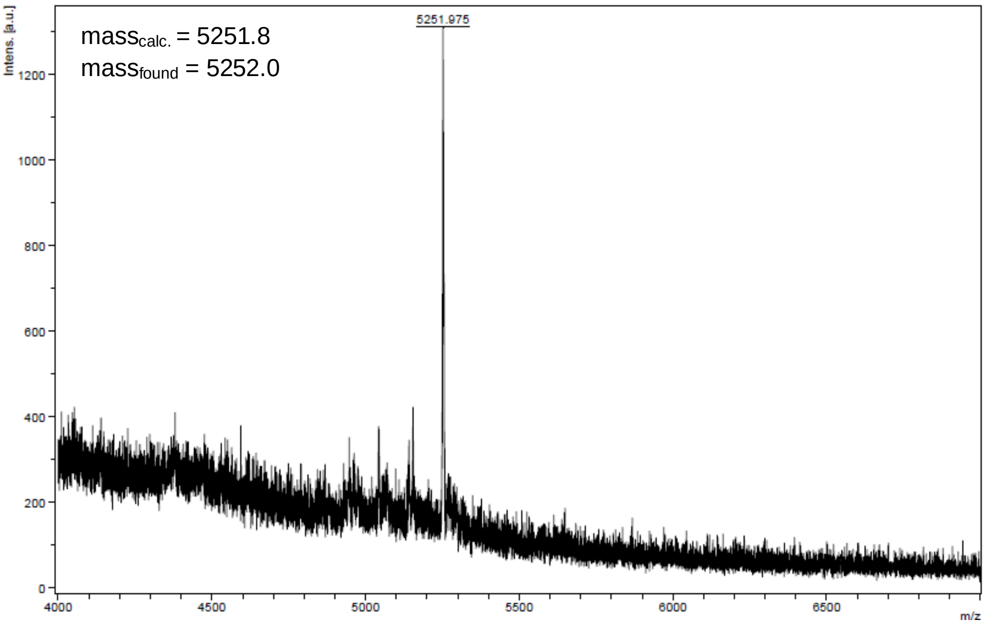

Lewis acid-promoted reactions

aza-Diels-Alder reaction

DNA conjugate 37: CPG-bound 16mer 7De-dATC-aldehyde conjugate **13** (Batch A) was reacted with aniline **29** and danishefsky's diene **36** according to RP-13.

HPLC trace of crude reaction mixture **37** (analytical RP-HPLC)

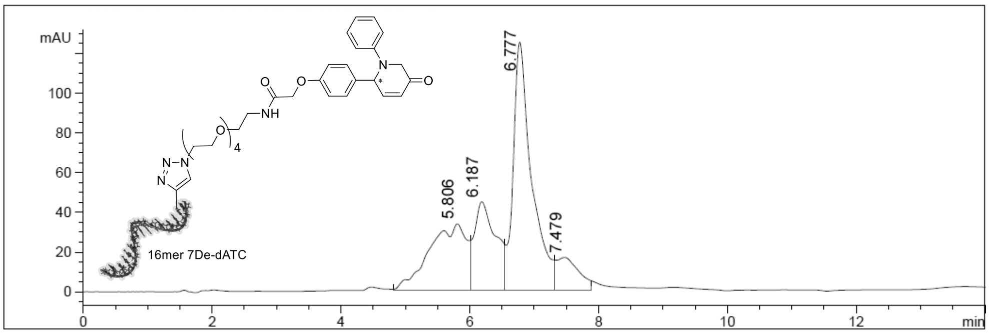

Peak list:

Ret. Time	Width min	Height	Area	Area %
5.806	0.670	33.149	1333.260	25.624
6.187	0.385	44.289	1022.680	19.655
6.777	0.329	124.521	2456.650	47.214
7.479	0.400	16.287	390.671	7.508

HPLC trace of isolated product **37** (analytical RP-HPLC)

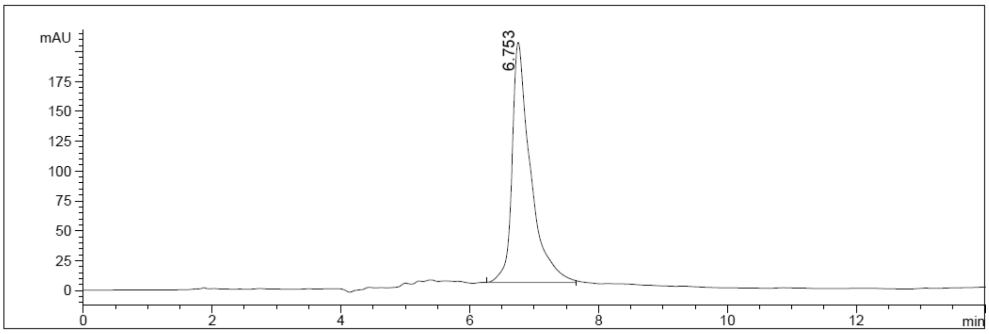

MALDI-MS spectrum of isolated product **37**

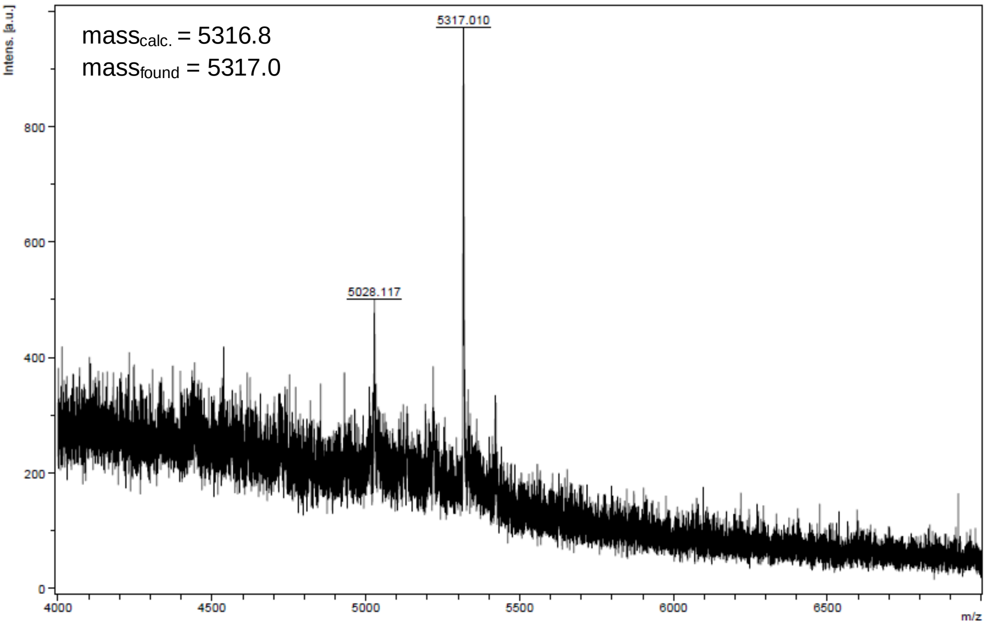

Petasis reaction

DNA conjugate 41: CPG-bound 16mer 7De-dATC-piperidine conjugate **38** was reacted with phenylboronic acid **39** and glyoxylic acid monohydrate **40** according to RP-14.

HPLC trace of crude reaction mixture **41** (analytical RP-HPLC)

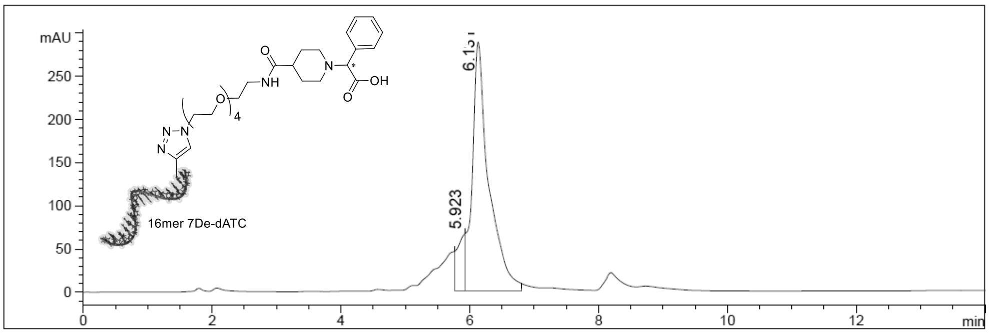

Peak list:

Ret. Time	Width min	Height	Area	Area %
5.923	0.136	66.107	538.617	9.513
6.131	0.296	288.072	5123.305	90.487

HPLC trace of isolated product **41** (analytical RP-HPLC)

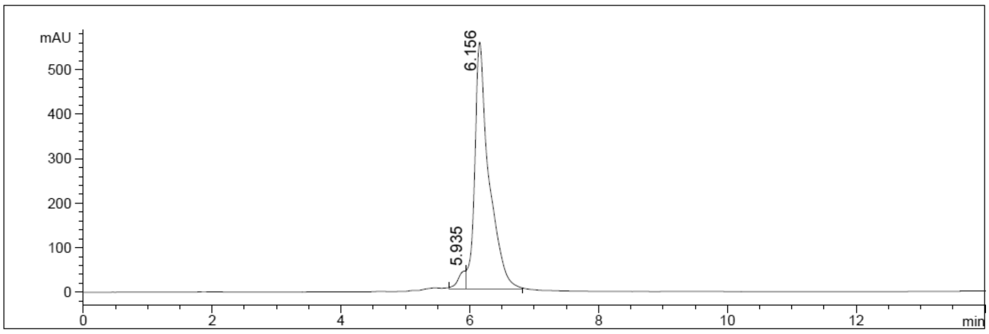

Peak list:

Ret. Time	Width min	Height	Area	Area %
5.935	0.132	40.027	315.843	3.581
6.156	0.256	553.993	8505.322	96.419

MALDI-MS spectrum of isolated product **41**

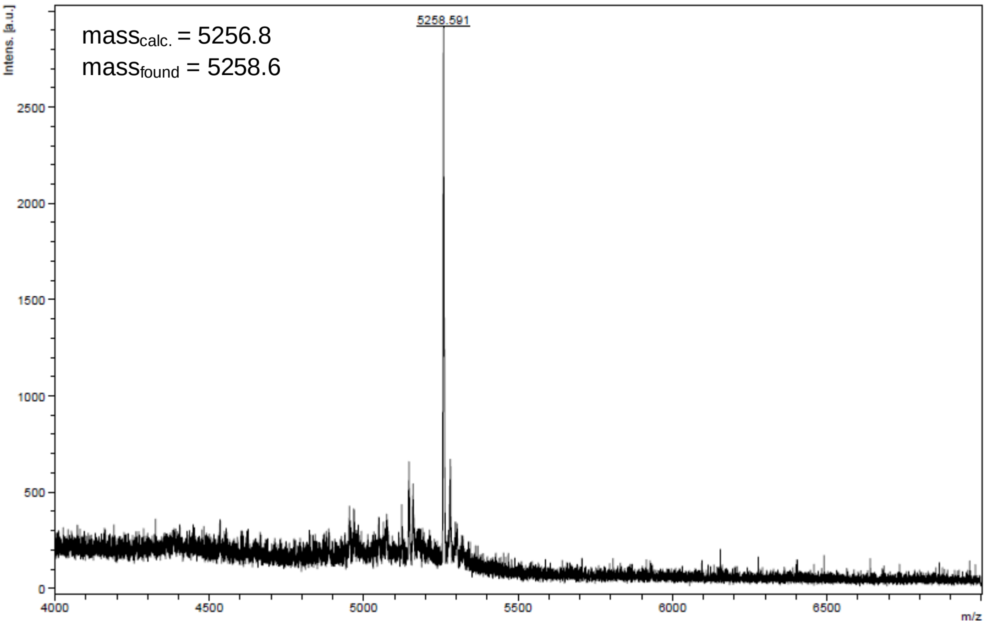

1,3-Dipolar cycloaddition

DNA conjugate 44: CPG-bound 16mer 7De-dATC-glycine conjugate **42** was reacted with benzaldehyde **34a** and *N*,*N*-dimethylacrylamide **43** according to RP-15.

HPLC trace of crude reaction mixture **44** (analytical RP-HPLC)

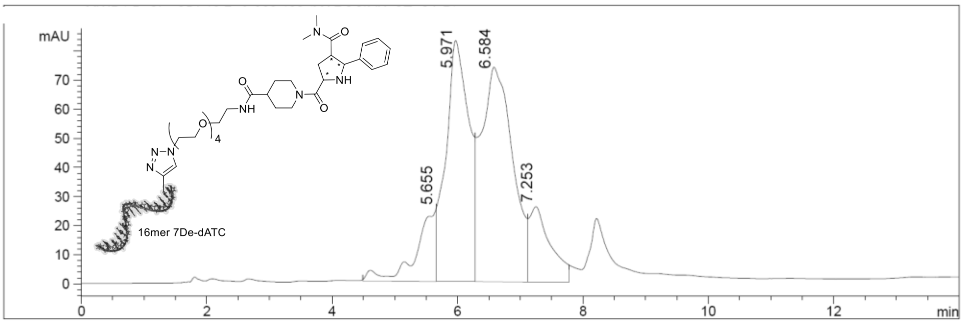

Peak list:

Ret. Time	Width min	Height	Area	Area %
5.655	0.370	24.695	547.793	9.384
5.971	0.424	82.834	2106.302	36.083
6.584	0.582	73.750	2577.183	44.149
7.253	0.390	25.889	606.125	10.383

HPLC trace of isolated product **44** (analytical RP-HPLC)

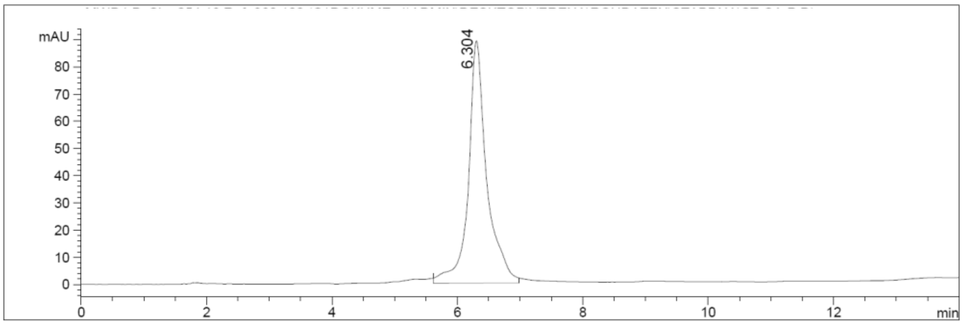

Peak list:

Ret. Time	Width min	Height	Area	Area %
6.304	0.341	89.168	1821.869	100.000

MALDI-MS spectrum of isolated product **44**

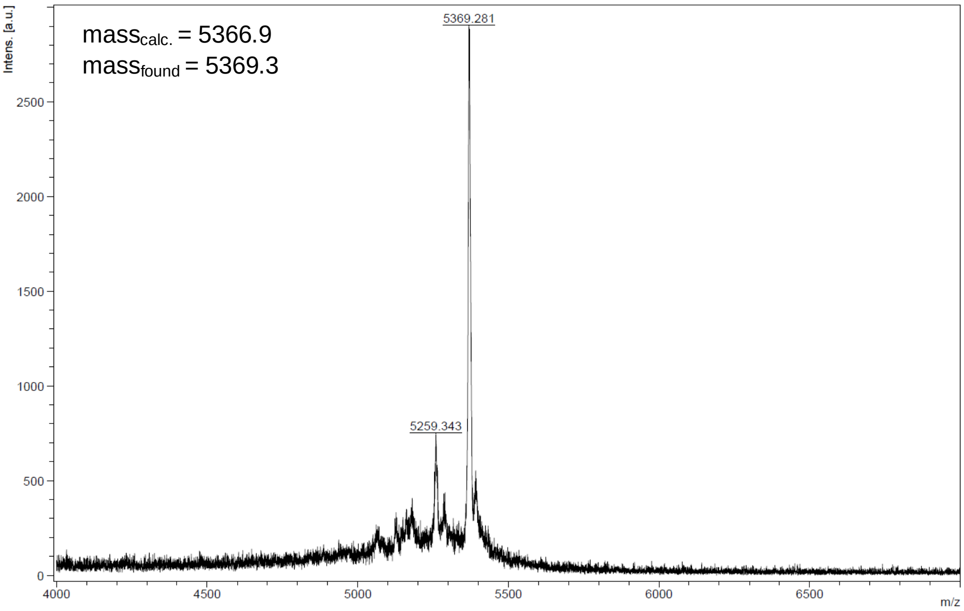

Castagnoli-Cushman reaction

DNA conjugate 46: CPG-bound 16mer 7De-dATC-aldehyde conjugate **13** (Batch A) was reacted with aniline **29** and homophthalic anhydride according to RP-16.

HPLC trace of crude reaction mixture **46** (analytical RP-HPLC)

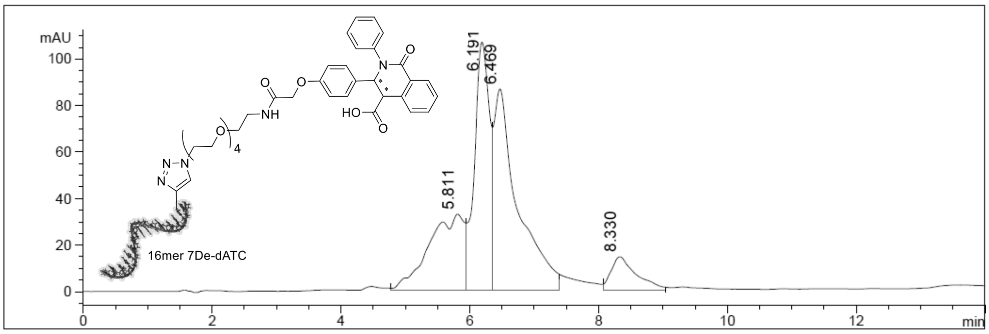

Peak list:

Ret. Time	Width min	Height	Area	Area %
5.811	0.631	32.579	1234.025	22.138
6.191	0.269	106.581	1720.041	30.857
6.469	0.431	86.415	2233.315	40.064
8.330	0.452	14.270	386.927	6.941

HPLC trace of isolated product **46** (analytical RP-HPLC)

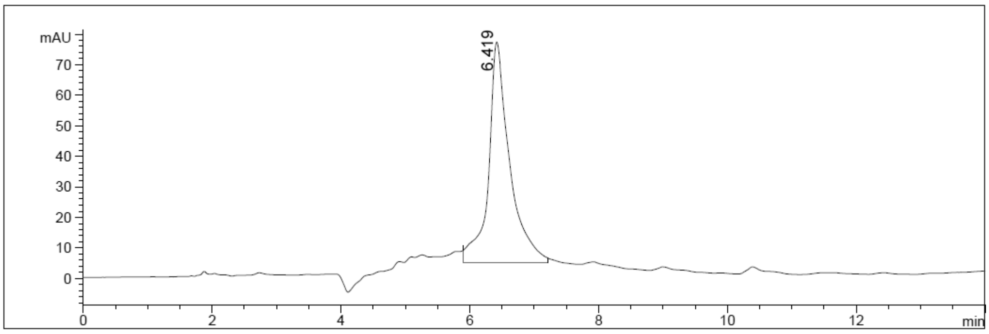

MALDI-MS spectrum of isolated product **46**

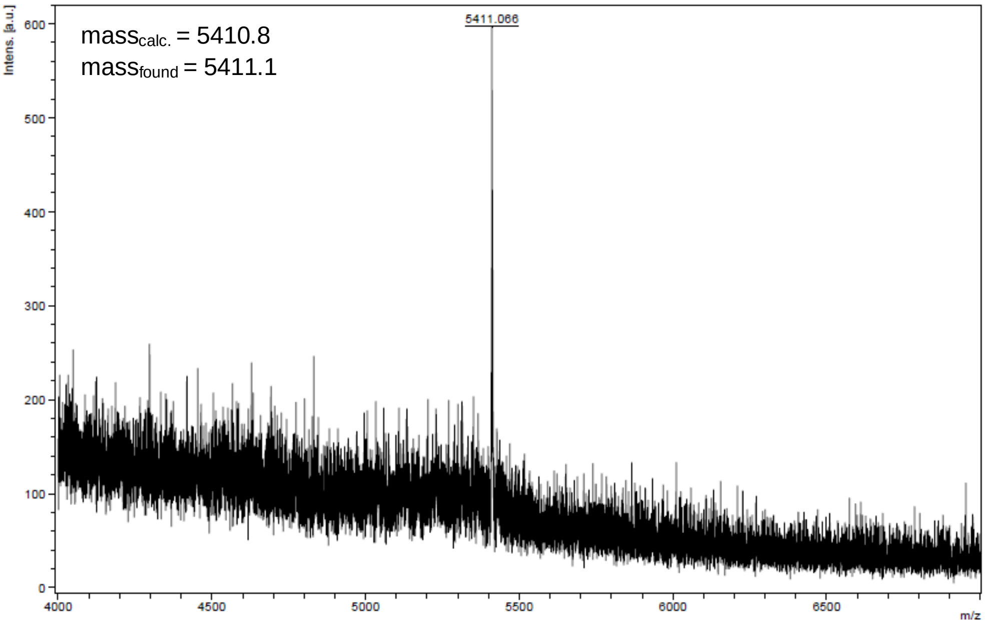

Yb(PFO)$_3$-mediated three-component synthesis of pyrazoles

Optimization

Table S12 – Optimization of the Yb(PFO)$_3$-mediated three-component synthesis of pyrazoles on CPG-bound TC.

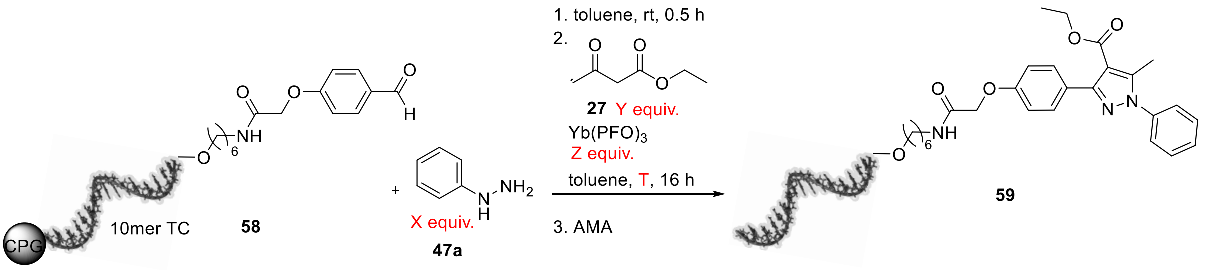

Entry	Reaction conditions	HPLC trace of the crude reaction mixture
1	TC-aldehyde conjugate **58**	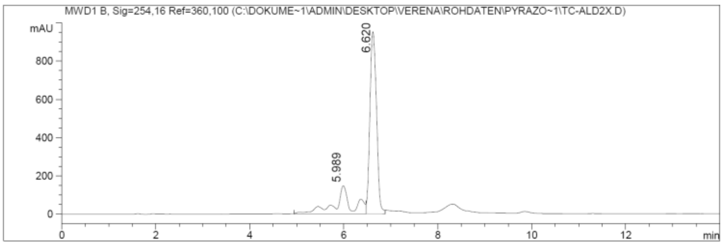
2	TC-pyrazole product **59**	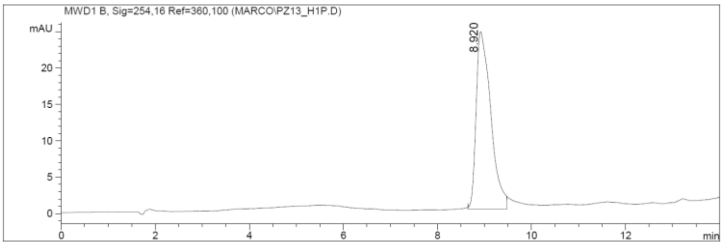
3	1. 1000 equiv. **47a**<br>2. 1000 equiv. **27**<br>100 equiv. Yb(PFO)3<br>80 °C<br><br>=> product traces in MALDI-MS	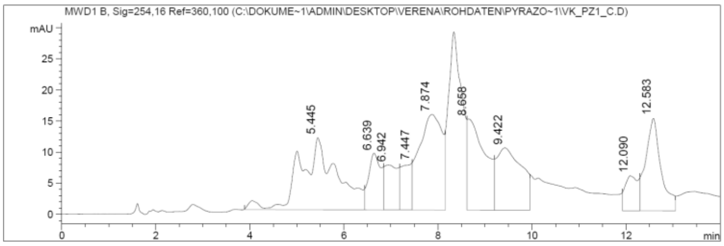
4	1. 500 equiv. **47a**<br>2. 1000 equiv. **27**<br>100 equiv. Yb(PFO)$_3$<br>80 °C<br><br>=> 33% conversion<br>=> 47% degradation	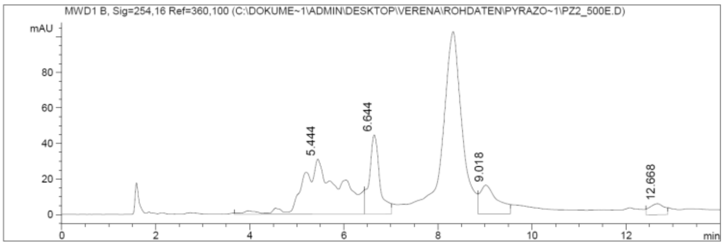

5

1. 250 equiv. **47a**
2. 1000 equiv. **27**
   100 equiv. $Yb(PFO)_3$
   80 °C
   => 31% conversion
   => 44% degradation

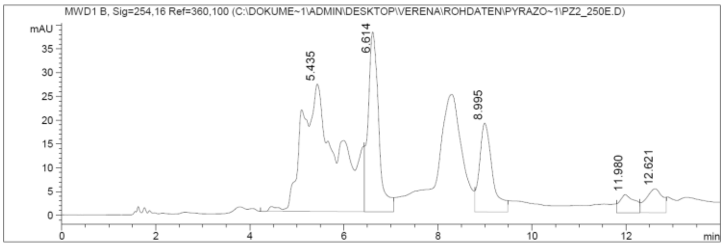

6

1. 250 equiv. **47a**
2. 1000 equiv. **27**
   250 equiv. $Yb(PFO)_3$
   80 °C

   => 64% conversion
   => 56% degradation

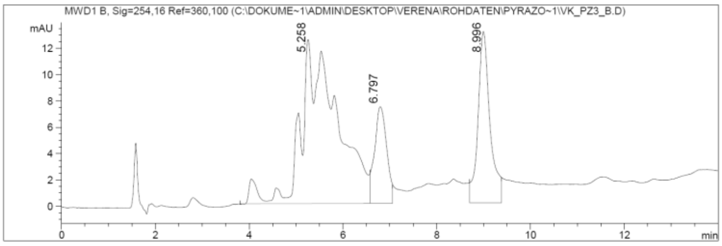

7

1. 250 equiv. **47a**
2. 3000 equiv. **27**
   250 equiv. $Yb(PFO)_3$
   80 °C

   => 83% conversion
   => 76% degradation

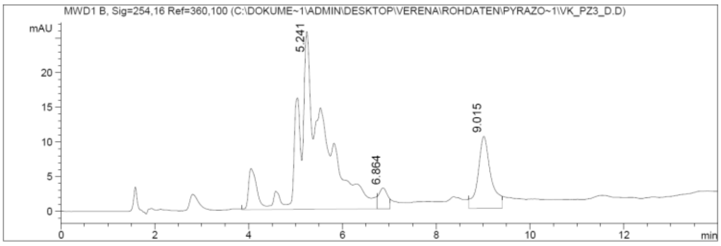

8

1. 250 equiv. **47a**
2. 1000 equiv. **27**
   250 equiv. $Yb(PFO)_3$
   50 °C

   => 63% conversion
   => 26% degradation

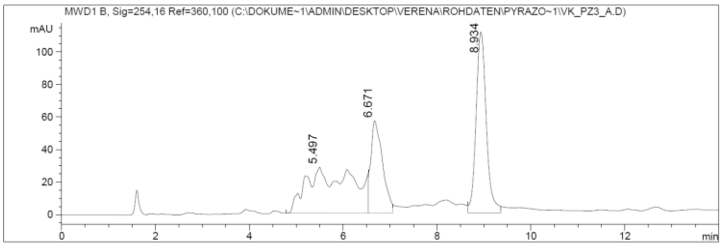

9

1. 250 equiv. **47a**
2. 3000 equiv. **27**
   250 equiv. $Yb(PFO)_3$
   50 °C

   => 67% conversion
   => 23% degradation

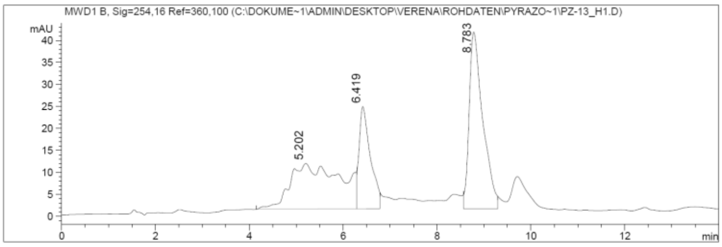

10

1. 250 equiv. **47a**
2. 3000 equiv. **27**
   0 equiv. $Yb(PFO)_3$
   50 °C

   => 0% conversion
   => 17% degradation

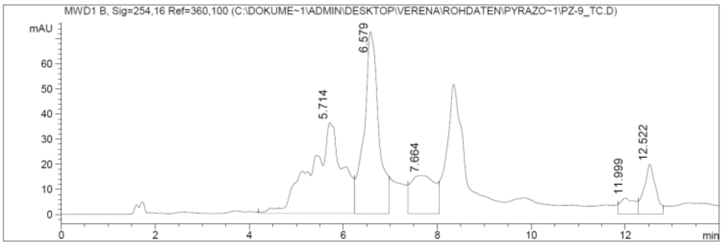

DNA-conjugate 59: CPG-bound 10mer TC-aldehyde conjugate **58** was reacted with phenylhydrazine **47a** and ethyl acetoacetate **27** according to RP-17.

HPLC trace of crude reaction mixture **58** (analytical RP-HPLC)

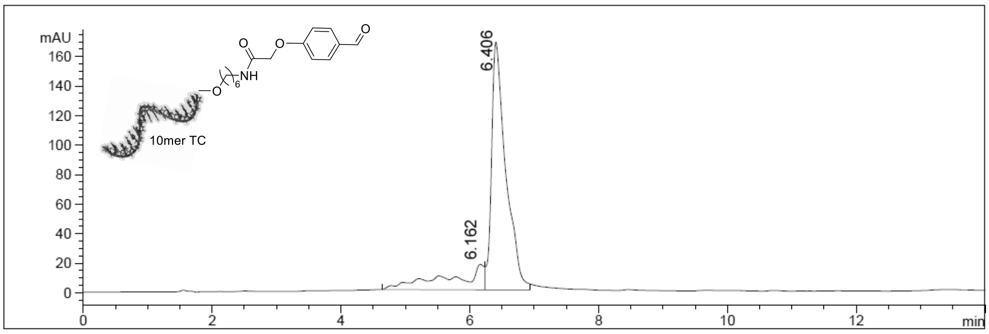

Peak list:

Ret. Time	Width min	Height	Area	Area %
6.162	0.628	17.337	653.460	20.229
6.406	0.256	168.084	2576.888	79.771

HPLC trace of crude reaction mixture **59** (analytical RP-HPLC)

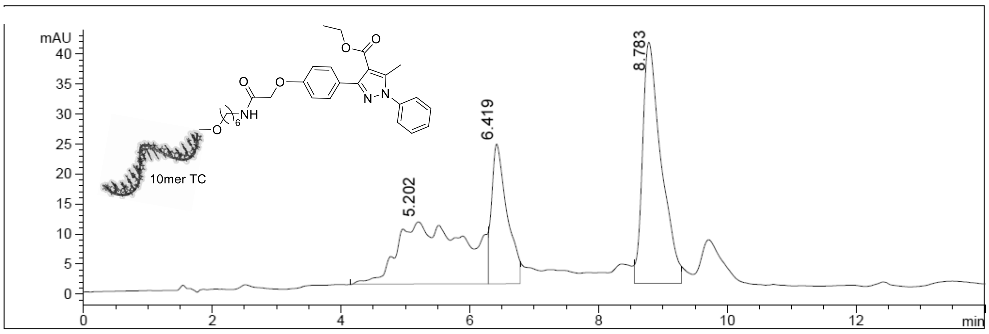

Peak list:

Ret. Time	Width min	Height	Area	Area %
5.202	1.224	10.328	758.468	38.816
6.419	0.277	23.261	387.187	19.815
8.783	0.335	40.175	808.360	41.369

HPLC trace of isolated product **59** (Analytical RP-HPLC)

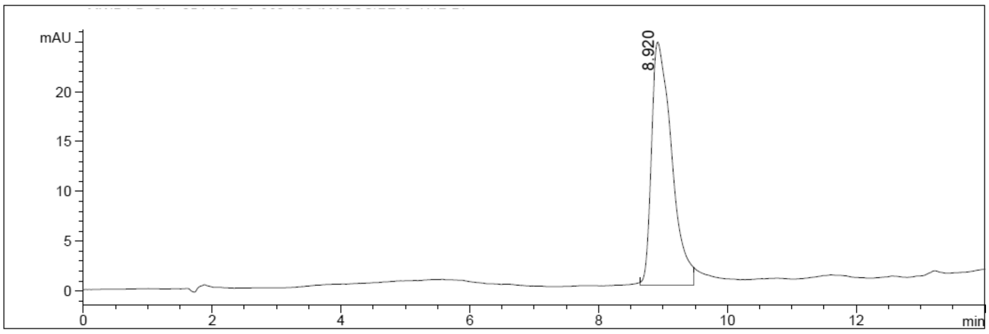

Peak list:

Ret. Time	Width min	Height	Area	Area %
8.920	0.362	24.406	529.737	100.000

MALDI-MS spectrum of the crude **59**

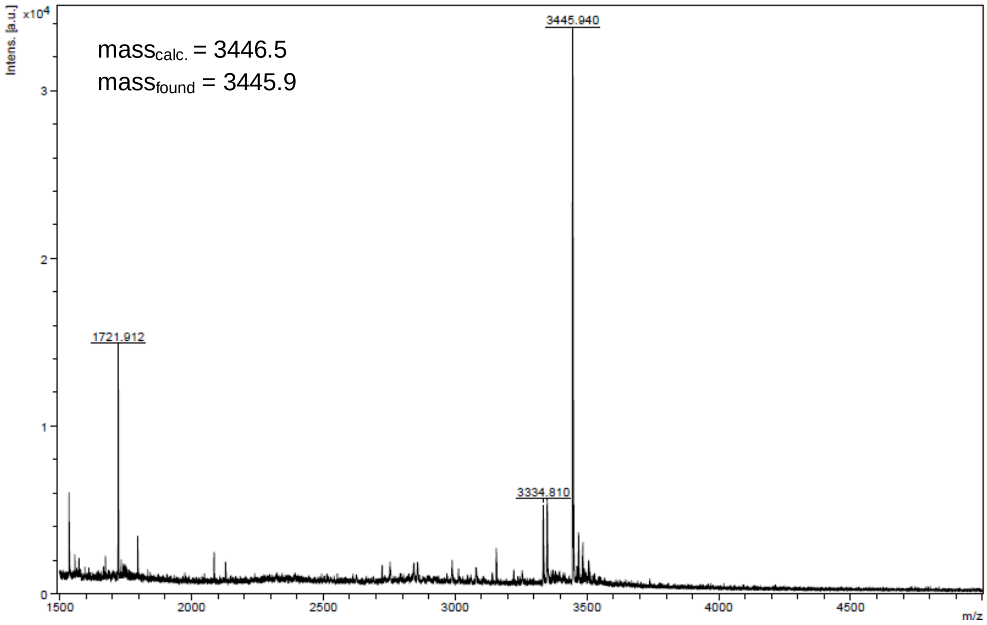

DNA-conjugate 60: CPG-bound 10mer ATGC-aldehyde conjugate **56** was reacted with phenylhydrazine **47a** and ethyl acetoacetate **27** according to RP-17.

HPLC trace of starting material **56** (analytical RP-HPLC)

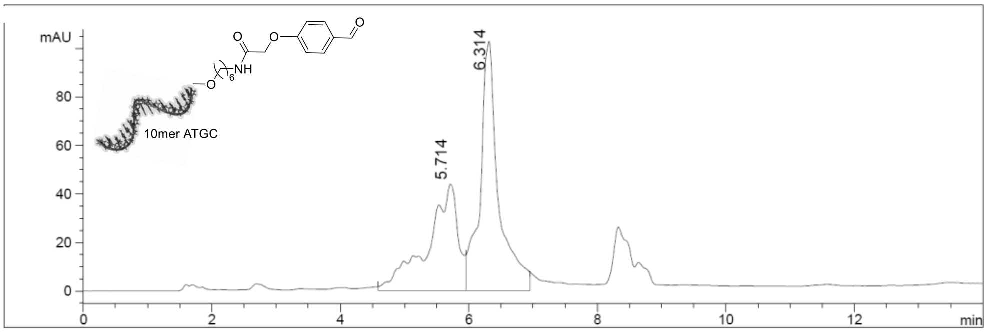

Peak list:

Ret. Time	Width min	Height	Area	Area %
5.714	0.554	43.887	1457.896	41.631
6.314	0.332	102.670	2044.085	58.369

HPLC trace of crude reaction mixture **60** (analytical RP-HPLC)

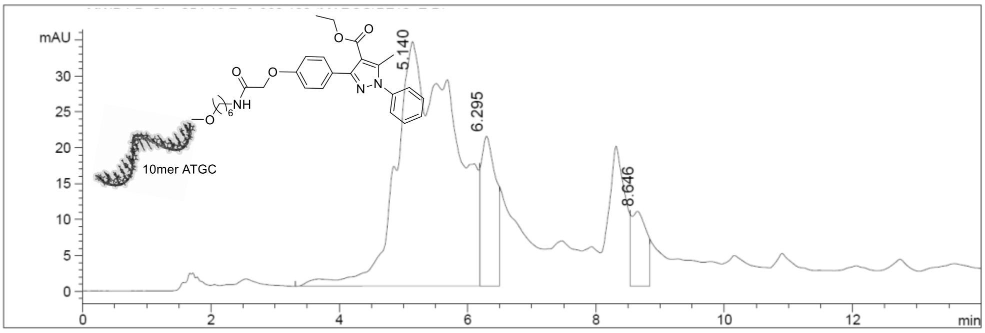

Peak list:

Ret. Time	Width min	Height	Area	Area %
5.140	1.048	33.973	2136.606	81.556
6.295	0.255	20.825	319.143	12.182
8.646	0.264	10.365	164.057	6.262

MALDI-MS spectrum of reaction crude **60**

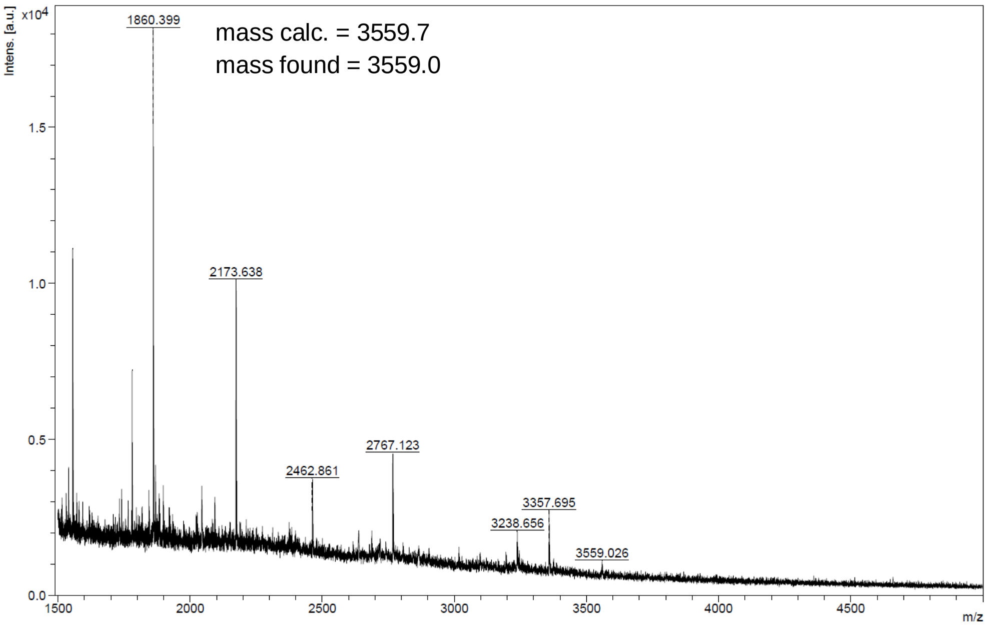

Table S13 – HPLC traces for the $Yb(PFO)_3$-mediated pyrazole synthesis on different DNA sequences.

**Oligonucleotide**	**HPLC traces**	**DNA degradation [%]**
10mer TC **59**	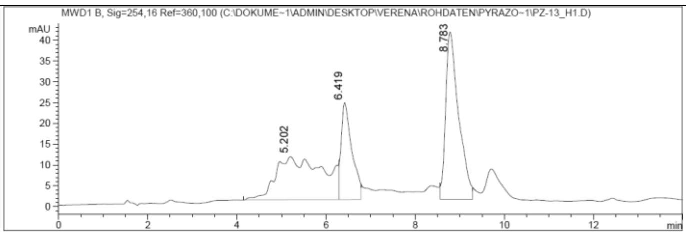	23
10mer ATGC **60**	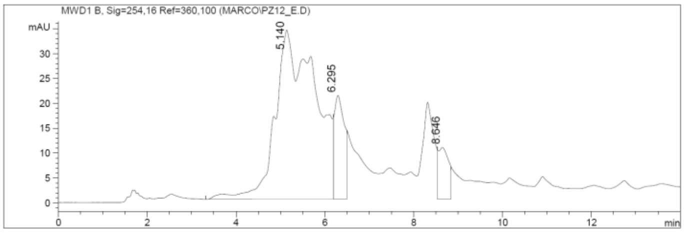	69
16mer 7De-dATC **48a**	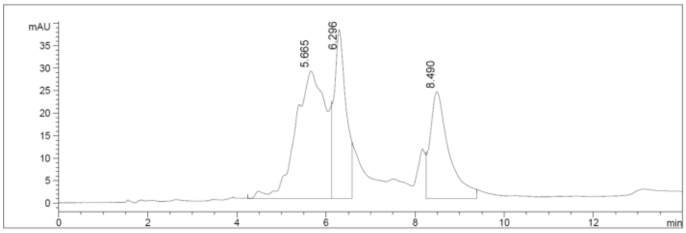	33

10mer TC = 5'-TTCCTCTCCT-3', 10mer ATGC = 5'-GTCATGATCT-3', 16mer 7De-dATC = 5'-CT*C TCT TT7De-dA 7De-dACT 7De-dACC T-3'

Scope

Table S14 – Scope of $Yb(PFO)_3$-mediated three-component synthesis of pyrazoles on CPG-bound 16mer 7De-dATC oligonucleotide-aldehyde conjugate **13** using different hydrazines **47**.[a]

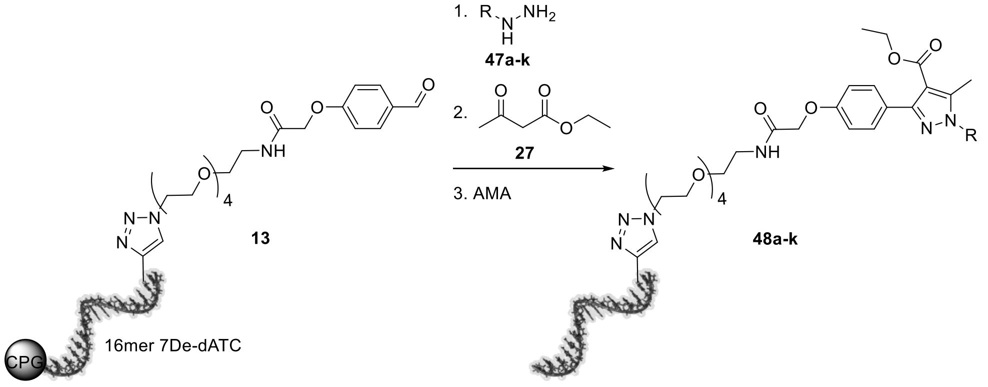

Entry	Product	Hydrazine	Conversion [%][b]	DNA degradation [%][c]	$Mass_{calc.}$ $Mass_{found}$[d]
1	**48a**	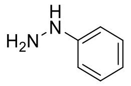 **47a**	50	33	5373.9<br>5371.0
2	**48b**	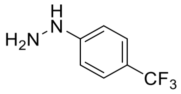 **47b**	44	17	5441.9<br>5443.5
3	**48c**	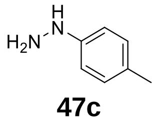 **47c**	44	29	5387.9<br>5388.7
4	**48d**	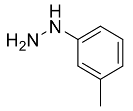 **47d**	39	35	5387.9<br>5389.6
5	**48e**	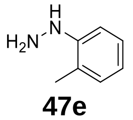 **47e**	41	46	5387.9<br>5390.4
6	**48f**	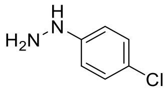 **47f**	56	20	5408.3<br>5409.5
7	**48g**	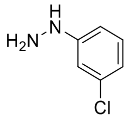 **47g**	51	20	5408.3<br>5410.1
8	**48h**	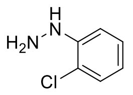 **47h**	56	25	5408.3<br>5409.6
9	**48i**	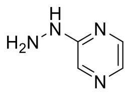	n.d.	-	n.d.

		**47i**			
10	**48j**	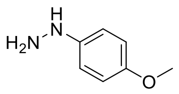 **47j**	27	50	5403.9<br>5405.0
11	**48k**	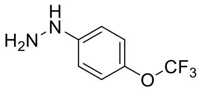 **47k**	47	25	5457.9<br>5459.5

[a] CPG-bound oligonucleotide conjugate **13** (20 nmol) with hydrazine **47** (250 equiv.,5 µmol) in 30 µL in toluene at ambient temperature for 30 min, followed by addition of ethyl acetoacetate **27** (3000 equiv., 60 µmol) and $Yb(PFO)_3$ (250 equiv., 5 µmol) suspended in 50 µL toluene and shaking of the reaction mixture at 50 °C for 16 h. DNA cleavage with AMA (30% aqueous ammonia / 40% aqueous methylamine, 1:1 (vol/vol)) at ambient temperature for 4 h. [b] Determined by analytical RP-HPLC analysis based on the ratios of **48** to **47**. [c] Determined by comparison of purities of the analytical RP-HPLC traces of starting material **47** and the crude reaction mixture **48**. [d] Measured by MALDI-MS. 7De-dATC = 5'-CT*C TCT TT7De-dA 7De-dACT 7De-dACC T-3'.

DNA conjugate 13: CPG-bound 16mer 7De-dATC-PEG(4)-NH_2 conjugate was reacted with 4-formyl-phenoxyacetic acid according to RP-04.

HPLC trace of crude reaction mixture **13** (Batch D, analytical RP-HPLC)

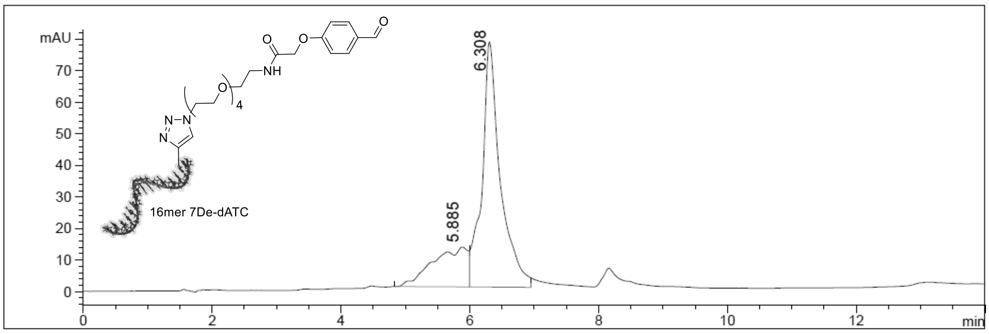

Peak list:

Ret. Time	Width min	Height	Area	Area %
5.885	0.628	12.621	475.272	23.565
6.308	0.331	77.614	1541.567	76.435

MALDI-MS spectrum of crude reaction mixture **13**

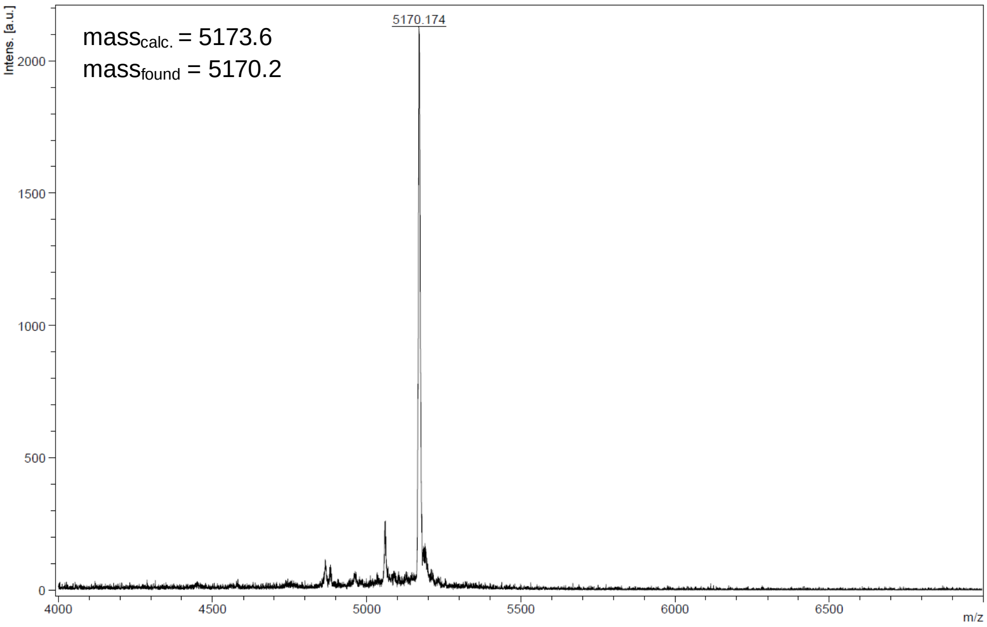

DNA conjugate 48a: CPG-bound 16mer 7De-dATC-aldehyde conjugate **13** (Batch D) was reacted with phenylhydrazine **47a** and ethyl acetoacetate **27** according to RP-17.

HPLC trace of crude reaction mixture **48a** (analytical RP-HPLC)

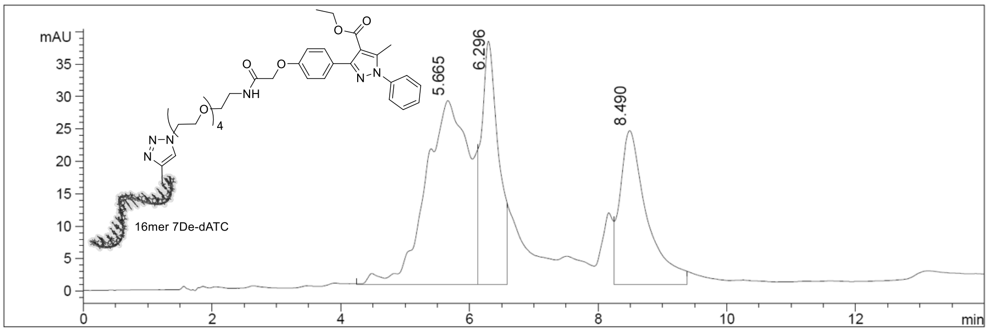

Peak list:

Ret. Time	Width min	Height	Area	Area %
5.665	0.790	28.376	1345.380	49.419
6.296	0.303	37.534	683.324	25.100
8.490	0.487	23.763	693.711	25.481

HPLC trace of isolated product **48a** (analytical RP-HPLC)

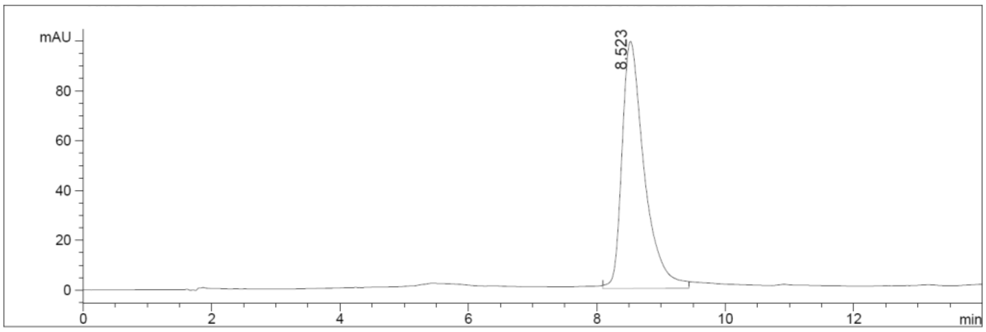

Peak list:

Ret. Time	Width min	Height	Area	Area %
8.523	0.402	99.283	2396.015	100.000

MALDI-MS spectrum of isolated product **48a**

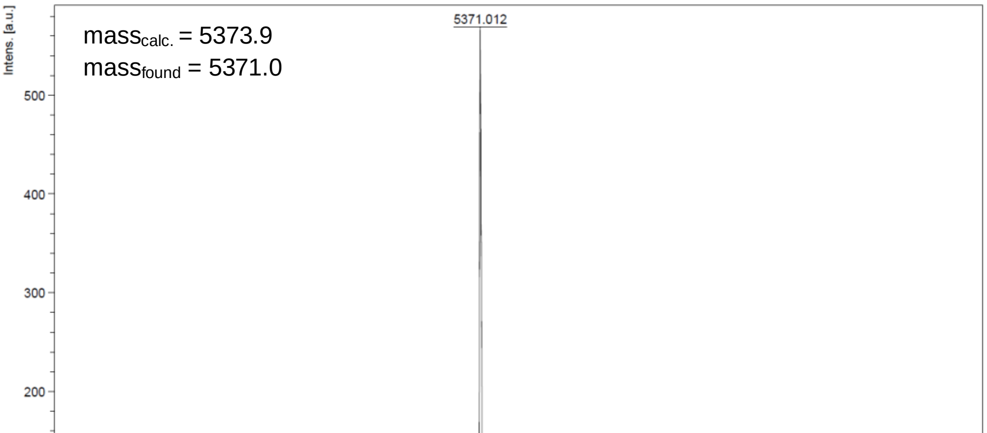

DNA conjugate 48b: CPG-bound 16mer 7De-dATC-aldehyde conjugate **13** (Batch D) was reacted with 4-(trifluoromethyl)phenylhydrazine **47b** and ethyl acetoacetate **27** according to RP-17.

HPLC trace of crude reaction mixture **48b** (analytical RP-HPLC)

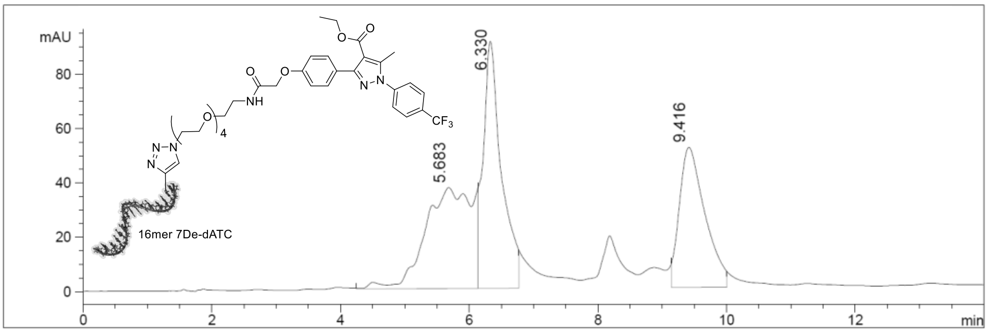

Peak list:

Ret. Time	Width min	Height	Area	Area %
5.683	0.846	37.178	1887.085	36.932
6.330	0.329	90.887	1796.700	35.163
9.416	0.461	51.550	1425.840	27.905

HPLC trace of isolated product **48b** (analytical RP-HPLC)

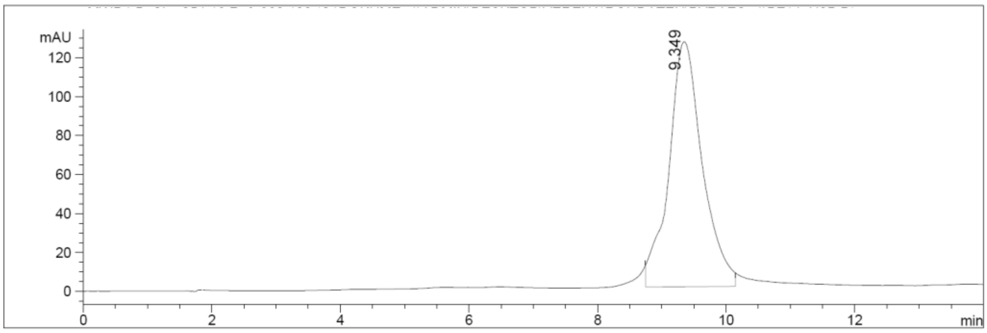

Peak list:

Ret. Time	Width min	Height	Area	Area %
9.349	0.602	125.780	4543.807	100.000

MALDI-MS spectrum of isolated product **48b**

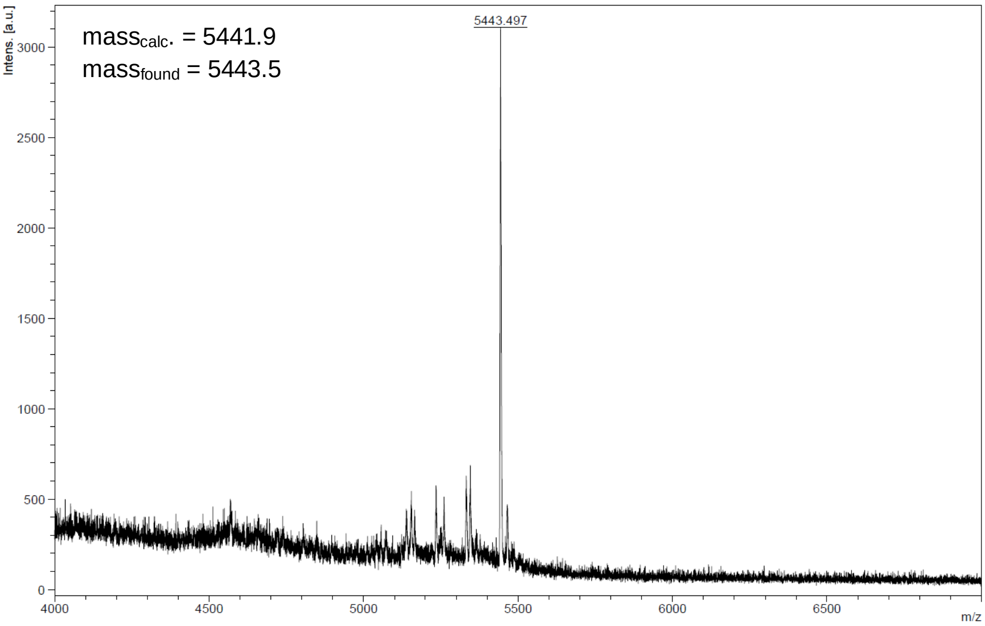

DNA conjugate 48c: CPG-bound 16mer 7De-dATC-aldehyde conjugate **13** (Batch D) was reacted with 4-methylphenylhydrazine **47c** and ethyl acetoacetate **27** according to RP-17.

HPLC trace of crude reaction mixture **48c** (analytical RP-HPLC)

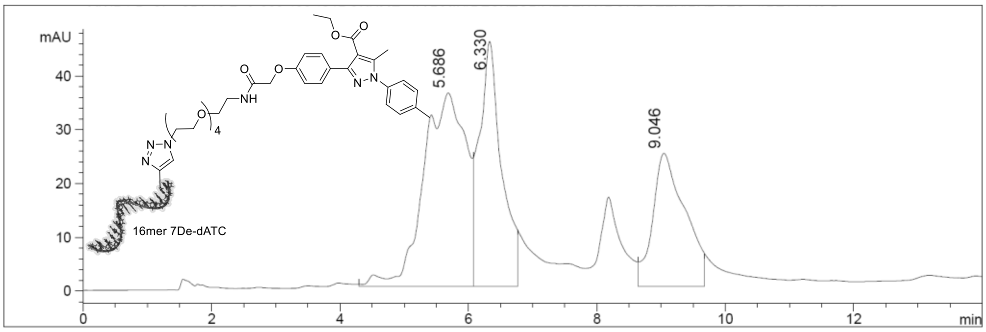

Peak list:

Ret. Time	Width min	Height	Area	Area %
5.686	0.769	35.984	1660.853	45.892
6.330	0.399	45.569	1090.502	30.132
9.046	0.584	24.763	867.683	23.976

HPLC trace of isolated product **48c** (analytical RP-HPLC)

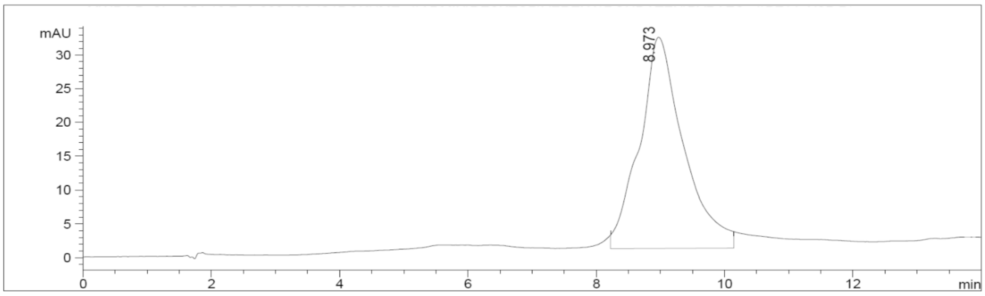

Peak list:

Ret. Time	Width min	Height	Area	Area %
8.973	0.798	31.276	1496.716	100.000

MALDI-MS spectrum of isolated product **48c**

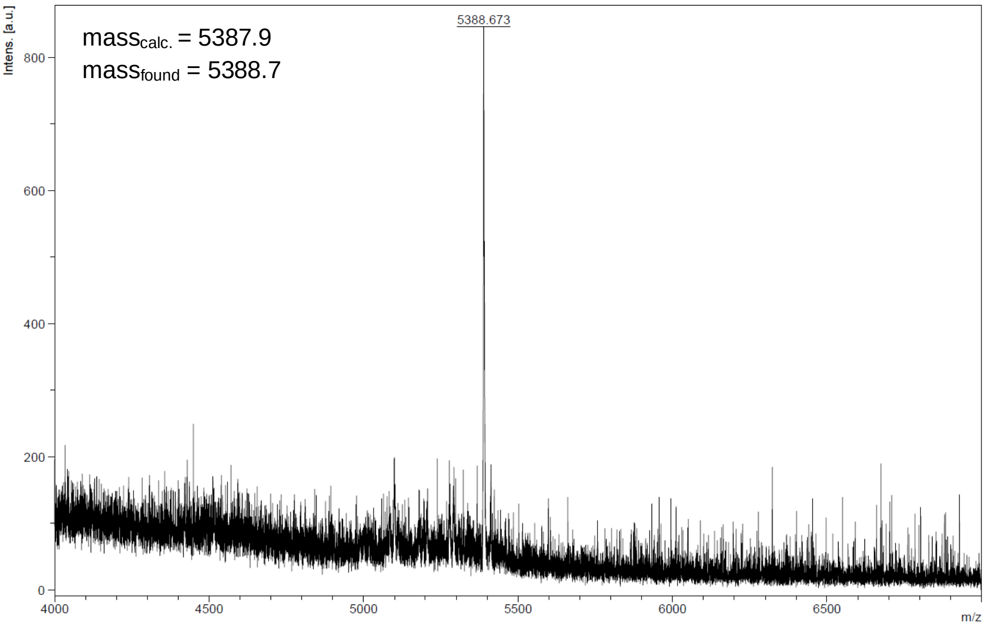

DNA conjugate 48d: CPG-bound 16mer 7De-dATC-aldehyde conjugate **13** (Batch D) was reacted with 3-methylphenylhydrazine **47d** and ethyl acetoacetate **27** according to RP-17.

HPLC trace of crude reaction mixture **48d** (analytical RP-HPLC)

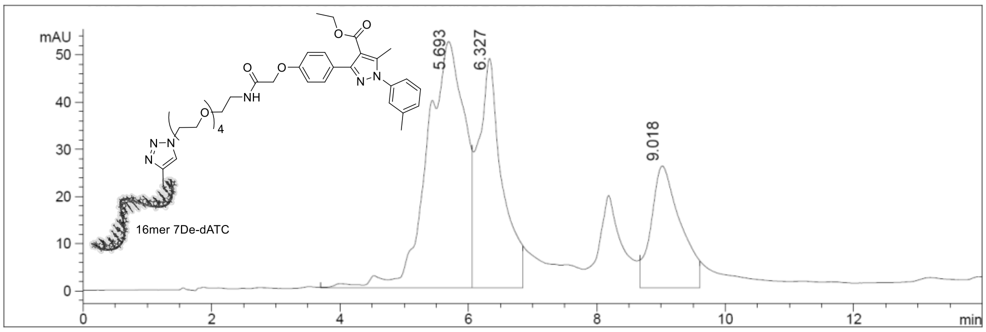

Peak list:

Ret. Time	Width min	Height	Area	Area %
5.693	0.680	52.181	2128.686	50.563
6.327	0.433	48.579	1260.958	29.952
9.018	0.530	25.799	820.354	19.486

HPLC trace of isolated product **48d** (analytical RP-HPLC)

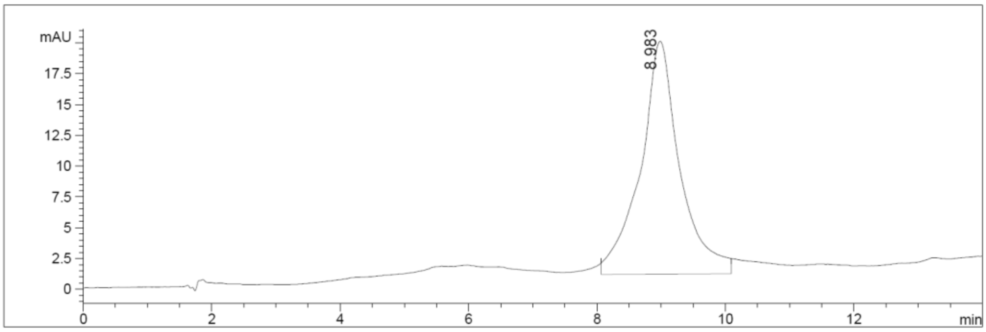

Peak list:

Ret. Time	Width min	Height	Area	Area %
8.983	0.713	18.891	807.881	100.000

MALDI-MS spectrum of isolated product **48d**

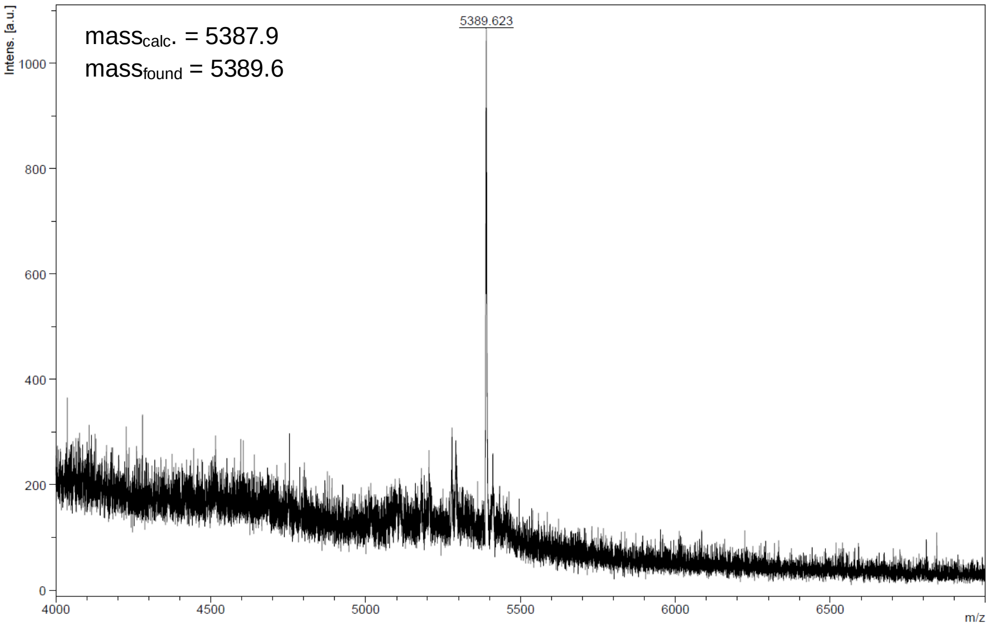

DNA conjugate 48e: CPG-bound 16mer 7De-dATC-aldehyde conjugate **13** (Batch D) was reacted with 2-methylphenylhydrazine **47e** and ethyl acetoacetate **27** according to RP-17.

HPLC trace of crude reaction mixture **48e** (analytical RP-HPLC)

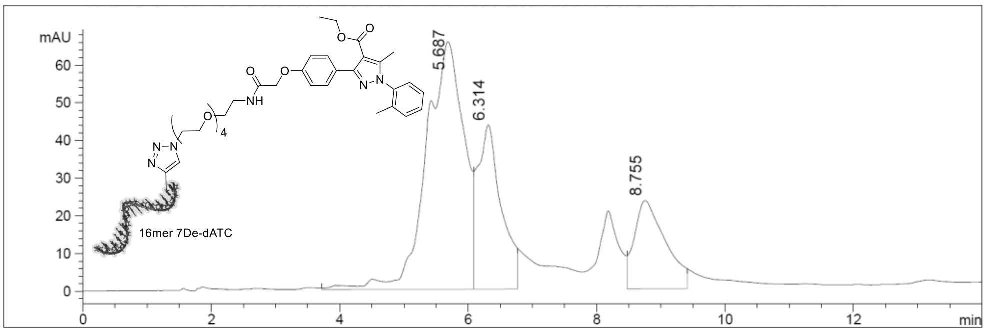

Peak list:

Ret. Time	Width min	Height	Area	Area %
5.687	0.681	65.629	2682.498	58.554
6.314	0.426	43.523	1113.078	24.297
8.755	0.560	23.393	785.640	17.149

HPLC trace of isolated product **48e** (analytical RP-HPLC)

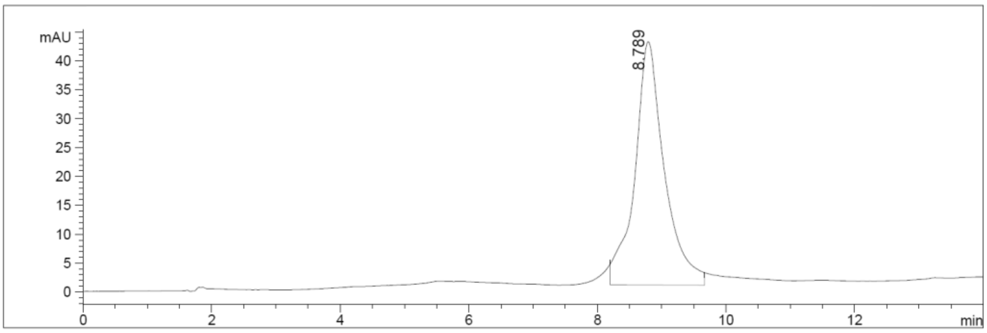

Peak list:

Ret. Time	Width min	Height	Area	Area %
8.789	0.535	42.092	1349.989	100.000

MALDI-MS spectrum of isolated product **48e**

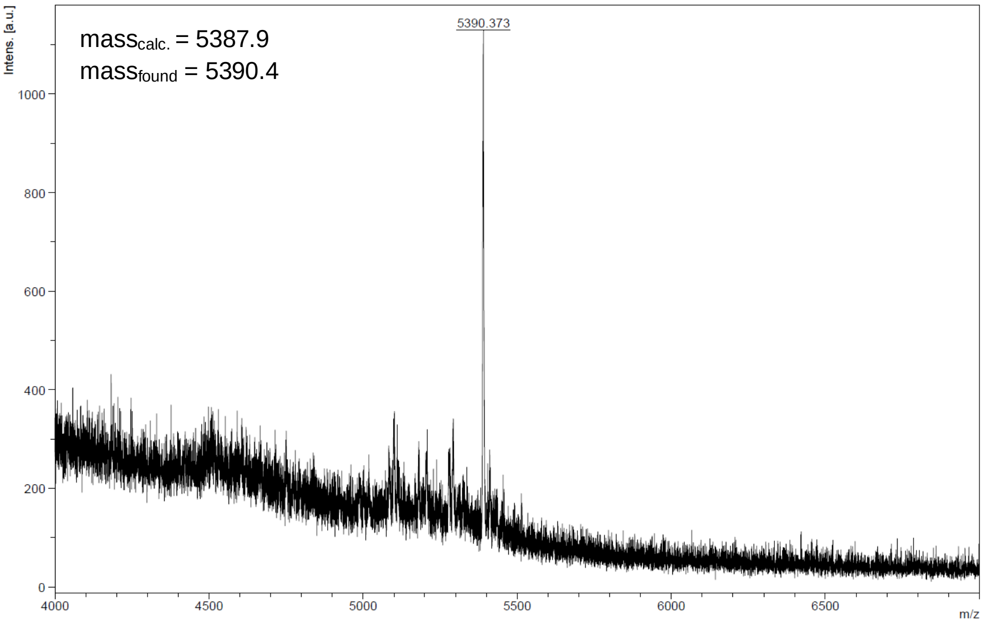

DNA conjugate 48f: CPG-bound 16mer 7De-dATC-aldehyde conjugate **13** (Batch D) was reacted with 4-chlorophenylhydrazine **47f** and ethyl acetoacetate **27** according to RP-17.

HPLC trace of crude reaction mixture **48f** (analytical RP-HPLC)

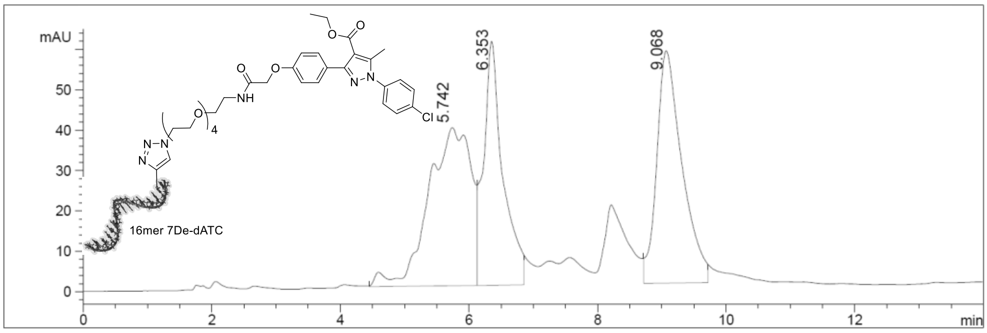

Peak list:

Ret. Time	Width min	Height	Area	Area %
5.742	0.763	39.164	1793.282	38.758
6.353	0.347	60.401	1256.698	27.161
9.068	0.457	57.524	1576.895	34.081

HPLC trace of isolated product **48f** (analytical RP-HPLC)

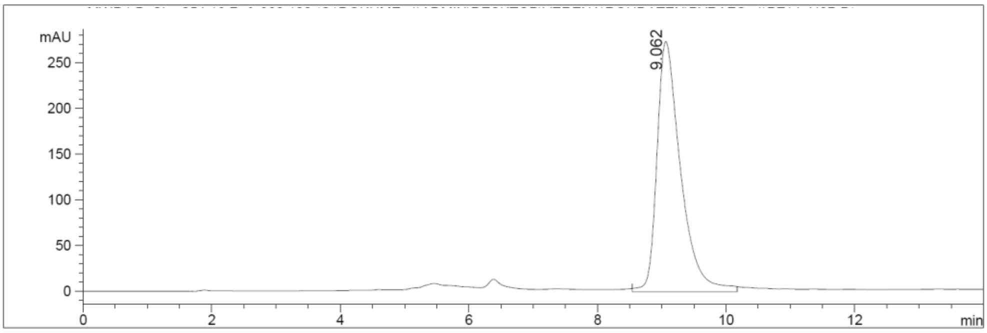

Peak list:

Ret. Time	Width min	Height	Area	Area %
9.062	0.429	274.044	7047.611	100.000

MALDI-MS spectrum of isolated product **48f**

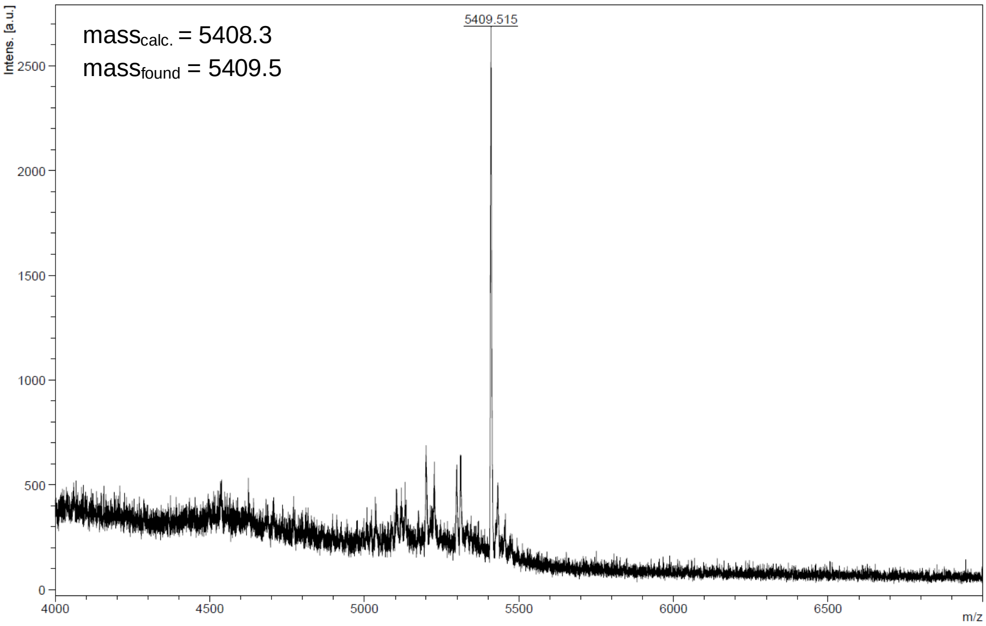

DNA conjugate 48g: CPG-bound 16mer 7De-dATC-aldehyde conjugate **13** (Batch D) was reacted with 3-chlorophenylhydrazine **47g** and ethyl acetoacetate **27** according to RP-17.

HPLC trace of crude reaction mixture **48g** (analytical RP-HPLC)

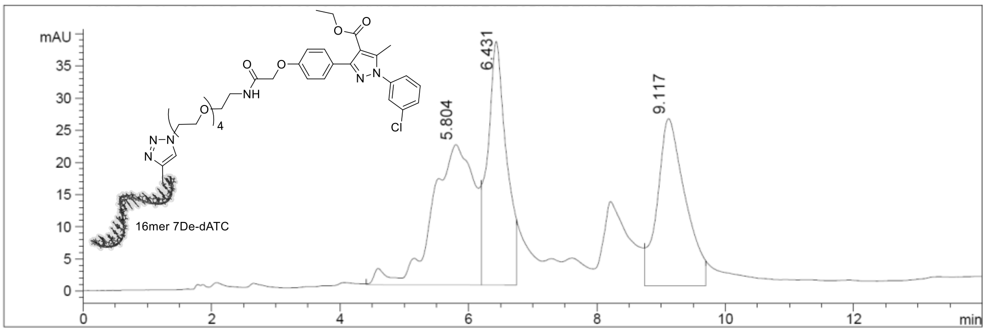

Peak list:

Ret. Time	Width min	Height	Area	Area %
5.804	0.764	21.810	999.686	39.307
6.431	0.335	37.852	759.715	29.872
9.117	0.504	25.925	783.862	30.821

HPLC trace of isolated product **48g** (analytical RP-HPLC)

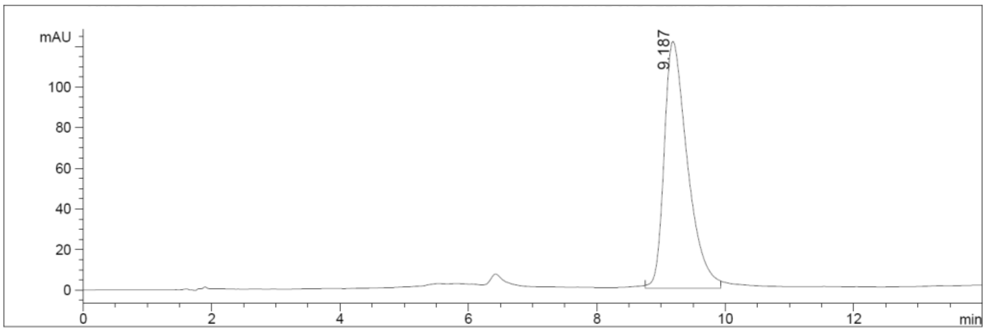

Peak list:

Ret. Time	Width min	Height	Area	Area %
9.187	0.421	121.578	3074.228	100.000

MALDI-MS spectrum of isolated product **48g**

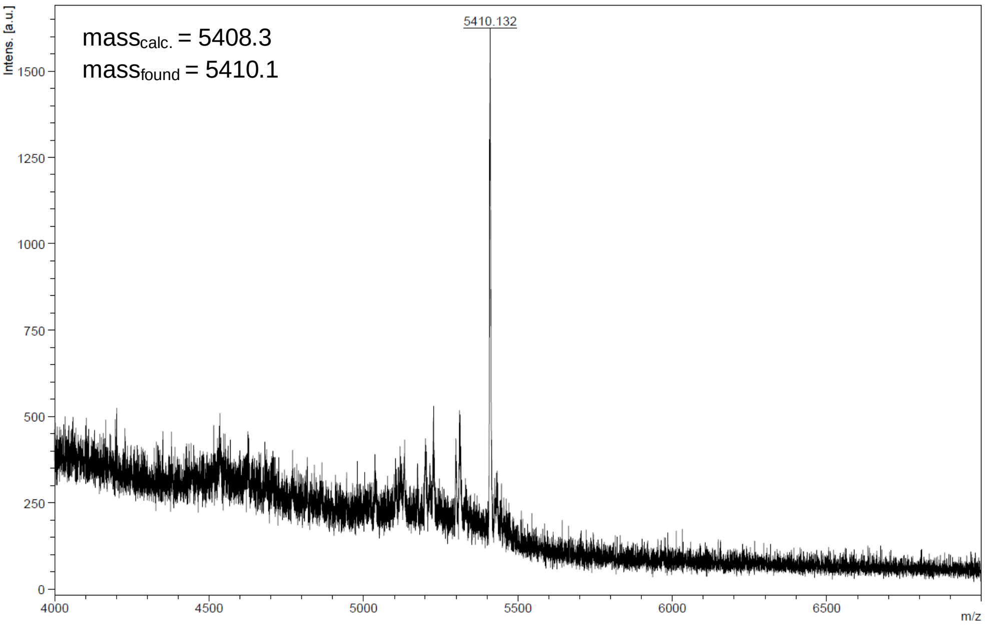

DNA conjugate 48h: CPG-bound 16mer 7De-dATC-aldehyde conjugate **13** (Batch D) was reacted with 2-chlorophenylhydrazine **47h** and ethyl acetoacetate **27** according to RP-17.

HPLC trace of crude reaction mixture **48h** (analytical RP-HPLC)

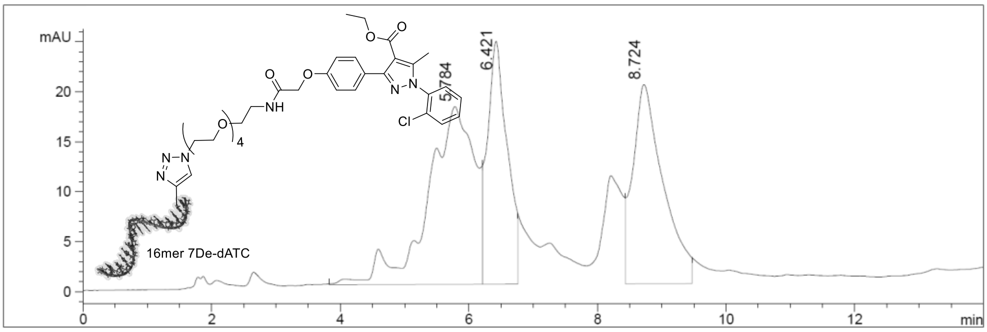

Peak list:

Ret. Time	Width min	Height	Area	Area %
5.784	0.842	17.802	899.453	43.333
6.421	0.356	24.323	520.123	25.058
8.724	0.548	19.955	656.123	31.610

HPLC trace of isolated product **48h** (analytical RP-HPLC)

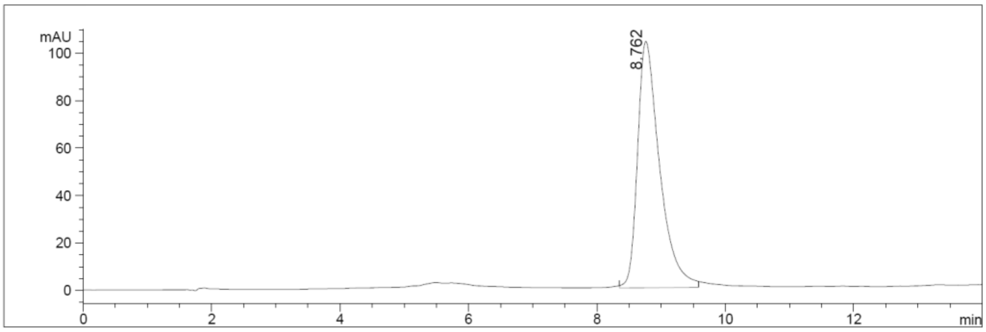

Peak list:

Ret. Time	Width min	Height	Area	Area %
8.762	0.398	104.018	2482.484	100.000

MALDI-MS spectrum of isolated product **48h**

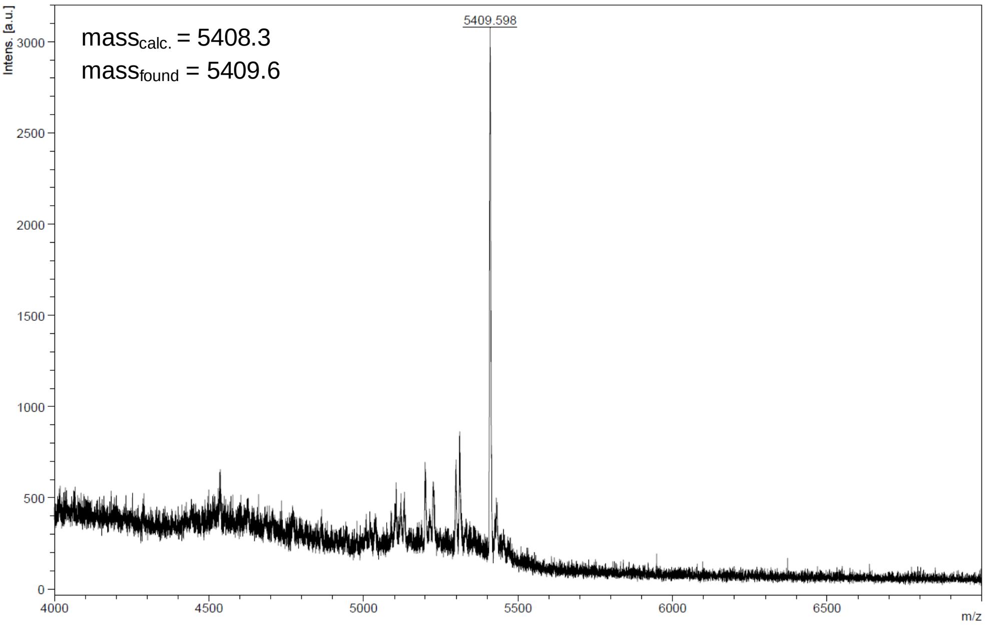

DNA conjugate 48i: CPG-bound 16mer 7De-dATC-aldehyde conjugate **13** (Batch D) was reacted with 2-hydrazinpyrazin **47i** and ethyl acetoacetate **27** according to RP-17.

HPLC trace of crude reaction mixture **48i** (analytical RP-HPLC)

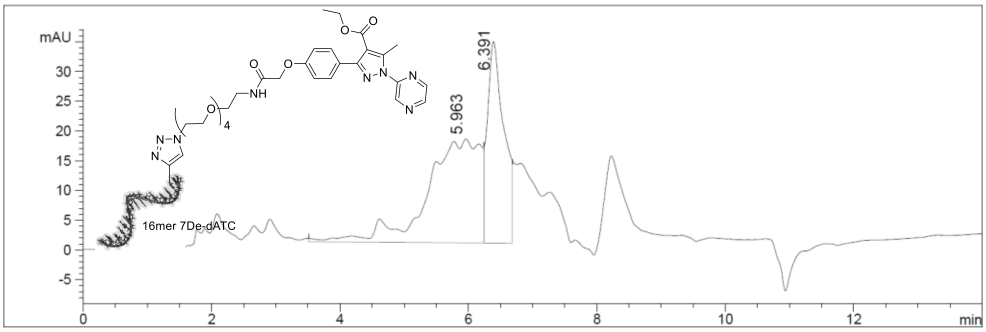


```
Peak list:

Ret. Time   Width min   Height     Area       Area %
------------------------------------------------------
5.963       0.961       17.468     1007.551   62.550
6.391       0.297       33.883     603.245    37.450
------------------------------------------------------
```

MALDI-MS spectrum of isolated product **48i**

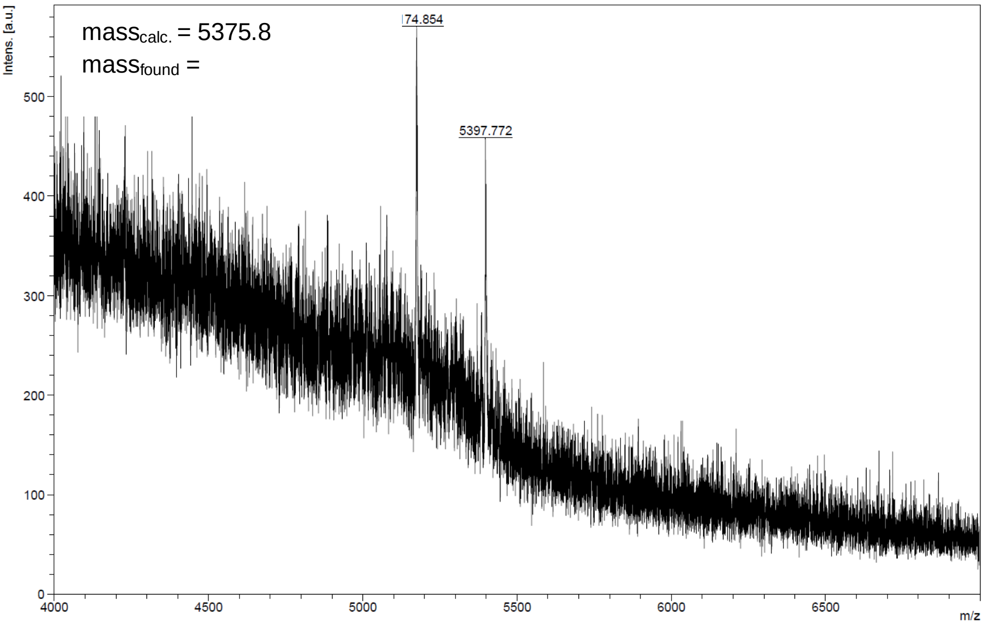

DNA conjugate 48j: CPG-bound 16mer 7De-dATC-aldehyde conjugate **13** (Batch D) was reacted with 4-methoxyphenylhydrazine **47j** and ethyl acetoacetate **27** according to RP-17.

HPLC trace of crude reaction mixture **48j** (analytical RP-HPLC)

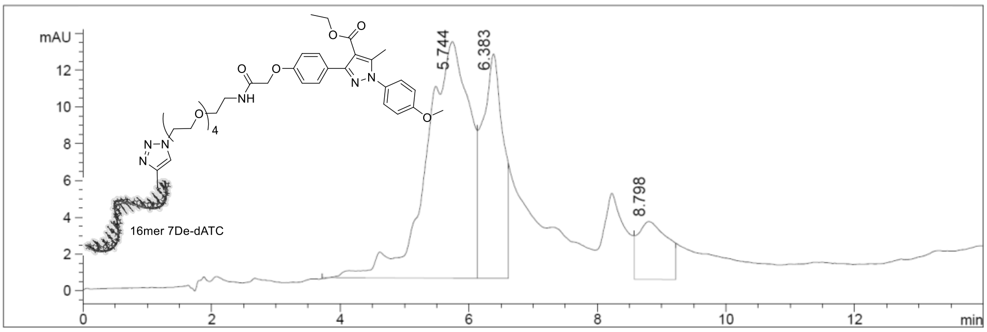

Peak list:

Ret. Time	Width min	Height	Area	Area %
5.744	0.785	12.865	605.765	62.022
6.383	0.368	12.203	269.473	27.590
8.798	0.540	3.133	101.455	10.388

HPLC trace of isolated product **48j** (analytical RP-HPLC)

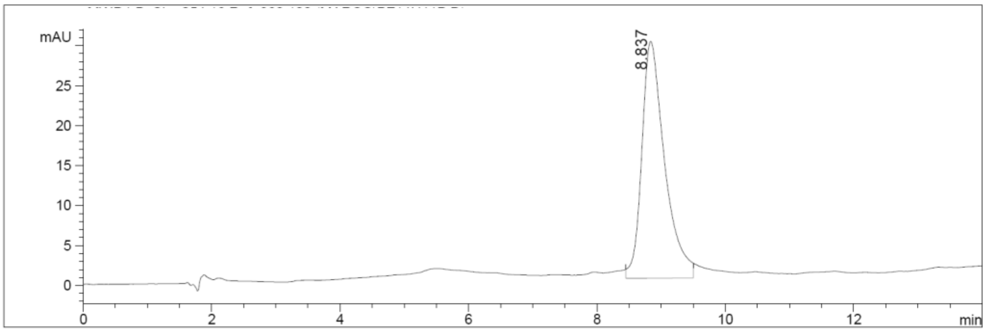

Peak list:

Ret. Time	Width min	Height	Area	Area %
8.837	0.412	29.629	732.109	100.000

MALDI-MS spectrum of isolated product **48j**

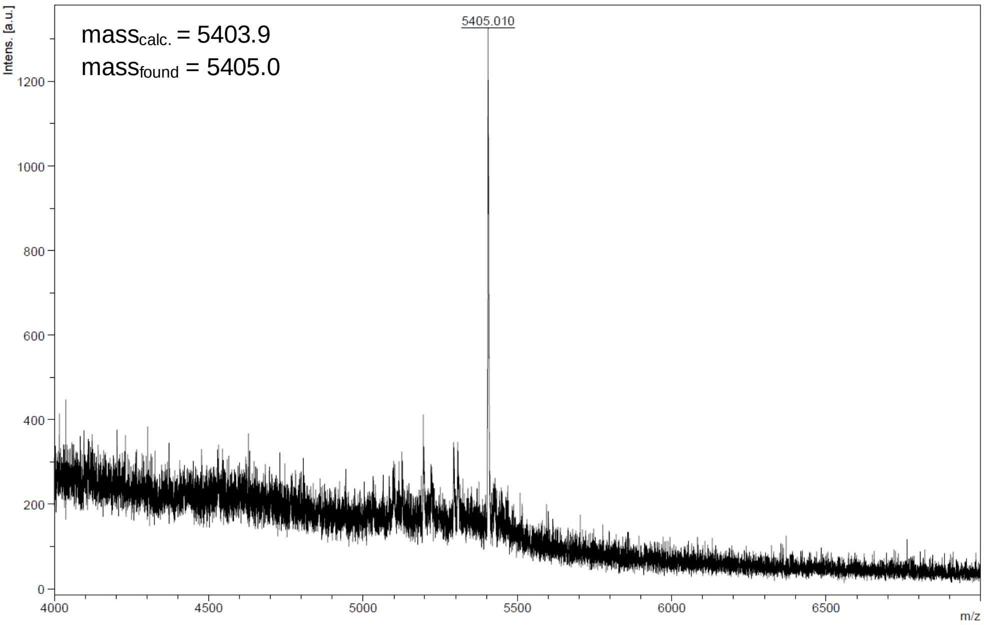

DNA conjugate 48k: CPG-bound 16mer 7De-dATC-aldehyde conjugate **13** (Batch D) was reacted with 4-(trifluoromethoxy)phenylhydrazine **47k** and ethyl acetoacetate **27** according to RP-17.

HPLC trace of crude reaction mixture **48k** (analytical RP-HPLC)

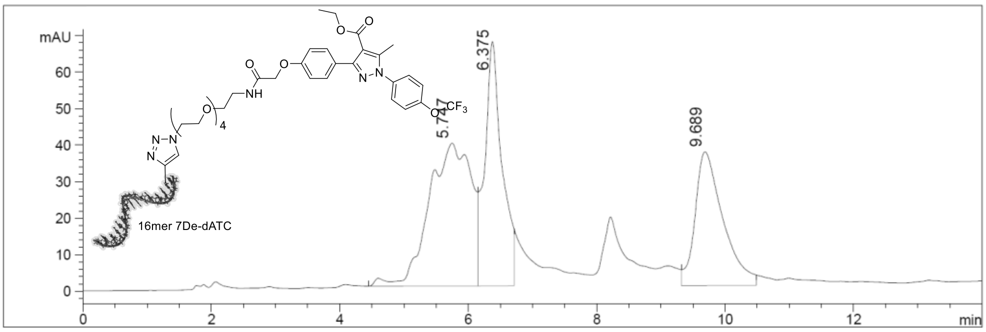

Peak list:

Ret. Time	Width min	Height	Area	Area %
5.747	0.762	39.069	1785.672	43.099
6.375	0.312	66.879	1251.458	30.205
9.689	0.504	36.587	1106.072	26.696

HPLC trace of isolated product **48k** (analytical RP-HPLC)

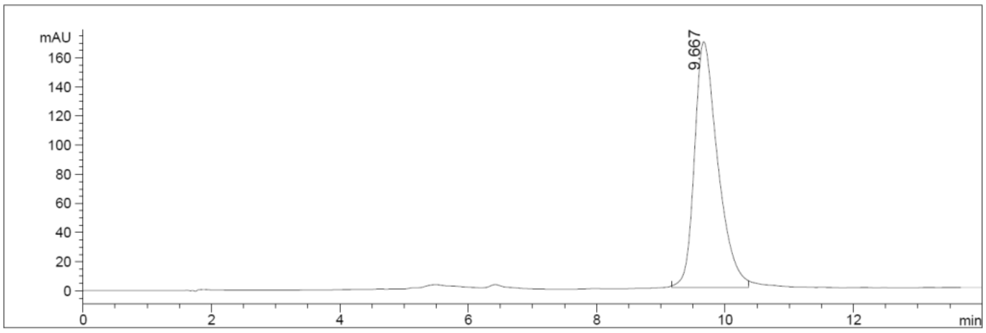

Peak list:

Ret. Time	Width min	Height	Area	Area %
9.667	0.423	168.749	4277.805	100.000

MALDI-MS spectrum of isolated product **48k**

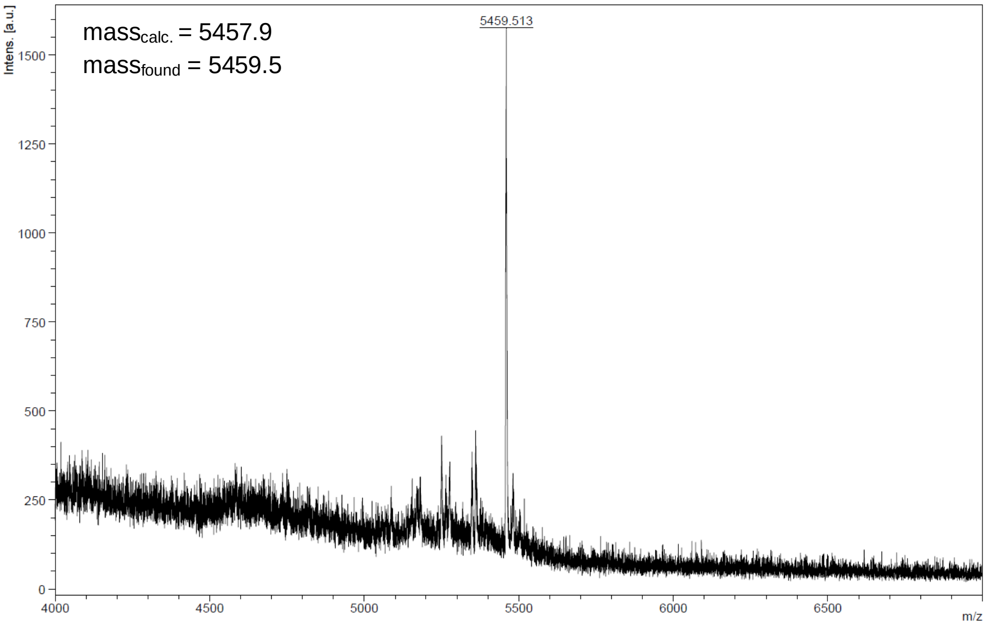

Au(I)/Ag(I)-promoted pyrazoline-containing spiroheterocycle synthesis

DNA conjugate 51: CPG-bound 16mer 7De-dATC-aldehyde conjugate **13** (Batch B) was reacted with pent-4-yn-1-ol **49** and *tert*-butyl 2-benzylhydrazine-carboxylate **50** according to RP-18.

HPLC trace of crude reaction mixture **51** (analytical RP-HPLC)

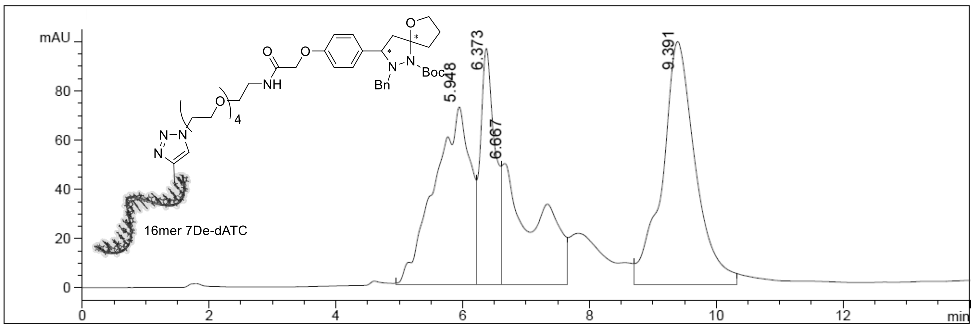

Peak list:

Ret. Time	Width min	Height	Area	Area %
5.948	0.655	71.978	2828.204	28.921
6.373	0.273	95.770	1570.882	16.064
6.667	0.613	49.011	1801.821	18.425
9.391	0.606	98.465	3578.145	36.590

HPLC trace of isolated product **51** (analytical RP-HPLC)

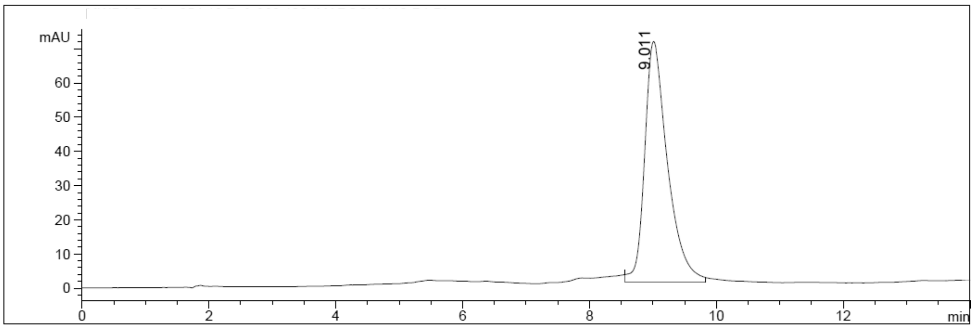

Peak list:

Ret. Time	Width min	Height	Area	Area %
9.011	0.419	70.358	1767.636	100.000

MALDI-MS spectrum of isolated product **51**

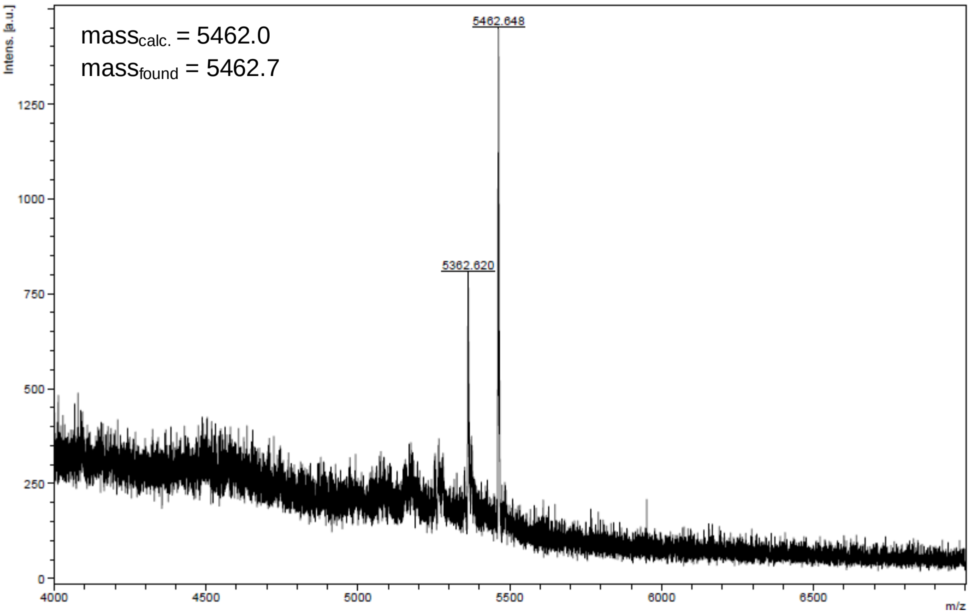

Au(I)/Ag(I)-promoted pyrazoline synthesis

Table S15 – Scope of Au(I)/Ag(I)-promoted pyrazoline synthesis on CPG-bound 16mer 7De-dATC oligonucleotide-alkyne conjugate **52** using different aldehydes **34**.[a]

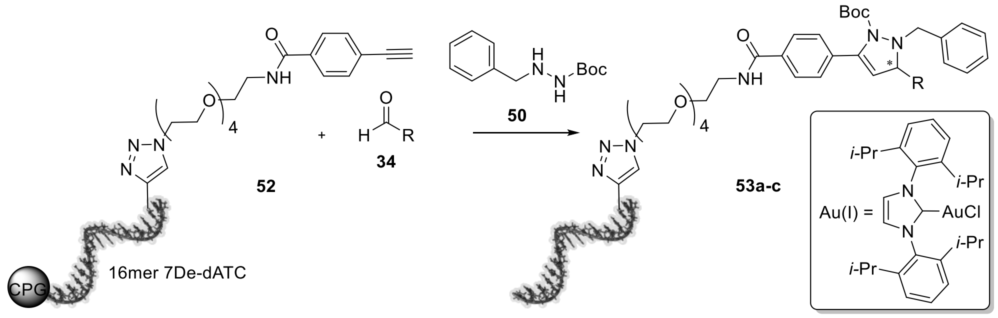

Entry	Product	Hydrazide	Conversion [%][b]	DNA degradation [%][c]	$Mass_{calc.}$ $Mass_{found}$[d]
1	**53a**	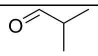 **34s**	85	<5	5416.0<br>5415.0
2	**53b**	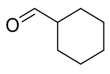 **34r**	87	11	5456.1<br>5454.4
3[e]	**53c**	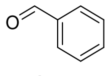 **34a**	88	14	5450.0<br>5448.4

[a] CPG-bound oligonucleotide conjugate **52** (20 nmol), aldehyde **34** (1000 equiv., 20 µmol), *tert*-butyl 2-benzylhydrazinecarboxylate **50** (1000 equiv.,20 µmol) and Au(I)/AgOTf (250 equiv., 5 µmol) in 50 µL in acetonitrile at 50 °C. DNA cleavage with AMA (30% aqueous ammonia / 40% aqueous methylamine, 1:1 (vol/vol)) at ambient temperature for 4 h. [b] Determined by analytical RP-HPLC analysis based on the ratios of **53** to **52**. [c] Determined by comparison of purities of the analytical RP-HPLC traces of starting material **52** and the crude reaction mixture **53**. [d] Measured by MALDI-MS. [e] Reaction was performed in glacial acetic acid instead of acetonitrile. 7De-dATC = 5'-CT*C TCT TT7De-dA 7De-dACT 7De-dACC T-3'.

DNA conjugate 52: CPG-bound 16mer 7De-dATC-PEG(4)-NH_2 conjugate was reacted with 4-ethynylbenzoic acid according to RP-04.

HPLC trace of crude reaction mixture **52** (analytical RP-HPLC)

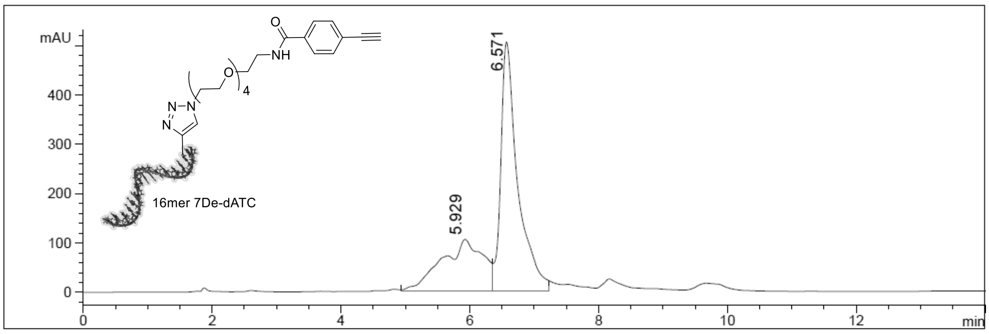

Peak list:

Ret. Time	Width min	Height	Area	Area %
5.929	0.743	104.156	4646.222	33.665
6.571	0.302	504.528	9155.146	66.335

MALDI-MS spectrum of crude reaction mixture **52**

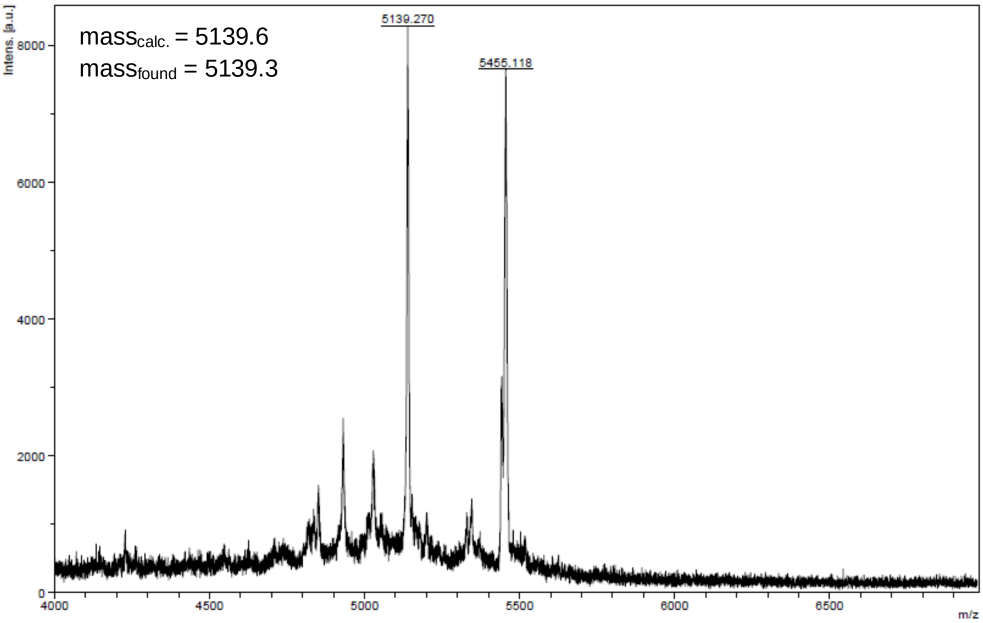

Under the condition of DNA deprotection, likely water is added to the alkyne, yielding a ketone or aldehyde. This reaction explains the second peak in the mass spectrum.

DNA conjugate 53a: CPG-bound 16mer 7De-dATC-alkyne conjugate **52** was reacted with isobutyraldehyde **34s** and *tert*-butyl 2-benzylhydrazine-carboxylate **50** according to RP-19.

HPLC trace of crude reaction mixture **53a** (analytical RP-HPLC)

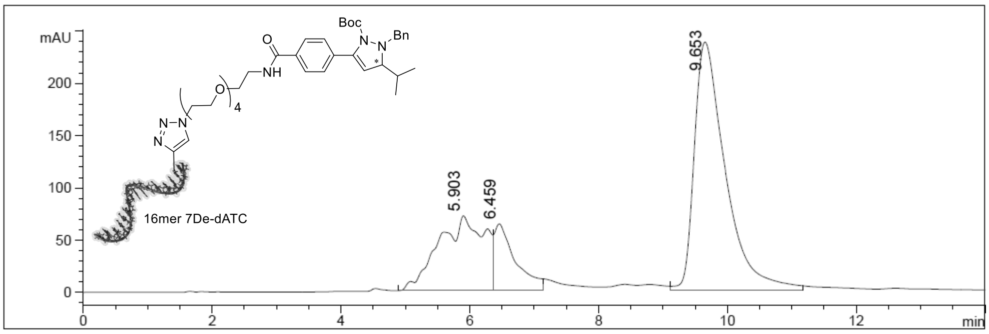

Peak list:

Ret. Time	Width min	Height	Area	Area %
5.903	0.828	71.525	3553.703	27.288
6.459	0.380	63.881	1456.983	11.188
9.653	0.562	237.692	8012.460	61.525

HPLC trace of isolated product **53a** (analytical RP-HPLC)

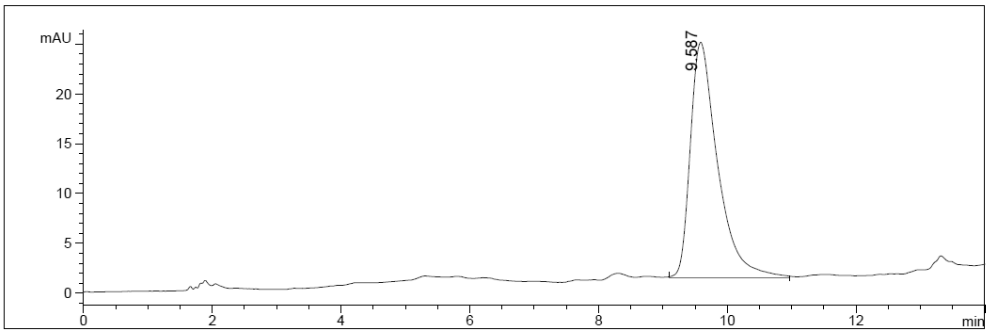

Peak list:

Ret. Time	Width min	Height	Area	Area %
9.587	0.494	23.657	701.268	100.000

MALDI-MS spectrum of isolated product **53a**

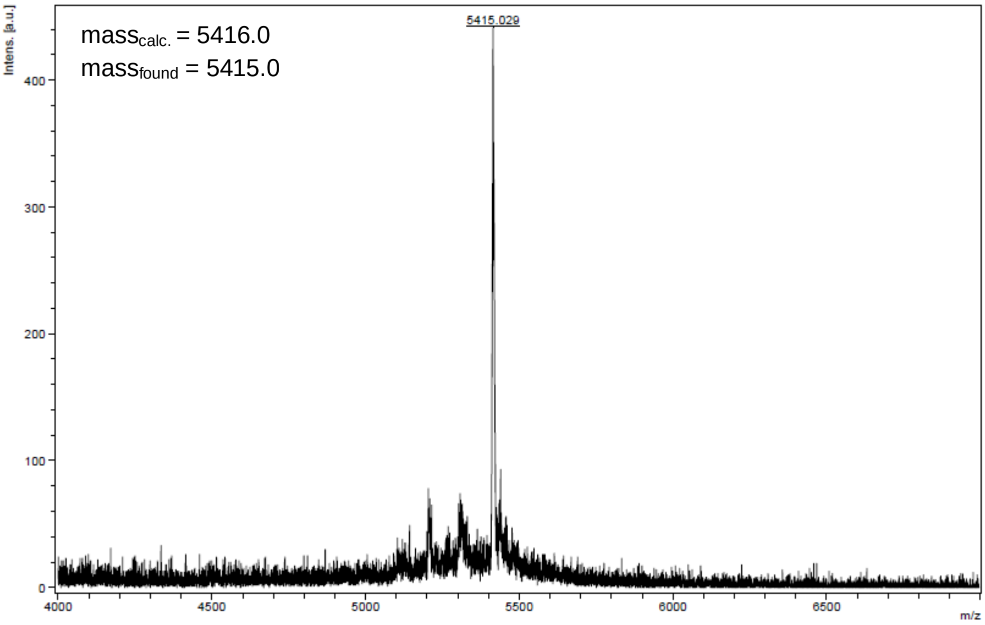

DNA conjugate 53b: CPG-bound 16mer 7De-dATC-alkyne conjugate **52** was reacted with cyclohexanecarboxaldehyde **34r** and *tert*-butyl 2-benzylhydrazine-carboxylate **50** according to RP-19.

HPLC trace of crude reaction mixture **53b** (analytical RP-HPLC)

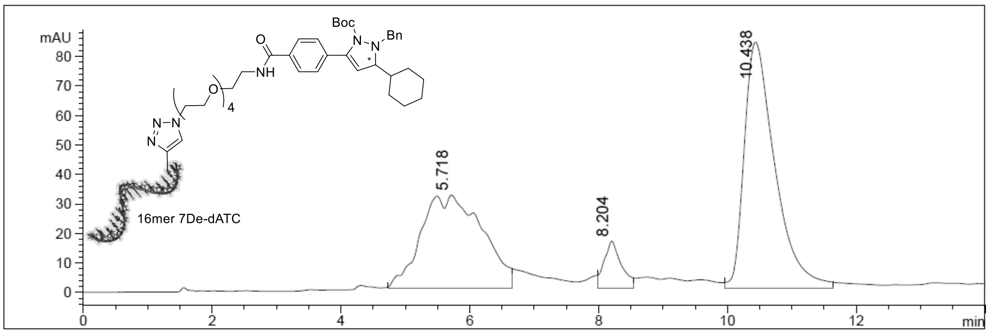

Peak list:

Ret. Time	Width min	Height	Area	Area %
5.718	1.113	31.711	2118.429	40.722
8.204	0.310	16.133	300.159	5.770
10.438	0.555	83.549	2783.553	53.508

HPLC trace of isolated product **53b** (analytical RP-HPLC)

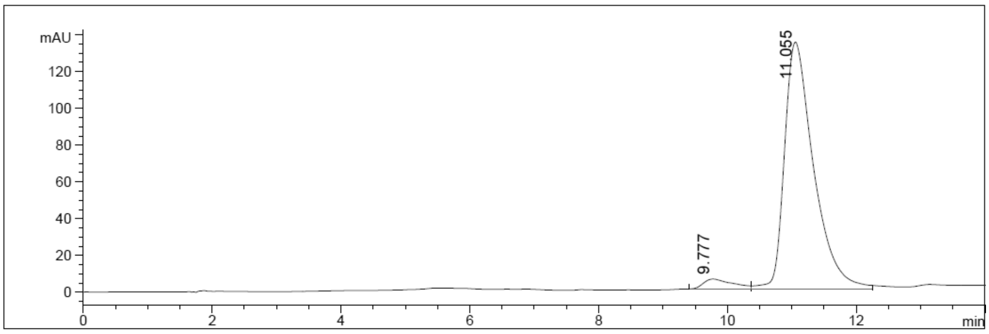

Peak list:

Ret. Time	Width min	Height	Area	Area %
9.777	0.541	5.629	182.787	4.116
11.055	0.528	134.410	4257.947	95.884

MALDI-MS spectrum of isolated product **53b**

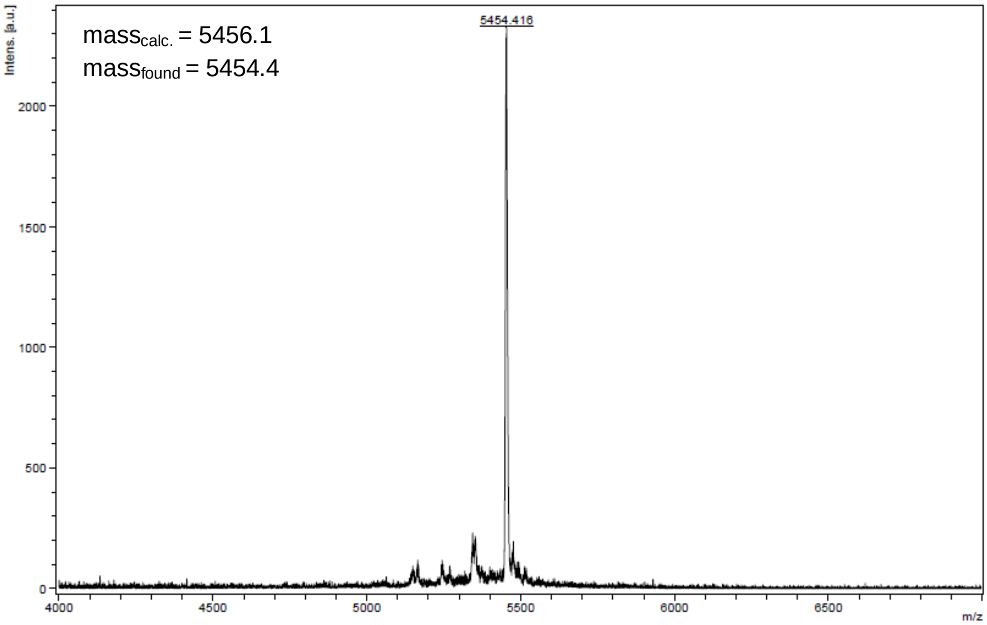

DNA conjugate 53c: CPG-bound 16mer 7De-dATC-alkyne conjugate **52** was reacted with benzaldehyde **34a** and *tert*-butyl 2-benzylhydrazine-carboxylate **50** according to RP-19.

HPLC trace of crude reaction mixture **53c** (analytical RP-HPLC)

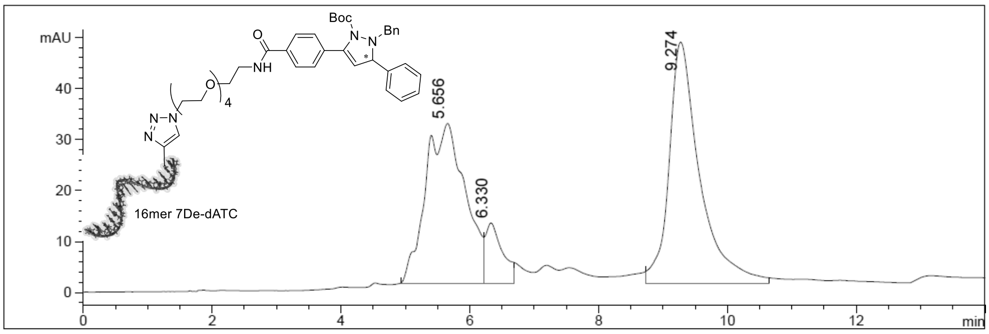

Peak list:

Ret. Time	Width min	Height	Area	Area %
5.656	0.714	31.289	1341.340	42.983
6.330	0.309	11.827	219.456	7.032
9.274	0.551	47.216	1559.808	49.984

HPLC trace of isolated product **53c** (analytical RP-HPLC)

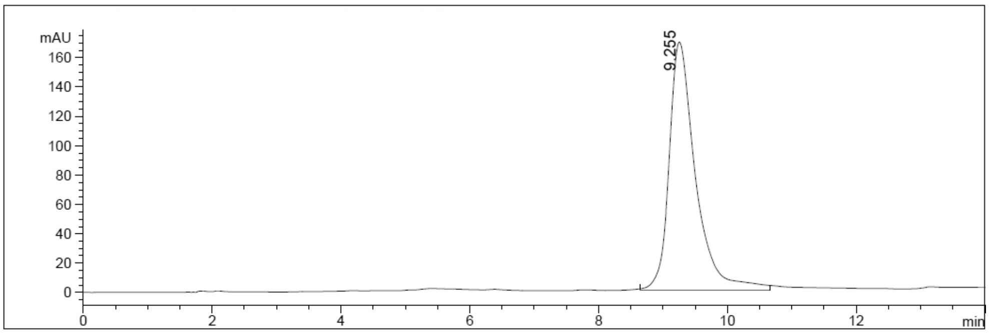

Peak list:

Ret. Time	Width min	Height	Area	Area %
9.255	0.475	168.953	4810.911	100.000

MALDI-MS spectrum of isolated product **53c**

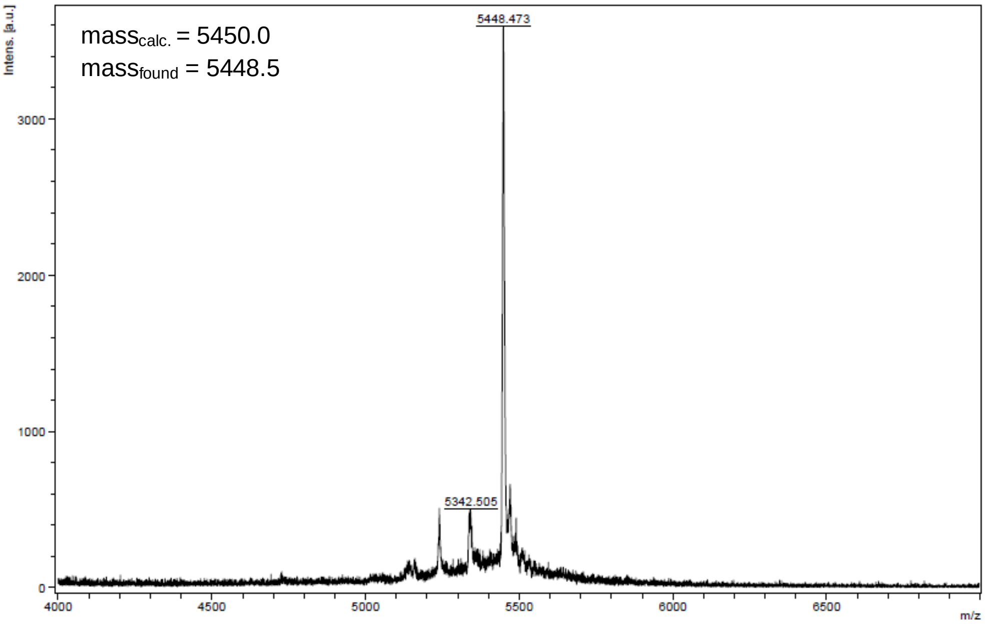

Au(I)/Ag(I)-promoted pyrazole synthesis

Table S16 – Scope of Au(I)/Ag(I)-promoted pyrazole synthesis on CPG-bound 16mer 7De-dATC oligonucleotide-alkyne conjugate **52** using different aldehydes **34**.[a]

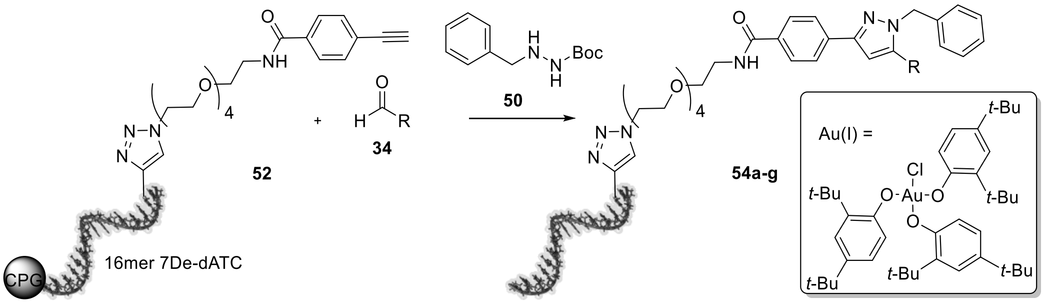

Entry	Product	Hydrazide	Conversion [%][b]	DNA degradation [%][c]	$Mass_{calc.}$ $Mass_{found}$[d]
1	**54a**	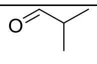 **34s**	63	<5	5313.8 5309.9
2	**54b**	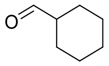 **34r**	53	14	5353.9 5351.4
3	**54c**	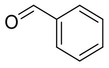 **34a**	88	8	5347.9 5350.5
4	**54d**	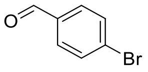 **34d**	69	14	5426.8 5429.3
5	**54e**	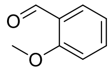 **34e**	60	9	5377.9 5372.4
6	**54f**	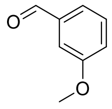 **34f**	61	9	5377.9 5374.0
7	**54g**	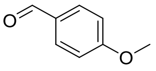 **34g**	77	12	5377.9 5381.2

[a] CPG-bound oligonucleotide conjugate **52** (20 nmol), aldehyde **34** (1000 equiv., 20 µmol), *tert*-butyl 2-benzylhydrazinecarboxylate **50** (1000 equiv.,20 µmol) and Au(I)/AgOTf (250 equiv., 5 µmol) in 50 µL in glacial acetic acid at 60 °C. DNA cleavage with AMA (30% aqueous ammonia / 40% aqueous methylamine, 1:1 (vol/vol)) at ambient temperature for 4 h. [b] Determined by analytical RP-HPLC analysis based on the ratios of **53** to **52**. [c] Determined by comparison of purities of the analytical RP-HPLC traces of starting material **52** and the crude reaction mixture **53**. [d] Measured by MALDI-MS. 7De-dATC = 5'-CT*C TCT TT7De-dA 7De-dACT 7De-dACC T-3'.

DNA conjugate 52: CPG-bound 16mer 7De-dATC-PEG(4)-NH_2 conjugate was reacted with 4-ethynylbenzoic acid according to RP-04.

HPLC trace of crude reaction mixture **52** (analytical RP-HPLC)

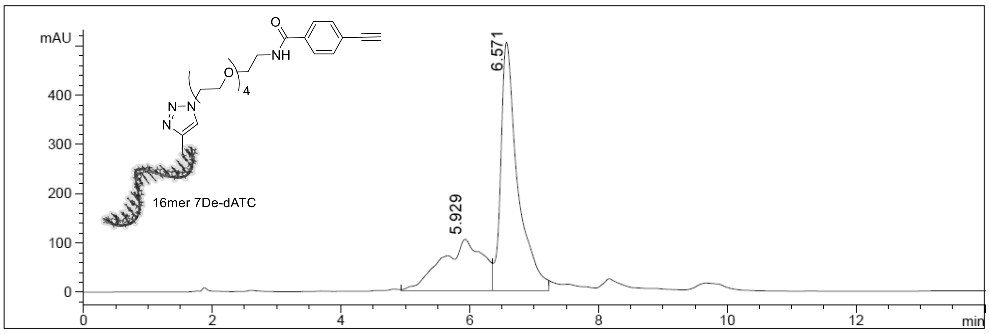

Peak list:

Ret. Time	Width min	Height	Area	Area %
5.929	0.743	104.156	4646.222	33.665
6.571	0.302	504.528	9155.146	66.335

MALDI-MS spectrum of crude reaction mixture **52**

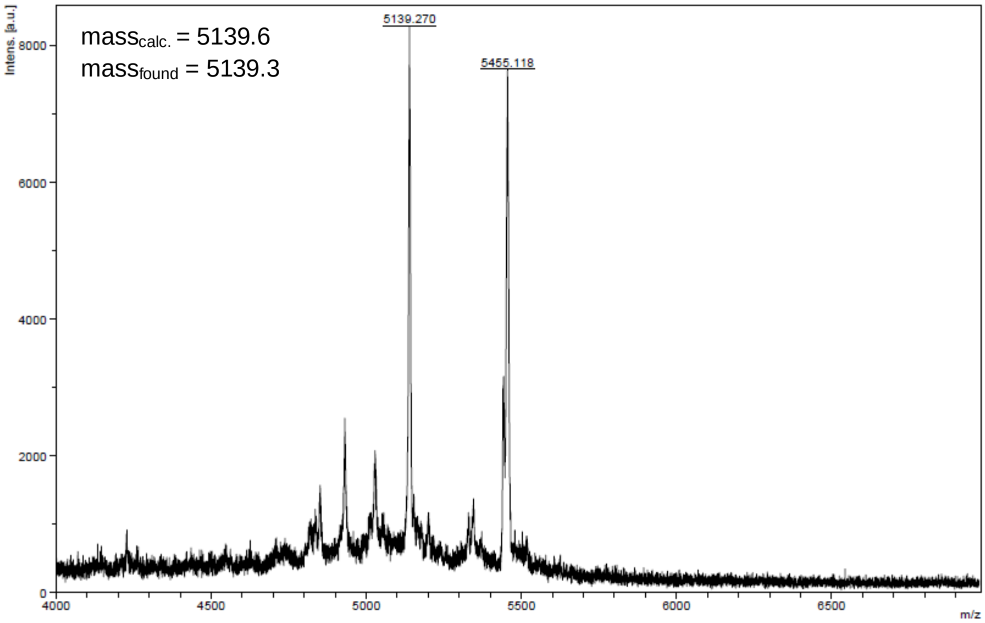

DNA conjugate 54a: CPG-bound 16mer 7De-dATC-alkyne conjugate **52** was reacted with isobutyraldehyde **34s** and *tert*-butyl 2-benzylhydrazine-carboxylate **50** according to RP-19.

HPLC trace of crude reaction mixture **54a** (analytical RP-HPLC)

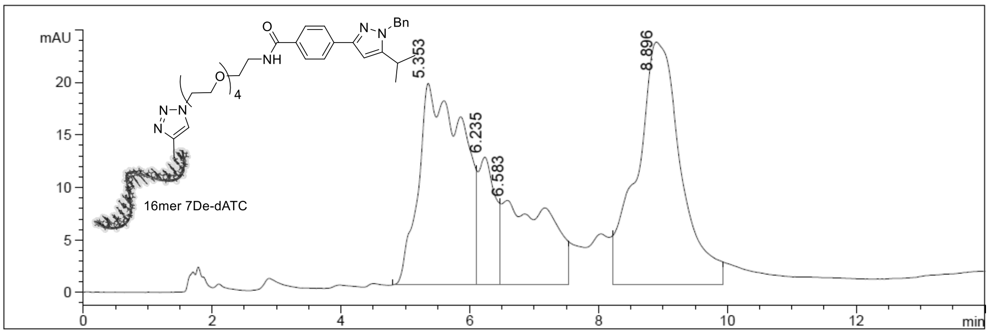

Peak list:

Ret. Time	Width min	Height	Area	Area %
5.353	0.792	19.111	907.923	34.472
6.235	0.303	12.068	219.548	8.336
6.583	0.868	7.949	414.116	15.723
8.896	0.791	23.027	1092.211	41.469

HPLC trace of isolated product **54a** (analytical RP-HPLC)

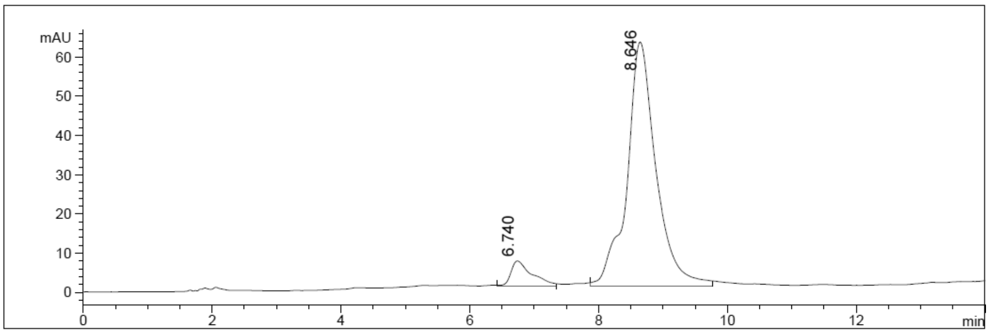

Peak list:

Ret. Time	Width min	Height	Area	Area %
6.740	0.370	6.313	140.337	6.752
8.646	0.520	62.082	1938.087	93.248

MALDI-MS spectrum of isolated product **54a**

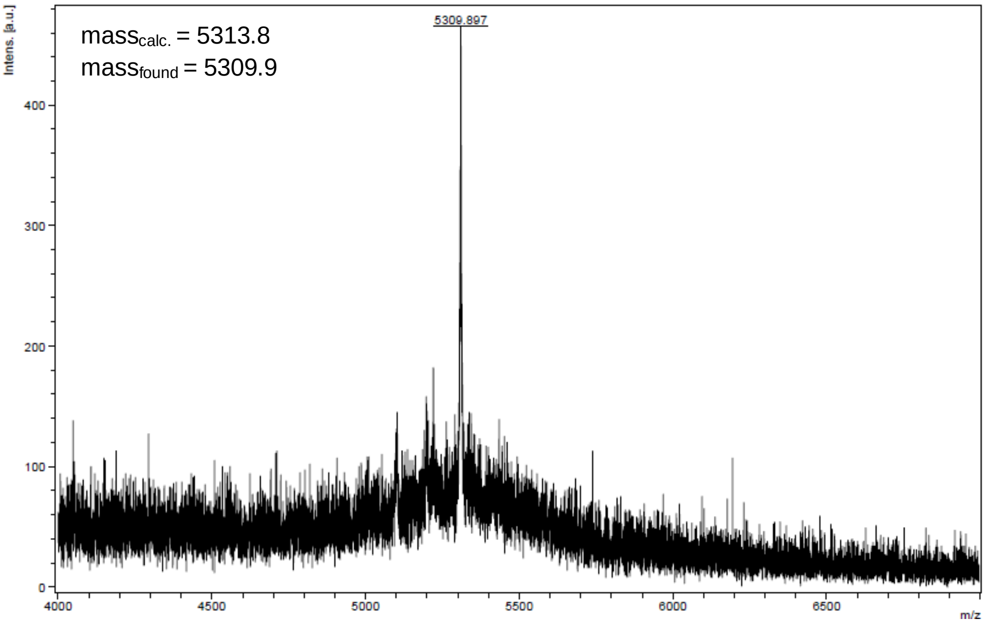

DNA conjugate 54b: CPG-bound 16mer 7De-dATC-alkyne conjugate **52** was reacted with cyclohexanecarboxaldehyde **34r** and *tert*-butyl 2-benzylhydrazine-carboxylate **50** according to RP-19.

HPLC trace of crude reaction mixture **54b** (analytical RP-HPLC)

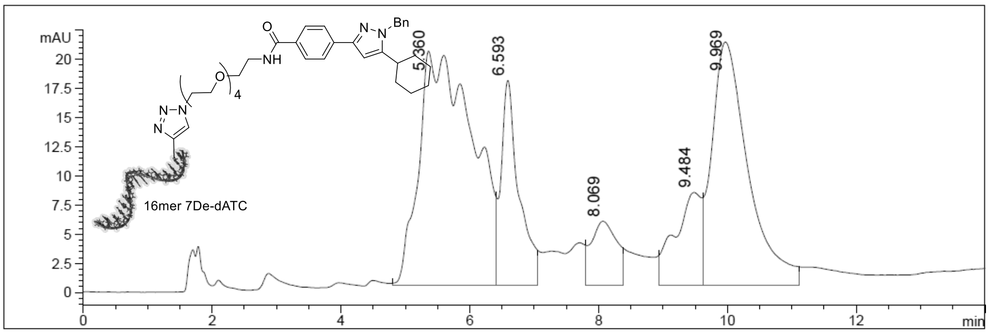


```
Peak list:

Ret. Time   Width min   Height     Area       Area %
----------------------------------------------------------
5.360       0.967       20.069     1164.920   43.238
6.593       0.322       17.549     338.655    12.570
8.069       0.458       5.469      150.139    5.573
9.484       0.476       7.940      226.923    8.423
9.969       0.651       20.830     813.593    30.198
----------------------------------------------------------
```

HPLC trace of isolated product **54b** (analytical RP-HPLC)

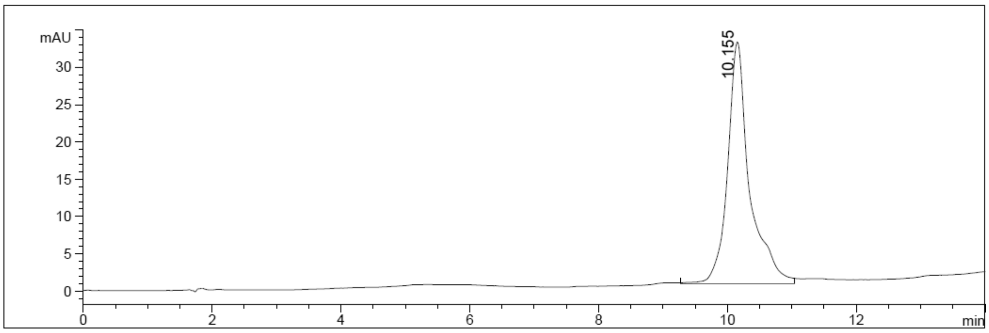

```
Peak list:

Ret. Time   Width min   Height     Area       Area %
----------------------------------------------------------
10.155      0.394       32.359     764.173    100.000
----------------------------------------------------------
```

MALDI-MS spectrum of isolated product **54b**

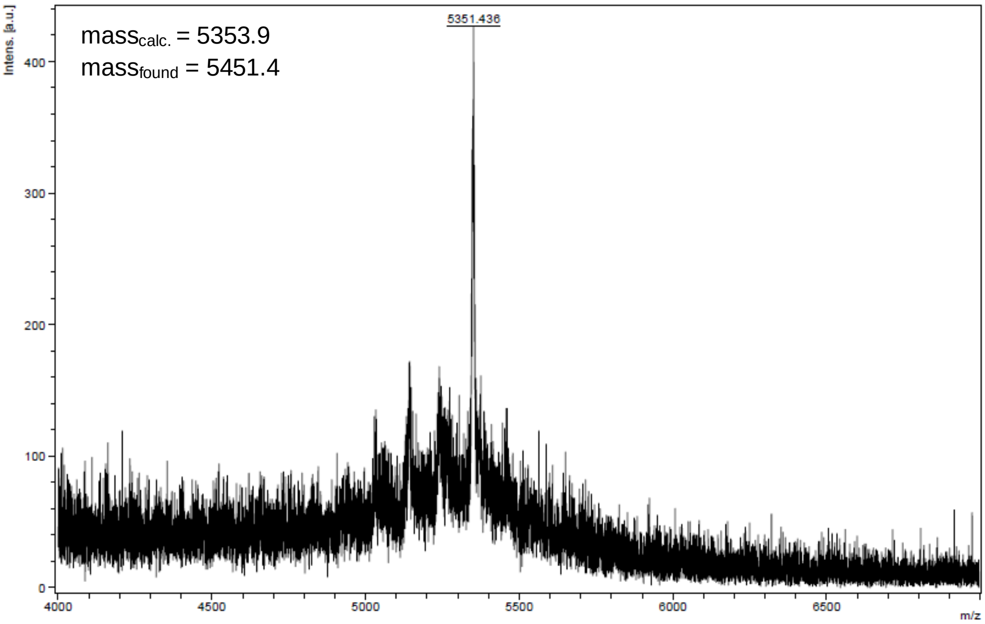

DNA conjugate 54c: CPG-bound 16mer 7De-dATC-alkyne conjugate **52** was reacted with benzaldehyde **34a** and *tert*-butyl 2-benzylhydrazine-carboxylate **50** according to RP-20.

HPLC trace of crude reaction mixture **54c** (analytical RP-HPLC)

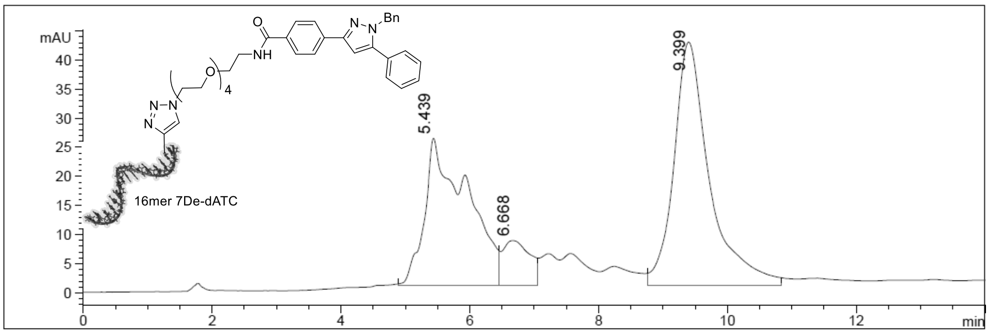

Peak list:

Ret. Time	Width min	Height	Area	Area %
5.439	0.767	25.246	1161.898	38.816
6.668	0.496	7.661	227.949	7.615
9.399	0.640	41.766	1603.498	53.569

HPLC trace of isolated product **54c** (analytical RP-HPLC)

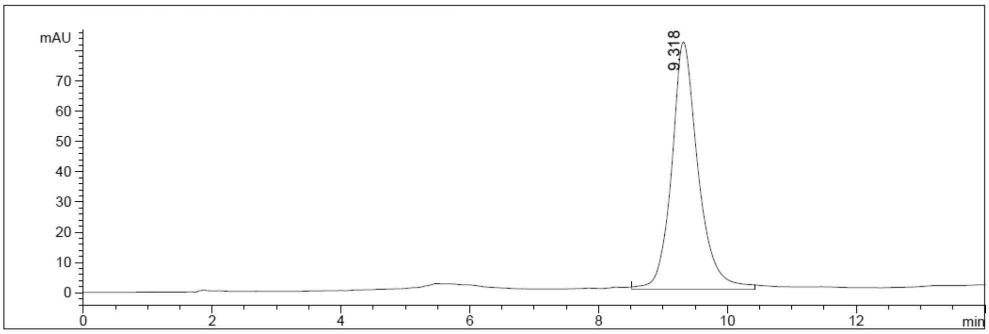

Peak list:

Ret. Time	Width min	Height	Area	Area %
9.318	0.477	81.696	2337.747	100.000

MALDI-MS spectrum of isolated product **54c**

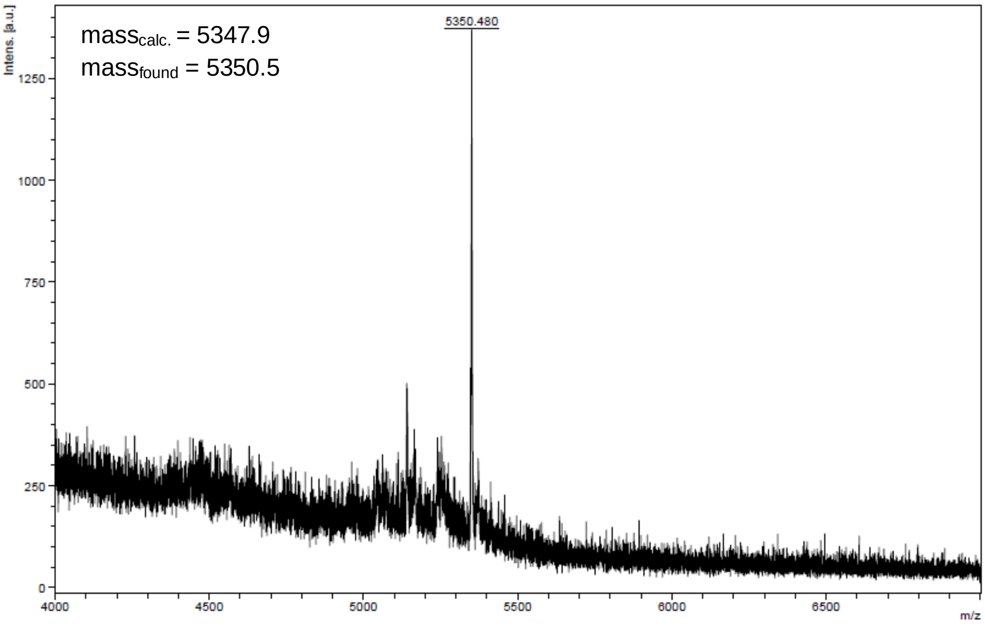

DNA conjugate 54d: CPG-bound 16mer 7De-dATC-alkyne conjugate **52** was reacted with 4-bromobenzaldehyde **34d** and *tert*-butyl 2-benzylhydrazine-carboxylate **50** according to RP-20.

HPLC trace of crude reaction mixture **54d** (analytical RP-HPLC)

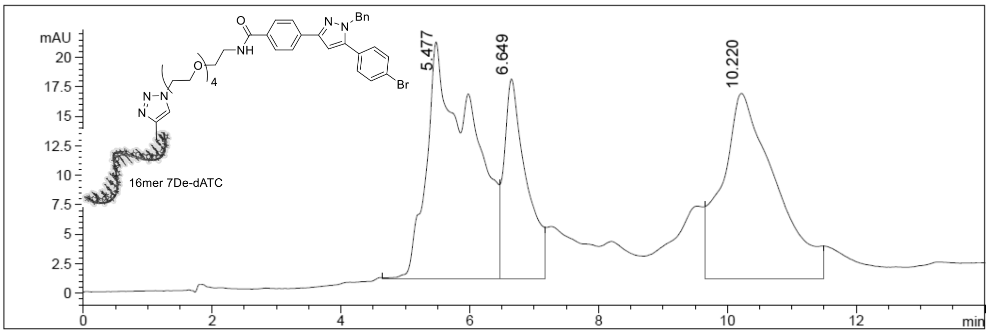

Peak list:

Ret. Time	Width min	Height	Area	Area %
5.477	0.813	20.148	982.209	42.615
6.649	0.404	17.008	412.096	17.880
10.220	0.962	15.783	910.525	39.505

HPLC trace of isolated product **54d** (analytical RP-HPLC)

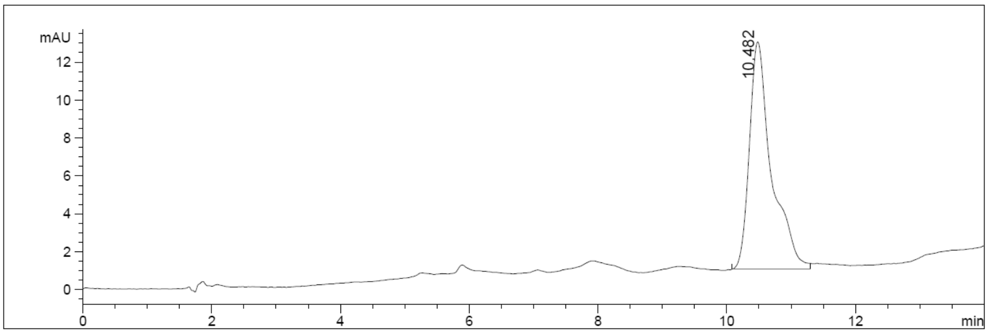

Peak list:

Ret. Time	Width min	Height	Area	Area %
10.482	0.397	11.989	285.902	100.000

MALDI-MS spectrum of isolated product **54d**

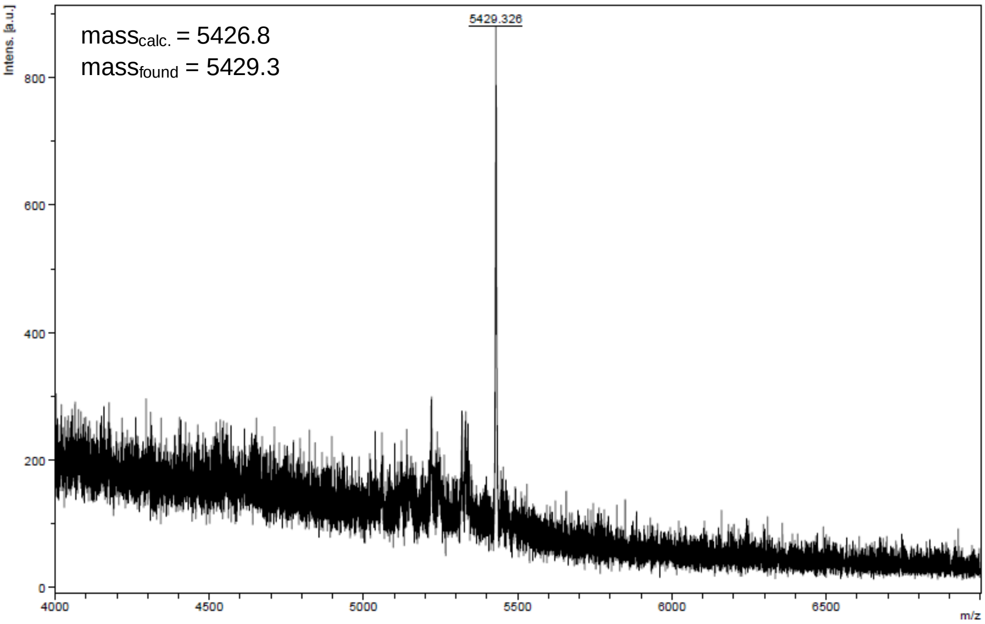

DNA conjugate 54e: CPG-bound 16mer 7De-dATC-alkyne conjugate **52** was reacted with *o*-anisaldehyde **34e** and *tert*-butyl 2-benzylhydrazine-carboxylate **50** according to RP-20.

HPLC trace of crude reaction mixture **54e** (analytical RP-HPLC)

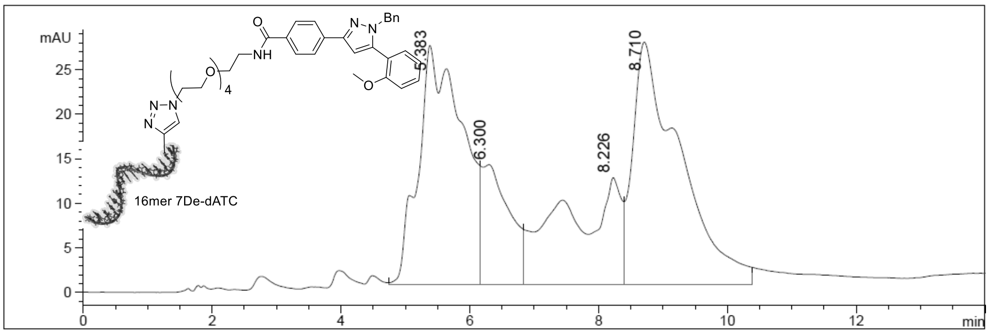

Peak list:

Ret. Time	Width min	Height	Area	Area %
5.383	0.794	26.817	1277.382	33.636
6.300	0.510	13.443	411.421	10.834
8.226	0.996	11.981	715.986	18.854
8.710	0.854	27.191	1392.830	36.676

HPLC trace of isolated product **54e** (analytical RP-HPLC)

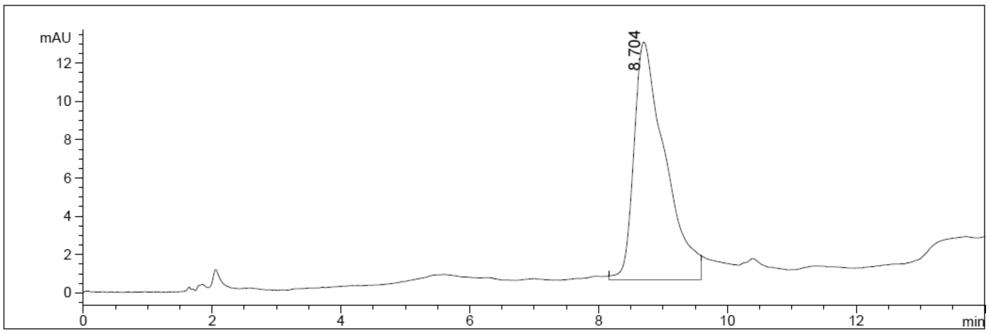

Peak list:

Ret. Time	Width min	Height	Area	Area %
8.704	0.561	12.408	417.761	100.000

MALDI-MS spectrum of isolated product **54e**

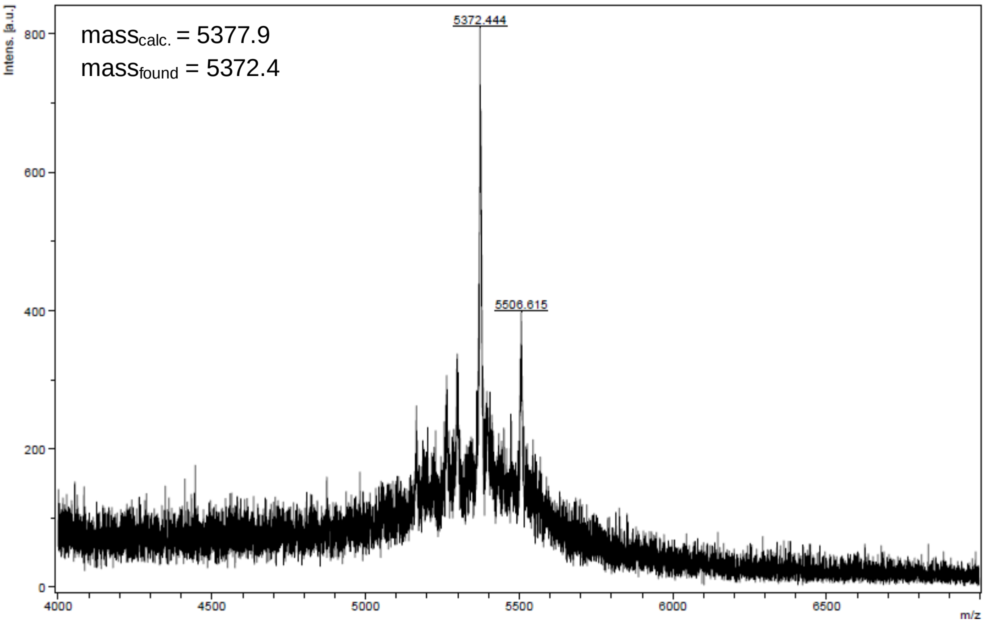

DNA conjugate 54f: CPG-bound 16mer 7De-dATC-alkyne conjugate **52** was reacted with *m*-anisaldehyde **34f** and *tert*-butyl 2-benzylhydrazine-carboxylate **50** according to RP-20.

HPLC trace of crude reaction mixture **54f** (analytical RP-HPLC)

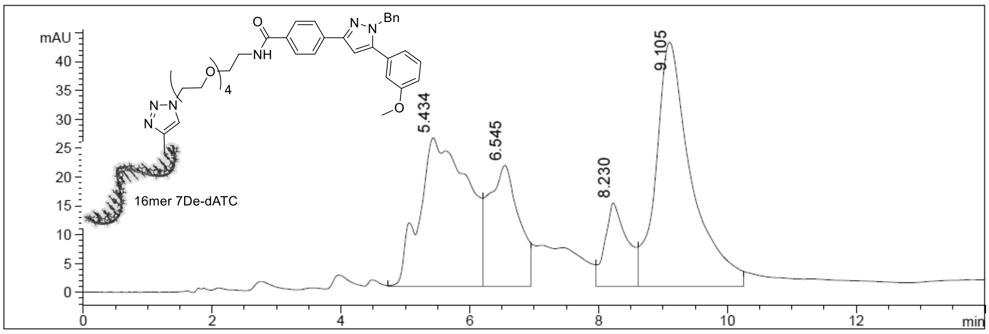

Peak list:

Ret. Time	Width min	Height	Area	Area %
5.434	0.873	25.707	1347.185	33.917
6.545	0.534	20.945	671.432	16.904
8.230	0.404	14.446	350.421	8.822
9.105	0.631	42.316	1602.960	40.357

HPLC trace of isolated product **54f** (analytical RP-HPLC)

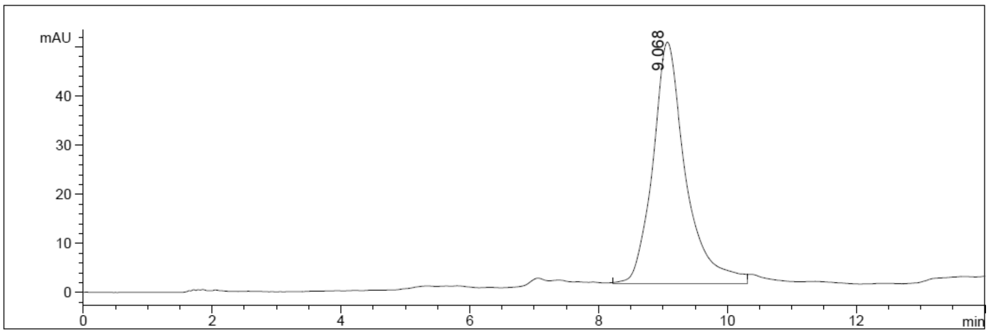

Peak list:

Ret. Time	Width min	Height	Area	Area %
9.068	0.591	49.164	1743.168	100.000

MALDI-MS spectrum of isolated product **54f**

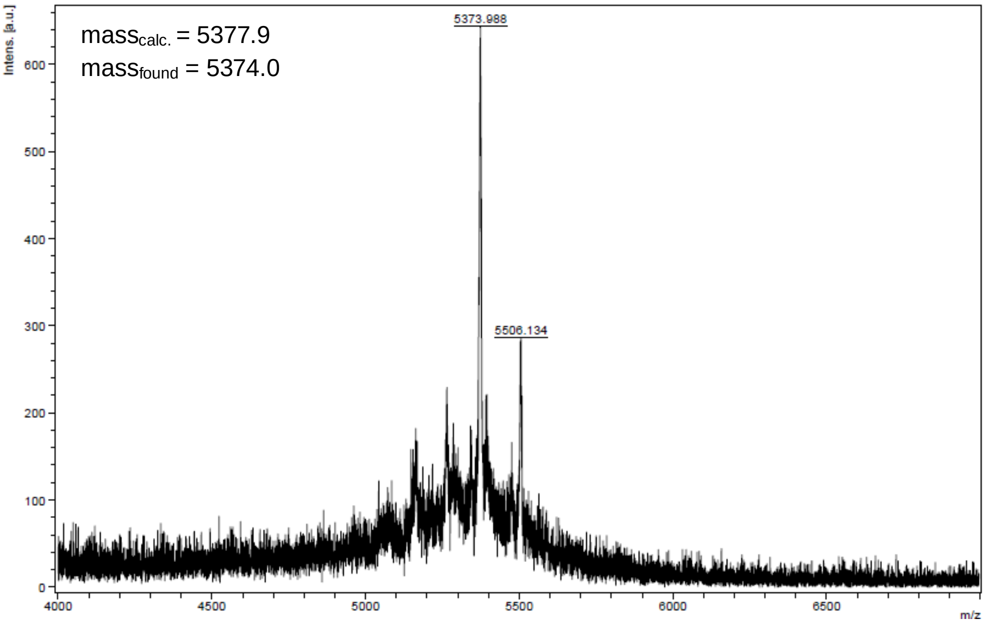

DNA conjugate 54g: CPG-bound 16mer 7De-dATC-alkyne conjugate **52** was reacted with *p*-anisaldehyde **34g** and *tert*-butyl 2-benzylhydrazine-carboxylate **50** according to RP-20.

HPLC trace of crude reaction mixture **54g** (analytical RP-HPLC)

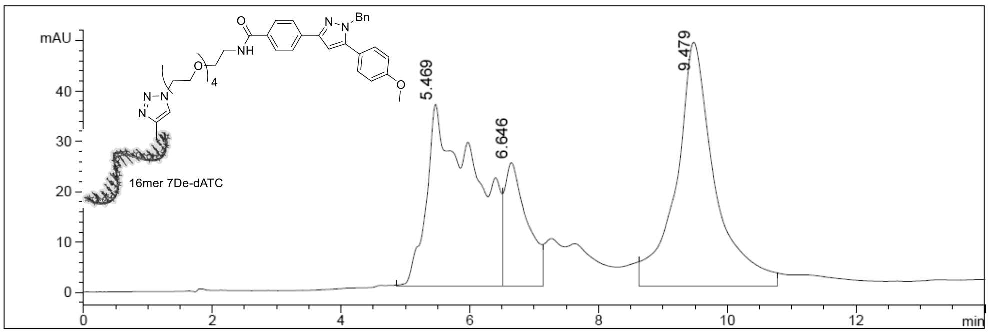

Peak list:

Ret. Time	Width min	Height	Area	Area %
5.469	0.872	36.069	1887.292	41.998
6.646	0.401	24.481	589.463	13.117
9.479	0.695	48.397	2016.981	44.884

HPLC trace of isolated product **54g** (analytical RP-HPLC)

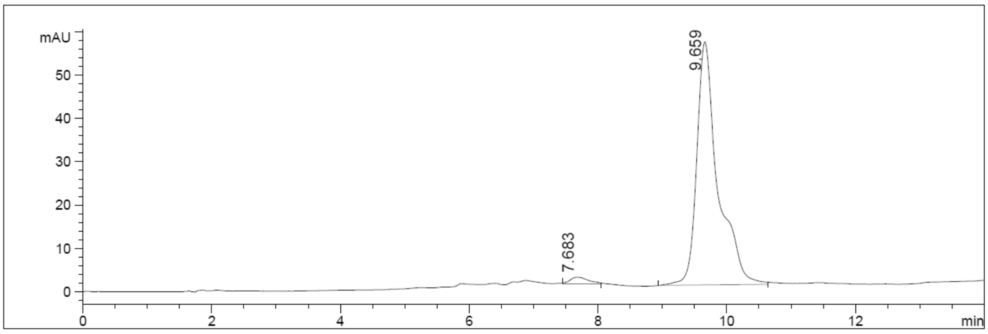

Peak list:

Ret. Time	Width min	Height	Area	Area %
7.683	0.340	1.575	32.166	2.444
9.659	0.382	56.083	1283.827	97.556

MALDI-MS spectrum of isolated product **54g**

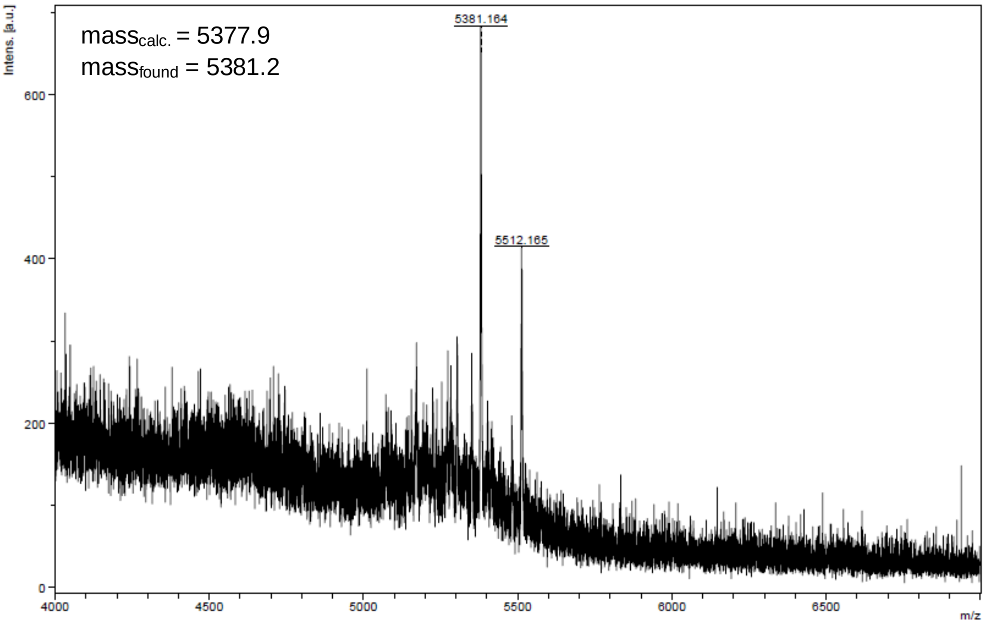

Boc cleavage in aqueous solution

DNA conjugate 32: CPG-bound 16mer 7De-dATC-Povarov conjugate **31** was Boc-deprotected according to RP-21.

HPLC trace of purified Boc-protected 16mer 7De-dATC-Povarov conjugate **31** (analytical RP-HPLC)

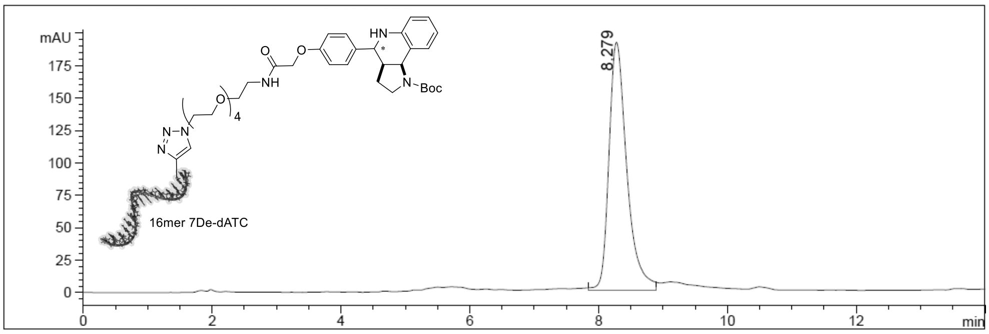

Peak list:

Ret. Time	Width min	Height	Area	Area %
8.279	0.319	190.998	3655.817	100.000

HPLC trace of Boc-deprotected 16mer 7De-dATC-Povarov conjugate **32** (analytical RP-HPLC)

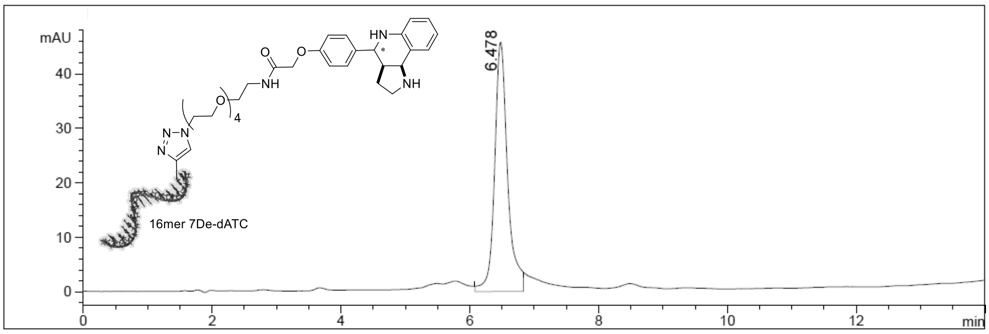

Peak list:

Ret. Time	Width min	Height	Area	Area %
6.478	0.209	45.772	639.304	100.000

MALDI-MS spectrum of isolated product **32**

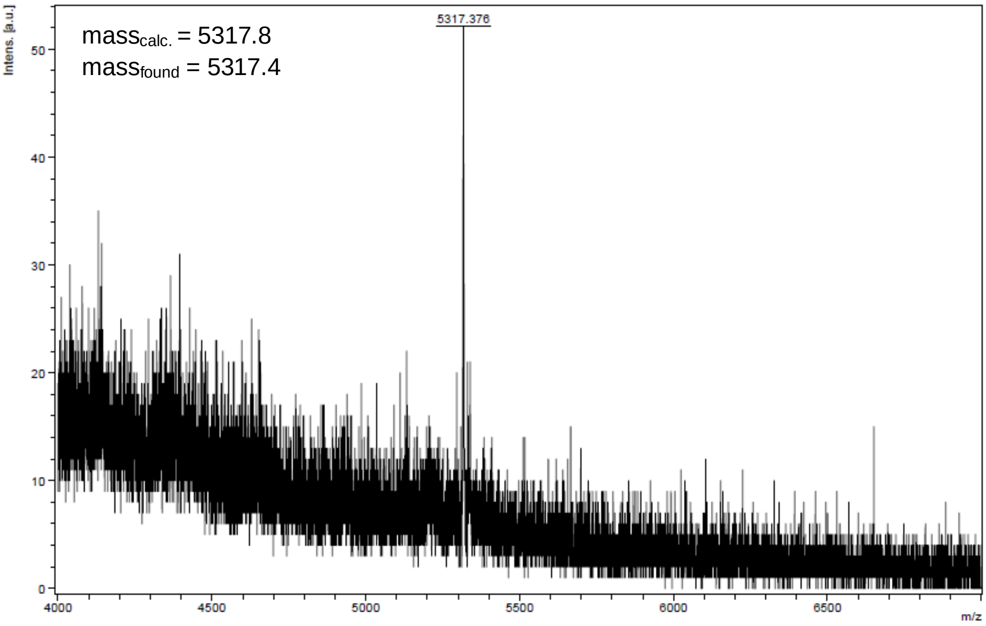

DNA conjugate 55: CPG-bound 16mer 7De-dATC-U-4CR/aza-Wittig conjugate **25d** was Boc-deprotected according to RP-21.

HPLC trace of purified Boc-protected 16mer 7De-dATC-U-4CR/aza-Wittig conjugate **25d** (analytical RP-HPLC)

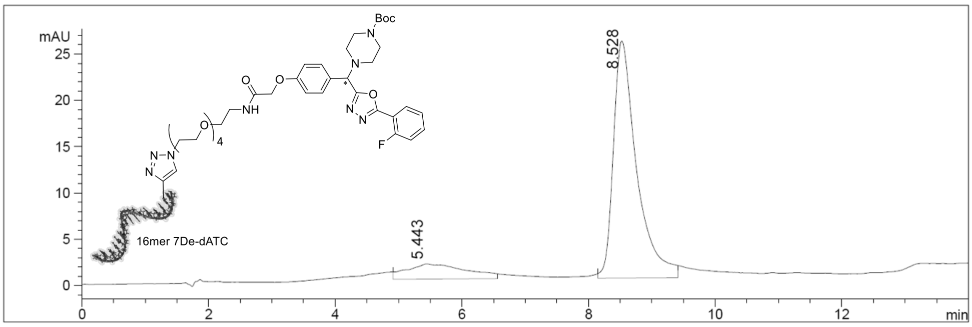

Peak list:

Ret. Time	Width min	Height	Area	Area %
5.443	1.100	1.640	108.212	14.166
8.528	0.427	25.594	655.668	85.834

HPLC trace of purified Boc-deprotected 16mer 7De-dATC-U-4CR/aza-Wittig conjugate **55** (analytical RP-HPLC)

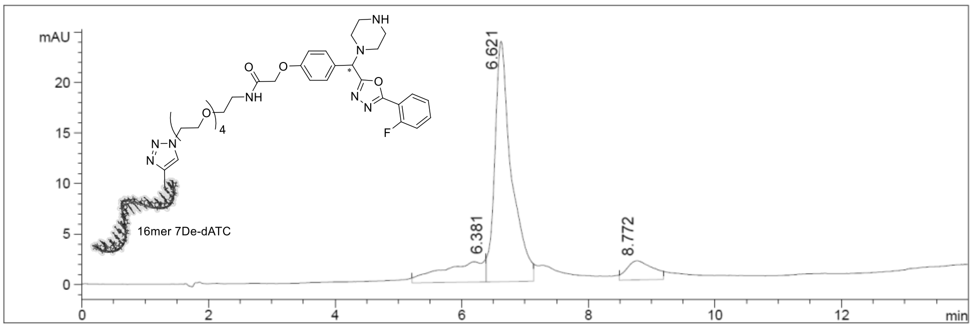

Peak list:

Ret. Time	Width min	Height	Area	Area %
6.381	0.712	2.281	97.477	16.606
6.621	0.305	23.808	434.980	74.101
8.772	0.476	1.910	54.555	9.294

MALDI-MS spectrum of reaction crude **55**

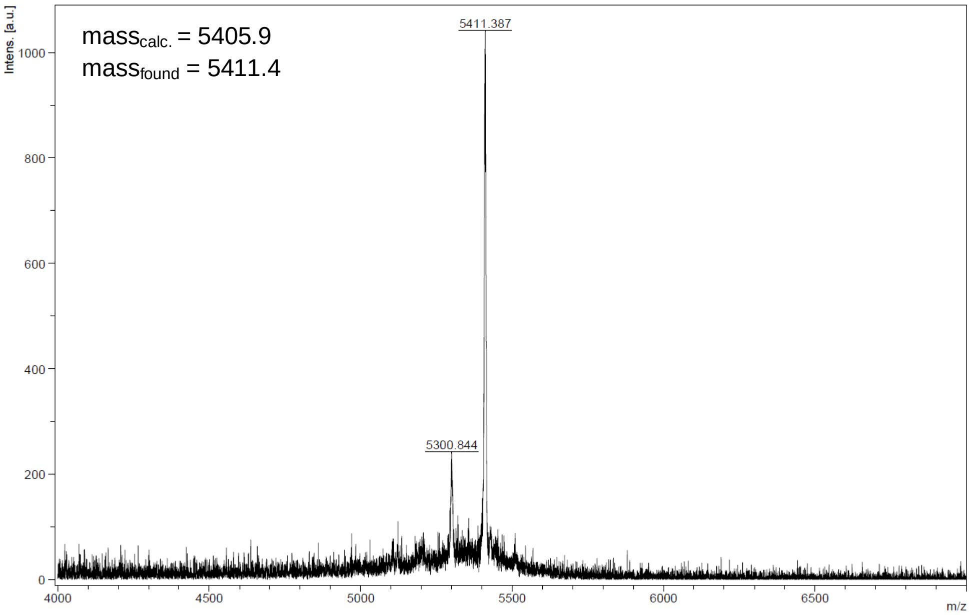

qPCR analysis after treating DNA with common DEL synthesis methods

According to the above-mentioned protocols for ligation experiments, the stabilized or native barcode *If* were ligated with the hairpin-DNA *HP* as well as with the counter code *I'* and the duplex DNAs *II/II'* and *III/III'* (Figure S12). Afterwards 80 pmol of the gel extracted ligation products were treated with commonly used reaction conditions in DEL synthesis (Table S17). The Suzuki reaction was purified by treatment with 1,3,5-triazine-2,4,6-trithiol trisodium salt solution (15% in H_2O) for 1 h, subsequently centrifuged and then the supernatant was precipitated with ethanol. The amide coupling was purified by ethanol precipitation.

For qPCR experiments the following were combined in PCR plate wells (GK480K-50, *Kisker*) in a total volume of 20 µL: DNA template (5 µL, ligation product 3 after treatment with different reaction conditions), 200 nM forward primer (0.8 µL, 5 µM stock), 200 nM reverse primer (0.8 µL, 5 µM stock), SsoAdvanced universal SYBR® Green supermix (10 µL, *Bio-Rad*) and H_2O (3.4 µL). For all qPCR experiments the following amplification method using the *CFX Connect Real-Time PCR System* from *Bio-Rad* was performed: hot start at 95 °C for 30 s, then 35 cycles of 95 °C for 15 s (denaturation), 60 °C for 30 s (annealing) and 72 °C for 30 s (elongation).

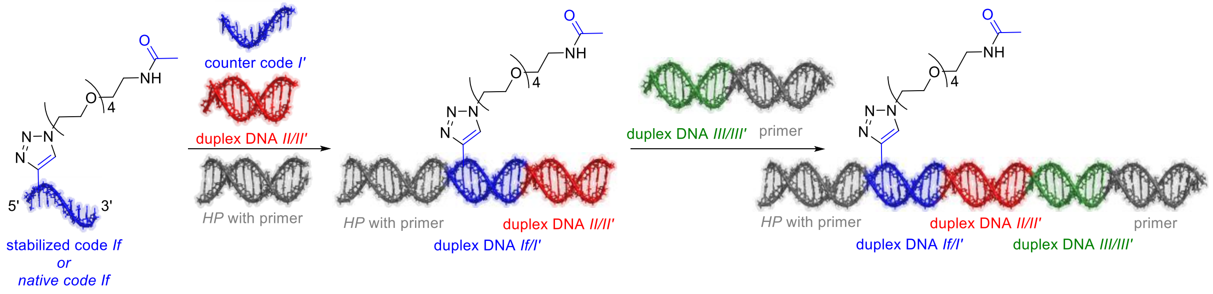

Figure S11 – Encoding scheme for test ligations with chemically stabilized/native barcode *If*.

Table S17 – Investigated DEL synthesis methods.

Reaction	Conditions
Amide coupling	R-COOH, EDC·HCl, HOAt, DIPEA, rt, 18 h[29]
Suzuki reaction	$R\text{-}B(OH)_3$, $Pd(PPh_3)_4$, Na_2CO_3, 80 °C, 1.5 h[33]

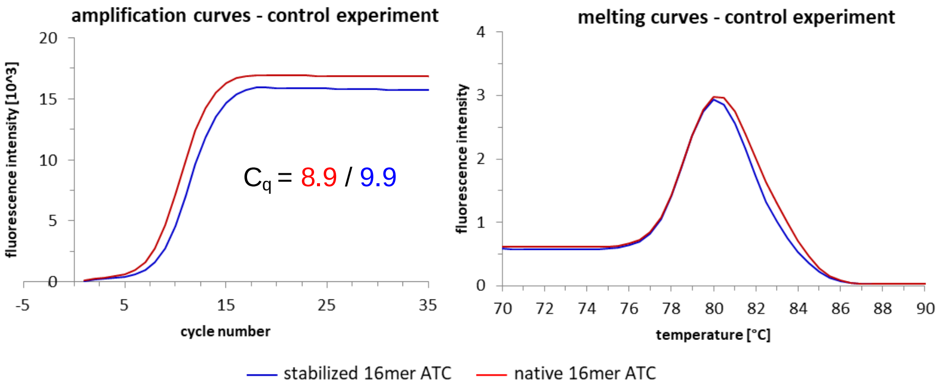

Figure S12 – Amplification and melting curves (qPCR) of the ligation product containing stabilized DNA barcode *If* or its native analogue (5'-CT*C TCT TT7De-dA 7De-dACT 7De-dACC T-3').

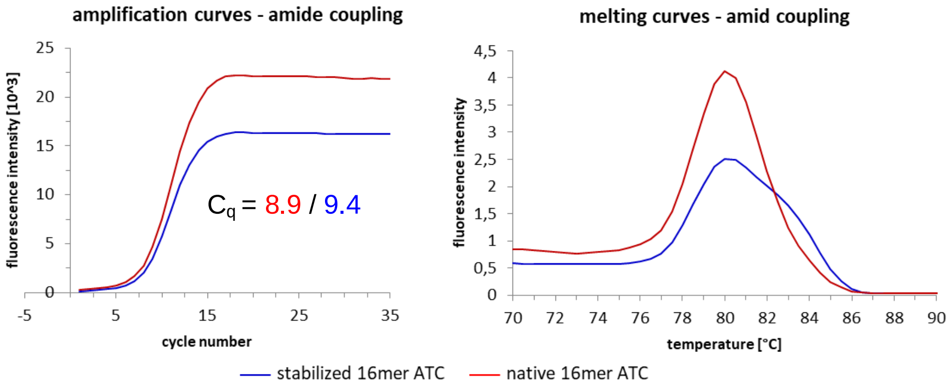

Figure S13 – Amplification and melting curves (qPCR) of the ligation product containing stabilized DNA barcode *If* or its native analogue (5'-CT*C TCT TT7De-dA 7De-dACT 7De-dACC T-3') after treatment with amide coupling conditions.

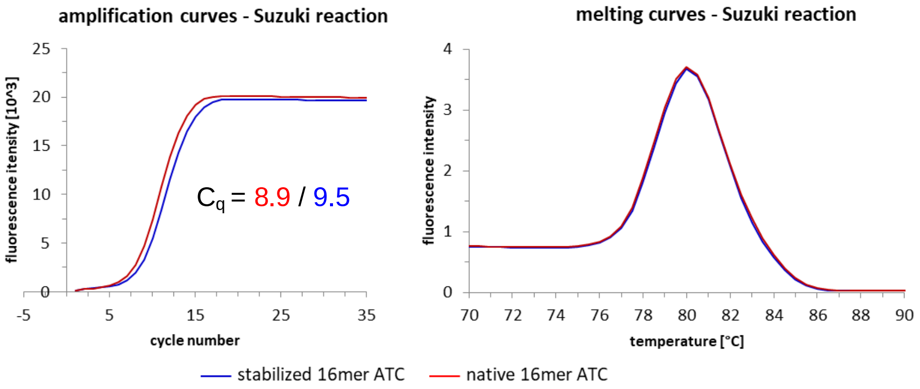

Figure S14 – Amplification and melting curves (qPCR) of ligation product containing stabilized DNA barcode *If* or its native analogue (5'-CT*C TCT TT7De-dA 7De-dACT 7De-dACC T-3') after treatment with Suzuki reaction conditions.

Practical aspects of DEL synthesis initiated with CPG-bound stabilized DNA barcodes

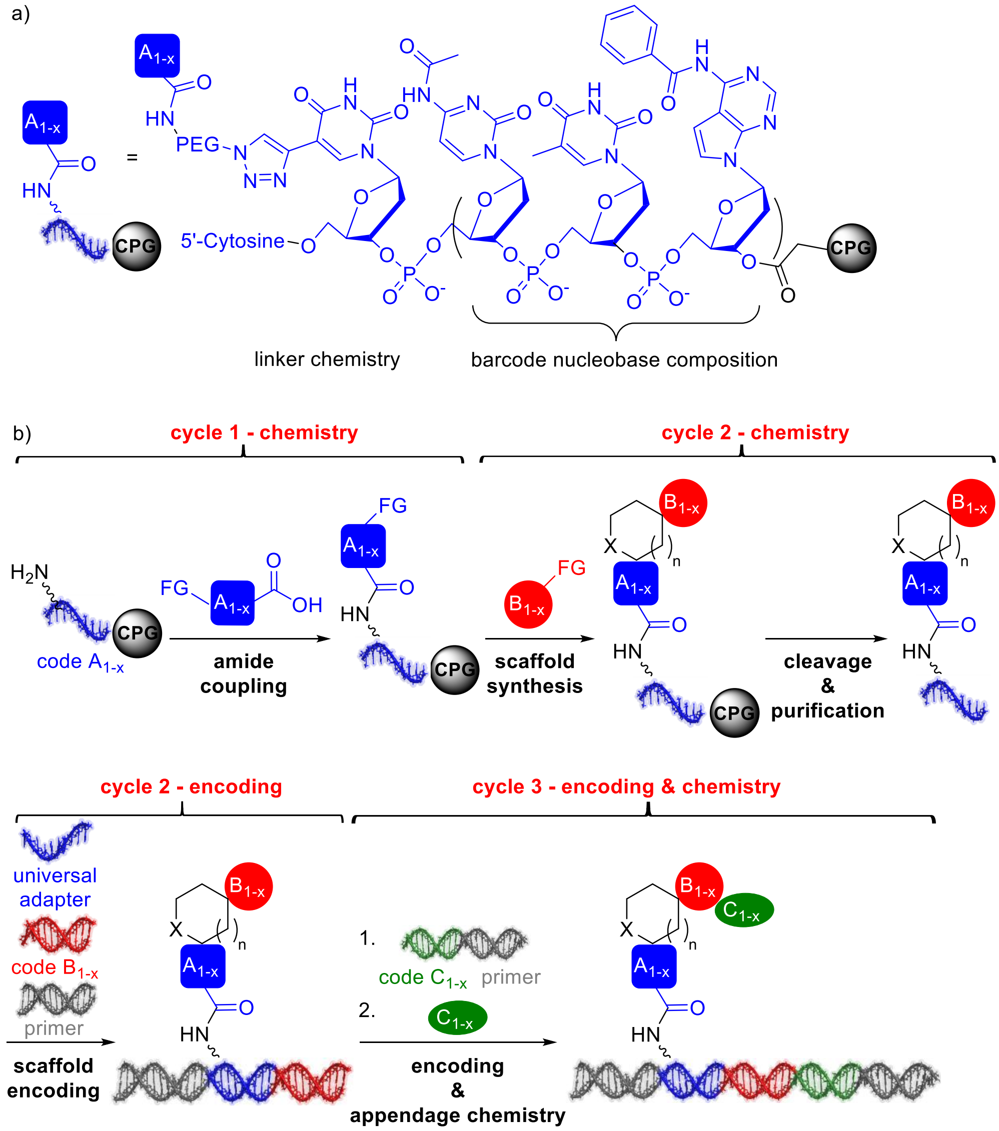

Figure S15 – Barcoding strategy using CPG-coupled chemically stabilized barcodes. a) Structure of the nucleobase-protected DNA-barcode. b) DEL synthesis strategy.

Phosphoramidite building block and DNA synthesis

The 2'-deoxy-7-deaza-adenine phosphoramidite building block is either commercially available or can be prepared in three high-yielding steps from the readily available nucleoside which is offered by several vendors in multi-gram quantities. All DNA barcodes were synthesized by standard phosphoramidite DNA chemistry requiring 30 µmol of the phosphoramidite (ca. 25 mg) per coupling. The yields did not differ from standard DNA synthesis. The cost of a single barcode composed of T, C and 7-deazaA and the alkyne linker is ca. 3-4 times higher than native DNA. This calculation includes five of the 7-deazaA on average and considers that larger libraries will require longer barcodes of 10 or

12 nucleobases if the Hamming distance is set to two or three. An amount of 1 micromol of DNA is sufficient for 200 scaffold-forming reactions on an encoded building block (see below).

Barcode design

A sequence designer programmed with R allows for design of 8-12mer ATC-barcodes with selectable maximal number of individual nucleobases, homomeric strings of individual nucleobases, and both Hamming and Levenshtein distances. This allows for design of hundreds of 8mer barcodes with a Hamming distance of 2 or 1000 s of 10mer/12mer barcodes with a Hamming distance of 2.

DEL synthesis

The reactions shown in this work were all performed with 20-30 nmol of CPG. In a library synthesis we mix 4 nmol aliquots from a 1 micromol DNA-batch for subsequent pooled on-DNA reactions, and that we pool 25 CPG-coupled encoded building blocks with high fidelity. Thus, 200 reactions can be performed on one batch of the DNA-barcode, and each with e.g. 25 building blocks in one run. The hybrid solid phase/solution phase workflow enables library sizes of 105-107 compounds in one synthesis campaign. For instance, reacting 50 cycle 1 BBs on chemically modified DNA barcodes x 200 diverse cycle 2 scaffold-forming reactions in two reaction batches will lead to 10.000 cycle 1-encoded scaffold-diverse molecules. The products are isolated following extensive washing to remove reagent excess by automated HPLC according to their retention time. Unreacted starting materials and DNA damage products are in our experience eluting early and therefore removed from the product pool in this purification step. Following DNA ligation for encoding cycle 2-chemistry, the library can e.g. be reacted with 100 cycle 3 BBs yielding a 1 mio DEL. Library sizes in the 105-107 range from both academic and company DEL research groups have shown to be highly productive numerous times. The CPG strategy has some limitations: Reactions that require strong inorganic bases are beyond the scope of the CPG-strategy as they cleave the DNA from the solid phase, strong nucleophiles such as hydrazines (see pyrazole synthesis in this work) damaged the DNA, and both Michael acceptors and methyl esters were unstable to the cleavage cocktail as shown in previous publications. However, ethyl ester building blocks were perfectly stable and can be used for cycle 3 chemistry. For reading out the sequencing data we use an algorithm that accounts for differences in amplification efficiency by calculating enrichment factors. The details can be found in Kunig *et al*, *Angew. Chem. Int Ed.* **2019**.[34]

References

[1] Ingrid Luyten, Piet Herdewijn, *Eur. J. Med. Chem.* **1998**, *33*, 515-576.

[2] Patrick C. Newman, Victor U. Nwosu, David M. Williams, Richard Cosstick, Frank Seela, and Bernard A. Connolly, *Biochemistry* **1990**, *29*, 9891-9901.

[3] E. A. Kowal, M. Ganguly, P. S. Pallan, L. A. Marky, B. Gold, M. Egli, M. P. Stone, *J. Phys. Chem. B* **2011**, *115*, 13925-13934.

[4] X. Peng, H. Li, F. Seela, *Nucleic Acids Res.* **2006**, *34*, 5987-6000.

[5] A. Hottin, A. Marx, *Acc. Chem. Res.* **2016**, *49*, 418-427.

[6] S. Jäger, G. Rasched, H. Kornreich-Leshem, M. Engeser, O. Thum, M. Famulok, *J. Am. Chem. Soc.* **2005**, *127*, 15071-15082.

[7] E. Eremeeva, M. Abramov, P. Marlière, P. Herdewijn, *Org. Biomol. Chem.* **2017**, *15*, 168-176.

[8] C. J. Whitfield, R. C. Little, K. Khan, K. Ijiro, B. A. Connolly, E. M. Tuite, A. R. Pike, *Chem. Eur. J.* **2018**, *24*, 15267-15274.

[9] M. Ondruš, V. Sýkorová, L. Bednárová, R. Pohl, M. Hocek, *Nucleic Acids Res.* **2020**, *48*, 11982-11993.

[10] L. Eberlein, F. R. Beierlein, N. J. R. van Eikema Hommes, A. Radadiya, J. Heil, S. A. Benner, T. Clark, S. M. Kast, N. G. J. Richards, *J. Chem. Theory Comput*. **2020**, 16, 2766-2777.

[11] M. J. Frisch, G. W. Trucks, H. B. Schlegel, G. E. Scuseria, M. A. Robb, J. R. Cheeseman, G. Scalmani, V. Barone, G. A. Petersson, H. Nakatsuji, X. Li, M. Caricato, A. V. Marenich, J. Bloino, B. G. Janesko, R. Gomperts, B. Mennucci, H. P. Hratchian, J. V. Ortiz, A. F. Izmaylov, J. L. Sonnenberg, D. Williams-Young, F. Ding, F. Lipparini, F. Egidi, J. Goings, B. Peng, A. Petrone, T. Henderson, D. Ranasinghe, V. G. Zakrzewski, J. Gao, N. Rega, G. Zheng, W. Liang, M. Hada, M. Ehara, K. Toyota, R. Fukuda, J. Hasegawa, M. Ishida, T. Nakajima, Y. Honda, O. Kitao, H. Nakai, T. Vreven, K. Throssell, J. A. Montgomery, Jr., J. E. Peralta, F. Ogliaro, M. J. Bearpark, J. J. Heyd, E. N. Brothers, K. N. Kudin, V. N. Staroverov, T. A. Keith, R. Kobayashi, J. Normand, K. Raghavachari, A. P. Rendell, J. C. Burant, S. S. Iyengar, J. Tomasi, M. Cossi, J. M. Millam, M. Klene, C. Adamo, R. Cammi, J. W. Ochterski, R. L. Martin, K. Morokuma, O. Farkas, J. B. Foresman, D. J. Fox, *Gaussian 16*, Rev. C.01, Gaussian Inc., Wallingford CT, 2016.

[12] F. Neese, *WIREs Comput. Mol. Sci.* **2012**, 2, 73-78.

[13] F. Neese, *J. Comput. Chem*. **2003**, 24, 1740-1747.

[14] F. Pavošević, P. Pinski, C. Riplinger, F. Neese, E. F. Valeev, *J. Chem. Phys*. **2016**, 144, 144109.

[15] T. Kloss, J. Heil, S. M. Kast, *J. Phys. Chem. B* **2008**, 112, 4337-4343.

[16] T. Pongratz, P. Kibies, L. Eberlein, N. Tielker, C. Hölzl, S. Imoto, M. Beck Erlach, S. Kurrmann, P. H. Schummel, M. Hofmann, O. Reiser, R. Winter, W. Kremer, H. R. Kalbitzer, D. Marx, D. Horinek, S. M. Kast, *Biophys. Chem*. **2020**, 257, 106258.

[17] N. Tielker, L. Eberlein, S. Güssregen, S. M. Kast, *J. Comput.-Aided Mol. Des*. **2018**, 32, 1151-1163.

[18] S. M. Kast, T. Kloss, *J. Chem. Phys*. **2008**, 129, 236101.

[19] J. Wang, R. M. Wolf, J. W. Caldwell, P. A. Kollman, D. A. Case, *J. Comput. Chem.* **2004**, 25, 1157-1174.

[20] J. Wang, W. Wang, P. A. Kollman, D. A. Case, *J. Mol. Graph. Model*. **2006**, 25, 247-260.

[21] M. J. Frisch, G. W. Trucks, H. B. Schlegel, G. E. Scuseria, M. A. Robb, J. R. Cheeseman, G. Scalmani, V. Barone, B. Mennucci, G. A. Petersson, H. Nakatsuji, M. Caricato, X. Li, H. P. Hratchian, A. F. Izmaylov, J. Bloino, G. Zheng, J. L. Sonnenberg, M. Hada, M. Ehara, K. Toyota, R. Fukuda, J. Hasegawa, M. Ishida, T. Nakajima, Y. Honda, O. Kitao, H. Nakai, T. Vreven, J. A. Montgomery, Jr., J. E. Peralta, F. Ogliaro, M. Bearpark, J. J. Heyd, E. Brothers, K. N. Kudin, V. N. Staroverov, R. Kobayashi, J. Normand, K. Raghavachari, A. Rendell, J. C. Burant, S. S. Iyengar, J. Tomasi, M. Cossi, N. Rega, J. M. Millam, M. Klene, J. E. Knox, J. B. Cross, V. Bakken, C. Adamo, J. Jaramillo, R. Gomperts, R. E. Stratmann, O. Yazyev, A. J. Austin, R. Cammi, C. Pomelli, J. W. Ochterski, R. L. Martin, K. Morokuma, V. G. Zakrzewski, G. A. Voth, P. Salvador, J. J. Dannenberg, S. Dapprich, A. D. Daniels, Ö. Farkas, J. B. Foresman, J. V. Ortiz, J. Cioslowski, D. J. Fox, *Gaussian 09*, Rev. E.01, Gaussian Inc. Wallingford CT, **2009**.

[22] H. J. C. Berendsen, J. R. Grigera, T. P. Straatsma, *J. Phys. Chem*. **1987**, 91, 6269-6271.

[23] L. Martínez, R. Andrade, E. G. Birgin, J. M. Martínez, *J. Comput. Chem*. **2009**, 30, 2157-2164.

[24] J. C. Phillips, R. Braun, W. Wang, J. Gumbart, E. Tajkhorshid, E. Villa, C. Chipot, R. D. Skeel, L. Kalé, K. Schulten, *J. Comput. Chem*. **2005**, 26, 1781-1802.
[25] J. Imig, A. Brunschweiger, A. Brümmer, B. Guennewig, N. Mittal, S. Kishore, P. Tsikrika, A. P. Gerber, M. Zavolan, J. Hall, *Nat. Chem. Biol.* **2015**, *11*, 107-114.
[26] V. B. K. Kunig, C. Ehrt, A. Dömling, A. Brunschweiger, *Org. Lett.* **2019**, *21*, 7238-7243.
[27] M. Potowski, F. Losch, E. Wünnemann, J. K. Dahmen, S. Chines, A. Brunschweiger, *Chem. Sci.* **2019**, *10*, 10481-10492.
[28] M. Potowski, V. B. K. Kunig, F. Losch, A. Brunschweiger, *Med. Chem. Commun.* **2019**, *10*, 1082-1093.
[29] M. Klika Škopić, H. Salamon, O. Bugain, K. Jung, A. Gohla, L. J. Doetsch, D. dos Santos, A. Bhat, B. Wagner, A. Brunschweiger, *Chem. Sci.* **2017**, *8*, 3356-3361.
[30] M. Potowski, R. Esken, A. Brunschweiger, *Bioorg. Med. Chem.* **2020**, *28*, 115441.
[31] L. Wang, J. Han, J. Sheng, H. Tian, Z. Fan, *Catal. Commun.* **2005**, *5*, 201-204.
[32] M Klika Škopić, S. Willems, B. Wagner, J. Schieven, N. Krause, A. Brunschweiger, *Org. Biomol. Chem.* **2017**, *15*, 8648–8654.
[33] M. L. Malone,B. M. Paegel, *ACS Comb. Sci.* **2016**, *18*, 182−187.
[34] V. B. K. Kunig, M. Potowski, M. Akbarzadeh, M. Klika Škopić, D. Dos Santos Smith, L. Arendt, I. Dormuth, H. Adihou , B. Andlovic, H. Karatas, S. Shaabani, T. Zarganes-Tzitzikas, C. G Neochoritis, R. Zhang, M. Groves, S. M Guéret, C. Ottmann, J. Rahnenführer, R. Fried, A. Dömling, A. Brunschweiger, *Angew. Chem. Int. Ed.* **2020**, *59*, 20338-20342.
